# Full-length merozoite surface protein 1 formulated with GLA-SE adjuvant in malaria pre-exposed adults: a randomised, controlled, double-blind, parallel-group, single-centre Phase Ib trial

**DOI:** 10.1016/j.eclinm.2025.103585

**Published:** 2025-10-25

**Authors:** Aina-ekisha Kahatano, Maxmillian Mpina, Fiona Vanobberghen, Omary Hassan, Nsiande Urasa, Ibrahim Sasamalo, Sarah Mswata, Daniel Paris, Suzanne Gajewski, Meera Saxena, Kristin Fürle, Viktoria Kiehl, Ernst Böhnlein, Andrea Aschenbrenner, Michael Lanzer, Richard Thomson-Luque, Ally Olotu, Claudia Daubenberger

**Affiliations:** a Biomedical Research and Clinical Trials Department, Ifakara Health Institute, Tanzania; b Swiss Tropical and Public Health Institute, Allschwil, Switzerland; c University of Basel, Basel, Switzerland; d Sumaya-Biotech GmbH & Co. KG, Heidelberg, Germany; e Centre for Infectious Diseases, Parasitology, Heidelberg University, Medical Faculty, University Hospital Heidelberg, Heidelberg, Germany

**Keywords:** *Plasmodium falciparum*, Full-length merozoite surface protein 1, 3D7 strain, GLA-SE adjuvant, Subunit vaccine, Tanzania

## Abstract

**Background:**

A highly effective blood-stage malaria vaccine targeting the merozoite surface protein 1 (MSP1) of *Plasmodium falciparum* (Pf) might complement the imperfect protection conferred by the currently available first-generation pre-erythrocytic malaria vaccines RTS,S/AS01E and R21/Matrix-M. We investigated for the first time the safety, reactogenicity and immunogenicity of full-length recombinant PfMSP1 (MSP1_FL_) formulated in Glucopyranosyl Lipid Adjuvant-Stable Emulsion (GLA-SE) adjuvant as a malaria vaccine in a malaria pre-exposed adult population.

**Methods:**

We conducted a randomized, controlled, double-blind, parallel-group, single-centre Phase Ib trial in healthy participants living in a malaria-endemic setting. The trial was conducted in Bagamoyo, Tanzania between August 2023 and April 2024 and was the first study of this vaccine formulation in malaria pre-exposed participants. Forty participants were enrolled into two groups (n = 20). Eligible males and females aged 18–45 years were randomised (1:1) to receive 3 monthly doses of either the SUM-101 malaria vaccine (150 μg MSP1 _FL_ derived from the 3D7 Pf strain formulated in 5 μg of adjuvant GLA-SE) or the Verorab rabies vaccine (control) given intramuscularly at days 0, 28 and 56. Participants were monitored for solicited adverse events up to day 7 and unsolicited adverse events up to day 28 post-vaccination through clinical observation and laboratory assessments, including haematology and blood biochemistry. The primary safety and reactogenicity outcomes were (i) local and systemic solicited adverse events (AEs) at least possibly related to SUM-101 or Verorab over 7 days after each vaccination, (ii) local and systemic unsolicited reactogenicity over 28 days after each vaccination, (iii) any severe adverse event (SAE) occurring after the first vaccination until the participant's last visit, (iv) changes in clinical laboratory safety parameters from baseline (day 0) and up to 28 days post-vaccination, and (v) changes in clinical laboratory safety parameters from pre-vaccination time points (day 0, day 28 and day 56) to 28 days following each respective dose. The primary immunogenicity outcome was the magnitude and persistence of the vaccine-induced IgM and IgG responses to SUM-101, measured by ELISA as fold-change relative to the baseline. Immunogenicity was assessed by measuring longevity and fold changes (versus baseline) of MSP1-specific IgG and IgM antibody responses, IgG subclass distribution, and Fc-receptor mediated effector functions. All participants were followed for 140 days after first dose. The trial is registered on ClinicalTrials.gov under NCT05644067.

**Findings:**

Out of the 40 enrolled participants, 37 (93%) completed all vaccinations and follow-up procedures. Within 7 days post-vaccination, 52 solicited adverse events were reported that were at least possibly related to the Investigational Medicinal Product (IMP): 26 events occurred in 10 participants (50%) in the SUM-101 arm, and 26 events occurred in 13 participants (65%) in the Verorab arm. The majority of the solicited adverse events were mild to moderate in severity, including injection site pain, headache, and fatigue. No severe solicited adverse events, serious adverse events (SAEs), or unsolicited vaccine-related adverse events were reported. SUM-101 elicited robust MSP1-specific IgM and IgG antibody titers, with peak levels observed on day 56 and day 84, respectively. IgM antibody titers increased following each dose, peaking on day 56 with a median fold-change from baseline of 2.65 (Inter-Quartile Range (IQR): 0.80–5.10), and gradually declined after day 84 but remained elevated through day 140. IgG titers rose progressively, peaking on day 84 with a median fold-change of 20.77 (IQR: 2.60–45.29), and remained above baseline on day 140 (median 10.77, IQR: 1.98–27.07). All IgG subclasses (IgG1, IgG2, IgG3, and IgG4) exhibited increased titers. The median (IQR) IgG titers at baseline were 504 (143–1055) and 413 (239–2310) AU in the Verorab and SUM-101 groups, respectively, and the corresponding values for IgM titers were 61 (34–128) and 57 (26–95) AU. On day 84, MSP1-specific antibodies mediated Fc-receptor-dependent phagocytosis, respiratory burst, natural killer cell, and complement system activation at their highest levels compared to baseline. SUM-101 induced strain-transcending antibodies binding merozoites from Pf3D7 and PfFCB1 strains. Participants with higher pre-vaccination MSP1-specific IgG levels reached peak responses after two vaccinations, indicating that SUM-101 boosts pre-existing, naturally acquired malaria immunity. In the Verorab arm, the median values of the MSP1-specific IgG and IgM antibody titers remained stable from baseline up to day 140.

**Interpretation:**

Vaccination with SUM-101 was safe and well-tolerated among adults living in a malaria-endemic region of Tanzania. Our findings justify further development of SUM-101 as component of next-generation subunit malaria vaccines.

**Funding:**

Sumaya Biotech GmbH & Co. KG, Heidelberg, funded this study partly through support by a Venture Loan Agreement to Sumaya Biotech GmbH & Co. KG by the EU Malaria Fund Berlin GmbH & Co. KG.

**Research in context**

**Evidence before this study**

A highly effective and durable vaccine against the most deadly human malaria parasite, *Plasmodium falciparum*, is urgently needed. A multi-stage vaccine, combining blood-stage-targeting vaccines with the approved pre-erythrocytic stage vaccines RTS,S/AS01, or R21/Matrix-M, is a promising strategy to achieve this target. However, the identification of suitable asexual blood-stage vaccine candidates, followed by their GMP-compliant production and clinical development, has proven challenging.

We searched PubMed for research articles using the terms “malaria” AND “blood stage” AND “vaccine” AND “clinical study”. No date or language filters were applied. We found the following blood-stage vaccine candidates as adjuvanted recombinant proteins or synthetic peptides evaluated in Phase I and Phase II studies: merozoite surface protein 1, merozoite surface protein 2, merozoite surface protein 3, glutamate-rich protein, apical merozoite antigen 1, reticulocyte-binding protein homolog 5, VAR2CSA, serine repeat antigen 5, sporozoite threonine-asparagine-rich protein, liver-stage antigen 1, exported protein 1, PfF2, multiple epitope thrombospondin-related adhesion protein (ME-TRAP), erythrocyte binding antigen-175 (EBA-175) Pf11.1, P27A, SPf66, and Combination B. RH5 is the only candidate so far that has moved into phase 2 field studies resulting in interim results of 55% (95% CI: 20–75%) protection against clinical malaria in Burkinabe children.

Full-length merozoite surface protein 1 (MSP1FL) is rich in B- and T-cell epitopes, enhancing its potential to elicit cellular and humoral immune responses in human populations. In malaria monkey models, using the MSP1FL for immunization resulted in significant protection against live parasite challenge. In contrast, vaccinating with MSP1 carboxy-terminal subunits or composite formulations comprising a range of MSP1 domains resulted in poor performance in early-phase human trials. A recent, first-in-human Phase Ia trial conducted in malaria naïve volunteers showed the safety, tolerability, and immunogenicity of the MSP1FL-based vaccine formulated with the GLA-SE adjuvant. All vaccinated individuals seroconverted, and MSP-1 specific antibody levels were maintained for at least six months post-vaccination, indicative of potentially long-lasting immunity. These anti-MSP1 antibodies also displayed functional activities in several ex vivo Fc-receptor-dependent functional assays targeting merozoites.

**Added value of this study**

As a follow-up on the Phase Ia study, we report here on the first evaluation of MSP1FL/GLA-SE formulation for safety, tolerability, and immunogenicity in the target population of a malaria vaccine (Phase Ib). This vaccine candidate shows a favorable safety profile and high immunogenicity in healthy Tanzanian adults. By exploring the same vaccine dose, formulation, and regimen in malaria naïve and pre-exposed populations, we show that in Tanzanian participants, mainly long-lived pre-existing memory B cell responses (high IgG responses) were induced with limited induction of IgM responses (*de novo* induced antibody response). A range of Fc-receptor dependent effector mechanisms were observed at peak antibody responses in vaccinees including strain-crossreactive antibodies targeting merozoites.

**Implications of all the available evidence**

As a result of these data, MSP1FL/GLA-SE has progressed to an ongoing Phase Ib trial in 5–17-month-old children in Burkina Faso (NCT06618885) to assess the safety, tolerability and immunogenicity of this vaccine candidate for the first time in young African children. Also, understand the potential of SUM-101 to confer vaccine induced protection, a Phase Ib study in adult participants, (NCT07124156), is expected to start in Q3 2025 in Bagamoyo, Tanzania. This RCT study will include a controlled human malaria infection of viable intra-erythrocytic parasites of the 3D7 Pf strain.

## Introduction

Malaria continues to pose a significant threat to the well-being and economic prosperity of nearly half of the global population. In 2023, malaria claimed an estimated 597,000 lives, and approximately 263 million cases were reported.^1^ Malaria affects primarily vulnerable populations or those at the margins of society, including children under 5 years of age, pregnant women, the rural poor, migrants, refugees and indigenous peoples in malaria-endemic countries.^2^ The most severe and deadly form of malaria is caused by *Plasmodium falciparum* (Pf), a protozoan parasite transmitted by infected female Anopheline mosquitoes during blood meals. After asymptomatic multiplication in the liver (pre-erythrocytic stage), the parasite initiates a pathogenic cycle within red blood cells (asexual blood stage), potentially precipitating a range of disease manifestations, including prostrating febrile illness, anaemia, vaso-occlusive events, and, in severe cases, life-threatening dysfunction of vital organs such as the lungs, kidneys, and brain.^2^

The drive to eliminate malaria has been stymied by diminishing efficacy of chemotherapeutic treatments and vector control measures, partly due to the emergence of drug-resistant parasites and insecticide-resistant vectors.^1^ Between 2021 and 2023, the World Health Organization (WHO) endorsed the first two vaccines against Pf, reflecting decades of international research efforts.^3^^,^^4^ However, the partial protection provided by these pre-erythrocytic vaccines does not reach the desired threshold of >90% efficacy against Pf to enable malaria elimination.^5^ Moreover, their efficacy has been shown to wane over time. Developing a next-generation, blood-stage malaria vaccine that complements the currently introduced vaccines might enhance field efficacy against clinical disease and/or infection and/or extend its duration of protection.

The merozoite surface protein 1 (PfMSP1) is prominently displayed on the surface of merozoites—the invasive form of asexual blood stages.^6^ PfMSP1 is an essential protein during both liver and blood stage development, as demonstrated by failed knock-out experiments showing ablation of merozoite formation.^6^ PfMSP1 is structurally organized into 17 domains based on sequence polymorphisms. Among these, seven domains are highly polymorphic, five are semi-conserved, and five are highly conserved. Historically, MSP1 has been characterized by three prototypical sequence variants: MSP1-D from the PfMAD20 strain (as in the 3D7 clone) and MSP1-F from the WELLCOME strain (as in the FCB1 clone) and R033.^6^ Initially synthesized as a precursor of approximately 196 kDa, it undergoes processing by a subtilisin-like protease (PfSUB1) upon egress from the host erythrocyte, resulting in the generation of four noncovalently attached subunits with molecular weights of p83, p30, p38 and p42.^6^ SUM-101, as a candidate malaria vaccine, consists of full-length recombinant PfMSP1 (MSP1_FL)_ (1720 amino acids) derived from the 3D7 strain, manufactured recombinantly in *E. coli* as a “heterodimer” composed of two peptide chains corresponding to the N- and C-terminal fragments of MSP1, namely p83/30 and p38/42, respectively.^6^ This protein, MSP1_FL_, was administered together with Glucopyranosyl Lipid Adjuvant-Stable Emulsion (GLA-SE) as an adjuvant, which is a stable oil-in-water nano-emulsion of the toll-like receptor 4 (TLR4) agonist glucopyranosyl lipid A.^7^ A Phase Ia study conducted in adult malaria naïve Caucasian participants demonstrated the safety, tolerability, and immunogenicity of SUM-101, with all vaccinated individuals seroconverting, independent of the MSP1_FL_ dose given.^8^ Vaccine recipients maintained vaccine-induced antibody levels for at least five months post-vaccination, indicative of induction of potentially long-lasting immunity.^8^ These anti-MSP1 antibodies displayed Fc-receptor-dependent functions, including antibody-dependent respiratory burst, opsonic phagocytosis, natural-killer (NK) cell activation, complement C1q fixation and C3b and C5–C9 deposition.^9^ Crucially, vaccination with MSP1_FL_ demonstrated induction of strain-transcending functional antibody activity.^9^

Here we report on a Phase Ib clinical trial aimed to evaluate safety, reactogenicity and immunogenicity of SUM-101 in adults living in malaria-endemic areas. The primary objectives were (i) evaluation of safety and reactogenicity of SUM-101 in healthy adults of African origin previously exposed to the parasite, and (ii) assessment of the immunogenicity of SUM-101 in these individuals in comparison to the Verorab control vaccine. The secondary objective was to measure the SUM-101 vaccine-induced antibody levels and *in vitro* effector functions in participants who received SUM-101 versus Verorab control vaccine. The exploratory objective was to compare the distribution and duration of SUM-101 induced immunoglobulin isotypes between malaria pre-exposed and malaria naïve participants who received the same dosing and regimen of SUM-101 vaccination. Understanding the potential impact of malaria pre-exposure on the quality, quantity and duration of SUM-101 induced humoral immunity constitutes an essential step in its clinical development pathway.

## Methods

### Study design and participants

This was a randomized, controlled, double-blind, parallel-group, single-centre Phase Ib clinical trial conducted in a malaria-endemic region of Tanzania. The Ifakara Health Institute, Bagamoyo Clinical Trial Facility in Bagamoyo, Tanzania implemented the study between August 2023 and April 2024. Forty participants split into 2 groups of 20 participants, were randomized (1:1) to receive 3 doses of either the SUM-101 malaria vaccine or the Verorab rabies vaccine (control) on days 0, 28 and 56. For a sample size of 40, we would have 90% probability of observing at least one adverse event occurring at a frequency of 5.6% or higher. Group 1 contained a sentinel group of 3 participants. A safety monitoring committee (SMC) reviewed pre-defined no-go safety criteria (e.g. any SAE related to vaccination or any related AE grade 3 or higher persisting >24 or 48 h) after 48-h of the first vaccination in sentinel group. A second review of safety criteria was conducted after day 14 of first vaccination of the remaining group 1 participants. Vaccination of group 2 participants commenced after a positive opinion from the SMC. Healthy men and non-pregnant, non-breastfeeding women aged 18–45 years, residing in the study catchment area for at least two years and willing to practice strict contraceptive measures were eligible for inclusion. Participants were recruited through community-based sensitization meetings held in the township wards of Kiromo, Dunda, and Magomeni in Bagamoyo District with low (<5%) malaria prevalence in the study catchment area. All eligible participants were given an insecticide treated bed net and reminded at scheduled visits to use it. Main exclusion criteria included participation in a previous malaria vaccine trial in the last 3 years or previous rabies vaccination, body mass index of <18 or >30 kg/m^2^, malaria positivity, and significant clinical or laboratory findings suggestive of chronic disease. After obtaining informed consent, participants completed an assessment questionnaire to demonstrate literacy and comprehension of the study.

### Ethical approvals

The study was conducted in compliance with the Declaration of Helsinki (2013), ICH-GCP E6 (R2) and the national legal and regulatory requirements. The study was approved by the Ifakara Health Institute Review Board (Clinical trial protocol (CTP) v1.1: IHI/IRB/No:40-2022, CTP v2.0: IHI/IRB/AMM/NO:32-3023) and the National Health Research Ethics Sub-Committee (CTP v1.1: NIMR/HQ/R.8a/Vol.IX/4145, CTP v2.0: NIMR/HQ/R.8b/Vol.I/1200), and the Tanzania Medicines and Medical Devices Authority (CTP, v1.1: BC.69/96/56/2, CTP, v2.0: BC.69/96/56/08). In addition, the trial received a positive opinion from the Ethics Committee of Northern and Central Switzerland (CTP v1.1 and CTP v2.0: AO_2022-00060). Informed consent was obtained from all participants.

### Randomisation and masking

The randomisation sequence was computer-generated in three blocks for the sentinel group (n = 3), group 1 followers (n = 17) and the group 2 participants (n = 20). The block distribution was 2 (SUM-101): 1 (Verorab) for the sentinel group; 8 (SUM-101): 9 (Verorab) for group 1 followers; and 10 (SUM-101): 10 (Verorab) for group 2 participants, for an overall ratio of 1:1. The unblinded study pharmacist allocated participants to the treatment arm according to the randomisation list, in sequential order upon confirmation of their eligibility. The vaccines were prepared by the pharmacist in syringes that were covered to mask the contents. The participants, site staff (i.e. clinicians, nurses, laboratory personnel), the monitor(s), sponsor project team and the trial statistician were blinded to the treatment allocation.

### Procedures

The IMP, SUM-101, (150 μg MSP1 _FL_ lyophilized powder; Sumaya Biotech GmbH & Co. KG of Heidelberg, Germany) was re-constituted onsite with 250 μl of 0.9% sodium chloride and emulsified with 250 μl of GLA-SE adjuvant (5 μg; Access to Advanced Health Institute, Seattle, Washington, USA) to a final volume of 0.5 ml. Verorab (>2.5 IU; Sanofi Pasteur, Lyon, France) was prepared according to the manufacturer's instructions to a final volume of 0.5 ml. Both vaccines were administered intramuscularly into the deltoid muscle using masked syringes to maintain blinding of participants and vaccination team. Three vaccinations were given on day 0, day 28 and day 56. Participants remained under observation at the clinic for at least 2 h post-vaccination, followed by daily phone or home visits until day 6, and in-person visits on days 7, 14, and 28. Long-term follow-up visits were scheduled on day 112 and day 140 (4 and 5 months after 1st vaccination, respectively). During each on-site visit, investigators assessed the participant's clinical status, monitored adverse events and collected blood samples for biochemistry and haematology evaluation. Haematological parameters included haemoglobin, leucocytes, neutrophils, eosinophils, plates and haematocrits. Clinical chemistry included alanine transaminase, total bilirubin and creatinine at multiple time points (day 0, day 7, day 14, day 28, day 35, day 42, day 56, day 63, day 70, day 84, day 112 and day 140). Aspartate aminotransferase and blood glucose were assessed at baseline only. Blood samples for immunological assessments were collected on days 0 (before vaccination), day 28 and day 56, as well as on days 63, 84, 112 and 140. According to the same schedule, malaria testing was performed by quantitative polymerase chain reaction (qPCR) and thick blood smear (TBS) except at day 63. Vaccination decisions were based on concordant negative results from both microscopy and qPCR, which were available prior to vaccination and conducted with the same blood draw whereby the sample was split for qPCR and blood slide reading. Additionally, qPCR served as a confirmatory method for microscopy findings.

Any malaria-positive participant received treatment per national guidelines. Solicited local and systemic adverse events (AEs) were recorded for seven days following each vaccination. Unsolicited AEs were documented up to 28 days post-vaccination. All serious and non-serious AEs were recorded from the first dose until the end of the study and monitored until resolution (Supplementary Figure S1).

### Outcomes

The primary safety and reactogenicity outcomes were (i) local and systemic solicited adverse events (AEs) at least possibly related to SUM-101 or Verorab over 7 days after each vaccination, (ii) local and systemic unsolicited reactogenicity over 28 days after each vaccination, (iii) any severe adverse event (SAE) occurring after the first vaccination until the participant's last visit, (iv) changes in clinical laboratory safety parameters from baseline (day 0) and up to 28 days post-vaccination, and (v) changes in clinical laboratory safety parameters from pre-vaccination time points (day 0, day 28 and day 56) to 28 days following each respective dose. The primary immunogenicity outcome was the magnitude and persistence of the vaccine-induced IgM and IgG responses to SUM-101, measured by ELISA as fold-change relative to the baseline. Secondary endpoints included characterization of antibody subclasses (IgG1, IgG2, IgG3 and IgG4). Fc-receptor dependent functional activities of antibodies included complement fixation (C1q), deposition (C3b), membrane attack complex formation (C5–C9), respiratory burst levels (ADRB), opsonic phagocytosis activity (OPA), and NK-cell activation (Ab-NK) levels. For ELISA, serum samples were serially diluted and all assays were performed using established protocols given in detail in the Supplementary Methods.^8^^,^^9^ In addition, we compared antibody levels between participants in the current study and historical data obtained from malaria-naïve participants (N = 6) from Phase Ia study in Heidelberg who received the identical vaccine product, 150 μg MSP1_FL_ formulated with 5 μg GLA-SE.^8^ All ELISA and functional antibody analyses were conducted at the Centre for Infectious Diseases, Parasitology, Heidelberg University, Medical Faculty, University Hospital Heidelberg, Heidelberg, Germany.

### Statistical analysis

No formal sample size calculation was performed; the sample size of 40 participants (20 per treatment arm) was within range typically used in early-phase trials and considered sufficient for the evaluation of safety, reactogenicity, and immunogenicity of SUM-101 in adults of African origin living in a malaria-endemic setting. Adverse events were coded using the Medical Dictionary for Regulatory Activities (MedDRA) version 27.0 and are summarized by preferred terms (PT) grouped by primary system organ class (SOC). MSP1-specific IgG and IgM antibody titres were summarised over time as both absolute values and fold changes relative to baseline (determined as “(yi-y0)/y0” for measurement “y” at time “i” versus its value “y0” at baseline). Antibody titre results were reported as geometric means with 95% confidence intervals (CIs), calculated using a normal approximation after natural logarithmic transformation (with zero values imputed as 0.1 for transformation purposes). Fold changes were summarized using arithmetic means and corresponding 95% CIs. Where appropriate, results were illustrated graphically. No formal testing was performed between the arms due to the exploratory and descriptive nature of the study. Descriptive comparisons were made to data from previous Phase Ia study in malaria-naïve participants. Baseline characteristics were assessed in the intention-to-treat population, defined as randomized participants. Safety and immunology analyses were performed in the safety population, comprising participants who received at least one dose of study vaccine. Immunology analyses were also performed in the per protocol population, defined as participants who completed the study without any major or critical protocol violation which could impact on the primary outcome, as determined during blinded data review. Analyses were performed using Stata version 18.^10^

Post-hoc, we stratified SUM-101 study participants according to their baseline MSP1-specific IgM and IgG titres into two groups based on the median antibody titre to understand the impact of pre-existing humoral immunity on SUM-101 induced responses. In addition, a heat plot was generated to illustrate the breadth of Fc-receptor dependent functional antibody results for each participant calculated as fold change from baseline to day 84 (C1q results which were negative due to the subtraction of the background were set to 0.001) categorised into centiles of the observed data.

### Role of the funding source

Sumaya AG served as the industrial study sponsor of the study and employees of Sumaya AG participated in the study design, data analysis, data interpretation, report writing and in the decision to submit the paper for publication.

## Results

**Fig. 1:**
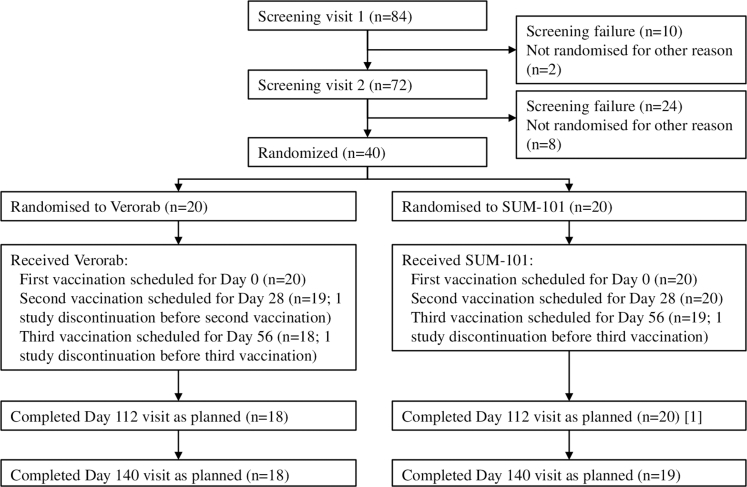
Trial profile.

Between August 30th and November 7th 2023, 84 adults were screened for eligibility; 50 met inclusion criteria and 40 were randomized in a 1:1 ratio to receive either SUM-101 (n = 20) or Verorab (n = 20) (Fig. 1). The most common reasons for ineligibility were abnormal electrocardiograms (n = 9) and malaria positivity (n = 7). All participants received vaccinations as scheduled, except two participants in the Verorab group (one participant was lost to follow-up after first vaccination, and another discontinued after second dose, missing subsequent visits). In the SUM-101 group, one participant was withdrawn after the second vaccination but attended the day 112 follow-up visit. All results are reported in the safety population, which includes all 40 randomized participants.

Baseline characteristics were generally comparable between groups (Table 1). The majority of participants were male (33 (83%)), with a median age of 25 years (IQR 22–29), and median body mass index (BMI) of 21 (IQR 20–24) kg/m^2^. All participants were of Black ethnicity. The SUM-101 group had a slightly higher proportion of males (95% versus 70%), a younger median age (24 versus 27 years), and lower median BMI (20 versus 23 kg/m^2^) compared to the Verorab group (Table 1). All seven female participants (1 and 6 in SUM-101 and Verorab group, respectively) were of childbearing potential and reported consistent use of contraception.

**Table 1:** Baseline characteristics of study participants.

	Verorab N = 20	SUM-101 N = 20	Total N = 40
Sex			
Female	6 (30%)	1 (5%)	7 (18%)
Male	14 (70%)	19 (95%)	33 (83%)
Age, years	27 (24–30)	24 (22–28)	25 (22–29)
Race, black	20 (100%)	20 (100%)	40 (100%)
BMI, kg/m^2^	23 (20–24)	20 (19–23)	21 (20–24)
Of child bearing potential (among females)	6/6 (100%)	1/1 (100%)	7/7 (100%)

Results are given as number, (%) or median (IQR).

Solicited local and systemic AEs considered at least possibly related to SUM-101 or Verorab vaccination and occurring within seven days post-dose were reported in 10 (50%) participants in the SUM-101 group and 13 (65%) participants in the Verorab group, with 26 events reported in each group. The most common AE in both arms was injection site pain (Table 2). All events were mild or moderate (Grade 1–2), and no Grade 3 or 4 events were observed (Supplementary Table S1). Most resolved within one–two days, and all participants continued as per protocol. No local and systemic unsolicited reactogenicity events were reported within 28 days after each vaccination in either study group. There were a further 23 AEs classified as other AEs (i.e., other than local and systemic solicited AEs assessed as IMP-related recorded up to seven days after each vaccination, and other than local and systemic unsolicited reactogenicity recorded up to 28 days after each vaccination): 12 in the SUM-101 group and 11 in the Verorab group, affecting 7 (58%) and 8 (73%) participants, respectively (Supplementary Tables S2 and S3). All were Grade 1 or 2, assessed as unrelated or unlikely related to study vaccination, resolved completely and did not impact continued participation as per protocol. One case of malaria occurred in the SUM-101 group 29 days after the first vaccination. The participant was treated on the same day and continued with the subsequent vaccinations as scheduled. No serious adverse events (SAEs) were reported during the study.

**Table 2:** Local and systemic adverse events at least possibly related to SUM-101 or Verorab over 7 days after each vaccination, by MedDRA system organ class and preferred term.

System organ class preferred term	Verorab group N = 20	SUM-101 group N = 20
**Any event, n (%) [m]**	**13 (65%) [26]**	**10 (50%) [26]**
**General disorders and administration site conditions**		
Vaccination site pain	9 (45%) [12]	10 (50%) [13]
Fatigue	3 (15%) [3]	2 (10%) [2]
Pyrexia	1 (5%) [1]	2 (10%) [2]
Chills	0	2 (10%) [2]
Vaccination site induration	0	1 (5%) [1]
Vaccination site pruritus	0	1 (5%) [1]
Vaccination site rash	1 (5%) [1]	0
**Nervous system disorders**		
Headache	4 (20%) [6]	1 (5%) [1]
**Gastrointestinal disorder**		
Vomiting	2 (10%) [2]	0
Nausea	0	1 (5%) [2]
Gastrointestinal disorder	0	1 (5%) [1]
**Musculoskeletal and connective tissue disorders**		
Arthralgia	0	1 (5%) [1]
**Skin and subcutaneous tissue disorders**		
Rash	1 (5%) [1]	0

Results are n = number of participants with at least one adverse event (%) and m = number of events.

Clinical laboratory evaluations did not indicate any clinically significant changes in biochemistry, haematology or urinalysis parameters (Supplementary Figures S2–S23). Assessments of vital signs (i.e. body weight, axillary body temperature, respiratory rate, heart rate and blood pressure) did not indicate any clinically significant abnormalities. All female participants underwent pregnancy testing prior to each vaccination and no pregnancies were noted throughout the study.

**Fig. 2:**
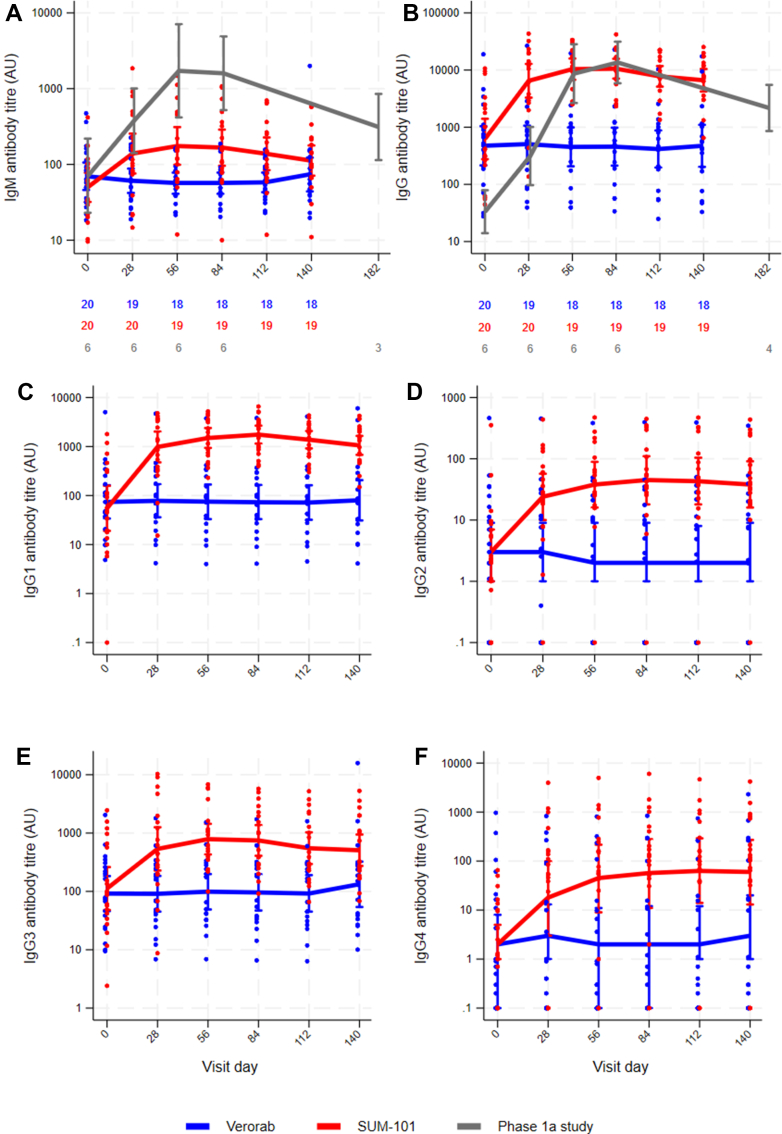
Antibody responses in Phase Ia and Phase Ib study participants measured against the Pf3D7 MSP1-D allelic variant. Results are given as geometric mean and 95% confidence interval. Dots represent individual participants. Y axes are on the log scale, and have different scales to best illustrate the data. Where applicable, numbers under the graphs indicate the number of participants contributing to each datapoint. (A, B) Total IgM (A) and IgG (B) antibody titres of Phase Ia and Phase Ib study participants. (C–F) IgG isotypes of Phase Ib study participants, (C) IgG1 isotype, (D) IgG2 isotype, (E) IgG3 isotype and (F) IgG4 isotype depicted of SUM-101 vaccinees versus Verorab controls.

At baseline, the geometric mean of the MSP1-specific IgM antibody titres was higher in the Verorab group (70 AU [95% CI: 46, 106]) compared to the SUM-101 group (50 AU [95% CI: 32, 78]) (Fig. 2A). In the Verorab arm, MSP1-specific IgM and IgG antibody titres remained stable throughout follow-up, and no overt fold changes from baseline were observed (Fig. 2A and B and Supplementary Figure S24). In contrast, participants receiving SUM-101 showed progressive increase in the geometric mean IgM titres following each of the first two monthly vaccinations and then declined slowly until day 140 (Fig. 2A, Supplementary Figure S24A and B). By day 28, the mean fold change from baseline was 4.25 (95% CI: 0.63, 7.87) increasing to peak on day 56 (one month after second vaccination) with mean fold change of 5.39 (95% CI: 2.10, 8.68). This response was maintained at day 84 (one month after third vaccination) with mean fold change of 4.92 [95% CI: 1.73, 8.11]). IgM titres decreased gradually thereafter but remained higher compared to baseline at day 112 (3.61 (95% CI: 1.10, 6.12), and day 140 (2.31 (95% CI: 0.93, 3.68) (Supplementary Figure S24A and B).

The geometric mean of MSP1-specific IgG titres at baseline was lower in the Verorab group (474 AU [95% CI: 215, 1043]) compared to the SUM-101 group (622 AU [95% CI: 276, 1405]) (Fig. 2B). In the SUM-101 group, IgG titres increased progressively following each vaccination and then declined slowly until day 140 (Fig. 2B, Supplementary Figure S24C and D). The mean fold change from baseline was 37.08 (95% CI: −0.95, 75.11) at day 28 rising to 77.04 (95% CI: 0.79, 153.3) at day 56. Peak responses were observed at day 84 (one month after third dose), with a mean fold change of 94.96 (95% CI: −14.18, 204.1). Although titres declined over time, they remained elevated compared to baseline, with mean fold changes of 55.11 (95% CI: 1.53, 108.7) at day 112 and 45.65 (95% CI: 1.28, 90.02) at day 140 (Supplementary Figure S24C and D).

Baseline MSP1-specific IgG titres were substantially higher among participants from Tanzania (geometric mean: 543 AU) compared to malaria-naïve participants from a previous Phase Ia study in Heidelberg (33 AU), consistent with prior natural exposure. Following vaccination with same SUM-101 formulation (150 μg MSP1 _FL_ + 5 μg GLA-SE), malaria-naïve individuals showed robust IgM and IgG responses, peaking one month after the third dose (geometric mean titres of 1595 AU for IgM and 13,578 AU for IgG; mean fold changes from baseline of 67 and 889, respectively). Interestingly, the malaria exposed participants from Tanzania showed sustained MSP1-specific IgG responses at later time points, despite a lower peak titer of 10759 AU for IgG at day 84. Three months after the final vaccination with SUM-101 (day 140), geometric mean IgG titres in Tanzanian participants remained elevated (6630 AU) compared to 2171 AU in malaria-naïve individuals at four months post-vaccination (day 182; Fig. 2A and B).

In the SUM-101 group, geometric mean titres of MSP1-specific IgG1, IgG2, IgG3 and IgG4 subclasses increased after vaccinations. For IgG1, the geometric mean titre was 55 AU at baseline and the highest value of 1750 AU was at day 84 (one month after the third vaccination). For IgG2, the geometric mean titre was 3 AU at baseline and the highest value of 45 AU was at day 84 (one month after the third vaccination). For IgG3, the geometric mean titre was 112 AU at baseline and the highest value of 787 AU was at day 56 (one month after second vaccination). For IgG4, the geometric mean titre was 2 AU at baseline and the highest value of 63 AU was at day 112 (two months after the third vaccination). In the Verorab arm, the geometric mean values of the MSP1-specific IgG1, IgG2 and IgG4 subclass titres remained broadly stable over time (Fig. 2C–F).

We stratified the SUM-101 recipients post-hoc into two groups based on the median baseline of MSP1-specific IgG and IgM titres. Participants with IgG antibody titres above the median peaked one month after the second vaccination (day 56), while those below the median peaked one month after the third vaccination (day 84). For IgM, both groups peaked at day 56 (Supplementary Figures S25 and S26).

**Fig. 3:**
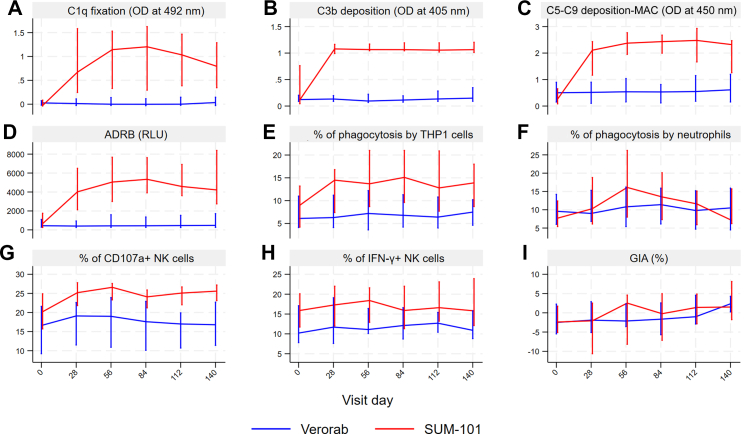
A–I: Fc-mediated effector mechanisms of vaccine-induced antibodies measured against the Pf3D7 MSP1-D allelic variant. (A–C) Levels of antibody-dependent complement activation measured by C1q fixation (A), C3b deposition (B) and C5–C9 deposition (membrane attack complex, MAC) (C). Levels of antibody-dependent respiratory burst (ADRB) of neutrophils (D). Levels of antibody-dependent opsonic phagocytosis activity (OPA) using MSP1-D-coupled microsphere beads by neutrophils (E) and THP1 cells (F). Antibody-dependent natural killer cell (Ab-NK) activity measured by degranulation (CD107a) (G) and IFN-γ production (H). Percentage of growth inhibition (I). For this GIA, we have not enriched for MSP1 binding antibodies from our serum samples before testing. Results are median and IQR.

SUM-101 induced high titres of MSP1-binding antibodies that stimulate different Fc-mediated immune effector mechanisms. We assessed classical complement system activation by C1q fixation (Fig. 3A), deposition by C3b (Fig. 3B) and membrane attack complex generation (C5–C9) (Fig. 3C). The serum antibodies induced activation of neutrophils to show respiratory burst activity (Fig. 3D) and induced phagocytosis by neutrophils (Fig. 3E) and THP1 cells (Fig. 3F). NK cells were stimulated resulting in enhanced expression of CD107a^+^ as marker of degranulation (Fig. 3G) and higher levels of IFN-γ production (Fig. 3H). No *in vitro* growth inhibition of asexual blood stage parasites was observed (Fig. 3I).

**Fig. 4:**
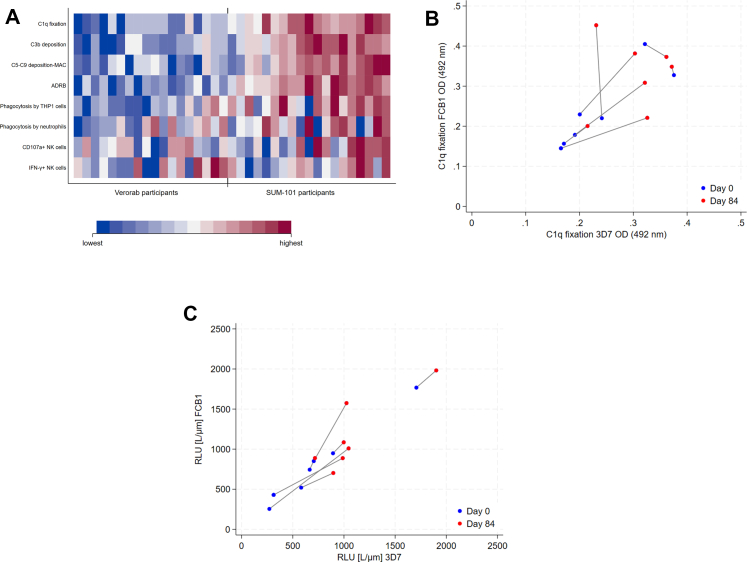
A: Heatmap of eight Fc-mediated effector functions assessed at day 84 in serum samples. Columns represent individual participants, while rows represent specific Fc-mediated functions. The quantity of the Fc-receptor mediated effector function increases in centiles from 1 to 100 and is demonstrated by darker shades of red (shown in the scale). B and C: Fc-mediated effector functions tested *in vitro* against merozoites of Pf strains Pf3D7 and PfFCB1 in C1q complement fixation (B) and ADRB (C) using serum samples collected on day 0 and day 84 in SUM-101 group participants.

The breadth of the combined Fc-mediated immune effector mechanisms group were compared between the Verorab group and the SUM-101 group (Fig. 4A). The SUM-101 group showed a higher activity level and breadth of different effector mechanisms compared to the Verorab group. Within the SUM-101 group, the breadth of the Fc-mediated immune effector mechanisms varied between participants as depicted in the heatmap (Fig. 4A).

We assessed complement fixation and respiratory burst using merozoites of two Pf strains, the homologous Pf3D7 strain, originating from West Africa and heterologous PfFCB1 strain, originating from Colombia.^11^ We selected sera collected at day 84 from SUM-101 vaccine recipients (n = 7) showing the strongest C1q fixation activity (fold change between day 0 and day 84 > 100) and evaluated C1q fixation against live merozoites. C1q fixation correlated positively between Pf3D7 and PfFCB1 merozoites confirming the functional activity against whole parasites of different genetic backgrounds (Fig. 4B). In addition, we also selected sera at day 84 from SUM-101 vaccine recipients (n = 7) with the strongest ADRB activity (fold change day 0 versus day 84 ADRB>20). ADRB was detected against Pf3D7 and PfFCB1 with positive correlation (Fig. 4C).

## Discussion

We report here on the outcome of a double-blind, placebo-controlled, Phase Ib study evaluating the safety, reactogenicity, and immunogenicity of SUM-101 in healthy adult volunteers living in a malaria-endemic region of Tanzania. The current study was the first in a population living in a malaria-endemic setting and the second study in which participants were administered this antigen and adjuvant combination.^8^ GLA-SE was selected based on its well-documented safety profile and its ability to enhance Th1-polarized CD4^+^ T-cell responses to co-administered antigens, particularly relevant as CD4^+^ T cells play a critical role in protective immunity against blood-stage malaria. Similar to the Phase Ia study, the safety results of the current study indicate that vaccination with SUM-101 is safe and well tolerated, with no patterns of AEs suggestive of off-target effects. There were no vaccine-related SAEs, and all reported AEs were mild or moderate in severity.

While peak IgG titers at one month after the last vaccination are comparable between the Phase Ia and Phase Ib volunteers, the calculated fold change is around 10 times lower in the malaria pre-exposed population based on higher baseline levels in pre-exposed population. We found a notably lower IgM titer induction in pre-exposed Tanzanian participants compared to the malaria naïve participants vaccinated with the same SUM-101. This differential IgM induction likely reflects the underlying immunological landscape shaped by prior malaria exposure. In endemic populations, repeated natural infections may prime the immune system toward recall responses, with SUM-101 activating the pre-existing memory B cells that have undergone class switching favoring rapid IgG production over *de novo* IgM responses. In contrast, malaria-naïve individuals mount a more conventional primary immune response that includes robust IgM and IgG induction. The phenomenon of recall responses being preferred over *de novo* responses against an antigenic variant upon re-encounter has been recently shown to potentially last lifelong.^12^^,^^13^ In support of the idea of preferred boosting of pre-existing, IgG class-switched immunity after SUM-101 inoculation is our observation that peak vaccine-induced IgG responses are reached after the second vaccination in volunteers starting with higher baseline antibody levels. From a vaccine development perspective, these findings suggest that SUM-101 is capable of effectively boosting pre-existing MSP1-specific immunity in endemic populations, reinforcing the importance of tailoring immunization strategies to prior exposure status.

Human populations residing in malaria-endemic regions naturally acquire immunity after repeated Pf infections targeting the asexual blood stage and antibodies play a key role in eliminating or controlling asexual blood stage parasites.^14^, ^15^, ^16^, ^17^, ^18^ This observation led to the identification and development of a number of asexual blood stage vaccine candidates that are currently pursued in clinical development. Apart from the most advanced asexual blood stage vaccine candidate, RH5, more candidates have progressed to clinical evaluation with promising results, including the pregnancy-associated malaria antigen VAR2CSA, also formulated with GLA-SE, and the N-terminal domain of the Pf serine repeat antigen 5 (BK-SE36) formulated with aluminum hydroxyl gel.^19^

Antibodies targeting asexual blood stage parasites can exert a range of Fc-mediated effector functions, including recruitment of complement factors,^20^^,^^21^ NK cell activation,^22^ merozoite opsonization by monocytes^23^^,^^24^ and neutrophils^25^ and respiratory burst and release of ROS by neutrophils.^26^ While so far no good correlates of immune protection against natural infections or vaccination are defined, several lines of evidence support the idea that Fc-receptor dependent, functional antibodies targeting PfMSP1 might contribute to protection against asexual blood-stage parasites.^6^^,^^27^ Nukumama et al., demonstrated a positive correlation between the breadth of these Fc-receptor-dependent antibody functions and level of control of asexual blood stage parasite growth *in vivo*.^17^ Here, eight different Fc-receptor-mediated antibody effector functions were assessed. While the breadth of functional effector functions at peak response varied between volunteers, these responses have the potential to complement the limited protection offered by approved pre-erythrocytic malaria vaccines by targeting break-through parasites released from the liver.

Efficacious malaria vaccines need to elicit malaria strain-transcending immunity. Large population-level studies of prevalence and distribution of MSP1 genotype frequencies have consistently revealed tremendous genetic diversity in this protein, focused particularly on domains 2, 4, 6, 8, 10, 14 and 16, and that genotype frequencies oscillate considerably over time. For example, a study in a single Malian town, based only on MSP1-19, which is encoded by the relatively conserved domain 17 of the MSP1 protein, revealed that the prevalence of the Pf3D7 variant was ∼16%.^28^ When combined with the very high recombination rate in Pf, and the tremendous variation in domains other than 17, this observation suggests that the frequency of Pf3D7-encoded MSP1FL is essentially negligible globally. In fact, in a search of >500 MSP1 variants available in GenBank collected from all continents where malaria is endemic showed no other allele with 100% sequence similarity to the Pf3D7-encoded MSP1FL variant.^6^ It is therefore encouraging that SUM-101 induced antibodies were active against live merozoites of the homologous Pf3D7 and heterologous PfFCB1 strains in Fc-receptor-dependent complement activation and ADRB indicating that conserved regions of MSP1 seem to be a target of functional antibodies.

In malaria, negative antibody feedback has been shown to occur against immunodominant CSP epitopes (NANP repeat) in animal models.^29^ Blocking of antigen-specific B cells by antibody feedback can potentially hamper or skew the generation of potent natural or vaccine-induced antibodies targeting essential blocking sites.^30^ Pre-existing PfMSP1 antibodies could hamper SUM-101 induced boosting of protective antibody responses by masking antigenic regions in the vaccine, hence preventing B cell recognition of those epitopes. Future studies comparing and contrasting the number and location of conserved and strain-specific MSP1 epitopes targeted by IgG and IgM antibodies before and after SUM-101 vaccination in malaria naïve and malaria pre-exposed populations will allow to dissect the proportion of recall versus *de novo* generated antibody specificities.

One limit of our study is the follow up period of 140 days which does not allow to address the durability of the SUM-101 induced antibodies. The question of need for booster vaccinations like recommended for RTS,S or R21 will be addressed at later stages of the vaccine development program.

In summary, the findings of this second in human study of SUM-101 in adults participants justify embarking on next steps in the clinical development program of SUM-101. Currently, the safety, tolerability, and immunogenicity in younger children in Ouagadougou, Burkina Faso, is evaluated in an ongoing Phase Ib study (NCT06618885). To assess potential of SUM-101 to confer vaccine induced protection, a Phase Ib study in adult participants, (NCT07124156), is expected to start in Q3 2025 in Bagamoyo, Tanzania. This RCT study will include a controlled human malaria infection of viable intra-erythrocytic parasites of the 3D7 Pf strain.

## Contributors

CD, AO, MM, RTL, ML, AA and EB conceptualized the study, acquired funding, designed methodology, were involved with project administration and study supervision. AO served as clinical site principal investigator in Tanzania. AEK, AO, MS, OH, NU, IS, SM, SG and MM contributed to the clinical assessments in the study and sample collection, administration and storage. FV, RTL, KF, VK, MM, and CD contributed to data generation, analysis and interpretation of the secondary study endpoints. FV and RTL performed the statistical analysis of primary and secondary study endpoints. FV, DP, AO, SG, MS and CD have accessed and verified all the underlying data collected and presented. All authors contributed to the writing and provided approval for the final submitted version to be published and take responsibility for the decision to submit for publication. CD had overall accountability for the study.

## Data sharing statement

Any request for study data, including de-identified participant data, data dictionaries and study documents (protocol, statistical analysis plan, informed consent), will be reviewed by the study sponsors (industrial sponsor: Sumaya Biotech and academic sponsor: SwissTPH) and granted based on scientific merit of the proposal, available funding and signed data use agreement. Data access can be requested through corresponding author: Claudia.Daubenberger@swisstph.ch.

## Declaration of interests

FV, DP, SG, MS, and CD declared that part of their contribution to this work was funded through Sumaya Biotech GmbH & Co. KG, Heidelberg. EB and ML are shareholders of Sumaya GmbH & Co. KG. EB and AA were employees of Sumaya GmbH & Co. KG. RTL is currently an employee of Sumaya GmbH & Co. KG. No declarations: AEK, MM, OH, NU, IS, SM, KF, VK, and AO.

## Appendix A Supplementary data

Supplementary Methods

Protocol
