# Supplementary material for: Full-length merozoite surface protein 1 formulated with GLA-SE adjuvant in malaria pre-exposed adults: a randomised, controlled, double-blind, parallel-group, single-centre Phase Ib trial
Source: eClinicalMedicine. 2025 Oct 25;89:103585. doi: 10.1016/j.eclinm.2025.103585 (PMC12595093; doi:10.1016/j.eclinm.2025.103585)
Supplement: Supplementary Material [file mmc1.docx]

**Full-length merozoite surface protein 1 formulated with GLA-SE adjuvant is safe and immunogenic in malaria pre-exposed adults and boosts strain-transcending, Fc-receptor dependent functional antibodies targeting merozoites: a randomised, controlled, double-blind, parallel-group, single-centre Phase Ib trial**

Aina-ekisha Kahatano1*, Maxmillian Mpina1*, Fiona Vanobberghen2,3, Omary Hassan1, Nsiande Urasa1, Ibrahim Sasamalo1, Sarah Mswata1, Daniel Paris2,3, Suzanne Gajewski2,3, Meera Saxena2,3, Kristin Fürle4, Viktoria Kiehl4, Ernst Böhnlein4, Andrea Aschenbrenner4, Michael Lanzer5, Richard Thomson-Luque4,5, Ally Olotu1**†**, Claudia Daubenberger2,3**†**

**SUPPLEMENT**

**Supplementary Table 1. Details of local and systemic solicited AEs at least possibly related to SUM-101 or Verorab after vaccination and up to 7 days later.**

|  |  | **Verorab group**  **N=20** | **SUM-101 group**  **N=20** |
| --- | --- | --- | --- |
| Total | - | 26 (13, 50%) | 26 (10, 38%) |
| Last vaccination number | 1 | 9 (7, 27%) | 12 (8, 31%) |
|  | 2 | 7 (6, 23%) | 7 (5, 19%) |
|  | 3 | 10 (6, 23%) | 7 (4, 15%) |
| Duration (days), median (IQR) [range] |  | 1 (1, 2) [0, 4] | 1 (1, 2) [0, 3] |
| Severity | Mild (grade 1) | 23 (13, 50%) | 22 (10, 38%) |
|  | Moderate (grade 2) | 3 (2, 8%) | 4 (2, 8%) |
| Pattern | Intermittent | 13 | 7 |
|  | Continuous | 13 | 19 |
| Relation to IMP | Definitely related | 19 (12, 46%) | 22 (10, 38%) |
|  | Probably related | 6 (3, 12%) | 2 (2, 8%) |
|  | Possibly related | 1 (1, 4%) | 2 (1, 4%) |
| Action taken with IMP | None (participant will continue as per protocol) | 25 | 26 |
|  | Not applicable (participant already completed all vaccinations) | 1 | 0 |
| Outcome | Resolved / Recovered | 26 | 26 |

Results are number of events (and number, % of participants with at least one AE where indicated), except as indicated for duration.

**Supplementary Table 2. Details of other Adverse Events**

|  |  | **Verorab group**  **N=20** | **SUM-101 group**  **N=20** |
| --- | --- | --- | --- |
| Total | - | 11 (8, 73%) | 12 (7, 58%) |
| Duration (days), median (IQR) [range] |  | 7 (3, 9) [1, 11] | 6 (4, 8) [3, 14] |
| Severity | Mild (grade 1) | 2 (2, 18%) | 6 (5, 42%) |
|  | Moderate (grade 2) | 9 (7, 64%) | 6 (4, 33%) |
| Pattern | Intermittent | 1 | 2 |
|  | Continuous | 10 | 10 |
| Relation to IMP | Unlikely related | 6 (6, 55%) | 6 (5, 42%) |
|  | Not related | 5 (3, 27%) | 6 (4, 33%) |
| Action taken with IMP | None (participant will continue as per protocol) | 8 | 12 |
|  | Not applicable (participant already completed all vaccinations) | 3 | 0 |
| Outcome | Resolved / Recovered | 11 | 12 |

Results are number of events (and number, % of participants with at least one AE where indicated), except as indicated for duration.

**Supplementary Table 3. Other adverse events, by system organ class, preferred term, and severity.**

| **MedDRA primary system organ class** | **MedDRA primary preferred term** | **Severity** | **Verorab group**  **N=20** | **SUM-101 group**  **N=20** |
| --- | --- | --- | --- | --- |
| Gastrointestinal disorders | Gastritis | Mild (grade 1) | 0 | 1 (1, 5%) |
| Infections and infestations | Gastroenteritis | Moderate (grade 2) | 0 | 1 (1, 5%) |
| Infections and infestations | Impetigo | Moderate (grade 2) | 0 | 1 (1, 5%) |
| Infections and infestations | Malaria | Mild (grade 1) | 0 | 1 (1, 5%) |
| Infections and infestations | Sepsis | Moderate (grade 2) | 1 (1, 5%) | 0 |
| Infections and infestations | Tonsillitis | Moderate (grade 2) | 1 (1, 5%) | 1 (1, 5%) |
| Infections and infestations | Upper respiratory tract infection | Moderate (grade 2) | 3 (3, 15%) | 2 (2, 10%) |
| Infections and infestations | Urinary tract infection | Mild (grade 1) | 1 (1, 5%) | 1 (1, 5%) |
| Infections and infestations | Urinary tract infection | Moderate (grade 2) | 2 (2, 10%) | 0 |
| Infections and infestations | Vulvovaginal candidiasis | Moderate (grade 2) | 1 (1, 5%) | 0 |
| Injury, poisoning and procedural complications | Muscle strain | Mild (grade 1) | 0 | 1 (1, 5%) |
| Nervous system disorders | Migraine | Moderate (grade 2) | 1 (1, 5%) | 0 |
| Respiratory, thoracic and mediastinal disorders | Rhinitis allergic | Mild (grade 1) | 0 | 1 (1, 5%) |
| Skin and subcutaneous tissue disorders | Dermatitis allergic | Mild (grade 1) | 1 (1, 5%) | 1 (1, 5%) |
| Skin and subcutaneous tissue disorders | Dermatitis allergic | Moderate (grade 2) | 0 | 1 (1, 5%) |

Results are number of events (and number, % of participants with at least one AE).

**Supplementary Figure 1: Flow chart of clinical and laboratory based study procedures.**

**
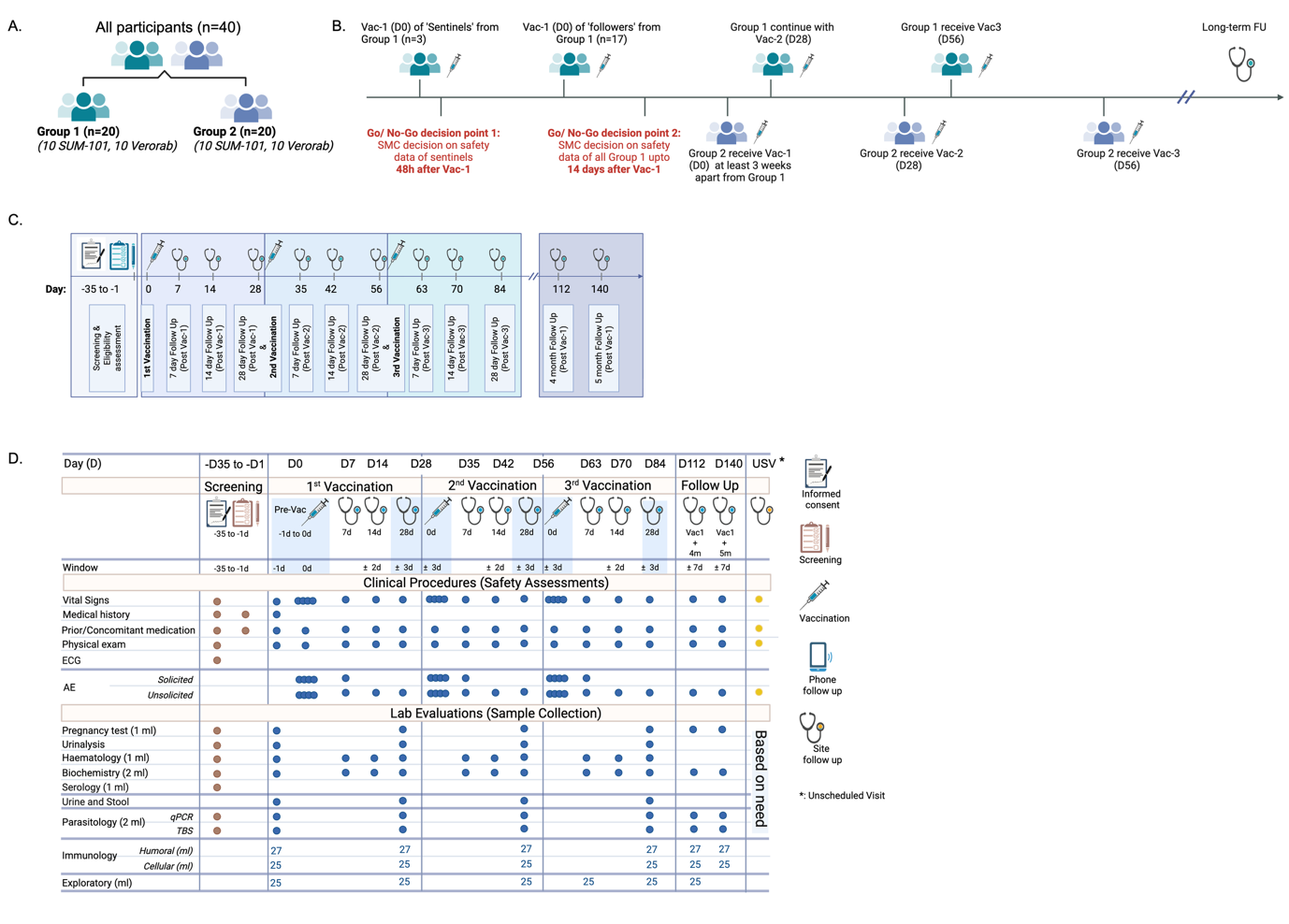
**

Panel A shows allocation of study participants to the two different arms. Panel B demonstrates the protocol defined follow up visits, including the SMC meetings after the frist group vaccination and the progression to group 2. Panel C shows the vaccinations and the follow up visits taking place after each vaccination and during follow up period. Panel D summarizes the time points, repeats of clinical and laboratory based procedures and sample volumes collected throughout the study.

**Supplementary Figure 2. Changes in ALT levels over time versus baseline.**


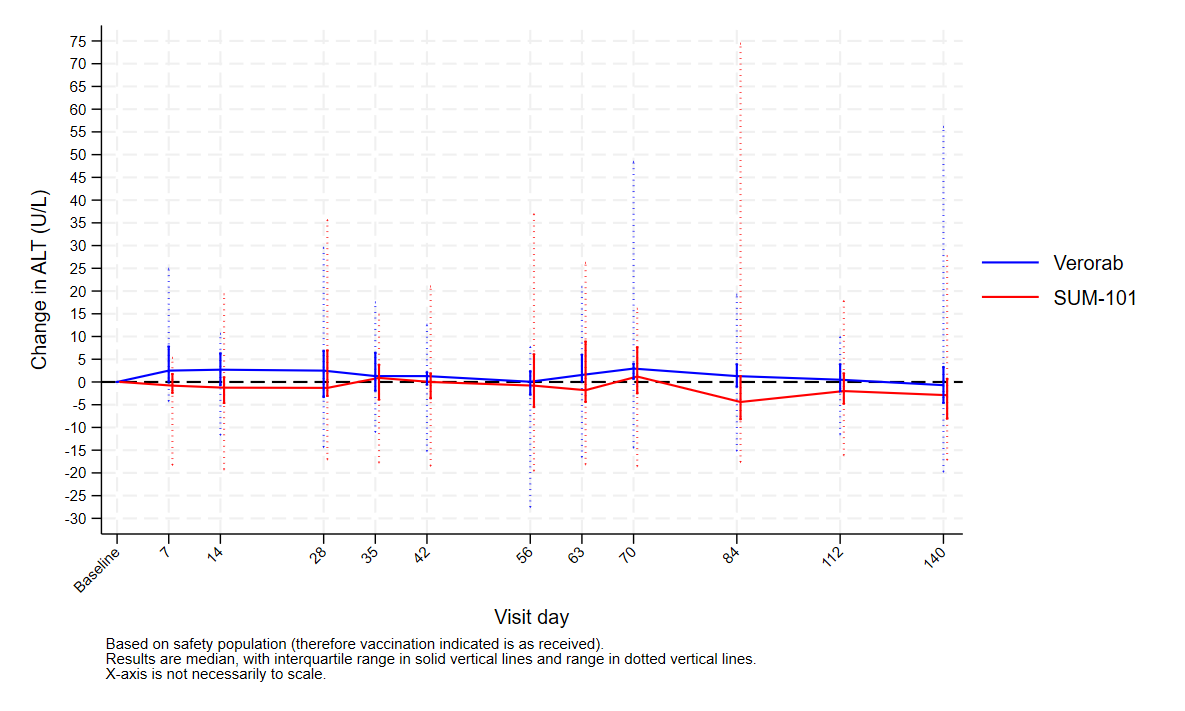


**Supplementary Figure 3. Change in bilirubin levels over time versus baseline.**


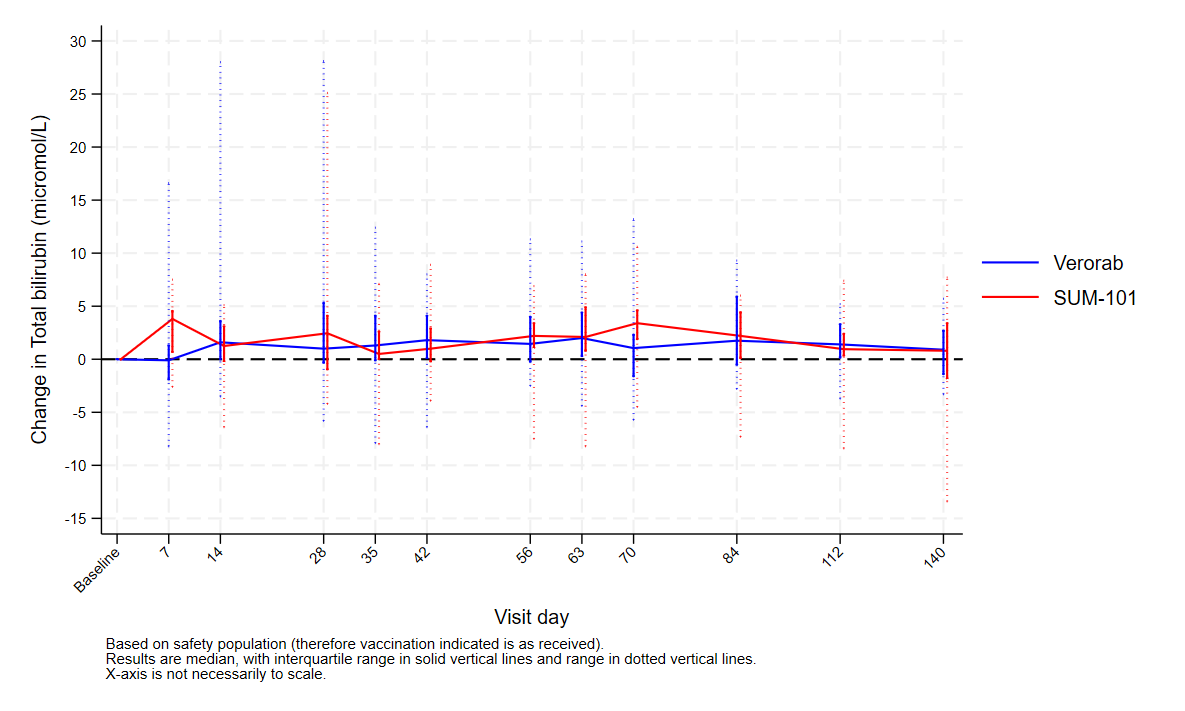


**Supplementary Figure 4. Change in creatinine levels over time versus baseline.**


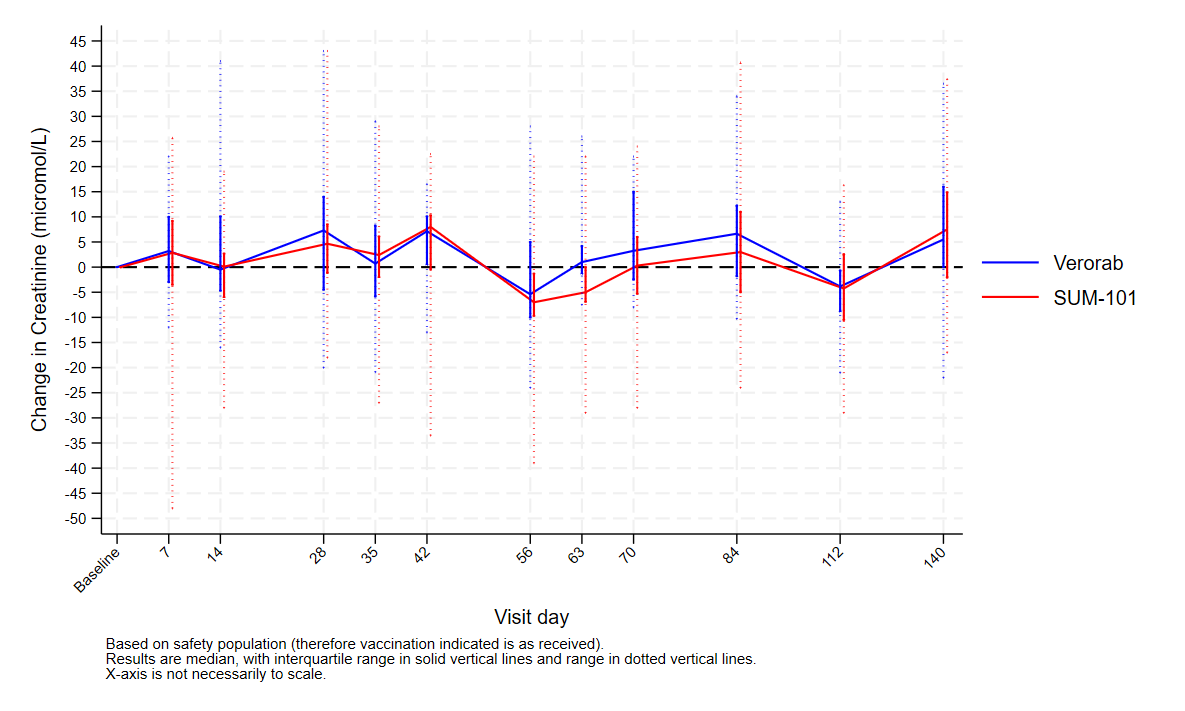


**Supplementary Figure 5. Change in ALT levels between just prior and up to 28 days after each vaccination.**


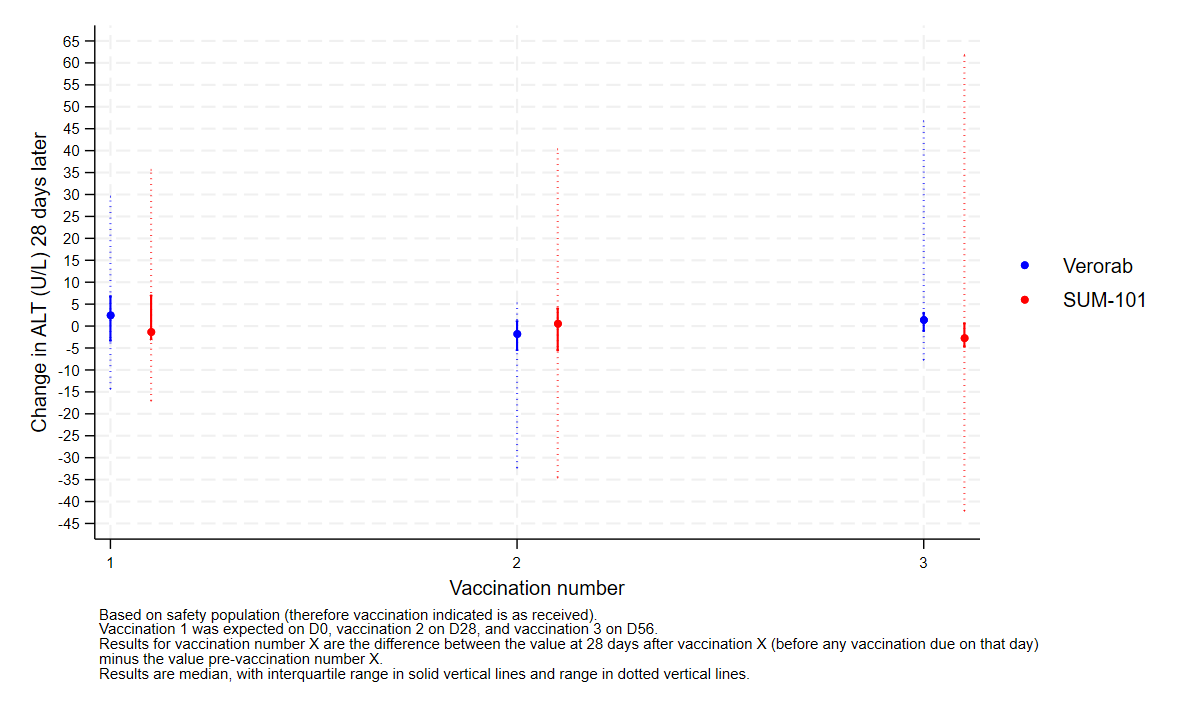


### **Supplementary Figure 6. Change in bilirubin levels between just prior and up to 28 days after each vaccination.**


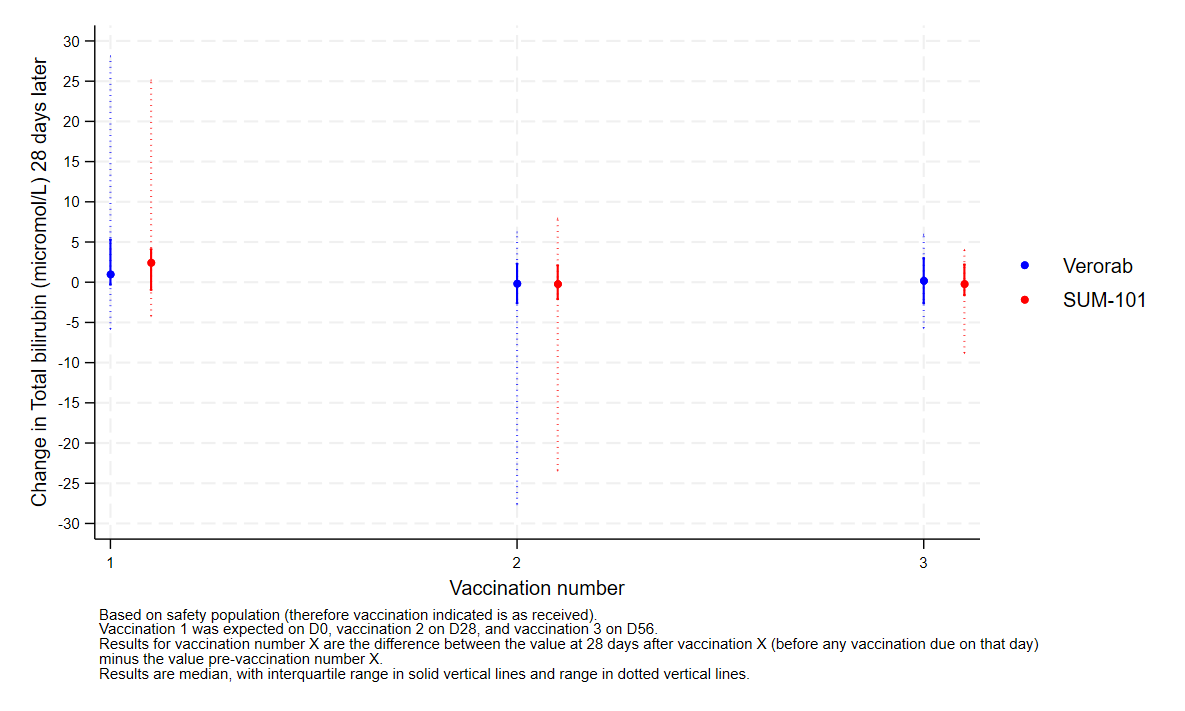


### **Supplementary Figure 7. Change in creatinine levels between just prior and up to 28 days after each vaccination.**


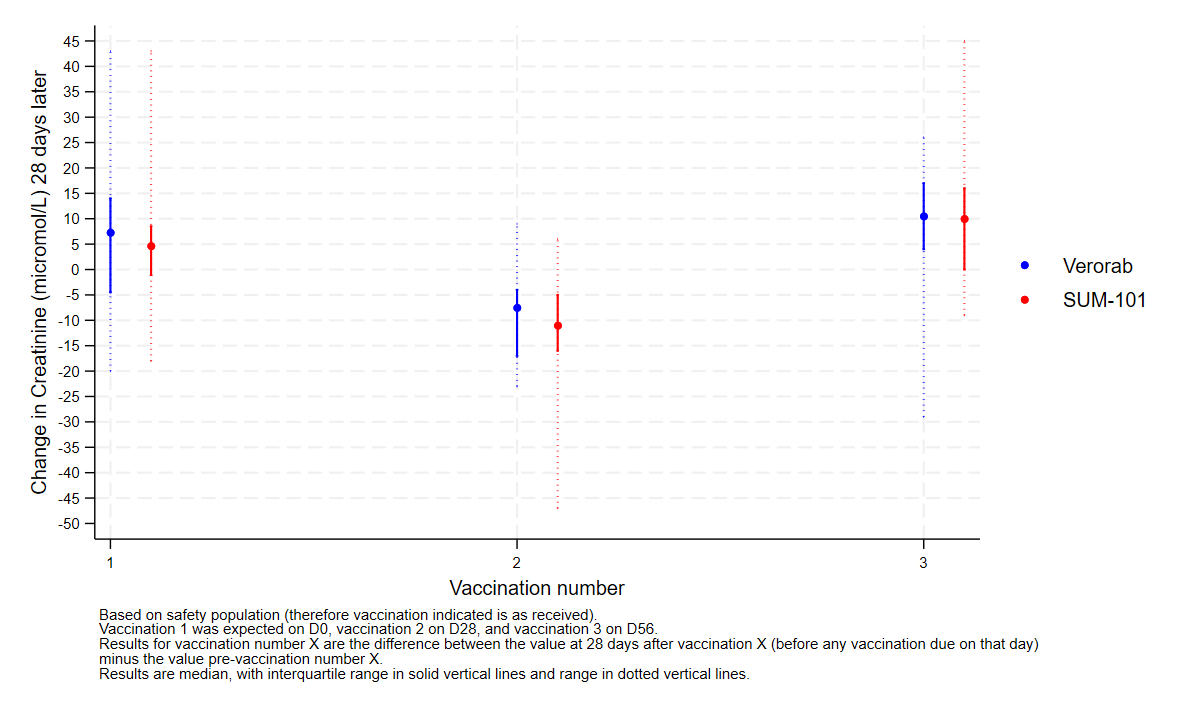


### **Supplementary Figure 8. Change in haemoglobin levels over time versus baseline.**


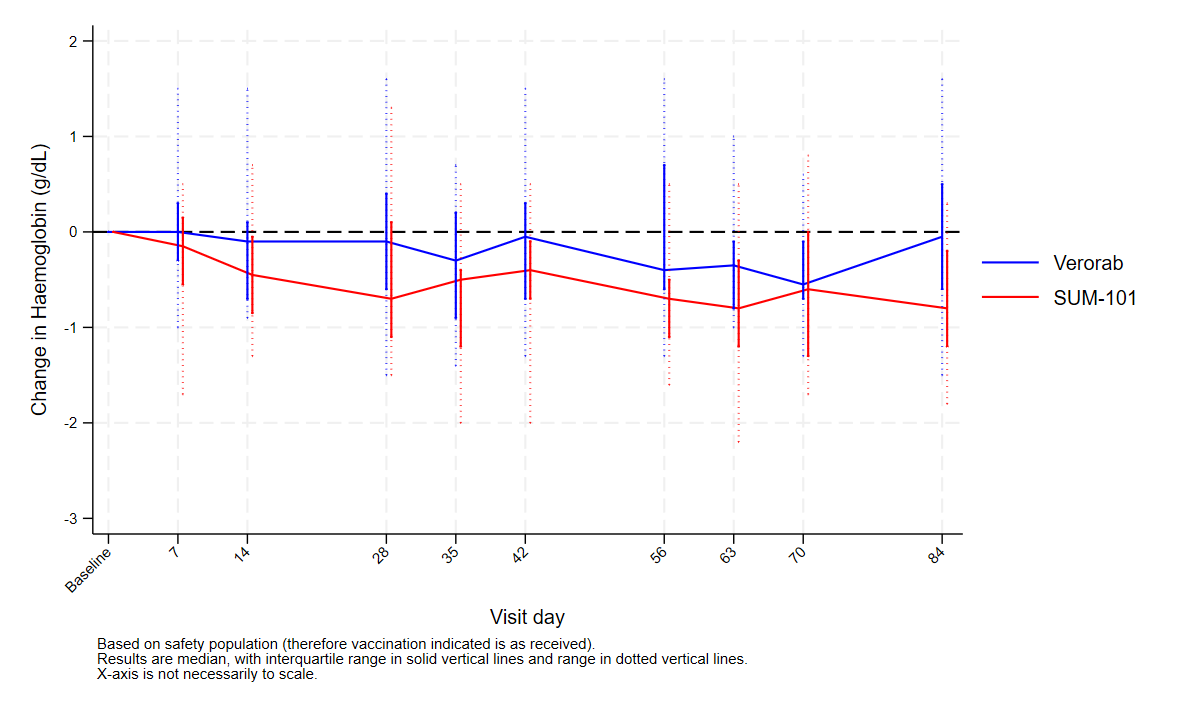


### **Supplementary Figure 9. Change in leucocytes levels over time versus baseline.**

**
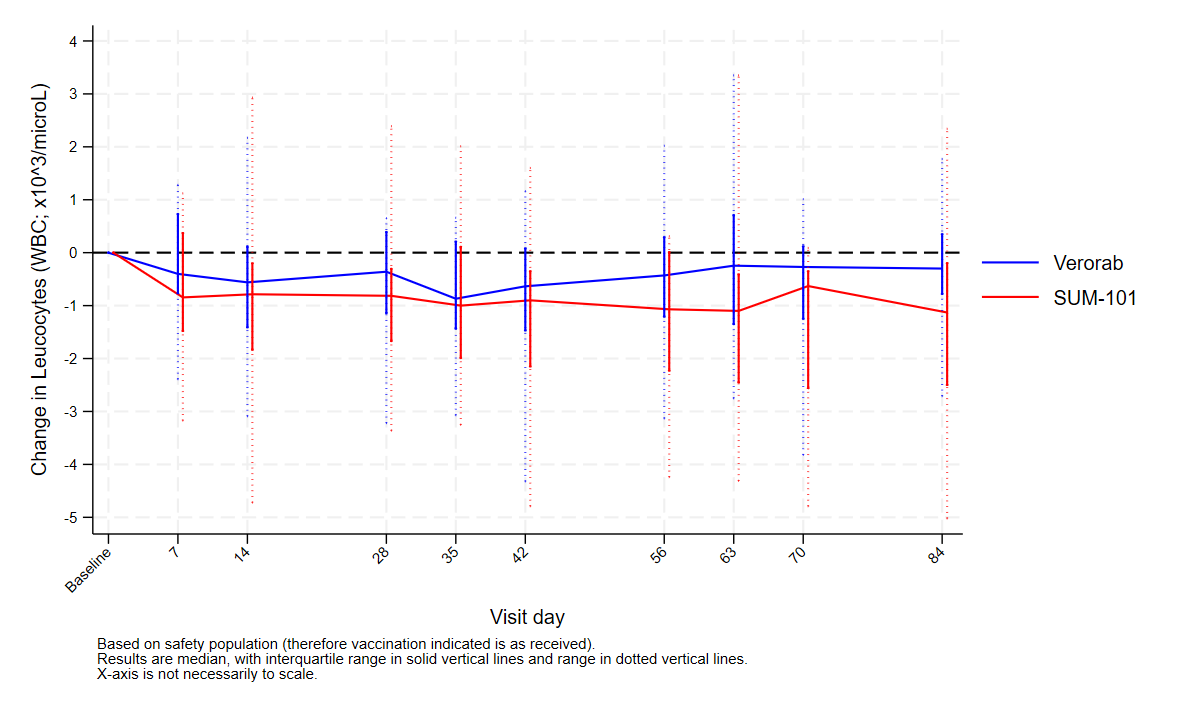
**

### **Supplementary Figure 10. Change in neutrophils levels over time versus baseline.**

**
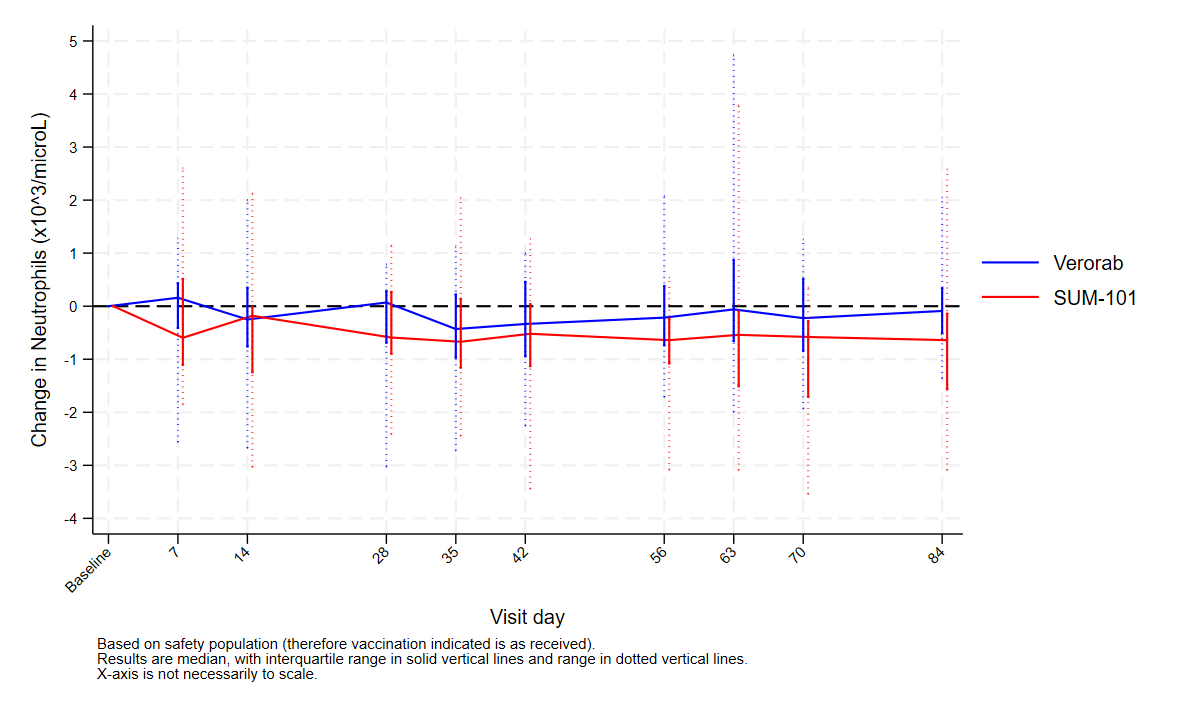
**

### **Supplementary Figure 11. Change in eosinophils levels over time versus baseline.**

**
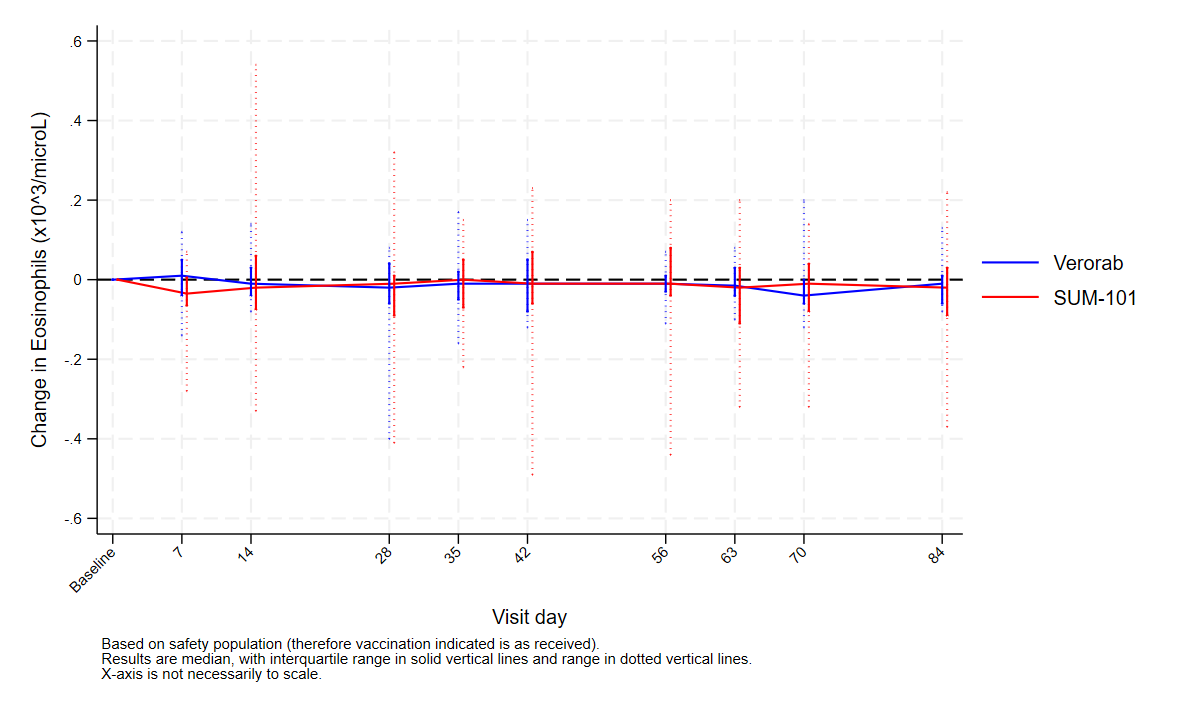
**

### **Supplementary Figure 12. Change in platelets levels over time versus baseline.**

**
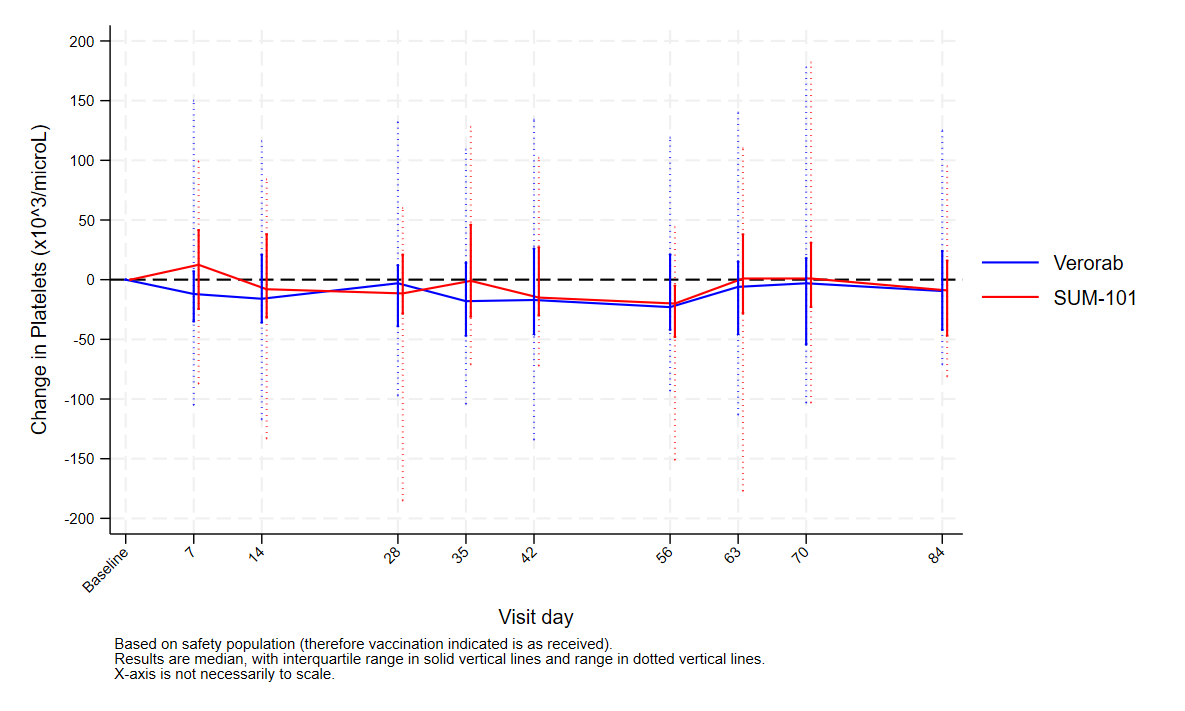
**

### **Supplementary Figure 13. Change in haematocrit levels over time versus baseline.**

**
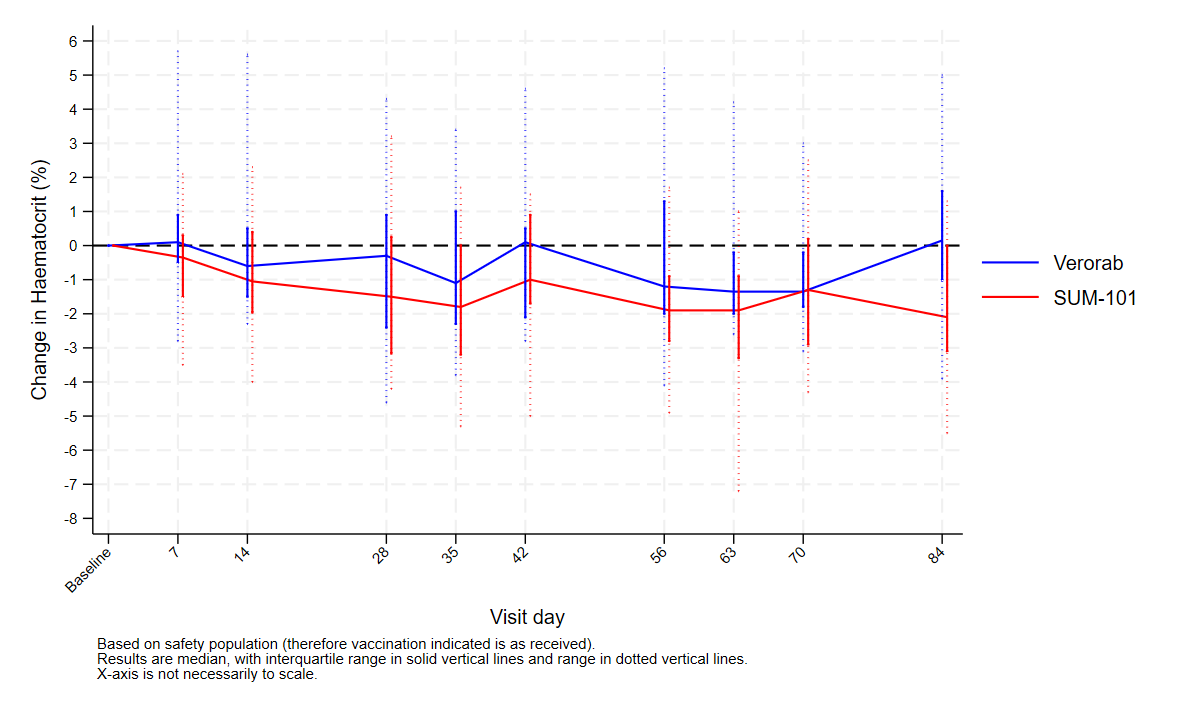
**

### **Supplementary Figure 14. Change in haemoglobin levels between just prior and up to 28 days after each vaccination.**

**
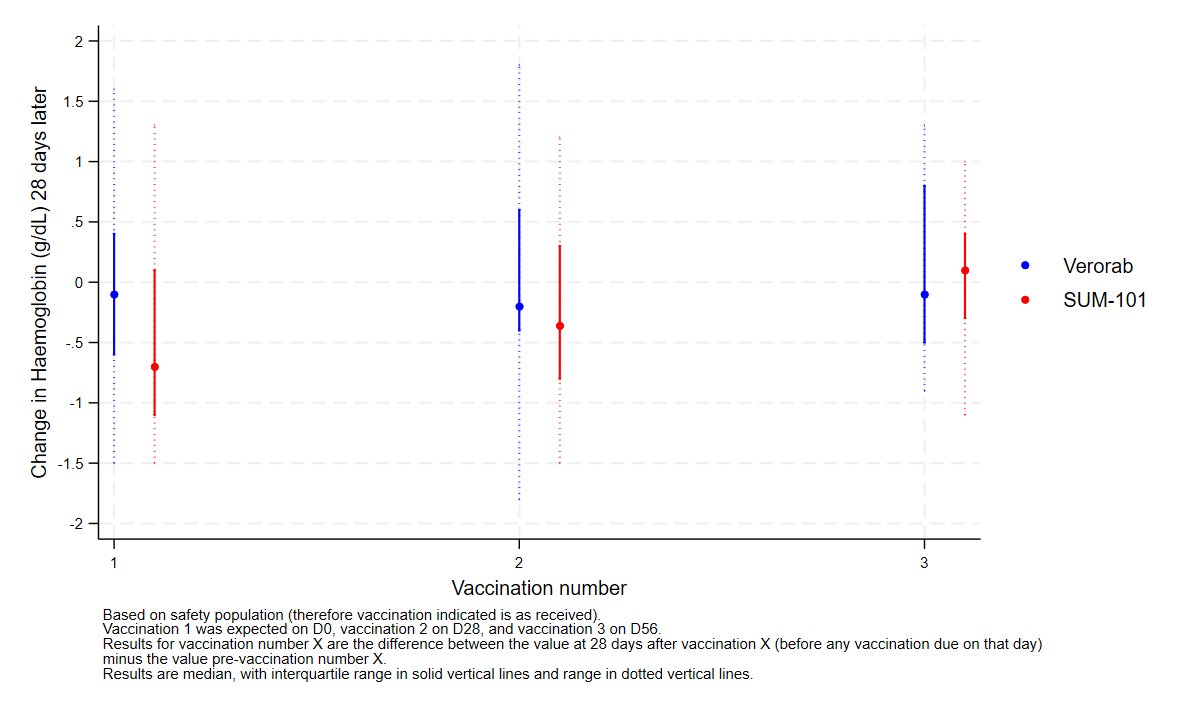
**

**Supplementary Figure 15. Absolute haemoglobin values over time until one month after third vaccination.**


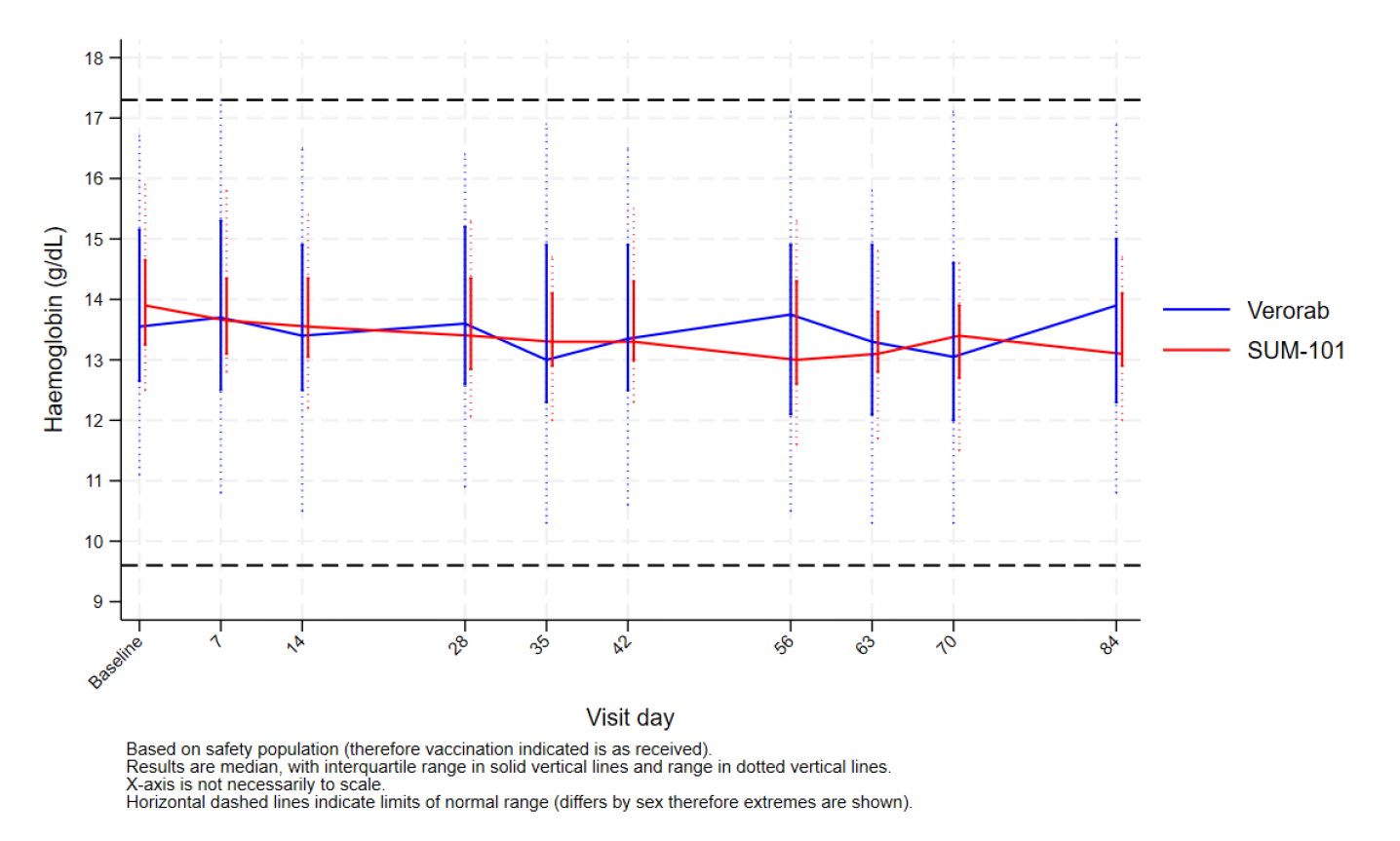


### **Supplementary Figure 16. Change in leucocytes levels between just prior and up to 28 days after each vaccination.**

**
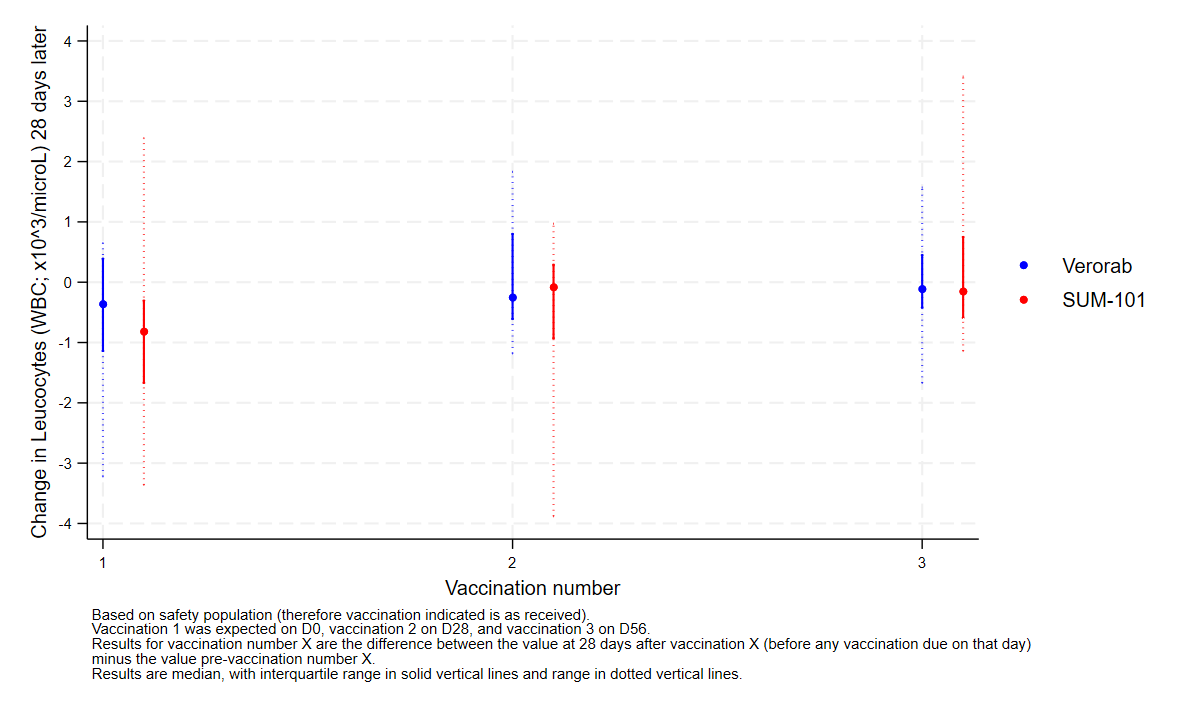
**

### **Supplementary Figure 17. Change in neutrophils levels between just prior and up to 28 days after each vaccination.**

**
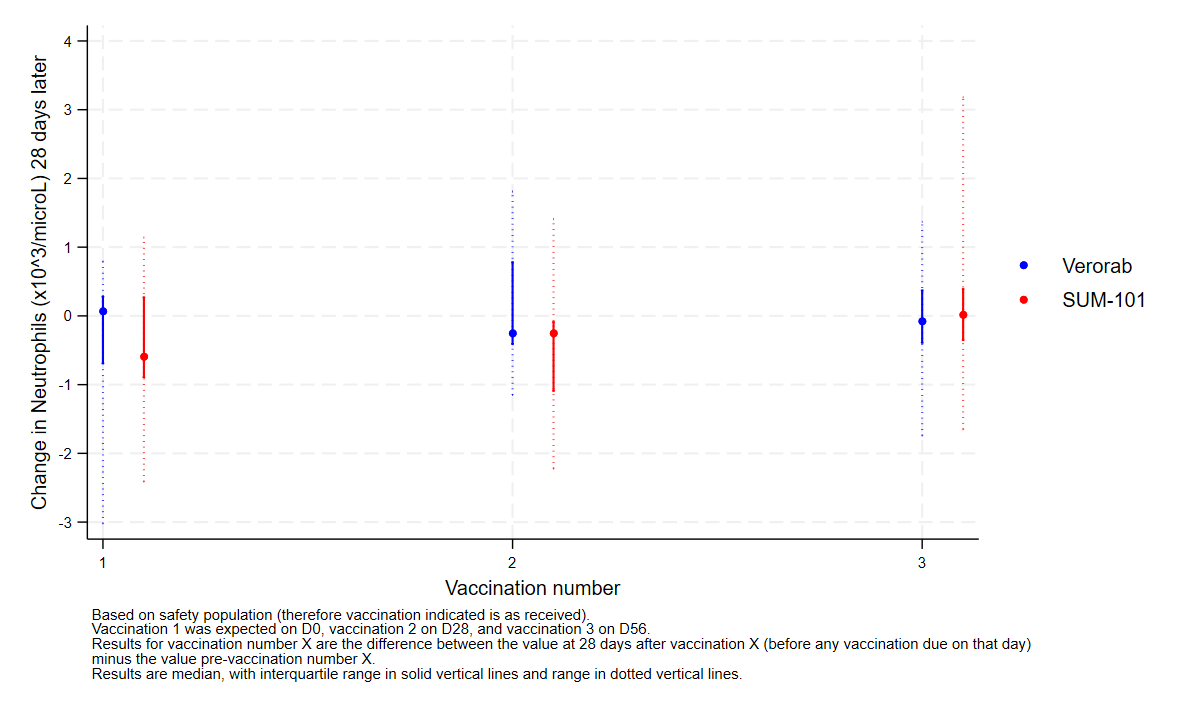
**

### **Supplementary Figure 18. Change in eosinophils levels between just prior and up to 28 days after each vaccination.**

**
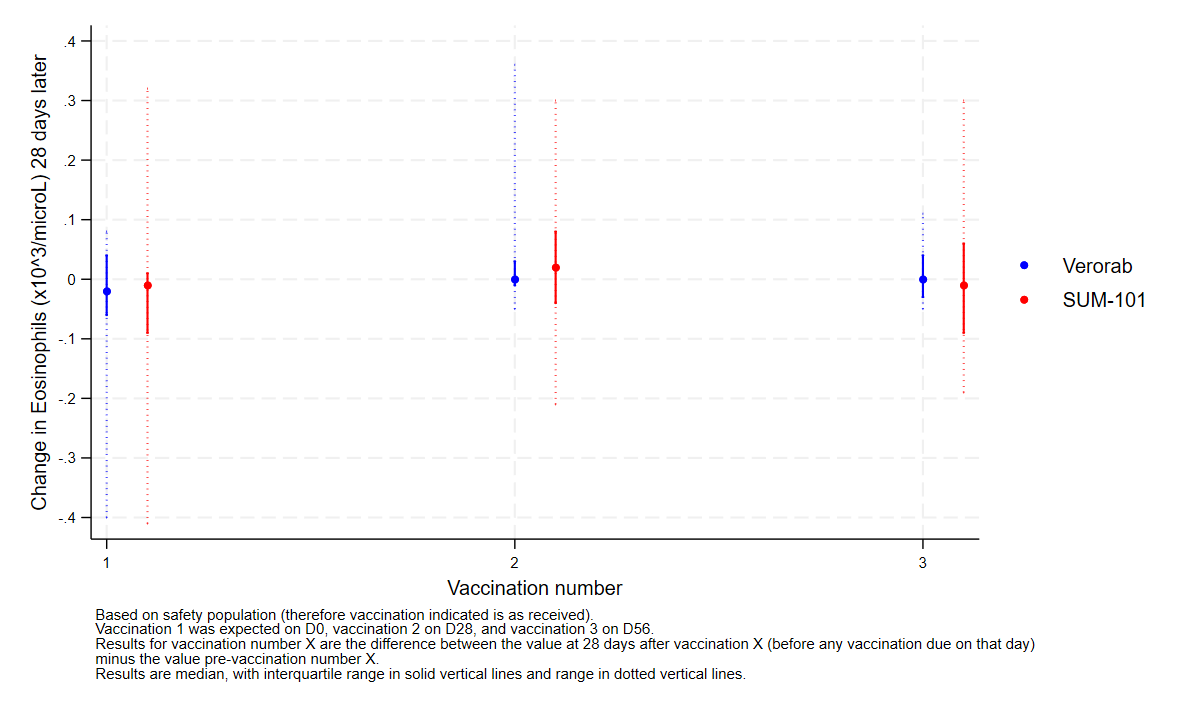
**

### **Supplementary Figure 19. Change in platelets levels between just prior and up to 28 days after each vaccination.**

**
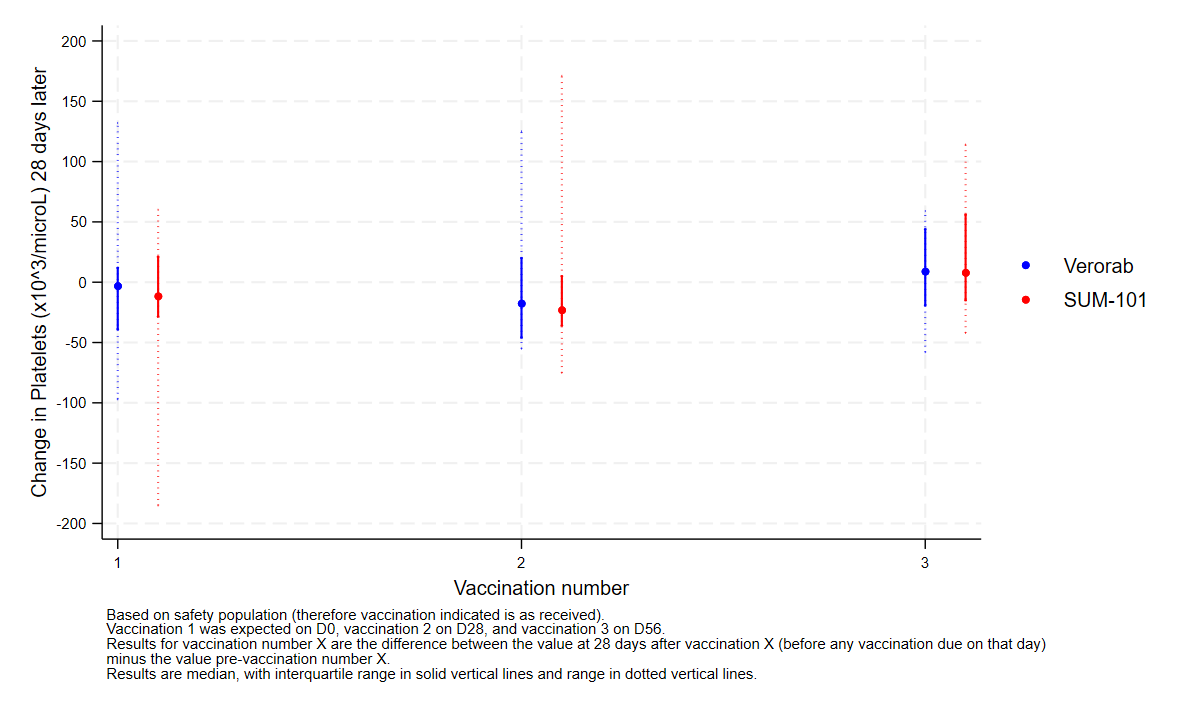
**

### **Supplementary Figure 20. Change in haematocrit levels between just prior and up to 28 days after each vaccination.**

**
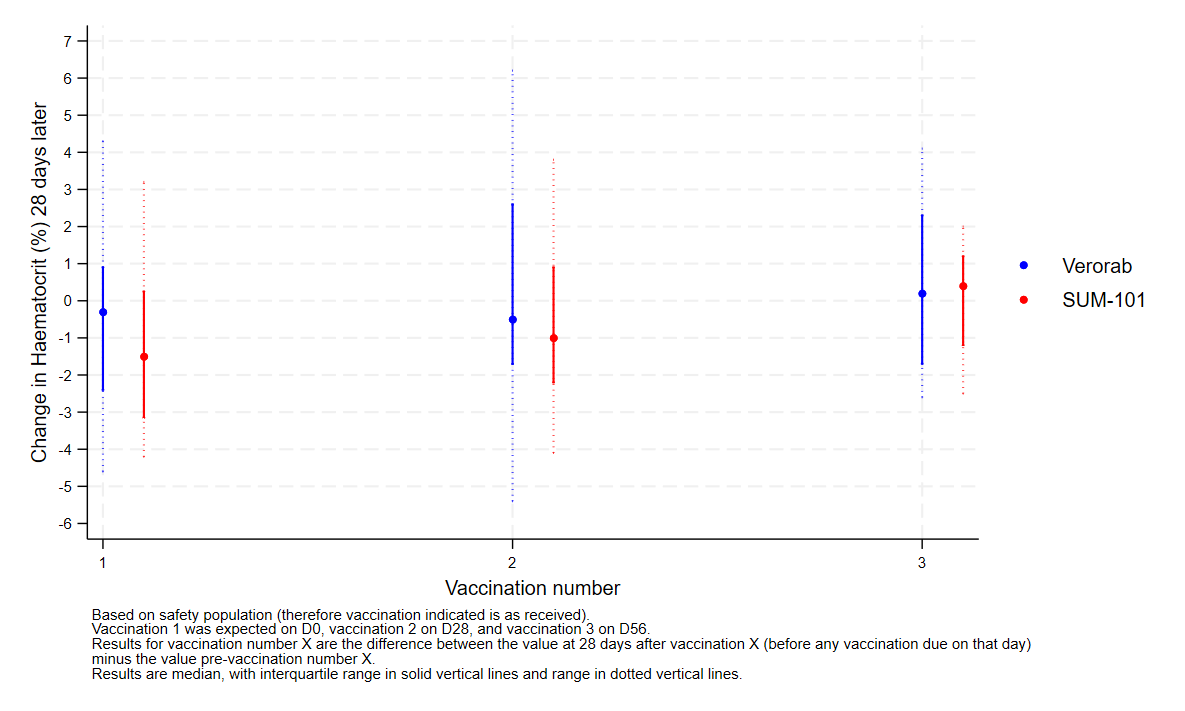
**

### **Supplementary Figure 21. Distribution of glucose results over time.**

**
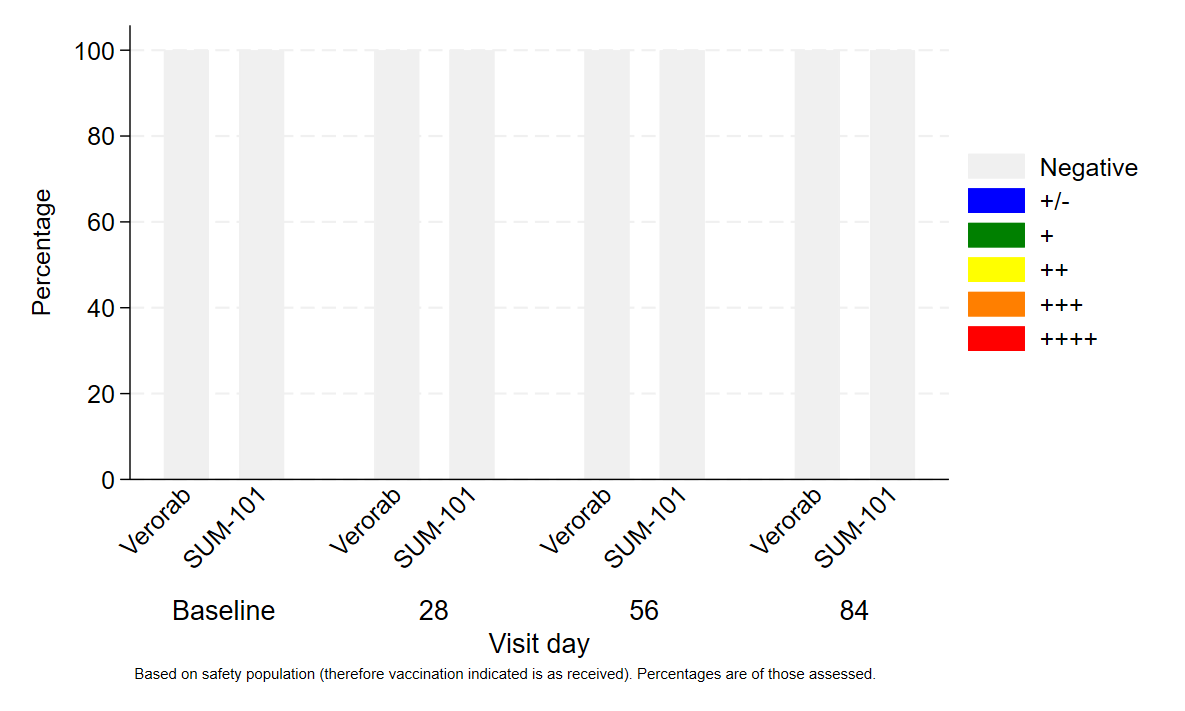
**

### **Supplementary Figure 22. Distribution of protein results over time.**

**
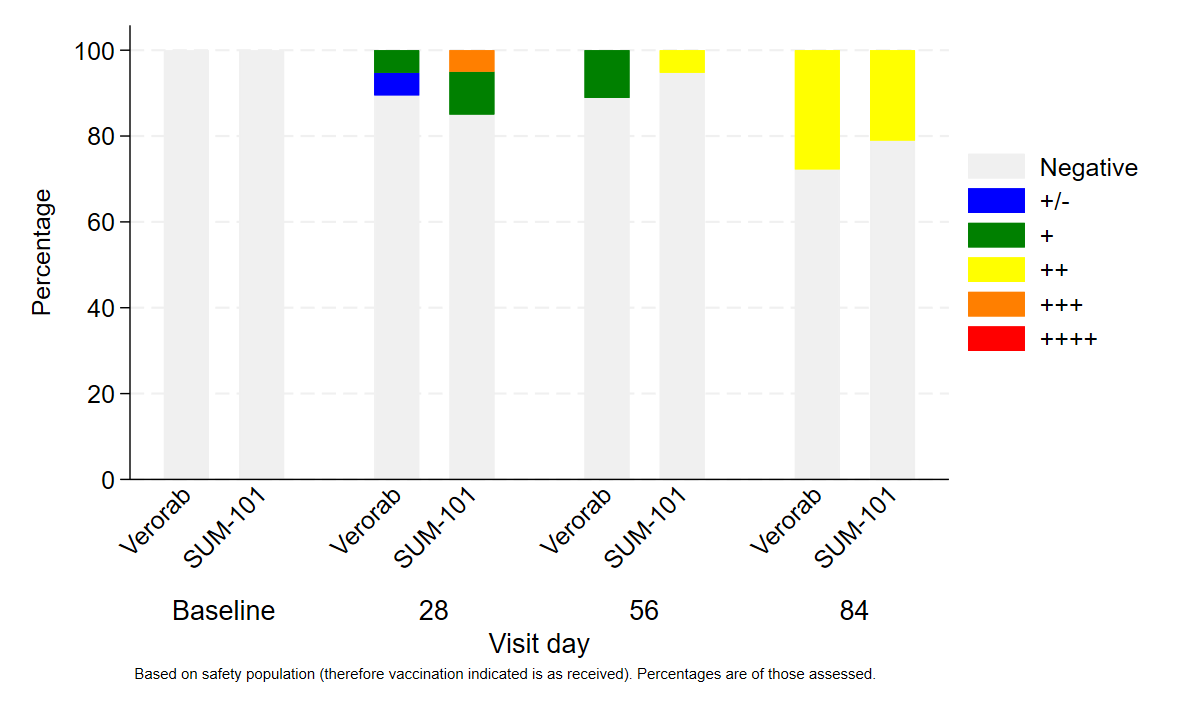
**

### **Supplementary Figure 23. Distribution of blood results over time.**


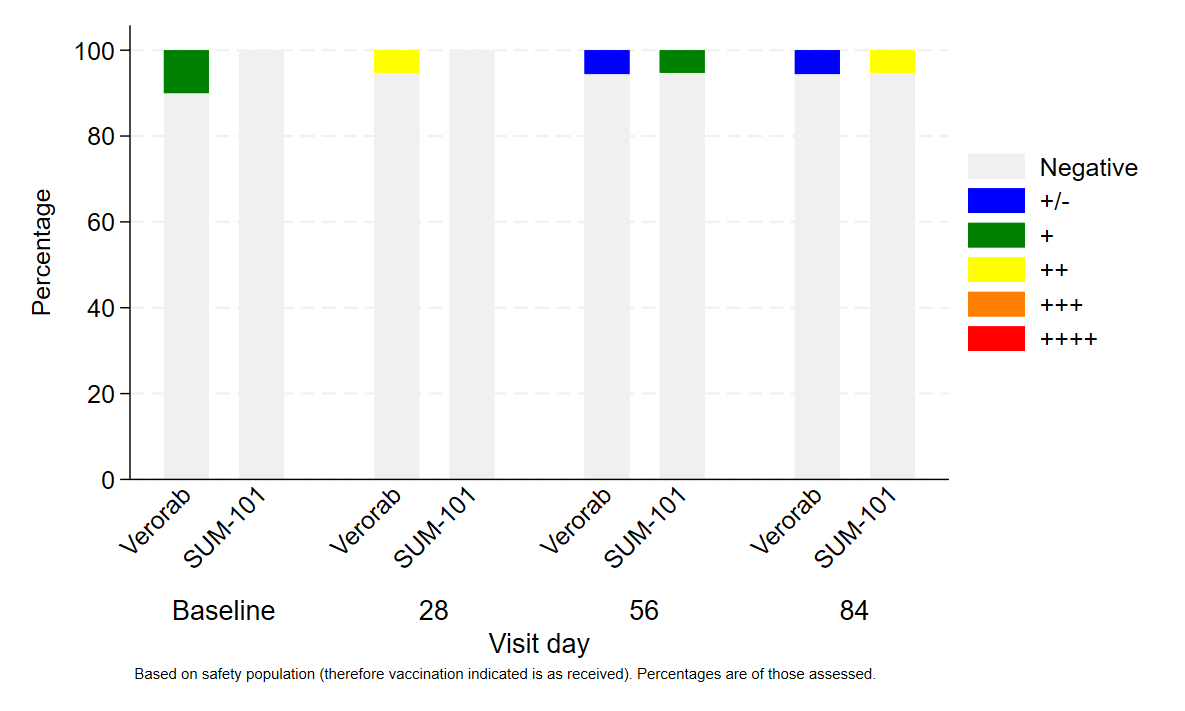


**Supplementary Figure 24. Fold changes in IgM and IgG antibody responses in Phase Ia and Phase Ib study participants.**

Results are mean and 95% confidence interval. Dots represent individual participants. Y axes have different scales to best illustrate the data. Panels B and D are the same data as presented in panels A and C, respectively, but omitting the Phase Ia study results in order to clearly visualise the results from the Phase Ib study. Where applicable, numbers under the graphs indicate the numbers of participants contributing to each datapoint.

**Supplementary Figure 25. IgM titres among participants in the SUM-101 group, categorised by whether baseline titre above or below the median.**

Results are geometric mean and 95% CI.

**Supplementary Figure 26. IgG titres among participants in the SUM-101 group, categorised by whether baseline titre above or below the median.**

Results are geometric mean and 95% CI.

**Supplementary Figure 27. Correlation of IgG reactivity against the prototypic sequences MSP1-D (from the MAD20 strain) and MSP1-F (from the WELLCOME strain).**


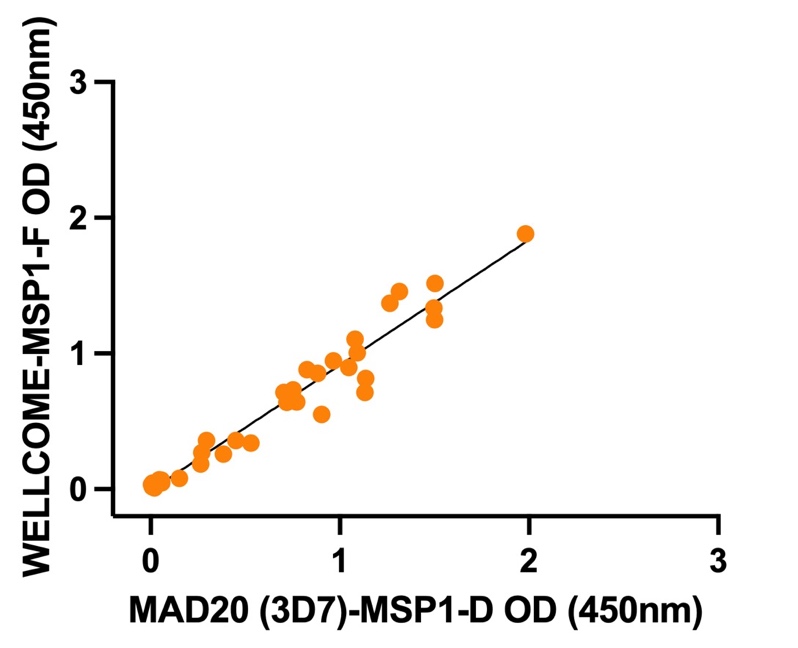


Results are ELISA values measured at OD 450nm

**Supplementary Figure 28. The full-length MSP1heterodimer construct is generated from two recombinant proteins expressed from two plasmids expressing the fragments p83/30 and p38/42.**
