# Supplementary material for: Full-length merozoite surface protein 1 formulated with GLA-SE adjuvant in malaria pre-exposed adults: a randomised, controlled, double-blind, parallel-group, single-centre Phase Ib trial
Source: eClinicalMedicine. 2025 Oct 25;89:103585. doi: 10.1016/j.eclinm.2025.103585 (PMC12595093; doi:10.1016/j.eclinm.2025.103585)
Supplement: Supplementary Methods [file mmc2.pdf]

SUM-101 PHASE IB TANZANIA: IMMUNOLOGY METHODS

Enzyme-linked immunosorbent assay (ELISA) for antibody titers

Total IgG and IgM antibody levels were assessed by ELISA using MaxiSorp plates (Thermo Fisher Scientific, #442404) coated overnight at 2-8°C with 100 µl/well of 100 nM recombinant $MSP1_{FL}$ (Glycotope Biotechnology GmbH, Heidelberg). The second day, plates were washed with 200 µl/well of TBST wash buffer (150 mM NaCl, 10 mM Tris (Roth #4855.2) and 0.05 % v/v Tween 20 (Merck, #9005-64-5), pH 8.0) using an AquaMax 4000 microplate washer (Molecular Devices). Blocking at RT for 1 hour was then conducted with TBST + 1% w/v milk powder (Roth, #68514-61-4). Serum samples from volunteers at all timepoints were initially diluted (starting dilutions between 1:100 to 1:2,000 for IgG and 1:20 to 1:200 for IgM) and then serially diluted two-fold and incubated for 2 hours followed by a wash step. For IgG titers, wells were incubated with goat anti-human IgG alkaline phosphatase-conjugated secondary antibodies (Sigma-Aldrich, #A1543) at a 1:20,000 dilution for 1 hour. After washing, p-nitrophenyl phosphate (Sigma-Aldrich #20-106) was added as a substrate and incubated for 1 hour at RT in the dark, followed by termination with 0.2 M NaOH (Sigma-Aldrich, #S9888). Absorbance was measured at optical density (OD) 405 nm using a Cytation 3 plate reader (BioTek). For IgM titers, wells were incubated with goat anti-human IgM horseradish peroxidase (HRP)-conjugated secondary antibodies (Sigma-Aldrich, #A3437) at a 1:20,000 dilution for 1 hour. After washing, the substrate SigmaFAST OPD (Sigma-Aldrich, #P9187) was incubated for 20 minutes in the dark before the reaction was stopped by the addition of 1M HCl and measured at OD 492 nm using a Cytation 3 plate reader (BioTek). To quantify IgG1, IgG2, IgG3, and IgG4 subclass titres, the same ELISA protocol was employed, but using subclass-specific peroxidase-conjugated secondary antibodies (Thermo Fisher, #A-10648 for IgG1, #05-0520 for IgG2, #05-3620 for IgG3 and #MH1742 for IgG4). Serum dilution ranged between from 1:5 to 1:400. For detection, 1-step Turbo TMB substrate (Thermo Fisher, #34022) was used, with a 20-minute incubation period at RT in the dark, and the reaction was stopped by the addition of 1M HCl (Honeywell Fluka, # 7647-01-0). ODs were recorded at 450 nm using a Cytation 3 plate reader (BioTek).

Complement-based assays

For the C1q fixation assay, MaxiSorp plates coated with $MSP1_{FL}$ or freshly isolated merozoites (please see section "merozoites isolation") were incubated overnight at 4°C, following the same protocol as the antibody titres´ ELISA assays explained above. The next day, the plates were washed with PBS supplemented with 0.05% Tween 20 using an AquaMax 4000 microplate washer (Molecular Devices) and then blocked with 1% w/v casein (Thermo Fisher,

#37528) in PBS at 37°C for 2 hour. After blocking, 50 µl of 1:25 diluted serum in blocking buffer was added to each well and incubated at 37°C for 1 hour. Following this incubation, the plates were washed and recombinant C1q (Merck Millipore #204876) at 10 µg/ml in blocking buffer was added and incubated for 30 minutes at 37°C. C1q binding was detected using anti-C1q horseradish peroxidase (HRP)-conjugated secondary antibodies (Abcam, #ab46191) at a 1:100 dilution in blocking buffer, incubated for 1 hour at 37°C. After washing, SigmaFAST OPD (Sigma-Aldrich, #P9187) was added for 30 minutes in the dark at room temperature to develop the signal. The reaction was halted with 1M HCl, and the absorbance was measured at OD 492 nm using a Cytation 3 plate reader (Biotek). For the C3b and C5-C9 (MAC) assays, the procedure mirrored those used for the C1q-fixation assay but using the C3b or SC5b-9-specific conjugates, substrate solutions and stop solutions contained in the MicroVue Complement iC3b EIA kit (Quidel, #A006) and MicroVue Complement Sc5b-9 Plus kit (Quidel, #A020). 50 µl of 1:10 diluted serum in blocking buffer was employed. 50 µl of either C3b or SC5b-9 conjugate was added to each well and incubated for 30 minutes at 37°C. For signal development, 100 µl of the specific substrate solution was added for 30 minutes at 37°C and then stopped with the respective stop solution. Absorbance was recorded at OD 405 nm for C3b and OD 450 nm for C5-C9 (MAC) detection using a Cytation 3 plate reader (Biotek).

Culture of THP1 cells

THP1 cells (ATCC, #TIB202) were cultured in RPMI 1640 medium (Gibco by life technologies #52400-041) supplemented with 2mM L-glutamine and 25mM HEPES, 1% penicillin-streptomycin (containing 10,000 units/ml penicillin and 10,000 µg/ml streptomycin) (Invitrogen, #15140122), and 10% fetal bovine serum (FBS) (Invitrogen, #A5256701). THP1 cells were maintained in a humidified incubator at 37°C with 5% CO2. THP1 cell density was checked daily and kept between 1 x 10^5 and 1 x 10^6 cells/ml. THP1 cells were passaged when the density surpassed 1 x 10^6 THP1 cells/ml or on day 6, whichever came first.

Isolation of Neutrophils and Natural Killer (NK) cells from peripheral blood

Blood from healthy malaria-naïve donors was collected in heparin vacutainers and separated using a Ficoll-Histopaque (Sigma-Aldrich, #10771) gradient. The resulting peripheral blood mononuclear cell (PBMC) and neutrophil layers were separated.

Neutrophils were isolated from the erythrocyte pellet by dextran sedimentation and hypotonic lysis of erythrocytes as published elsewhere (Blank et al). Briefly, blood from three donors (18 mL each) was pooled and mixed 1:1 with 3% dextran (Roth, # 9004-54-0) in 0.9% NaCl, then incubated for 18 minutes at RT to sediment erythrocytes. Following centrifugation

at 500 × g for 10 minutes at 4°C, the white cell layer was collected and resuspended in 0.9% NaCl. This suspension was layered on Ficoll-Histopaque and centrifuged at 400 × g for 35 minutes at room temperature. The thin polymorphonuclear layer containing neutrophils was then resuspended in ice-cold distilled water and kept on ice for 30 seconds to lyse any remaining erythrocytes, after which an equal volume of 1.8% NaCl (Sigma Aldrich, #7647-14-5) was added. After another centrifugation at 500 × g for 5 minutes at 4°C, the pellet was washed with Hanks' balanced salt solution (Thermo Fisher Scientific, #88284) and resuspended in cold PBS (Thermo Scientific, # J61196.AP). The quality and concentration of the neutrophils were assessed using a hemacytometer after staining with trypan blue. The final neutrophils concentration was adjusted to 2.5 × 10^7 cells/mL in sterile PBS. Neutrophils were adjusted to a concentration of 3.3 x 10^5 neutrophils/mL and 10 x 10^6 neutrophils/mL for OPA and ADRB assays respectively and then the stored in ice-cold culture media or PBS on ice until used within 3 hours post-isolation.

For NK cell activity assays, isolated PBMCs were washed NK cell medium which contained ice-cold RPMI 1640 supplemented with 2 mM L-glutamine (Gibco Life technologies, #11875093), 10% FBS, and 1% penicillin-streptomycin, and counted using a hemocytometer. NK cells were isolated from PBMCs using the the NK Cell Isolation Kit (Miltenyi Biotec, # 30-092-657),following the manufacturer's instructions. Briefly, 20 µl of antibody mix was added to 1X10^6 PBMCs and incubated for 30 minutes at 4°C. Then, 40µl of magnetic beads was added and further incubated for 30 minutes at 4°C. LS columns placed on the MACS station were rinsed with 3 ml RPMI 1640. PBMCs were then added, the column was then rinsed with 5 ml medium and the flow-through was collected in 15 mL falcon tubes (Greiner Bio-One, #188261). The flow-through was centrifuged at 1500 rpm, 5 minutes at 4°C and the pellet resuspended in 1 NK-cell medium. NK cells were counted on a hemocytometer and viability was assessed using Trypan blue (Gibco, #208478). NK cells were adjusted to 2.5 x 10^5 NK cells/mL and stored in ice-cold NK-cell media until use.

Opsonic phagocytosis activity (OPA) assay

For the opsonic phagocytosis activity (OPA) assay a suspension of $MSP1_{FL}$-coupled phycoerythrin (PE)-fluorescent beads (Polysciences, #18660-5) was added to U-bottomed 96-well plates (NeoLab, #FE-0181) and opsonized for 1 hour at 37°C with serum at a 1:1,000 dilution in PBS. The plates were then centrifuged at 2000 x g for 7 minutes and washed with PBS manually. Opsonized beads were resuspended in OPA medium (RPMI 1640 with 2mM L-glutamine, 25mM HEPES, 10% FBS, and 1% penicillin-streptomycin) before being incubated with 5 x 10^4 of either THP1 cells or freshly-isolated neutrophils (as described above) at 37°C. Phagocytosis was halted by centrifugation at 1200 rpm for 7 minutes at 4°C, followed by washing with ice-cold buffer (0.5% BSA and 2 mM EDTA in PBS). The THP1 cells/neutrophils

were then resuspended in 2% paraformaldehyde (Pierce^{TM}, #20908) in PBS, and the proportion of phagocytes containing PE-fluorescent beads was analyzed using a FACS Canto II (BD Biosciences). Data analysis was performed with the FlowJo V10 software.

Antibody-dependent respiratory burst (ADRB) assay

Opaque 96-well Lumitrac microplates (Greiner Bio-One, #10154961) were coated with $MSP1_{FL}$ at 100 nM in PBS as explained above or with or freshly isolated merozoites (please see section “merozoites isolation”). The plates were then washed with 200 µl/well of PBS and blocked with 1% casein in PBS. Plates were incubated at 37°C for 1 hour, followed by the addition of 50 µL of volunteer’s serum from every timepoint at a 1:25 dilution in PBS per well. After a subsequent 1-hour incubation at 37°C, the plates were washed with PBS and luminol (Sigma, #A8511-5G) at 0.04 mg/mL was added to each well at a volume of 50 µL. Freshly isolated neutrophils were introduced in the wells at a concentration of 10^7 cells/mL, and chemiluminescence activity measured in relative light units (RLU) was measures using the Biotek Cytation 3 reader, with readings taken every 2 minutes over the course of 1 hour.

NK cell activity assays

$MSP1_{FL}$-coated plates were washed with PBS and blocked with 1% casein/PBS for 4 hours at 37°C. Following this, the plates were washed again and incubated with volunteer´s serum at 1:XXX dilution for 2 hours at 37°C. Next, 5 x 10^4 freshly isolated human NK cells were added to the plates along with a 1:70 dilution of anti-human CD107a PE (BD Biosciences, # 560948), a 1:200 dilution of brefeldin A (Sigma-Aldrich, # B6542), and a 1:200 dilution of monensin (Sigma, # 22373-78-0) and incubated for 18 hours at 37°C. The cells were then transferred to 96-well V-bottom plates, centrifuged at 1500 rpm for 5 minutes at 4°C, and washed with ice-cold buffer (0.5% BSA and 2 mM EDTA (BD Biosciences, # 15816118) in PBS). NK cells viability was assessed using XXX dye (XXX, #). NK cell surface markers were stained with an antibody cocktail of a 1:17 dilution of anti-CD56 APC (BD Biosciences) and a 1:33 dilution of anti-CD3 PE-Cy5 (BD Biosciences, #) for 30 minutes at 4°C in the dark. After washing, NK cells were fixed and permeabilized at 4°C using the Cytofix/Cytoperm™ Fixation/Permeabilization Kit (BD Biosciences, #554714) for 10 minutes at 4°C. Intracellular IFN-γ was detected by staining with a 1:33 dilution of anti-IFN-γ PE-Cy7 (BD Biosciences, #560924) for 1 hour at 4°C. After washing with Cytofix/Cytoperm, NK cells were resuspended and their activity (proportion of NK cells with CD107a and/or IFN-γ staining) was measured using a FACS Canto II (BD Biosciences). Data analysis was performed using FlowJo V10 software.

Plasmodium falciparum in vitro **culture and synchronization**

A *P. falciparum* 3D7 strain laboratory line was cultured in 12 ml of culture medium (Cmed) containing: RPMI 1640 medium supplemented with 2mM L-Glutamine and 25mM HEPES (Gibco by life technologies, #52400-041), 0,5% Albumax (Thermo Fisher, #11020021), 0.1 mM hypoxanthine (c.c. pro GmbH, #Z-41-M), gentamycin (c.c. pro GmbH #Z-28-M)) in 25 cm^2 culture flasks (Greiner, #95767) at a hematocrit of 4% using freshly-obtained human O+ erythrocytes (Bloodbank Heidelberg). The cultures were maintained in an atmosphere of 5% CO_2, 5% O_2, and 90% N_2 at 37°C. To assess parasitemia, 0.5 ml of the culture was centrifuged at 300 x g for 3 minutes, and the supernatant was discarded. A drop of the resulting pellet diluted 1:1 with RPMI was placed on a glass slide, smeared with another slide, fixed, and stained using the Hemacolor staining kit (Sigma Aldrich #1.11661). At least 300 uninfected and infected erythrocytes were counted under a microscope to determine the level of parasitemia and to identify the different stages of the parasite.

$$Parasitemia = \frac{infected\ erythrocytes}{total\ erythrocytes} * 100$$

Plasmodium falciparum **Growth Inhibition Assay (GIA)**

A *P. falciparum* 3D7 culture was synchronized with 5% D-sorbitol (Chem solute Bio, #8125.1000) to schizont stage and then adjusted to 0.6% parasitemia in 4% hematocrit using Cmed. Serum from volunteers at different timepoints was added to 25 µl of parasite suspension in Cmed per well. The sera samples were pipetted in duplicate into 96-well U-bottom plates. Rabbit IgG against AMA-1 (DiCo) (purified IgG from rabbits immunized with a mixture of 7 AMA-1 alleles, BioGenes, Berlin, Germany) and WHO (NIBSC, #10/198) standards served as positive controls, while plain erythrocytes were used as a background control. For the parasite control wells, 25 µl of Cmed and 25 µl of the parasite suspension were added to the wells without antibodies. The plates were incubated at 37°C in a gas chamber with 5% CO_2, 5% O_2, and 90% N_2 for one parasite cycle (40-48 hours). After incubation, each well was resuspended with cold PBS, and the plates were frozen at -20°C to lyse the cells. Following thawing, each well was resuspended with a pLDH (plasmodium Lactate DeHydrogenase) substrate buffer containing an NBT tablet (2 mg/10 ml) (Sigma-Aldrich #N5514-25TAB), 50 µl APAD (stock 10 mg/ml) (Sigma Aldrich #A5251), and 200 µl Diaphorase (stock 50 units/ml) (Sigma Aldrich #D5540-300UN) for biochemical detection of pLDH. The OD was measured at 650 nm using a Cytation 3 microplate reader. Inhibition was calculated using the following formula:

$$Inhibition\ [\%] = 100 - \frac{(A650\ serum\ sample - A650\ erythroc.\ control)}{(A650\ parasite\ control - A650\ erythoc.\ control)}\ x\ 100\%$$

Isolation of P. falciparum merozoites

Merozoites of *Plasmodium falciparum* strains 3D7 and FCB1 were isolated as previously described (Boyle et al., 2010). Highly synchromized P. falciparum were cultured as explained above until a yield of 4 mL pellet volume of >5% parasitemia of mature-trophozoites was achieved. Mature-stage trophozoites were then purified using LS MACS magnetic columns (Milteny Biotec, 130-042-401). These trophozoites contain hemozoin crystals—a by-product of hemoglobin digestion—which allow them to be retained in the magnetic field, while uninfected erythrocytes and ring-stage parasites pass through (Mata-Cantero et al., 2014). After extensive washing with RPMI, the column was removed from the magnet to elute the purified trophozoites. Purified trophozoites were resuspended in fresh complete culture medium and cultured to progress to schizont stage. Mature schizonts were then passed through 1.2 µm filters into 50 mL Falcon tubes pre-blocked with 1% casein in phosphate-buffered saline (PBS) to harvest merozoites. The merozoites were pelleted by centrifugation at 4,000 × g for 15 minutes and resuspended in PBS to proceed to coat the assay plates.
