# Supplementary material for: Full-length merozoite surface protein 1 formulated with GLA-SE adjuvant in malaria pre-exposed adults: a randomised, controlled, double-blind, parallel-group, single-centre Phase Ib trial
Source: eClinicalMedicine. 2025 Oct 25;89:103585. doi: 10.1016/j.eclinm.2025.103585 (PMC12595093; doi:10.1016/j.eclinm.2025.103585)
Supplement: Protocol [file mmc3.pdf]

Clinical Study Protocol

"A randomised, controlled, double-blind, parallel group, single center Phase Ib trial to assess safety, reactogenicity, immunogenicity and parasite growth rates after Controlled Human Malaria Infection of a candidate dual-stage malaria vaccine, SumayaVac-1 (MSP-1 with GLA-SE as adjuvant) in healthy malaria exposed adults of African origin aged 18-45 years"

**Protocol Number**	**P2267-22**		
**Version Number**	1.1	**Document Date**	12.09.2022
**Study Registration**	Clinicaltrial.gov<br>TMDA registry		
**Sponsor Contact**	Swiss Tropical and Public Health Institute<br>Kreuzstrasse 2, 4123 Allschwil, Switzerland<br>Tel: +41 61 284 81 11		
**Principal Investigator**	Dr. Ally Olotu<br>Ifakara Health Institute (IHI)<br>P.O. Box 74, Bagamoyo, Tanzania<br>Email: aolotu@ihi.or.tz<br>Tel: +255 718 927 104		
**Funding Agency**	Sumaya Biotech GmbH & Co. KG		
**Investigational medicinal product**	SumayaVac-1 (SUM-101)		
**Short Study Title**	Assessment of the malaria vaccine candidate SumayaVac-1 in healthy adults aged 18-45 years living in a malaria endemic country		

The information contained in this document is confidential. It is intended solely for the Investigators, potential Investigators, consultants, or applicable Independent Ethics Committees and Regulatory Authorities. It is understood that this information will not be disclosed to others without prior written authorisation from the Sponsor, except where required by applicable local laws.

Based on Protocol Template, Swissethics-WHO for Clin Trials_v3.1_06.07.2020

1 GENERAL INFORMATION

I. List of Investigators and other persons involved

Names and titles of key persons in study

Names	Institution	Position	Function in study
Dr. Ally Olotu	Ifakara Health Institute	Senior Research Scientist, Program Leader	Principal Investigator
Dr. Florence Milando	Ifakara Health Institute	Research Scientist	Investigator
Dr. Maxmillian Mpina	Ifakara Health Institute	Project Leader	Investigator
Dr. Neema Balige	Ifakara Health Institute	Research officer	Investigator
Dr. Omary Hassan	Ifakara Health Institute	Research officer	Investigator
Dr. Saumu Ahmed	Ifakara Health Institute	Research Scientist	Investigator
Dr. Bakari Mwalimu	Ifakara Health Institute	Research officer	Investigator
Jane Nyandele	Ifakara Health Institute	Research Scientist	Investigator
Sarah Mswata	Ifakara Health Institute	Research Scientist	Investigator

II. Signatures

Sponsor Representative

Signature	DocuSigned by:<br>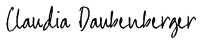<br>Signer Name: Claudia Daubenberger<br>Signing Reason: I approve this document<br>Signing Time: 16-Sep-2022 | 09:18:48 CEST<br>5D[illegible]59854D	Date of Signature<br><br>16-Sep-2022 | 09:18:52 CEST
Name	Prof. Dr. Claudia Daubenberger	
Title	Scientific Lead	
Institution	Swiss Tropical and Public Health Institute	
Address	Kreuzstrasse 2<br>4123 Allschwil<br>Switzerland	
Phone	+41 61 284 82 17	

Medical responsible

Signature	DocuSigned by:<br>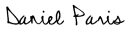<br>Signer Name: Daniel Paris<br>Signing Reason: I approve this document<br>Signing Time: 16-Sep-2022 | 09:04:27 CEST<br>6[illegible]B115E05CD1DA9875	Date of Signature<br><br>16-Sep-2022 | 09:04:32 CEST
Name	Prof. Dr. Daniel Paris	
Title	Chief Medical Officer	
Institution	Swiss Tropical and Public Health Institute	
Address	Kreuzstrasse 2<br>4123 Allschwil<br>Switzerland	
Phone	+41 61 284 81 23	

Project Responsible

Signature	DocuSigned by:<br>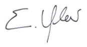<br>Signer Name: Eric Huber<br>Signing Reason: I approve this document<br>Signing Time: 16-Sep-2022 | 08:07:25 CEST<br>[illegible]3EE1094663965A1E67D747FA3F	Date of Signature<br><br>16-Sep-2022 | 08:07:29 CEST
Name	Eric Huber	
Title	Project Leader	
Institution	Swiss Tropical and Public Health Institute	
Address	Kreuzstrasse 2<br>4123 Allschwil<br>Switzerland	
Phone	+41 61 284 89 72	

Statistician

Signature	DocuSigned by: Fiona Vanobberghen<br>Signer Name: Fiona Vanobberghen<br>Signing Reason: I have reviewed this document<br>Signing Time: 19-Sep-2022 | 09:10:02 CEST<br>39E[illegible]BC3B84712F5365C8	Date of Signature<br>19-Sep-2022 | 09:10:04 CEST
Name	Fiona Vanobberghen	
Title	Statistician	
Institution	Swiss Tropical and Public Health Institute	
Address	Kreuzstrasse 2<br>4123 Allschwil<br>Switzerland	
Phone	+41 61 284 87 16	

Industrial Sponsor representative

Signature	DocuSigned by: Ernst Böhnlein<br>Name des Unterzeichners: Ernst Böhnlein<br>Signiergrund: Ich genehmige dieses Dokument<br>Signierzeit: 16-Sep-2022 | 10:31:59 MESZ<br>48[illegible]1EE946789C0866152F0	Date of Signature<br>16-Sep-2022 | 10:32:09 MESZ
Name	Dr. Ernst Böhnlein	
Title	Managing Director	
Institution	Sumaya Biotech GmbH & Co. KG	
Address	Vangerowstrasse 20<br>69115 Heidelberg<br>Germany	
Phone	+49 6221 5 88 04 20	

Principal Investigator(s)

- I have read this protocol and agree that it contains all necessary details for carrying out this study. I will conduct the study as outlined herein and will complete the study within the time designated.
- I will ensure that all individuals and parties contributing to this study are qualified and I will implement procedures to ensure integrity of study tasks and data.
- I will provide copies of the protocol and all pertinent information to all individuals responsible to me who assist in the conduct of this study. I will discuss this material with them to ensure they are fully informed regarding the drug and the conduct of the study.
- I will use only the informed consent forms approved by the Sponsor or its representative and will fulfil all responsibilities for submitting pertinent information to the Independent Ethics Committees responsible for this study.
- I agree that the Sponsor or its representatives shall have access to any source documents from which Case Report Form information may have been generated.
- I agree to conduct the study in compliance with the current version of the Declaration of Helsinki, ICH-GCP E6(R2) as well as all the national legal and regulatory requirements.

Principal Investigator

Signature	DocuSigned by: 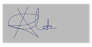 Signer Name: Ally Olotu<br>Signing Reason: I approve this document<br>Signing Time: 21-Sep-2022 | 09:44:14 CEST	Date of Signature<br>21-Sep-2022 | 09:44:24 CEST
Name	Dr. Ally Olotu 3BE062[illegible]235F4CA5803E77C4D831A97E	
Title	Principal Investigator	
Institution	Ifakara Health Institute (IHI)	
Address	P.O. Box 74, Bagamoyo, Tanzania	
Phone	+255 718 927 104	

III. Table of contents

1 General Information ... 2
I. List of Investigators and other persons involved ... 2
II. Signatures ... 3
III. Table of contents ... 6
IV. Abbreviations ... 9
V. Synopsis ... 12
2 Background information ... 21
2.1 Malaria disease ... 21
2.2 Rational for MSP-1 vaccine ... 21
2.3 General treatment/ Administration ... 22
2.4 Efficacy ... 22
2.5 Scientific justification - Pharmacodynamics & Pharmacokinetics ... 23
2.6 Immunogenicity studies ... 23
2.7 Safety and tolerability ... 24
2.8 Toxicity ... 24
2.9 Justification for the dosage and dosage plan, and the length of treatment ... 24
3 Objectives and purpose ... 24
3.1 Study rationale and objectives ... 24
3.1.1 Study rationale ... 24
3.1.2 Primary objectives ... 24
3.1.3 Secondary objective ... 25
3.1.4 Exploratory objectives ... 25
3.2 Scientific justification and rationale ... 25
3.2.1 Scientific justification - study population ... 25
3.2.2 Scientific justification - vaccinations ... 26
3.2.3 Scientific justification - CHMI ... 26
3.2.4 Scientific justification - Dose ... 26
3.2.5 Scientific justification for deviation from Verorab® dosing schema ... 26
3.2.6 Scientific justification- retrospective helminth infection analysis ... 27
4 Study design ... 27
4.1 Primary and secondary endpoints ... 29
4.1.1 Primary endpoints ... 29
4.1.2 Secondary endpoints ... 30
4.1.3 Exploratory endpoints ... 30
4.2 Measures to minimize bias ... 31
4.2.1 Randomization ... 31
4.2.2 Double blinding of vaccination and unblinding ... 31
4.2.3 Masking of syringe ... 32
4.3 Study duration and duration of participant's participation ... 32
4.3.1 Schedule of events ... 33
4.4 Early termination of the study ... 34
4.5 Stopping rules ... 34
4.5.1 Study pause at any time point ... 34
4.5.2 Study pause at defined time-points ... 35
4.5.3 Study pause for individual participants before CHMI ... 35
4.6 Temporary contraindications for continuing vaccination/ CHMI ... 35
5 Selection of the study participants ... 36
5.1 Study setting ... 36
5.2 Recruitment ... 36
5.3 Inclusion criteria ... 37
5.4 Exclusion criteria ... 37
5.5 Criteria for discontinuation of study ... 38
5.5.1 Withdrawal and discontinuation of individual participants ... 38
5.5.2 Discontinuation because stopping rule is met ... 38
6 Treatment of participants ... 39
6.1 Identity of investigational products ... 39
6.1.1 Experimental intervention vaccination part ... 39
6.1.2 Experimental intervention CHMI part ... 39
6.2 Comparator ... 40
6.3 Packaging, labelling and supply ... 40
6.4 Storage conditions ... 40
6.5 Administration of experimental and comparator interventions ... 40
6.5.1 Experimental intervention - vaccination part ... 40

6.5.2 Experimental intervention - CHMI part 41
6.6 Dose modifications 41
6.7 Compliance with study interventions 41
6.8 Concomitant interventions (treatments) 41
6.9 Experimental Intervention accountability 42
6.10 Return or destruction of experimental Intervention 42
7 Study assessments 42
7.1 Procedures per visit 42
7.1.1 General order of assessments 42
7.1.2 Screening visit 1 (D-35 to D-1) 42
7.1.3 Screening visit 2 (D-35 to D-1) 43
7.1.4 Pre 1st Vaccination (D-1 to D0) 43
7.1.5 1st, 2nd and 3rd Vaccination visit (D0, D28 and D56) 44
7.1.6 1-6 days post 1st, 2nd and 3rd vaccination follow-up (D1-6, D29-34 and D57-62) 44
7.1.7 7 day site visit post 1st, 2nd and 3rd vaccination (D7, D35 and D63) 44
7.1.8 14 day site visit post 1st, 2nd and 3rd vaccination (D14, D42 and D70) 45
7.1.9 28 day site visit post 1st, 2nd and 3rd vaccination (D28, D56 and D84) 45
7.1.10 CHMI (D84) 45
7.1.11 1-4 days post CHMI (D85-88) 46
7.1.12 Site stay post CHMI (D89- maximum D115) 46
7.1.13 Malaria treatment post-CHMI (between D89-112) 47
7.1.14 Site discharge post CHMI confinement (between D89-115) 48
7.1.15 Vaccination site follow-up (D112, D140) and CHMI long-term site follow-up visits (D168, D252) 48
7.1.16 Unscheduled visit 49
7.2 Total blood volume 49
8 Assessment of safety 49
8.1 Safety assessments 49
8.1.1 Physical Examination 49
8.1.2 Vital Signs 50
8.1.3 12-lead ECG safety recording 50
8.1.4 Laboratory assessments 50
8.2 Collection and reporting of adverse events 51
8.2.1 Definition of adverse events (AEs) 51
8.2.2 Types and recording of AEs 51
8.2.3 Grading of adverse events severity 51
8.2.4 AE causality assessment 52
8.2.5 Serious adverse events (SAEs) 52
8.2.6 Expectedness of AEs 52
8.2.7 Reporting Obligations 53
8.3 Follow-up of adverse events 54
8.4 Data collection and follow-up of prematurely terminated participants 54
9 Statistics 54
9.1 Hypothesis 54
9.2 Determination of sample size 54
9.3 Description of statistical methods 54
9.3.1 Datasets to be analysed, analysis populations 55
9.4 Handling of data 56
10 Ethical considerations 56
10.1 Independent Ethics Committee (IEC) 56
10.2 Regulatory Authority (RA) 57
10.3 Evaluation of the risk-benefit ratio 57
10.4 Participant information and consent 57
10.5 Registration of clinical trial 58
10.6 Participant confidentiality 58
10.7 Participants requiring particular protection 58
10.8 Insurance 58
10.9 Participant reimbursement 59
10.10 Protocol amendments 59
11 Quality control and quality assurance: description of measures 59
11.1 Data handling and record keeping / archiving 59
11.1.1 Case Report Forms 59
11.1.2 Specification of source documents 59
11.1.3 Record keeping/ archiving 60
11.2 Data management 60
11.2.1 Data management system 60
11.2.2 Data security, access and back-up 60
11.2.3 Analysis and archiving 60

11.2.4 Electronic and central data validation 60
11.3 Risk management 61
11.3.1 Risk identification, assessment and mitigation 61
11.3.2 Study-specific preventive measures 61
11.4 Safety monitoring committee (SMC) 62
11.5 Translations - Reference language 62
11.6 Storage of biological material and related health data 63
12 Funding 63
13 Dissemination of results and publication policy 63
14 References 65
15 Appendices 68
15.1 Laboratories analysing biological materials 68

IV. Abbreviations

AAHI	Access to Advanced Health Institute
ADCC-NK cells	Antibody-Dependent Cell-Mediated Cytotoxicity Natural Killer Cells
ADRB	Antibody-Dependent Respiratory Burst
AE	Adverse Event
ALT	Alanine Aminotransferase
ALU	Artemether/ Lumefantrine
AST	Aspartate Aminotransferase
A-V heart block	Atrioventricular heart block
BCTF	Bagamoyo Clinical Trial Facility
BMI	Body Mass Index
BP	Blood Pressure
BRTC	Bagamoyo Research Trial Center
CD4	Cluster of Differentiation 4
CD8	Cluster of Differentiation 8
CHMI	Controlled Human Malaria Challenge
CRF	Case Report Form
CRP	C-Reactive Protein
CTCAE	Common Terminology Criteria for Adverse Events
DVI	Direct Venous Inoculation
E. coli	Escherichia coli
ECG	Electrocardiogram
eCRF	Electronic Case Report Form
EDC	Electronic Data Capture
EKNZ	Ethikkommission Nordwest- und Zentralschweiz
ELISA	Enzyme-Linked Immunosorbent Assay
ELISpot	Enzyme-Linked Immune Absorbent Spot
GCP	Good Clinical Practice
GIA	Growth Inhibition Assay
GLA-SE	Glucopyranosyl Lipid Adjuvant-Stable Emulsion
GMP	Good Manufacturing Practice
GPA	Glycophorin A
GPI	Glycosylphosphatidylinositol
ɣδ T-cell	Gamma Delta T-cells
HBV	Hepatitis B Virus
HCV	Hepatitis C Virus
HIV	Human Immunodeficiency Virus

HR	Heart Rate
i.m.	Intramuscular
ICF	Informed Consent Form
ICH	International Council on Harmonisation of Technical Requirements for Registration of Pharmaceuticals for Human Use
ICS	Intracellular Cytokine Staining
ID	Identification
IEC	Independent Ethics Committee
IFNγ	Interferon Gamma
IgG	Immunoglobulin G
IgM	Immunoglobulin M
IHI	Ifakara Health Institute
IL-10	Interleukin-10
IMP	Investigational Medicinal Product
INN	International Nonproprietary Name
IRB	Institutional Review Board
ISF	Investigator Site File
ITT	Intent to Treat
IU	International Unit
IUD	Intrauterine Device
IUS	Intrauterine Hormonal Releasing System
Kg	Kilogram
m^2	meter square
MAC	Membrane Attack Complex
MD	Doctor of Medicine
MHC	Major Histocompatibility Complex
ml	Millilitre
MSP-1	Merozoite Surface Protein-1
MSPDBL	MSP Duffy Binding-Like
NaCl	Sodium Chloride
NatHREC	National Health Research Ethics Review Committee
NOAEL	No Observed Adverse Effect Level
PBMC	Peripheral Blood Mononuclear Cells
PfSPZ	Plasmodium falciparum Sporozoites
PhD	Doctor of Philosophy
PI	Principal Investigator
PMR	Parasite Multiplication Rate

PV	Parasitophorous Vacuoles
qPCR	Quantitative Polymerase Chain Reaction
RA	Regulatory Authority
RBC	Reb Blood Cell
RDT	Rapid Diagnostic Test
ROS	Reactive Oxygen Species
RR	Respiratory Rate
SAE	Serious Adverse Event
SMC	Safety Monitoring Committee
SOP	Standard Operating Procedure
SUSAR	Suspected Unexpected Serious Adverse Reaction
Swiss TPH	Swiss Tropical and Public Health Institute
TBS	Thick Blood Smear
TH1	Type 1 T Helper
TMDA	Tanzania Medicines and Medical Devices Authority
TMF	Trial Master File
TSH	Tanzanian Shilling
USD	United States Dollar
WBC	White Blood Cell
WHO	World Health Organization
WOCBP	Women of Child Bearing Potential
µg	Microgram
µl	Microlitre

V. Synopsis

**Sponsor**	Swiss Tropical and Public Health Institute
**Project Lead**	Claudia Daubenberger & Eric Huber
**Study Title**	A randomised, controlled, double-blind, parallel group, single center Phase Ib trial to assess safety, reactogenicity, immunogenicity and parasite growth rates after Controlled Human Malaria Infection of a candidate dual-stage malaria vaccine, SumayaVac-1 (MSP-1 with GLA-SE as adjuvant) in healthy, malaria exposed adults of African origin aged 18-45 years
**Short Title**	Assessment of the malaria vaccine candidate SumayaVac-1 in healthy adults aged 18-45 years living in a malaria endemic country
**Protocol Version and Date**	v1.1, 12 September 2022
**Trial registration**	ClinicalTrials.gov (pending registration)<br>TMDA registry
**Study Category with Rationale**	Risk category according to HRA (Clin O) - category C<br>Investigational Medicinal Product is not authorized for use
**Clinical Phase**	Phase Ib
**Background and Rationale**	Malaria remains a major infectious disease causing a heavy burden of mortality and morbidity in populations living in tropical and subtropical regions. However, despite important insights gained in different vaccine development approaches, there is currently no vaccine with sufficient and long-lasting protection against malaria. The Phase Ia results of the proposed vaccine candidate showed promising results in Caucasian participants, however, results in the population living in the malaria affected regions are needed.

**Objective(s)**	**To evaluate in healthy adults of African origin previously exposed to the malaria parasite receiving SumayaVac-1 (SUM-101) versus rabies control (Verorab®) vaccine:**<br>**Primary Objectives**<br>• Safety and reactogenicity of SumayaVac-1 (SUM-101).<br>• Immunogenicity of SumayaVac-1 (SUM-101).<br>**Secondary Objectives**<br>• The relationship of SumayaVac-1 (SUM-101) vaccine-induced antibody levels, *in vitro* effector functions and isotype distribution with asexual blood stage parasite growth rates after homologous controlled human malaria infection (CHMI).<br>**Exploratory Objectives**<br>• Comparison of SumayaVac-1 (SUM-101) induced immunoglobulin isotype distribution and duration between malaria pre-exposed and malaria naïve participants from the previous Phase Ia study in Heidelberg.<br>• Fine scale epitope mapping of SumayaVac-1 (SUM-101) specific antibodies, including specific CD4+ and CD8+ T-cell epitopes using peptide arrays, to investigate the association between cellular immune response with humoral seroconversion and investigate the potential association with protection after homologous CHMI.<br>• Comparison of SumayaVac-1 (SUM-101) induced cellular immunity between malaria pre-exposed and malaria naïve participants from the previous Phase Ia study in Heidelberg.<br>• Investigation of the B- and T-Cell repertoire before and after SumayaVac-1 (SUM-101) vaccination, as well as after homologous CHMI.<br>• Integrated transcriptome and immunoglobulin gene repertoire analyses of MSP-1 specific B-cells using single-cell technologies.<br>• Glycosylation patterns of MSP-1 specific functional antibodies.<br>• Investigate off-target IgG and IgM repertoire after SumayaVac-1 (SUM-101) vaccination using immunoproteomics and their association with protection after homologous CHMI.<br>• Investigate the structure of MSP-1 protein bound to functional antibodies by cryo-electron tomography to map conformational epitopes.<br>• Mechanisms of malaria specific antibody diversity generation, post-translational modification of antibodies, and B- and T-cell memory generation and maintenance.<br>• Human and parasite transcriptome in peripheral blood during early stage asexual blood stage parasitemia after homologous CHMI.<br>• The quality and quantity of peripheral blood γδ T-cell responses in participants with or without homologous CHMI (all 40 participants).<br>• Liver to blood inoculum size of the CHMI-induced parasites after homologous CHMI (25 participants that undergo homologous CHMI).
**Primary, Secondary and Exploratory Endpoints**	**Primary endpoints:**<br>The following endpoints will be measured in all participants to evaluate the safety and reactogenicity of SumayaVac-1 (SUM-101):<br>• Local and systemic solicited adverse events (AEs) at least possibly related to the investigational medicinal product (IMP) recorded after each vaccination (done on D0, D28 and D56) up to 7 days later.

	• Local and systemic unsolicited reactogenicity recorded after each vaccination (done on D0, D28 and D56) up to 28 days later.<br>• Any serious adverse events (SAE) occurring after the 1st vaccination until the participant's last visit.<br>• Changes in laboratory safety parameters between baseline (D0 before 1st vaccination) to 28 days after each of the vaccinations.<br>• Changes in laboratory safety parameters between values just prior to each vaccination (on D0, D28 and D56) and values 28 days after that vaccination.<br>The following endpoints will be measured in all participants at D0 pre-vaccination, D28 (W4), D56 (W8), D84 (W12), D112 (W16), D140 (W20), and additionally at D168 (W24) and D252 (W36) for participants that undergo homologous CHMI, to evaluate the humoral immunogenicity:<br>• Longevity of antibody responses to SumayaVac-1 (SUM-101) by ELISA.<br>• Fold change of antibody responses to SumayaVac-1 (SUM-101) in comparison to baseline (D0 pre-vaccination).<br>**Secondary endpoints:**<br>The following endpoints will be measured in the sera or blood of all participants, at D0 pre-vaccination, D28 (W4), D56 (W8), D84 (W12), D112 (W16), D140 (W20), and additionally at D168 (W24) and D252 (W36) for participants that undergo homologous CHMI:<br>• Evaluation of the opsonic phagocytosis activity.<br>• Evaluation of complement fixation, activation and/or membrane attack complex (MAC) formation.<br>• Evaluation of antibody-dependent respiratory burst (ADRB) activity.<br>• Evaluation of antibody-dependent cellular cytotoxicity (ADCC-NK cells) activity.<br>• Evaluation of immune-mediated growth inhibition activity on a panel of *Plasmodium falciparum (P.falciparum)* lines.<br>• Development of *P.falciparum* parasitemia (pre-patent period, parasite multiplication rate (PMR) and percentage of parasite negative participants in SumayaVac-1 (SUM-101) versus Verorab® arm) by thick blood smear (TBS).<br>• Development of *P.falciparum* parasitemia (pre-patent period, PMR and percentage of parasite negative participants in SumayaVac-1 (SUM-101) versus Verorab® arm) by qPCR.<br>The following endpoints will be measured in all participants:<br>• Cellular immune responses to SumayaVac-1 (SUM-101) by (i) CD4+ and CD8+ ELISpot assays, (ii) SumayaVac-1 (SUM-101) specific cells characterised by flow cytometry-based immunophenotyping using intracellular cytokine staining (ICS), (iii) functional gene expression analysis, and/or other assays to be defined; at D0 pre-vaccination and D56 (W8), D84 (W12), D112 (W16) and D140 (W20).<br>The following endpoint will be assessed among SumayaVac-1 (SUM-101) participants only:<br>• Comparison of MSP-1 IgG antibody concentrations by ELISA between malaria pre-exposed participants in the current study and malaria naïve participants from the previous Phase Ia study in Heidelberg.

	**Exploratory endpoints:**<br>• Comparison of SumayaVac-1 (SUM-101) induced immunoglobulin isotype distribution and duration between malaria pre-exposed and malaria naïve participants from the previous Phase Ia study in Heidelberg.<br>• Fine scale epitope mapping of SumayaVac-1 (SUM-101) specific antibodies, including specific CD4+ and CD8+ T-cell epitopes using peptide arrays, to investigate the association between cellular immune response with humoral seroconversion and investigate the potential association with protection after homologous CHMI.<br>• Comparison of SumayaVac-1 (SUM-101) induced cellular immunity between malaria pre-exposed and malaria naïve participants from the previous Phase Ia study in Heidelberg.<br>• Investigation of the B- and T-Cell repertoire before and after SumayaVac-1 (SUM-101) vaccination, as well as after homologous CHMI.<br>• Integrated transcriptome and immunoglobulin gene repertoire analyses of MSP-1 specific B-cells using single-cell technologies.<br>• Glycosylation patterns of MSP-1 specific functional antibodies.<br>• Investigate off-target IgG and IgM repertoire after SumayaVac-1 (SUM-101) vaccination using immunoproteomics and their association with protection after homologous CHMI.<br>• Investigate the structure of MSP-1 protein bound to functional antibodies by cryo-electron tomography to map conformational epitopes.<br>• Measurement of asexual blood stage pre-patent period in participants having received SumayaVac-1 (SUM-101) versus Verorab® rabies vaccine after homologous CHMI.<br>• *Ex vivo* assessment of asexual blood stage parasite transcriptome during homologous CHMI in participants having received SumayaVac-1 (SUM-101) versus Verorab® rabies control vaccine.<br>• *Ex vivo* assessment of changes in human peripheral blood transcriptome before, during and after homologous CHMI in participants having received SumayaVac-1 (SUM-101) versus Verorab® rabies vaccine.<br>• Description of ɣδ T-cell receptor repertoire, transcriptome, functional activity and phenotypes before, during and after homologous CHMI in participants having received SumayaVac-1 (SUM-101) versus Verorab® rabies vaccine.<br>• Investigate the impact of the presence of intestinal helminth infections on vaccine-induced humoral immune response by comparing the quality and quantity of SumayaVac-1 (SUM-101) specific antibody isotypes between helminth infected and non-infected participants at baseline.<br>• Investigate the impact of the presence of intestinal helminth infections on SumayaVac-1 (SUM-101) vaccine-induced cellular immune responses by comparing the quality and quantity of SumayaVac-1 (SUM-101) specific cytokine production and ICS results between helminth infected and non-infected participants at baseline.
**Study Design**	This is a randomised, controlled, double-blind, parallel group, single center Phase Ib trial to assess the safety, reactogenicity, and immunogenicity and parasite growth rates after homologous CHMI of SumayaVac-1 (SUM-101) in healthy, malaria-exposed adults of African origin aged 18-45 years.

	The study is divided in two parts:<br>**Vaccinations (Part 1)**<br>In total, 40 participants will be enrolled (male and female). 20 participants will be randomised to receive three monthly inoculations (on D0, D28 and D56) with the investigational product, SumayaVac-1 (SUM-101), and 20 participants will be randomised to receive the registered rabies vaccine, Verorab®, as controls.<br>For operational reasons the participants will be split in two groups of 20 participants.<br>• **Group 1** will have a sentinel subgroup (2 SumayaVac-1 (SUM-101) & 1 Verorab® rabies vaccine) with a 48 hours safety surveillance period before the remaining 17 participants (8 SumayaVac-1 (SUM-101) & 9 Verorab® rabies vaccine) of the group 1 receive their 1st vaccination.<br>• **Group 2** will be composed of 20 participants (10 SumayaVac-1 (SUM-101) & 10 Verorab® rabies vaccine). All visits in group 2 will be shifted by 3 weeks compared to group 1 to create minimal overlap of study-related activities.<br>After each vaccination (done on D0, D28 and D56), the participants will remain at the facility for 2 hours prior to their discharge for home. The participant will be called daily by phone (or home visits if required) until 6 days post vaccination for follow-up.<br>For all participants, vaccination follow-up visits at the site will occur at 7, 14 and 28 days after each vaccination.<br>**CHMI (Part 2)**<br>In total, 25 participants (15 SumayaVac-1 (SUM-101) & 10 Verorab® vaccinated, selected randomly from the overall 40 participants) will undergo homologous CHMI with attenuated sporozoites 4 weeks after the 3rd vaccination.<br>For operational reasons, the participants will remain in the two separate groups defined in the vaccination part.<br>After the CHMI, the participant will stay for 2 hours at the facility before being discharged. The participant will be called daily by phone (or home visits if required) until 4 days post CHMI for follow-up.<br>5 days after CHMI, participants will return to the site and be admitted to the ward for closer observation during the period when parasitemia could possibly be detected.<br>The participant will remain in confinement at the site until he/she is considered positive for malaria or reaches 28 days post CHMI.<br>All participants will then receive anti-malarial treatment (for 3 days) and be discharged after being confirmed to be malaria negative.<br>CHMI follow-up visits at the site will take place at 3 months (D168) and 6 months (D252) post CHMI.
**Inclusion/Exclusion Criteria**	**Inclusion criteria**<br>1. Written informed consent obtained before any study procedure.<br>2. Literate participants aged 18-45 years of African origin.<br>3. Female and male participants practicing contraception from 4 weeks before 1st immunization and up to 12 weeks after the last immunization or CHMI.<br>4. Available to participate in follow-up for the duration of the study.<br>5. Contactable by phone during the whole study period.<br>6. At least two years residence in the Bagamoyo district or nearby districts in Coastal and Dar-es-Salaam regions and planning to reside there for at least 9 more months.

	7. Agreement to provide personal contact information and contact information of another household member or close friend.<br>8. Female participants must be willing to avoid pregnancy if selected for participation in the trial and to undergo multiple serum pregnancy testing.<br>9. Confirmation of understanding of design, procedures, risk and benefits of the study in a test with maximum of two attempts.<br>10. General good health based on assessment of medical history and clinical examination.<br>**Exclusion Criteria**<br>1. Previous participation in any malaria vaccine trial in the last 3 years.<br>2. Participation in any other clinical trial involving investigational medicinal products within 30 days prior to the screening assessment.<br>3. Previous history of drug or alcohol abuse interfering with normal social function within one year prior to enrolment.<br>4. Previous vaccination with a rabies vaccine.<br>5. Intake of chronic medication, especially immunosuppressive agents (steroids, immunomodulating drugs) during the 13 weeks preceding the screening visit or during the study period.<br>6. Known hypersensitivity to any of the vaccine components (adjuvant or protein) or anti-malarial treatments.<br>7. Body mass index (BMI) of <18 or >30 Kg/m^2.<br>8. Participants unable to be closely followed for social, geographic or psychological reasons.<br>9. Any vaccination from 4 weeks prior to the 1st vaccination and (none planned) up to 6 weeks after the 3rd vaccination or CHMI.<br>10. Symptoms, physical signs or laboratory values suggestive of systemic disorders, including renal, hepatic, cardiovascular, pulmonary, skin, immunodeficiency, psychiatric and other conditions, which could interfere with the interpretation of the trial results or compromise the health of the participants.<br>11. Abnormal electrocardiogram on screening: pathologic Q wave and significant ST-T wave changes, left ventricular hypertrophy, clinically significant arrhythmias, left bundle branch block, secondary or tertiary A-V (atrio-ventricular) heart block.<br>12. Any clinically significant laboratory values at screening outside of normal ranges for study participants.<br>13. Malaria positivity at screening (microscopy or qPCR positive).<br>14. Positive HIV, HBV or HCV tests.<br>15. For females: Positive pregnancy test or actively breast feeding.
**Measurements and Procedures**	Written informed consent will be taken prior to any study procedure. Healthy participants will be screened and randomised before administration of the study IMP.<br>**<u>Vaccinations (Part 1)</u>**<br>On the day of the 1st vaccination, the health status of the participant is re-checked and eligibility confirmed. Samples for safety, humoral, cellular and exploratory measurements are taken (baseline). Vaccination is administered and the participant remains at site for 2 hours before being discharged. Telephone follow-ups (or home visits if required) will be performed daily until 6 days after each vaccination. On 7, 14 and 28 days after each vaccination (done on D0, D28 and D56), the participant will be invited to come back to the facility and health status is checked.<br>Further participant follow-up visits will take place at D112 (W16) and D140 (W20) post 1st vaccination. Sampling for humoral and cellular responses

	will be performed at D112 (W16) and D140 (W20). Additionally, sampling for exploratory measurements will be performed at D112 (W16) (Vac1 + 4 month follow-up site visit).<br>**<u>CHMI (Part 2)</u>**<br>4 weeks after the 3rd vaccination, a randomly selected subset of participants (n=25; 15 SumayaVac-1 (SUM-101) & 10 Verorab® vaccinated) are invited to participate in the CHMI part of the study.<br>On the day of the CHMI, the health status of the participant is checked and eligibility confirmed. Samples for safety, cellular, humoral and exploratory measurements are taken. CHMI is performed and the participants remain at site for 2 hours before being discharged. Telephone follow-ups (or home visits if required) will be performed daily until 4 days post CHMI to check participant's health status.<br>5 days post CHMI, participants will return to the site to check the health status and for admission to the ward for closer observation.<br>During this confinement period at the site, clinical assessments and blood withdrawals will be performed until the participant is considered malaria positive (count of >500 parasites/µl in the qPCR or malaria symptoms with a positive rapid diagnostic test (RDT)) or reaches 28 days post CHMI. Blood samples will be stored for qPCR based assessment of asexual blood stage parasitemia and PMR. Once considered positive for malaria or at 28 days post CHMI (if still malaria negative), samples for exploratory measurements will be taken followed by anti-malarial treatment for 3 days. At the end of the anti-malarial treatment, the health status will be checked and blood samples will be collected to confirm asexual blood stage negativity.<br>When there is no medical contraindication, the participant will be discharged.<br>CHMI follow-up visits will take place at 3 months (D168) and 6 months (D252) post CHMI and will include health checks and sampling for humoral responses.
**Number of Participants with Rationale (if no Power Analysis conducted)**	**<u>Vaccination: 40 adult participants</u>**<br>Overall: 20 SumayaVac-1 (SUM-101) and 20 Verorab® control<br>**Group 1**<br>Sentinel sub-group<br>- 2 SumayaVac-1 (SUM-101)<br>- 1 Verorab® control<br>Follower Group<br>- 8 SumayaVac-1 (SUM-101)<br>- 9 Verorab® control<br>**Group 2**<br>- 10 SumayaVac-1 (SUM-101)<br>- 10 Verorab® control<br>**<u>CHMI: 25 from 40 adult participants who received vaccination</u>**<br>Overall: 15 SumayaVac-1 (SUM-101) and 10 Verorab® control<br>**Group 1**<br>- 12 (7 having received SumayaVac-1 (SUM-101) & 5 having received Verorab® control)<br>**Group 2**<br>- 13 (8 having received SumayaVac-1 (SUM-101) & 5 having received Verorab® control)<br>As this is a Phase I trial, no formal sample size calculation has been done. The sample size is considered sufficient to examine the safety and

	reactogenicity of SumayaVac-1 (SUM-101), and humoral and cellular immunogenicity.
**Study Product / Intervention**	<u>**Vaccination:**</u><br>Intra-muscular injection of SumayaVac-1 (SUM-101) composed of 150 µg MSP-1 + 5 µg GLA-SE<br><u>**CHMI:**</u><br>Direct venous inoculation of 3.2 x 10^3 purified, infectious *P. falciparum* sporozoites (PfSPZ Challenge (NF54 strain), Sanaria®)
**Control Intervention (if applicable)**	<u>**Vaccination control:**</u><br>Intra-muscular injection of rabies vaccine (Verorab®)
**Study Duration**	The total study duration including screening will be approximately 40 weeks including the screening period.<br>The study duration for each participant involved in the vaccinations (Part 1) and the CHMI (Part 2) (n=25) is 36 weeks.<br>For participants not undergoing CHMI (n=15) the study duration will be 20 weeks.
**Study Schedule**	First participant first visit: Sep 2022<br>Last participant last visit: Aug 2023
**Investigator(s)**	Ally Olotu
**Study Centre**	Bagamoyo Research and Training Center of the Ifakara Health Institute
**Statistical Analysis incl. Power Analysis**	See above for sample size justification.<br>To evaluate the safety and reactogenicity primary endpoints, AEs and SAEs will be presented according to the endpoint definitions above. AE reporting will include verbatim term, preferred term (PT), system organ class (SOC), treatment, severity, relationship to the interventional products, and seriousness, reporting numbers of participants experiencing each event and total numbers of each event. Laboratory safety parameters will be summarized as absolute values and changes at 28 days after each vaccination compared to baseline (before 1st vaccination) and compared to values just prior to each vaccination.<br><br>To evaluate the immunogenicity primary endpoints as defined above, SumayaVac-1 (SUM-101) vaccine induced humoral immunogenicity will be summarized as antibody responses to SumayaVac-1 (SUM-101) by ELISA over time, and fold changes of antibody responses relative to baseline.<br><br>For all analyses, data will be listed for each participant. Descriptive analyses will be performed (number of observations, arithmetic or geometric mean, standard deviation, minimum, maximum, median, interquartile range, as appropriate, for continuous data, and counts and percentages for categorical data). Results will be presented by vaccination received, overall and separately for participants who were/were not in the CHMI part.<br><br>Further details will be elaborated in a Statistical Analysis Plan (SAP), including for the secondary and exploratory objectives.
**Ethical consideration**	This study will be conducted in compliance with the protocol, the current version of the Declaration of Helsinki, the ICH-GCP as well as all national legal and regulatory requirements. This protocol will be reviewed by the

	Ethikkommission Nordwest- und Zentralschweiz (EKNZ, Ethics Committee of Northern and Central Switzerland) and also be reviewed and approved by the Ifakara Health Institute Review Board (IHI-IRB) and the National Health Research Ethics Sub-Committee (NatHREC) in Tanzania before starting the study.

2 BACKGROUND INFORMATION

2.1 Malaria disease

Malaria is a life threatening disease and considered to be one of the biggest global health burdens caused by an infectious disease, particularly in Sub-Saharan Africa [1]. The 2021 World Health Organization (WHO) malaria report estimated that there were 241 million malaria cases in 85 malaria endemic countries in 2020, an additional 14 million malaria cases compared to 2019. In total, an estimated 627,000 malaria-related deaths were reported in 2020, with the WHO African Region home to 95% of all reported malaria cases and deaths [2]. The malaria disease is caused by parasites belonging to *Plasmodium* species with *P. falciparum* accounting for 99.7% of estimated malaria cases in the WHO African Region in 2020 [2]. Malaria infections mainly affect vulnerable populations, namely children aged under 5 years, accounting for 77% of all malaria deaths, and primigravid pregnant women [2].

The parasite life cycle of malaria involves two hosts, the human host, where asexual reproduction takes place, and the mosquito vector, where sexual reproduction occurs [3]. Malaria transmission to humans is initiated through the bites of infected female Anopheles mosquitoes. During a blood meal, sporozoites of malaria parasites are inoculated into the human skin and some of these sporozoites are transported through the bloodstream to the liver [3]. In the liver, sporozoites infect hepatocytes and develop into liver stage merozoites 6-7 days after sporozoite inoculation [3]. Thousands of merozoites can then be released by each infected hepatocyte into the blood stream, where they initiate asexual reproduction by invading the red blood cells (RBCs) [3]. The invasion of RBCs by merozoites follows a sequence of complex receptor-ligand interactions and the formation of parasitophorous vacuoles (PV) after invagination of the RBC membrane [4]. This allows the parasites to enter a nutrient rich environment, highly protected by the immune system since RBCs lack the expression of Major Histocompatibility Complex (MHC) class I and MHC class II molecules. After erythrocyte invasion, merozoites develop into ring, trophozoite and schizont stages within the PV in about 48 hours in the case of *P. falciparum*, until newly developed merozoites egress by sequential rupture of the parasitophorous vacuole membrane and host cell membrane [5, 6]. Free merozoites invade new RBCs within minutes and during this short time window, the merozoite surface is exposed to factors of the immune system [4].

A number of membrane integral and peripheral proteins form a thick fibrillar coat on the merozoite surface. The Merozoite Surface Protein (MSP)-1 is one of the most abundant proteins on the merozoite surface which has been studied in detail with respect to its biology and structure [7, 8]. MSP-1 consists of four subunits, namely p83, p30, p38 and p42, which are held together non-covalently. A glycosylphosphatidylinositol (GPI) anchor links the complex to the merozoite membrane via the p42 subunit [9]. The MSP-1 complex is known to interact with a range of other MSPs, including MSP-3, MSP-6, MSP-7 and MSP-9, MSP Duffy binding-like (MSPDBL)-1 and MSPDBL-2, forming various different complexes on the parasite surface [10]. MSP-1 is essential for *P. falciparum* development given that MSP-1 knockdown mutants could not be generated *in vitro* [11]. MSP-1 might be involved in early RBC attachment and invasion of merozoites through binding to the RBC surface proteins glycophorin A (GPA) and band 3 [12, 13]. MSP-1 also interacts with the erythrocyte cytoskeleton protein spectrin and plays a role in egression from RBCs [14].

2.2 Rational for MSP-1 vaccine

Large, international research efforts have been invested into the development of anti-malaria vaccination strategies. Currently, only the first generation pre-erythrocytic malaria vaccine RTS,S (MosquirixTM, GSK Bio), based on the *P. falciparum* circumsporozoite protein, is under pilot implementation studies in three African countries [15-18]. On October 6th, 2021, RTS,S has been endorsed by WHO as a malaria vaccine for children living in moderate to high malaria transmission settings to be delivered in combination with strong ongoing vector control measures in place [19]. However, while 30% reduction of severe malaria is achieved, the protective efficacy of RTS,S is still moderate and short-lived. A 39% reduction in overall malaria incidence in a Phase III clinical study conducted in children who received four injections was achieved over a follow-up period of 48

months [20, 21]. Therefore, second generation malaria vaccine development approaches that complement RTS,S would be essential to further strengthen malaria control measures in the future.

The clinical presentation of malaria is caused by asexual blood stage parasites and immuno-epidemiological studies have shown that people living in malaria endemic areas can acquire immunity to clinical disease with time and age after repeated episodes of infection [22]. This protection is very likely based on the development of asexual blood stage parasite binding antibodies as has been demonstrated in the proof of concept study of passive antibody transfer in which transfusion of purified Immunoglobulin G (IgG) from malaria-immune adults to juvenile clinical malaria patients drastically reduced blood stage parasitemia [23]. A number of follow-up immuno-epidemiological studies have strongly suggested that antibodies targeting the proteins expressed on the merozoite surface confer protective immunity against asexual blood stage parasites [24]. Therefore, the idea of targeting the step of malaria parasite invasion of RBCs as an anti-malarial strategy has gained strong scientific support over many years [25]. Based on a number of biological, functional, epidemiological and immunological studies investigating MSP-1, this protein is currently considered a prime candidate antigen for vaccine development against malaria [25].

So far, vaccine development efforts have focused on the C-terminal p42 or p19 fragments of MSP-1. Preclinical studies in the non-human primate Aotus monkey model demonstrated that immunization with p42 formulated with Freund's adjuvant, Montanide, ISA720 or AS02A, can elicit partial protection against *P. falciparum* blood stage challenge with the homologous parasite strain [26]. Level of protection depended on the adjuvant used and correlated with the Enzyme-Linked Immunosorbent Assay (ELISA) titres and invasion inhibitory activity of antibodies elicited by the vaccine formulation [26].

Phase 1 and 2 trials in humans indicated that recombinant p42 formulated with AS02 adjuvant was immunogenic and elicited antibodies that recognized native MSP-1 [27, 28]. However, immunization of individuals residing in Africa with p42 formulated with AS02 did not protect against natural challenge in the field [29].

The lack of protection observed in these studies was most likely due to the inability of this vaccine formulation to elicit high-titre and functional, growth inhibitory antibodies against blood stage parasites [28]. Importantly, additive effects of inhibitory antibodies targeting all MSP-1 subunits, as well as some associated MSPs, have been shown to inhibit parasite growth in RBC cultures [30].

A recently completed Phase Ia clinical trial in Heidelberg, Germany, reported promising immunogenicity of a full-length recombinant MSP-1 “heterodimer” composed of two peptide chains corresponding to the N- and C-terminal fragments of MSP-1, namely p83/30 and p38/42, respectively [31]. Importantly, this complex seems to assemble similar to the native MSP-1 complex, raising the possibility of inducing broadly reactive antibodies by vaccination that will cross-react with the native, merozoite expressed MSP-1 resulting in enhanced anti-parasite immunity [32].

2.3 General treatment/ Administration

For this Phase Ib study, the same formulation tested in the previous Phase Ia study in Heidelberg, Germany will be used [31]. Sumaya Biotech GmbH & Co.KG will provide the MSP-1 malaria antigen and corresponding adjuvant (shipped from Access to Advanced Health Institute (AAHI), Seattle), while the control for the vaccination, a rabies vaccine (Verorab®), will be purchased locally in Tanzania. The vaccinations will be administered through intramuscular injection (i.m.) in the deltoid muscle.

The purified, cryo-preserved infectious *P. falciparum* sporozoites (strain NF54) for the Controlled Human Malaria Challenge (CHMI) will be provided by Sanaria Inc. and purchased by Sumaya Biotech GmbH & Co.KG. The sporozoites will be administered through direct venous inoculation (DVI) after thawing on the site.

2.4 Efficacy

As the previous Phase Ia trial was a first-in human study, no efficacy endpoints were defined, and therefore there are currently no data on the efficacy of the vaccine.

2.5 Scientific justification - Pharmacodynamics & Pharmacokinetics

In accordance with the *Guideline on clinical evaluation of vaccines* (EMEA/CHMP/VWP/164653/2005) [33], pharmacodynamics and pharmacokinetic studies are applicable if the vaccine contains novel adjuvants or excipients. This is not the case for this study.

However, immunogenicity studies characterising the immune response to the vaccine (as described below) essentially comprise pharmacodynamic studies for a vaccine.

2.6 Immunogenicity studies

Seroconversion and anti-MSP-1 IgG and IgM antibody titres

In the previous Phase Ia study, all 24 participants who received the vaccine seroconverted compared to the placebo (n=4) and the Glucopyranosyl Lipid Adjuvant-Stable Emulsion (GLA-SE) adjuvant-only groups (n=4), where an increase in MSP-1 specific antibody (IgG and IgM) titres was not observed.

The MSP-1 specific IgG and IgM antibody titres peaked around four weeks after the third immunization, exceeding the titres found in semi-immune individuals from malaria endemic areas. The fourth immunization administered 4 months after the 3rd vaccination boosted the declining MSP-1 antibody titres. Following the 4th immunization, specific IgG antibodies persisted for at least 6 months at levels seen in malaria semi-immune individuals. Long-lasting MSP-1 specific antibody titres were also observed for IgM.

There were no significant differences between the three dose levels (25 µg MSP-1, 50 µg MSP-1 or 150 µg MSP-1), regarding the IgG or IgM titres or their time courses. However, participants who received only three immunizations instead of four, generally had lower MSP-1 specific IgM and IgG levels 12 months after the first immunization irrespective of the dose.

Antibody-dependent respiratory burst (ADRB)

In the evaluation of the Phase Ia study, the ADRB assay was only assessed at baseline and four weeks post 3rd and 4th vaccination. The *in vitro* assay showed that the IMP could induce antibodies indicating its ability to stimulate immune effector cells and neutrophil granulocytes upon opsonization of merozoites. The arithmetic mean values of the amount of reactive oxygen species (ROS) for all three groups of vaccinated participants increased from baseline to four weeks post vaccination.

Growth inhibition assay (GIA)

In the previous Phase Ia study, no growth inhibitory activity could be shown in the GIA by analysing the purified IgG collected four weeks post 3rd vaccination from 14 SumayaVac-1 (SUM-101) immunised participants.

ELISpot investigations

In the completed Phase Ia study, enzyme-linked immunosorbent spot-forming cell assay (ELISpot) assays were performed at baseline and 3 and 6 months post 1st vaccination and prior to 4th vaccination.

To investigate MSP-1 specific CD4+ and CD8+ T-cell responses in paired assays, peripheral blood mononuclear cells (PBMCs) were exposed to full-length MSP-1 (as a recall antigen) in cultured interferon gamma (IFN-γ) ELISpot assay. Results from the selected participants showed significant increase in cultured ELISpot responses at 4 weeks post 3rd vaccination and remained stable in 5 out of 6 participants for more than 4 months. One participant had a decrease in PBMCs in recall stimulation at 6 months post 1st vaccination.

Those results suggest that as a next step, CHMI studies could help evaluate the efficacy of the vaccine candidate induced immunity against a homologous *P. falciparum* strain.

2.7 Safety and tolerability

In the previous Phase Ia clinical trial, the vaccination was generally well tolerated. There were no serious adverse events (SAEs), no dose-limiting toxicities, and no events resulting in permanent disability or premature withdrawal from the study. There was further no pattern of adverse events (AEs), suggestive of off-target effects. A total of 562 AEs occurred in the 32 participants with local injection site reactions exhibiting the highest frequency.

Laboratory values did not show any clinically relevant pattern of change except for an increase in C-reactive protein (CRP, a marker for systemic inflammation) in the first days after each vaccination. In all vaccination observation cycles, 93% of AEs were of mild nature and transient. Only two severe events occurred, which were assessed as being not related to the vaccination. One event was a hypertension grade III (according to the Common Terminology Criteria for Adverse Events 4.0 (CTCAE)) prior to vaccination, which resolved without treatment and was attributed to the stress of the anticipated vaccination. The other was a tendon rupture occurring 50 days after the last vaccination. Overall, tolerability was comparable in all dose groups (25 µg MSP-1, 50 µg MSP-1 or 150 µg MSP-1) and the GLA-SE group, and the vaccine did not show any dose-dependent adverse effects. In the post-trial follow-up, one participant showed a moderate event of a transient inflammatory disease, and one severe adverse event occurred, which was an unrelated accidental injury from sports. All other post trial contacts did not reveal any clinically relevant adverse events.

2.8 Toxicity

The MSP-1 protein is produced in *Escherichia coli (E.coli)* bacteria and supplied with >99% purity under good manufacturing practice (GMP) conditions and as stable substance. Examination in Sprague-Dawley rats at a dose >200 fold (based on body weight) of the intended highest human dose and >15 fold for *Aotus griseimembra* monkeys did not reveal any compound-related findings [30].

2.9 Justification for the dosage and dosage plan, and the length of treatment

In the Phase Ia clinical trial, antibody titres were independent of the immunization dose (25 µg, 50 µg and 150 µg). It is unclear whether the lower doses would be suitable in a clinical setting and in the proposed Phase Ib trial. Individuals living in malaria endemic areas (adults and infants) might require a higher dose given their lower responsiveness to vaccination compared with healthy malaria naïve adults [34]. Thus, three monthly inoculations (to be done on D0, D28 and D56) with the 150 µg dose are planned.

3 OBJECTIVES AND PURPOSE

3.1 Study rationale and objectives

3.1.1 Study rationale

The proposed clinical trial is the second administration of this antigen and adjuvant combination to humans [31]. The trial will enrol healthy adults pre-exposed to the malaria parasite and will be the first administration of the IMP to healthy participants with some pre-existing immunity. The goal of the study is to test the vaccine candidate early in the development path in the population living in malaria endemic areas. The vaccination part of this study will be performed in a randomised, double-blinded, controlled design to evaluate the safety, reactogenicity and immunogenicity of the candidate malaria vaccine, SumayaVac-1 (SUM-101) (MSP-1 with GLA-SE as adjuvant).

CHMI will be done in a subset of participants after the SumayaVac-1 (SUM-101) vaccinations, to assess the reduction in PMR in comparison to the rabies control vaccine (Verorab®).

3.1.2 Primary objectives

- To evaluate the safety and reactogenicity of SumayaVac-1 (SUM-101) in healthy adults of African origin, previously exposed to the parasite in comparison to the Verorab® control vaccine.

- To evaluate the immunogenicity of SumayaVac-1 (SUM-101) in healthy adults of African origin previously exposed to the parasite in comparison to the Verorab® control vaccine.

3.1.3 Secondary objective

- To evaluate the relationship of SumayaVac-1 (SUM-101) vaccine-induced antibody levels, *in vitro* effector functions and isotype distribution with asexual blood stage parasite growth rates after homologous CHMI in participants having received SumayaVac-1 (SUM-101) versus rabies control (Verorab®) vaccine.

3.1.4 Exploratory objectives

- Comparison of SumayaVac-1 (SUM-101) induced immunoglobulin isotype distribution and duration between malaria pre-exposed and malaria naïve participants from the previous Phase Ia study in Heidelberg.
- Fine scale epitope mapping of SumayaVac-1 (SUM-101) specific antibodies, including specific CD4+ and CD8+ T-cell epitopes using peptide arrays, to investigate the association between cellular immune response with humoral seroconversion and investigate the potential association with protection after homologous CHMI.
- Comparison of SumayaVac-1 (SUM-101) induced cellular immunity between malaria pre-exposed and malaria naïve participants from the previous Phase Ia study in Heidelberg.
- Investigation of the B- and T-Cell repertoire before and after SumayaVac-1 (SUM-101) vaccination, as well as after homologous CHMI.
- Integrated transcriptome and immunoglobulin gene repertoire analyses of MSP-1 specific B-cells using single-cell technologies.
- Glycosylation patterns of MSP-1 specific functional antibodies.
- Investigate off-target IgG and IgM repertoire after SumayaVac-1 (SUM-101) vaccination using immunoproteomics and their association with protection after homologous CHMI.
- Investigate the structure of MSP-1 protein bound to functional antibodies by cryo-electron tomography to map conformational epitopes.
- To evaluate mechanisms of malaria specific antibody diversity generation, post-translational modification of antibodies and B- and T-cell memory generation and maintenance.
- To establish human and parasite transcriptome in peripheral blood during early stage asexual blood stage parasitemia in participants having received SumayaVac-1 (SUM-101) versus rabies control (Verorab®) vaccine after CHMI.
- To study the quality and quantity of peripheral blood γδ T-cell responses in participants having received SumayaVac-1 (SUM-101) versus rabies control (Verorab®) vaccine with or without CHMI (all 40 participants).
- To calculate liver to blood inoculum size of the CHMI-induced parasites in participants having received SumayaVac-1 (SUM-101) versus rabies control (Verorab®) vaccine (25 participants that undergo CHMI).

3.2 Scientific justification and rationale

3.2.1 Scientific justification - study population

This study includes healthy men and healthy non-pregnant and non-breastfeeding women between 18 and 45 years old residing in Tanzania and of African origin.

As this is a Phase Ib clinical trial, healthy participants residing in a malaria endemic country are essential to evaluate the safety and reactogenicity in the target population.

Healthy participants of both genders will be enrolled to generate data on both sexes. Women of childbearing potential (WOCBP) and men will be required to use a highly effective form of contraception (see section 11.3.2).

3.2.2 Scientific justification - vaccinations

The rationale for 3 immunisations results from preclinical studies in mice and rabbits indicating that 3 immunisations led to higher titres than 2 immunisations. Further, pyrogenicity studies conducted in rabbits with a dose equivalent to four times the human dose regimen did not highlight any safety issue [35].

The 0, 4 and 8 weeks immunisation schedule for the three vaccinations in the vaccination part of the study can later be easily integrated into the immunisation programme for childhood vaccines as recommended by the WHO.

Preferred site for routine vaccinations is by i.m. administration. Further, injection of small volumes like 0.5 ml into the deltoid muscle is a very frequently practiced vaccination procedure. After technically correct injection, the local AEs at injection site are generally mild and transient [36].

3.2.3 Scientific justification - CHMI

CHMI by *P. falciparum* sporozoites (PfSPZ) challenge is a procedure in which recipients can be purposefully infected with malaria by introduction of sporozoites intravenously [37]. Several trials of CHMI by PfSPZ challenge in humans -also successfully conducted in Bagamoyo- have been completed [37-42] and data from these trials indicate that PfSPZ challenge is safe and efficacious when administered by intra-dermal, intra-muscular and direct venous inoculation routes. Malaria-naïve subjects undergoing CHMI develop clinical malaria while, in malaria pre-exposed subjects, symptoms of parasitemia are generally absent or mild, due to naturally acquired immunity [37]. CHMI studies to assess malaria vaccines in endemic African countries are now being commonly performed and enable the evaluation of a vaccine candidate in a small number of individuals in a short period of time.

After CHMI, the participant will return to the site before the first merozoites exit the liver (after about 6-7 days) and will be treated as soon as (i) the quantitative polymerase chain reaction (qPCR) threshold of >500 parasites/µl is reached, or (ii) participant presents malaria symptoms with a positive TBS/ RDT, or (iii) stays negative for malaria but completes 28 days after CHMI. Hence, high levels of parasites and prolonged parasitemia are unlikely to occur. The participant will be confined and therefore will be in very close contact to the study staff in the time the parasitemia develops. The participant will remain confined at the site until the end of malaria treatment and discharged after a final check and confirmation by two consecutive negative malaria qPCR results. With these measures, the risk of developing severe malaria can be minimised.

3.2.4 Scientific justification - Dose

150 µg MSP-1 protein was the highest dose used in the preceding Phase Ia trial with the same IMP in healthy, malaria-naïve participants and was shown to have good safety and tolerability [31]. This dose was also considered as the no-observed-adverse-effect-level (NOAEL) in toxicology studies in rats, which gives a safety factor of >200 considering the body weight of the study population. The dose of 150 µg MSP-1 protein is used in the current proposed study where the participants are pre-exposed to malaria and helminth infections resulting in a likely reduced immune responsiveness.

3.2.5 Scientific justification for deviation from Verorab® dosing schema

The dosing scheme proposed for Verorab® is generally the following: Three doses of 0.5 ml are administered at D0, D7 and D28 for vaccination. In order to maintain double-blinding, the dosing scheme for Verorab® will be the same as for the IMP (D0, D28 and D56).

The rabies vaccine, Verorab®, has been chosen as the comparator because participants will benefit from receiving the rabies vaccine as rabid animals reside in the study area and three doses of the rabies vaccine induces protective antibody levels. When the rabies vaccine is administered according to the recommended vaccination schedule (D0, D7, D21), nearly 100% of subjects attain a protective titre. High antibody titres have also been demonstrated with off-label immunization with the rabies vaccines. Among participants in England, Germany, France and Belgium who received two vaccinations one month apart, nearly 100% of the participants developed specific antibodies and the geometric mean titre for the group was 10 IU/ml [43, 44] well above the protective antibody

titres >0.5 IU/ml. Our proposed vaccination schedule of D0, D28 and D56 is therefore expected to be successful in conferring protective immunity against rabies among the control participants. However, all participants will be advised to seek medical attention immediately if bitten or scratched by a potentially rabid animal.

3.2.6 Scientific justification- retrospective helminth infection analysis

The potential impact of prevalent worm infections on vaccine immunogenicity and efficacy has gained increasing attention. Evidence has been generated over the past years that active helminth infections are associated with lower responses to a wide range of vaccination approaches including tetanus toxoid (subunit vaccine), Bacille Calmette-Guerin (live attenuated vaccine) and cholera (live attenuated vaccine) [45]. Vaccination of helminth-infested participants resulted in lower vaccine-induced T-cell proliferation and Type 1 T helper (TH1) cytokine production but increased immune-regulatory cytokine production like interleukin 10 (IL-10) [46-48]. Hence, a retrospective analysis on stool and urine samples collected at baseline (D0 before 1st vaccination), at D28 (before 2nd vaccination), D56 (before 3rd vaccination) and D84 (before CHMI, if applicable) will be performed to correlate antibody responses to any potential active helminth infection at that time.

4 STUDY DESIGN

This is a randomised, controlled, double-blind, parallel group, single center Phase Ib trial to assess safety, reactogenicity, immunogenicity and parasite growth rates after homologous CHMI of SumayaVac-1 (SUM-101) in healthy, malaria pre-exposed participants of African origin aged 18-45 years.

The study is divided in two parts (**Figure 1**):

Vaccinations (Part 1)

In total, 40 participants will be enrolled (male and female). 20 participants will be randomised to receive three monthly inoculations (on D0, D28 and D56) with the IMP, SumayaVac-1 (SUM-101), and 20 participants will be randomised to receive the registered rabies vaccine (Verorab®) as controls. For operational reasons the participants in the vaccinations part will be divided in two groups of 20 participants:

- **Group 1** will have a sentinel subgroup (2 SumayaVac-1 (SUM-101) & 1 Verorab® rabies vaccine) where the 48 hours post vaccination safety data will be analysed (see chapter 4.5.2) before the remaining 17 participants (8 SumayaVac-1 (SUM-101) & 9 rabies vaccine) of group 1 receive their first vaccination.
- **Group 2** will be composed of 20 participants (10 SumayaVac-1 (SUM-101) & 10 Verorab® rabies vaccine). All visits in group 2 will be shifted by 3 weeks compared to group 1 to create minimal overlap of study related activities.

After each vaccination (done on D0, D28 and D56), the participant will stay for 2 hours at the facility before being discharged. The participant will be called by phone or visited at home (if needed) daily until 6 days post vaccination for follow-up.

For all the participants, on-site vaccination follow-up visits will occur at 7, 14 and 28 days after each vaccination (done on D0, D28 and D56).

CHMI (Part 2)

In total, 25 participants (15 SumayaVac-1 (SUM-101) & 10 Verorab® vaccinated), will be selected randomly by an independent statistician from the list of participants who have received all three vaccinations according to the protocol and will undergo CHMI with attenuated sporozoites 4 weeks after the 3rd vaccination.

For operational reasons, the participants will remain in two separate groups as defined in the vaccination part (group 1: total 12, 7 SumayaVac-1 (SUM-101) & 5 Verorab® vaccinated; group 2: total 13, 8 SumayaVac-1 (SUM-101) & 5 Verorab® vaccinated).

In case a participant selected for CHMI does not take part in CHMI for any reason, this participant will be replaced in a blinded manner, if possible by another participant having received the same vaccine (SumayaVac-1 (SUM-101) or Verorab®) in order to keep the overall number of participants for the CHMI part of 15 participants with SumayaVac-1 (SUM-101) and 10 participants with Verorab®.

The participants must be parasitemia free prior to receipt of CHMI and hence they will be tested by qPCR on D63 and D84 (prior to CHMI) visits. Any participant found to be positive will be treated with anti-malarial treatment (artemether/ lumefantrine (ALU)) for 3 days. After treatment with ALU, daily parasitological sampling will continue until negative results are obtained for two consecutive daily qPCRs. If possible, as the first and preferred approach, this participant will be dropped from the CHMI part of the study and replaced by another randomly selected participant from the overall 40 participants from the SumayaVac-1 (SUM-101) or Verorab® arm, in a blinded manner. A participant from group 1 that tests positive for malaria before CHMI can be replaced by a participant from group 1 (including sentinels) or group 2. However, a participant from group 2 that tests positive for malaria before CHMI can only be replaced by a participant from group 2, for logistical reasons.

As the second approach, in the case that a replacement is not possible, and as the use of ALU may adversely affect vaccine take or CHMI effects due its action on liver stage parasites, CHMI inoculation will be postponed until at least 3 weeks after the day of completion of malaria treatment.

Only after pre-CHMI procedure results have been reviewed and the study clinician determines that there are no temporary or permanent reasons to put the CHMI on-hold (see section 4.5), the participant can receive the CHMI challenge. Delaying CHMI will only be possible for participants from group 1, for logistical reasons.

After the CHMI, the participant will stay at the facility for 2 hours before being discharged. The participant will be called by phone or visited at home (if needed) daily until Day 4 post CHMI for follow-up. 5 days post CHMI, participants will return to the site and be admitted to the ward for closer observation during the period when the first wave of asexual blood stage parasites are released from liver stage schizonts and can be detected.

The participant will remain in confinement at the site until he/she is considered malaria positive (between 5-28 days post CHMI) (see section 7.1.13). Once the participant is tested positive for asexual blood stage parasitemia, the blood sample will also be tested by genotyping for parasite differentiation to distinguish between the challenge strain and a potential, naturally acquired *P. falciparum* infection.

All participants will then receive anti-malarial treatment with ALU for 3 days plus a single dose of primaquine and be discharged after being confirmed by qPCR to be malaria negative (see section 7.1.14).

CHMI follow-up visits will take place at 3 months (D168) and 6 months (D252) post CHMI.

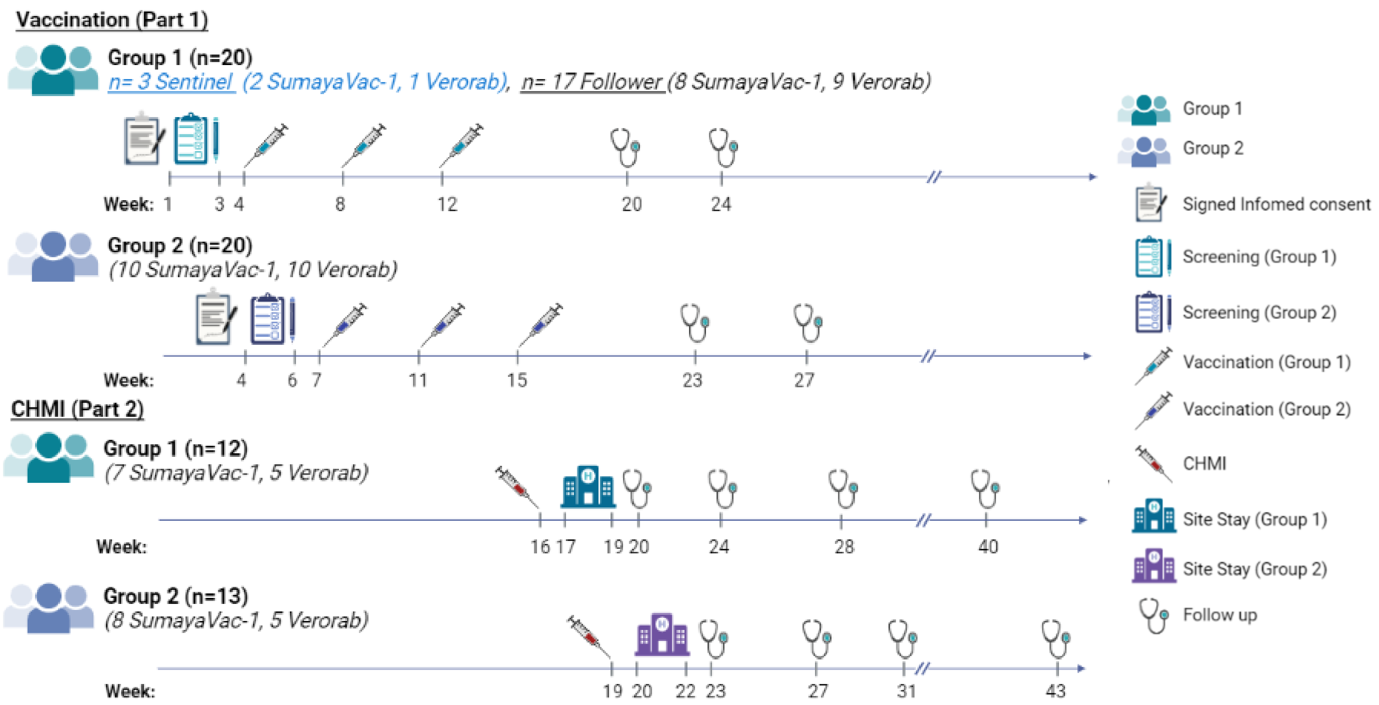

FIGURE 1 – STUDY DESIGN.
Time is indicated in calendar weeks from start of the study (not from randomisation for individual participants).

4.1 Primary and secondary endpoints

4.1.1 Primary endpoints

The following endpoints will be assessed in all participants to evaluate the safety and reactogenicity of SumayaVac-1 (SUM-101):

- Local and systemic solicited adverse events (AEs) at least possibly related to IMP recorded after each vaccination (done on D0, D28 and D56) up to 7 days later.
- Local and systemic unsolicited reactogenicity recorded after each vaccination (done on D0, D28 and D56) up to 28 days later.
- Any SAE occurring after the first vaccination until the participant's last visit.
- Changes in laboratory safety parameters between baseline (D0 before 1st vaccination) to 28 days after each of the vaccinations.
- Changes in laboratory safety parameters between values just prior to each vaccination (on D0, D28 and D56) and values 28 days after that vaccination.

The following endpoints will be measured in all participants at D0 pre-vaccination, D28 (W4), D56 (W8), D84 (W12), D112 (W16), D140 (W20), and additionally at D168 (W24) and D252 (W36) for participants that undergo homologous CHMI, to evaluate the humoral immunogenicity:

- Longevity of antibody responses to SumayaVac-1 (SUM-101) by ELISA.
- Fold change of antibody responses to SumayaVac-1 (SUM-101) in comparison to baseline (D0 pre-vaccination).

4.1.2 Secondary endpoints

The following endpoints will be measured in the sera or blood of all participants, at D0 pre-vaccination, D28 (W4), D56 (W8), D84 (W12), D112 (W16), D140 (W20), and additionally at D168 (W24) and D252 (W36) for participants that undergo homologous CHMI:

- Evaluation of the opsonic phagocytosis activity.
- Evaluation of complement fixation, activation and/or membrane attack complex (MAC) formation.
- Evaluation of antibody-dependent respiratory burst (ADRB) activity.
- Evaluation of antibody-dependent cellular cytotoxicity (ADCC-NK cells) activity.
- Evaluation of immune-mediated growth inhibition activity on a panel of *P. falciparum* lines.
- Development of *P. falciparum* parasitemia (pre-patent period, PMR and percentage of parasite negative participants in SumayaVac-1 (SUM-101) versus Verorab arm) by TBS following CHMI.
- Development of *P. falciparum* parasitemia (pre-patent period, PMR and percentage of parasite negative participants in SumayaVac-1 (SUM-101) versus Verorab arm) by qPCR following CHMI.

The following endpoints will be measured in all participants:

- Cellular immune responses to SumayaVac-1 (SUM-101) by (i) CD4+ and CD8+ ELISpot assays, (ii) SumayaVac-1 (SUM-101) specific cells characterised by flow cytometry-based immunophenotyping using intracellular cytokine staining (ICS), (iii) functional gene expression analysis, and/or other assays to be defined; at D0 pre-vac and D56 (W8), D84 (W12), D112 (W16) and D140 (W20).

The following endpoint will be assessed among SumayaVac-1 (SUM-101) participants only:

- Comparison of MSP-1 IgG antibody concentrations by ELISA between malaria pre-exposed participants in the current study and malaria naïve participants from the previous Phase Ia study in Heidelberg.

4.1.3 Exploratory endpoints

- Comparison of SumayaVac-1 (SUM-101) induced immunoglobulin isotype distribution and duration between malaria pre-exposed and malaria naïve participants from the previous Phase Ia study in Heidelberg.
- Fine scale epitope mapping of SumayaVac-1 (SUM-101) specific antibodies, including specific CD4+ and CD8+ T-cell epitopes using peptide arrays, to investigate the association between cellular immune response with humoral seroconversion and investigate the potential association with protection after homologous CHMI.
- Comparison of SumayaVac-1 (SUM-101) induced cellular immunity between malaria pre-exposed and malaria naïve participants from the previous Phase Ia study in Heidelberg.
- Investigation of the B- and T-Cell repertoire before and after SumayaVac-1 (SUM-101) vaccination, as well as after homologous CHMI.
- Integrated transcriptome and immunoglobulin gene repertoire analyses of MSP-1 specific B-cells using single-cell technologies.
- Glycosylation patterns of MSP-1 specific functional antibodies.
- Investigate off-target IgG and IgM repertoire after SumayaVac-1 (SUM-101) vaccination using immunoproteomics and their association with protection after homologous CHMI.
- Investigate the structure of MSP-1 protein bound to functional antibodies by cryo-electron tomography to map conformational epitopes.
- Measurement of asexual blood stage prepatent period in participants having received SumayaVac-1 (SUM-101) versus Verorab® rabies vaccine after CHMI.

- *Ex vivo* assessment of asexual blood stage parasite transcriptome during CHMI in participants having received SumayaVac-1 (SUM-101) versus Verorab® rabies control vaccine.
- *Ex vivo* assessment of changes in human peripheral blood transcriptome before, during and after CHMI in participants having received SumayaVac-1 (SUM-101) versus Verorab® rabies vaccine.
- Description of ɣδ T-cell receptor repertoire, transcriptome, functional activity and phenotypes before, during and after homologous CHMI in participants having received SumayaVac-1 (SUM-101) versus Verorab® rabies vaccine.
- Investigate the impact of the presence of intestinal helminth infections on vaccine-induced humoral immune response by comparing the quality and quantity of SumayaVac-1 (SUM-101) specific antibody isotypes between helminth infected and non-infected participants at baseline.
- Investigate the impact of the presence of intestinal helminth infections on SumayaVac-1 (SUM-101) vaccine-induced cellular immune responses by comparing the quality and quantity of SumayaVac-1 (SUM-101) specific cytokine production and ICS results between helminth infected and non-infected participants at baseline.

4.2 Measures to minimize bias

4.2.1 Randomization

In the vaccination part of the study, the participants will be randomised either to the SumayaVac-1 (SUM-101) vaccine or the Verorab® rabies vaccine which serves as a comparator.

For both groups the overall ratio will be 1:1. Three sentinel participants will be vaccinated first in Group 1.

The randomisation scheme is as follows:

- Group 1 (n=20): 3 sentinels (2 SumayaVac-1 (SUM-101), 1 Verorab®), followed by 17 participants (8 SumayaVac-1 (SUM-101), 9 Verorab®)
- Group 2 (n=20): 20 participants (10 SumayaVac-1 (SUM-101), 10 Verorab®)

An independent statistician (not part of the analysis team) at Swiss TPH will generate the allocation sequence using appropriate software. The independent statistician will provide the randomisation list in a secure manner to the site's pharmacist and will also maintain the randomisation list on a secure server with restricted access.

The pharmacist will allocate and prepare the vaccine for each participant according to the randomisation list (allocation of treatment to the next participant ID according to the sequential list) and label the prepared dose(s) with the participant ID. The randomisation list will be kept locked in the pharmacy and remain inaccessible to any other site staff. At the 2nd and 3rd vaccination, the pharmacist will refer to the randomisation list to confirm the vaccine allocated (SumayaVac-1 (SUM-101) or Verorab®) to each participant.

For the CHMI, a subset of 25 participants will be randomly selected by the independent statistician (see section 4). Participants belonging to the sentinel group will also be considered for CHMI.

- Group 1: a subset of 12 participants (7 randomised to SumayaVac-1 (SUM-101) & 5 randomised to Verorab® control)
- Group 2: a subset of 13 participants (8 randomised to SumayaVac-1 (SUM-101) & 5 randomised to Verorab® control)

The above subsets allow maintenance of blinding to randomised allocation.

4.2.2 Double blinding of vaccination and unblinding

This trial will be conducted in a double-blinded manner. Namely, the participants, site staff, sponsor staff, the study monitor(s) and the trial statistician will be blinded to the treatment allocation. The independent statistician and the pharmacist are not blind throughout the study.

The pharmacist will be supplied with opaque, sealed envelopes (prepared by an independent person), each labelled with the corresponding randomisation number on the outside and containing inside the allocated treatment. These envelopes will be kept in the pharmacy with restricted access and handed out to clinical staff in case of emergency.

If necessary, a separate unblinded monitor may be used to perform drug monitoring and to check accountability during the conduct of the study. Procedures should be followed to avoid unblinding of other study personnel.

If required and indicated, advisor(s) independent from the study team may be appointed to review unblinded data to assist decision-making during Safety Monitoring Meetings.

If unblinding is required earlier in the interest of a participant's safety, the Investigator will discuss the matter with the Sponsor before opening the individual code-break envelope for that participant. In a medical emergency, the Principal Investigator (PI) or delegate may open the individual code-break envelope for that participant without prior consultation with the Sponsor. In that event, the PI or delegate will notify the Sponsor as soon as possible that the randomisation code has been broken for the respective participant.

If the code for a participant is broken, the reason for doing so will have to be fully documented and entered in source documents and the electronic Case Report Form (eCRF).

At the end of the study upon unblinding, the participants will be informed about the vaccination they received during the study. The participants who received SumayaVac-1 (SUM-101), will be offered a rabies vaccination.

4.2.3 Masking of syringe

As the SumayaVac-1 (SUM-101) vaccine and the rabies control vaccine may not have the same appearance, in order to conduct the trial in double-blind manner as described in 4.2.2, the syringe (in both cases 0.5 ml) will be prepared and masked by the unblinded pharmacist before handing over to the nurse for administration. Details are described in the corresponding working instruction/standard operating procedure (SOP).

4.3 Study duration and duration of participant's participation

The total study duration will be approximately 40 weeks including the screening period.

The study duration for each participant involved in the vaccinations (Part 1) and the CHMI (Part 2) (n=25) is 36 weeks.

For participants not undergoing CHMI (n=15) the study duration will be 20 weeks.

4.3.1 Schedule of events

Study Visit (V)	V1	V2	V3		V4-V9	V10	V11	V12		V13-V18	V19	V20	V21		V22-V27	V28	V29	V30		V31-V34	V35			V36	V37	V38	V39	UV
Day (D)/ Week (W)	-D35 to -D1		D0		D1-6	D7	D14	D28 (W4)		D29-34	D35	D42	D56 (W8)		D57-62	D63	D70	D84 (W12)		D85-88	D89-115			D112 (W16)	D140 (W20)	D168 (W24)	D252 (W36)	Anytime
	Screening		1st Vaccination						2nd Vaccination					3rd Vaccination					CHMI					Follow Up				
	-35 to -1d		Pre-Vac -1d to 0d		1-6d	7d	14d	28d	0d	1-6d	7d	14d	28d	0d	1-6d	7d	14d	28d	0d	1-4d	5-28d	5-28d	5-28d	Vac1 + 4m	Vac1 + 5m	CHMI + 3m	CHMI + 6m	
Window	-35 to -1d		-1d	0d			± 2d	± 3d	± 3d			± 2d	± 3d	± 3d			± 2d	± 3d	± 3d				± 12h	± 7d	± 7d	± 7d	± 7d	
**Clinical Procedures**																												
Clinic Visit	●	●	●	●		●	●	●	●		●	●	●	●		●	●	●	●		●	●	●	●	●	●	●	●
Phone call/Home Visit [a]					●					●					●					●								
Informed consent	●																											
Vital Signs [b]	●		●	●●●●		●	●	●	●●●●		●	●	●	●●●●		●	●	●	●●●●		●●	●●	●	●	●	●	●	●
Medical history	●	●	●																									
Prior/Concomitant medication	●	●	●	●	●	●	●	●	●	●	●	●	●	●	●	●	●	●	●	●	●●	●●	●	●	●	●	●	●
Contraception use	●	●	●			●	●	●			●	●	●			●	●	●					●	●	●	●		
Physical exam [c]	●		●	●		●	●	●	●		●	●	●	●		●	●	●	●		●●	●●	●	●	●	●	●	●
Bed nets use [d]		●	●			●	●	●			●	●	●			●	●	●					●	●	●	●	●	
ECG	●																											
Eligibility	●	●	●					●					●					●										
Randomisation/CHMI selection			●															●										
Vaccination				●					●					●														
CHMI																			●									
Clinic Stay																					●	●						
AE – *Solicited*				●●●●	●	●			●●●●	●	●			●●●●	●	●			●●●●	●	●●							
AE – *Unsolicited*				●●●●	●	●	●	●	●●●●	●	●	●	●	●●●●	●	●	●	●	●●●●	●	●●	●●	●	●	●	●	●	●
**Lab Evaluations**																												
Pregnancy test (1 ml)	●		●					●					●					●						●	●	●	●	Based on need
Urinalysis	●		(●)[e]					●					●					●					●					
Haematology (1 ml)	●		(●)[e]			●	●	●			●	●	●			●	●	●			(●)[f]	(●)[f]	(●)[f]					
Biochemistry (2 ml)	●		(●)[e]			●	●	●			●	●	●			●	●	●			(●)[f]	(●)[f]	(●)[f]	●	●	●	●	
Serology (1 ml)	●																											
Urine and Stool			●					●					●					●										
Parasitology (2 ml) – *qPCR*	●		●					●					●			●		●			●●	●●	●	●	●	●	●	
Parasitology (2 ml) – *TBS*	●		●					●					●			●		●			●	●	●	●	●	●	●	
Parasitology (2 ml) – *RDT*																					(●●●●)[g]							
Immunology – *Humoral (ml)*			27					27					27					27						27	27	27	27	
Immunology – *Cellular (ml)*			25					25					25					25						25	25			
Exploratory (ml)			25					25					25			25		25				10		25				
Genotyping (ml)																						4						
Blood (ml)/ per visit	7		83			3	3	83			3	3	83			30	3	83			(---------61-89---------)[h]			82	57	32	32	
Total blood (ml) in study	~ 523-676 ml																											

a. Daily phone calls or home visit if necessary

b. Complete at Screening 1 and focused at all other visits

c. Complete at Screening 1 and focused at all other visits

d. Supplied at Screening visit 2

e. Repeated only if screening was done more than 7 days before

f. During CHMI, hematology and biochemistry are done on: (i) D89 (day of admission), (ii) D96 (7days after admission), (iii) day of testing positive for malaria or if negative, D112 (28 days after CHMI), (iv) on day of discharge. On day of admission, 2 ml of blood will be taken to assess intentional intake of anti-malarials

g. RDT will be done up to 4 times/ day if a participant has malaria symptoms but is qPCR/ TBS negative

h. If malaria negative till D112 then blood volume collected during CHMI stay is ~83 ml. If malaria positive starting from anyday between 14 days post CHMI to 28 days post CHMI, blood volume collected is betwen 61-89 ml

Legend: Informed consent; Screening; Vaccination; Phone follow up; Site follow up; CHMI; Site stay; Malaria positivity; Discharge

4.4 Early termination of the study

Premature termination of this study may occur due to a Regulatory Authority (RA) decision, change in opinion of the Independent Ethics Committee (IEC) or Institutional Review Board (IRB), or drug safety problems.

Both the Sponsor and the Investigator reserve the right to terminate the study at any time before enrolment of the intended number of participants, but intend to exercise this right only for valid scientific or administrative reasons. Should early termination be necessary, both parties will arrange the procedures on an individual study basis after review and consultation. In terminating the study, the Sponsor and the Investigator will assure that adequate consideration is given to the protection of the participants' interests.

Reasons for early termination by the Sponsor(s) may include but are not be limited to:

- Safety issues with the study drug (see stopping rules in section 4.5).
- Low enrolment rate.
- Protocol violations.
- Inaccurate or incomplete data.
- Unsafe or unethical practices.
- Following the recommendation of the Safety Monitoring Committee (SMC), RA or the IEC.
- Unforeseen events such as an endemic/ pandemic.

Reasons for early termination by the Investigator may include but are not be limited to:

- Ethical concerns.
- Lack of eligible healthy participants.
- When the safety of the participants is doubtful or at risk.
- Alterations in accepted clinical practice that make the continuation of a clinical trial unwise.
- Early evidence of harm from the experimental intervention.

In the event that a study is terminated early, either by the Sponsor or by the Investigator, the Investigator must:

- Complete all documentation and eCRFs to the fullest extent possible.
- Destroy or return all test articles (IMP including adjuvant, and sporozoites) according to the Sponsor's instructions and local regulations.
- Return all related study materials to the Sponsor.
- Answer all questions from the Sponsors or their representatives related to participant data collected before the termination of the study.
- Ensure that participants enrolled in the study who had not yet reached 28 days post last vaccination or CHMI follow-up visit are followed up with the necessary medical care.
- Provide in writing the reasons for the decision to RA, IEC, IRB and the Sponsor if needed.

4.5 Stopping rules

4.5.1 Study pause at any time point

In case one of the following stopping rule is met at any time point in the study, the study will be temporarily interrupted (see section 5.5.2), and the SMC will do an ad-hoc assessment and conduct a thorough review before a decision is taken to continue or terminate the study.

- Any SAE possibly, probably or definitely related to the SumayaVac-1 (SUM-101) vaccination or CHMI.
- Any cardiac event that does not meet the criteria for an SAE.
- On the advice of local safety monitor, investigator, sponsor, SMC, Regulatory Authority and Ethics Committee.

4.5.2 Study pause at defined time-points

There are stopping rules (**No-Go criteria**) defined at three time-points (see below) in the course of this study. If one stopping rule (No-Go criterion) is met, the PI will be asked to break the blind (see section 4.2.2) for the concerned participant(s). The No-Go criteria are only applicable if the concerned participant(s) received SumayaVac-1 (SUM-101).

The SMC will check at defined time-points (see below) that none of the No-Go criteria are met and document the recommendation as described in the SMC charter.

No-Go criteria before vaccination of followers in group 1

If one of the following criteria is applicable to the safety data of 48 hours after 1st vaccination of the sentinel sub-group (n=3), the study will be interrupted and further investigations are needed before any further vaccination is administered:

- Any SAE possibly, probably or definitely related to the SumayaVac-1 (SUM-101) vaccination.
- Any Grade 3 or 4 AE possibly, probably or definitely related to the vaccination within 48 hours after the 1st vaccination persisting at Grade 3 (or higher) for >24 hours.

No-Go criteria before 1st vaccination of group 2 or 2nd vaccination of group 1

If one of the following criteria is applicable to the group 1 safety data at day 14, the study will be interrupted after the 1st vaccination of group 1 (n=20) and further investigations will be needed before group 2 can receive the 1st vaccination or group 1 can further proceed to the 2nd and 3rd vaccinations.

- Any SAE possibly, probably or definitely related to the SumayaVac-1 (SUM-101) vaccination.
- >4 participants (>20% of group 1) experience the same grade 3 or 4 AE possibly, probably or definitely related to the vaccination beginning within 48 hours after the 1st vaccination and persisting at grade 3 (or higher) for >48 hours.

No-Go criteria after all vaccinations and CHMI of group 1 and before CHMI of group 2

- Any SAE possibly, probably or definitely related to the vaccination.
- Any SAE possibly, probably or definitely related to the CHMI.
- >4 participants (>20% of group 1) experience the same grade 3 or 4 AE possibly, probably or definitely related to the CHMI beginning within 48 hours after the CHMI and persisting at grade 3 (or higher) for >48 hours.

4.5.3 Study pause for individual participants before CHMI

- Participants who test positive for malaria at D63 (day 7 visit after the 3rd vaccination) or D84 (before CHMI) (also see section 4).
- Participants who have contraindication to CHMI should not undergo CHMI.

4.6 Temporary contraindications for continuing vaccination/ CHMI

- The participant is ill on the day of vaccination/ CHMI (the study products may be administered to persons with a minor illness based on the investigator's clinical judgment).
- The participant has abnormal laboratory parameters that have been determined to be clinically significant.
- The participant has malaria or has taken or is taking medication for malaria or for another acute illness that may affect the vaccine/ CHMI.
- On request of the participant.

5 SELECTION OF THE STUDY PARTICIPANTS

5.1 Study setting

The study will be conducted at the Bagamoyo Clinical Trial Facility (BCTF) in Tanzania where screening, enrolment and follow-up of the healthy participants will be done. BCTF is part of the Bagamoyo Research Trial Center (BRTC) and therefore has access to the institutional research clinical laboratory with adequate facilities for the conduct of safety and several other assessments.

5.2 Recruitment

After approval has been received by relevant Ethics Committees to work in the community, local authorities will be informed about the study in order to receive permission to approach the community. Adult residents of Bagamoyo town and surrounding areas of Bagamoyo district will be recruited using a rolling recruitment, screening and enrolment process. In addition, healthy participants from the site's participant database may be approached and invited if they meet the study profile and are interested. This process will continue until all study participants have been enrolled. The recruitment strategies are based on the experience from previous trials conducted by the clinical team in BCTF aiming to achieve adequate participant enrolment to reach target sample size. The estimated recruitment period is 3 months. Study advertisement (approved by IEC) will be used to invite targeted and potential community members to attend sensitization meetings which will be conducted at designated community areas. Locally appropriate methods of advertisement will be used, such as flyers, announcements via speakers or local radio stations. In addition to local community members, these sensitization meetings may also involve ward leaders, community health workers and key opinion leaders in the community. To ensure adequate delivery of the information, meetings will primarily be conducted in Swahili.

At the community-based sensitization meetings, Investigators will explain the current burden of malaria and the need for an adequate malaria vaccination will be discussed in a culturally appropriate manner. Investigators will also explain the concept of the clinical trial and the community members will be made aware of the known and potential risks and benefits of the investigational product to be used in this trial. The risks related to the CHMI will also be explained.

The outline of the proposed trial including the rationale, aims and study procedures such as consenting, screening process and randomisation will be shared. It will also be made clear that the study will require screening for malaria and other chronic conditions such as Human Immunodeficiency Virus (HIV) and hepatitis. Further, it will be made clear that the screening processes will include a range of criteria to be checked covering clinical status as well as the ability to comply with the protocol and ethical requirements. Moreover, it will be explained that exclusion from participating in the trial does not mean the potential participant is unhealthy, nor will their provision for clinical care, if relevant, be affected in any way. It will be made clear that the confidentiality of all this information will be protected.

Opportunity will be provided for the community members to ask questions and receive responses from the Investigators. Potentially interested participants will be asked to register their names at the end of the meetings with the study staff who will complete preliminary registration forms for all those interested in a possible participation. These registries will be used by the study team to complete individual trial participation checklists and generate list of potential participants who will be invited to the clinic based sensitization meeting at BCTF. All data will be kept confidential at all times, by the Investigator and relevant clinicians.

During the clinic-based sensitization meetings, Investigators will provide further details via the participant information sheet on the mechanism of the study product, risks, benefits and procedures such as blood sampling, in-patient stay, follow-up visits and the need for efficient contraception. Study team members will discuss the existing understanding of the participants with regards to the study and will share more detailed information and clarify any questions in Swahili.

At the same occasion they will confirm that the participants are able to read and write (to ensure adequate informed consent for participants). Those interested and considered as potential participants for screening will be provided with the participant information sheet to read at home and

for opportunity to discuss with their family members. Potential candidates will be contacted and invited to a screening visit at the BCTF where they will undergo the individual informed consent process prior to any study screening procedures.

To ensure that enrolment goals are met, this recruitment plan allows for longitudinal screening of the participants. In addition, longitudinal compliance with study visits and procedures will be encouraged through the provision of a study calendar to participants, using multiple contact methods (phone calls, home visits by community mobilizers, having names and phone numbers of close contacts), and free transportation to the BCTF if needed.

5.3 Inclusion criteria

1. Written informed consent obtained before any study procedure.
2. Literate participants aged 18-45 years of African origin.
3. Female and male participant practicing contraception from 4 weeks before 1st immunization and up to 12 weeks after the last immunization or CHMI.
4. Available to participate in follow-up for the duration of study.
5. Contactable by phone during the whole study period.
6. At least two years residence in the Bagamoyo district or nearby districts in Coastal and Dar-es-Salaam regions and planning to reside there for at least 9 more months.
7. Agreement to provide personal contact information and contact information of another household member or close friend.
8. Female participants must be willing to avoid pregnancy if selected for participation in the trial and to undergo multiple serum pregnancy testing.
9. Confirmation of understanding of design, procedures, risk and benefits of the study in a test with maximum of two attempts.
10. General good health based on assessment of medical history and clinical examination.

5.4 Exclusion criteria

1. Previous participation in any malaria vaccine trial in the last 3 years.
2. Participation in any other clinical trial involving investigational medicinal products within 30 days prior to the screening assessment.
3. Previous history of drug or alcohol abuse interfering with normal social function within one year prior to enrolment.
4. Previous vaccination with a rabies vaccine.
5. Intake of chronic medication, especially immunosuppressive agents (steroids, immunomodulating drugs) during the 13 weeks preceding the screening visit or during the study period.
6. Known hypersensitivity to any of the vaccine components (adjuvant or protein) or anti-malarial treatments.
7. Body mass index (BMI) of <18 or >30 Kg/m^2.
8. Participants unable to be closely followed for social, geographic or psychological reasons.
9. Any vaccination from 4 weeks prior to the 1st vaccination and (none planned) up to 6 weeks after the 3rd vaccination or CHMI.
10. Symptoms, physical signs or laboratory values suggestive of systemic disorders, including renal, hepatic, cardiovascular, pulmonary, skin, immunodeficiency, psychiatric and other conditions, which could interfere with the interpretation of the trial results or compromise the health of the participants.
11. Abnormal electrocardiogram (ECG) on screening: pathologic Q wave and significant ST-T wave changes, left ventricular hypertrophy, clinically significant arrhythmias, left bundle branch block, secondary or tertiary A-V heart block.

12. Any clinically significant laboratory values at screening outside of normal ranges for study participants.
13. Malaria positivity at screening (Microscopy or qPCR positive).
14. Positive HIV, Hepatitis B (HBV) or Hepatitis C (HCV) tests.
15. For females: Positive pregnancy test or actively breast feeding.

5.5 Criteria for discontinuation of study

5.5.1 Withdrawal and discontinuation of individual participants

In accordance with the principles of the current version of the Declaration of Helsinki and any other applicable regulations, a participant has the right to withdraw from the study at any time and for any reason and is not obliged to give his or her reasons for doing so.

If a participant withdraws consent, no further evaluations should be performed and no attempts should be made to collect additional data, with the exception of safety data, which should be collected if possible and in accordance with the participant's agreement. The Sponsor may retain and continue to use any data collected before any withdrawal of consent. However, if the participant explicitly asks the Investigator to destroy all identifiable samples taken from the participant and does not allow to analyse his/her data, the Investigator will accept and will ensure to initiate all necessary steps to do so. Participant withdrawal and any agreements made with the participant will be documented in the participant's file.

The Investigator may discontinue a participant from the study at any time in the interest of the participant's health and well-being. In addition, the participant may be discontinued for any of the following reasons:

- Administrative decision by the Investigator
- Participant non-compliance with study requirements
- Any event which requires discontinuation of the study involvement or results in inability to continue to comply with study procedures
- Recommendation by the SMC (see section 11.4)

Prior to discontinuing a participant from study treatment, it is recommended that the Investigator contacts the Sponsor's Medical Monitor and forwards appropriate supporting documents for review and discussion. The decision to discontinue a participant remains the responsibility of the treating physician and will not be delayed or refused by the Sponsor. If a female participant is discontinued due to pregnancy, she will be referred to routine pre- or ante-natal care. Further, since there are unknown risks for the participant and the unborn child, they will be followed up for safety purposes by reviewing their ante-natal assessment reports until delivery or after the end of pregnancy.

The reason(s) for discontinuation will be recorded in the eCRF and source documents.

Participants who discontinue the study before receiving any study IMP (or control, according to randomisation) will be replaced. Participants who discontinue the study after having received at least one dose of study IMP (or control, according to randomisation) will not be replaced.

If, for any reason, a participant is discontinued from the study before his/her last visit, the Investigator is required, as feasible, to perform the safety procedures planned for the end of treatment. The discontinued participant will be requested to come for follow up visits on D112 and D140 for safety (clinical and laboratory) evaluations.

5.5.2 Discontinuation because stopping rule is met

If one of the stopping rules is met (see section 4.5), the study will be temporarily interrupted and the Sponsor will request an ad hoc meeting of the SMC to review the event(s) and associated safety data.

After the SMC review and communication of recommendation, Swiss TPH, Sumaya Biotech and the PI will decide if a longer term safety hold is needed for further investigation or the study can be continued. The Ethics Committees and RAs need to be informed accordingly.

The temporary interruption of the study means that no further vaccinations or CHMI can be performed. Remaining study procedures related to safety should be completed. Remaining study procedures related to research assays, including collection of samples, may be continued if the participant agrees and Swiss TPH, Sumaya Biotech and the PI confirm that it should be done.

6 TREATMENT OF PARTICIPANTS

6.1 Identity of investigational products

Vaccination part (n=40):

During the vaccination part, the participants will be randomised to either 150 µg MSP-1 + 5 µg GLA-SE (SumayaVac-1 (SUM-101)) or the comparator rabies vaccine (Verorab®)

CHMI part (n=25):

During the CHMI part of the study, all 25 participants will receive direct venous inoculation of 3.2×10^3 purified, cryo-preserved infectious sporozoites (PfSPZ Challenge (NF54 strain), Sanaria Inc.).

6.1.1 Experimental intervention vaccination part

Proprietary name	SumayaVac-1 (SUM-101)
International Nonproprietary Name (INN)	Not applicable
ATC code, if officially registered	Not applicable
Manufacturer MSP-1 malaria antigen	Praxis Pharmaceuticals, Vitoria, Spain
Manufacturer GLA-SE	Access to Advanced Health Institute (AAHI), Seattle, USA
Manufacturer 0.9% NaCl	B. Braun Melsungen AG
Mode of administration	Intramuscular injection (i.m.) in the deltoid muscle
Storage instructions	Lyophilized protein 2 – 8°C<br>Ready to use solution in 0.9% NaCl and adjuvant at room temperature for up to 48 h (chemically stable)
MSP-1	150 µg lyophilized MSP-1 protein in 2 ml stoppered vials
Adjuvant	GLA-SE (Glucopyranosyl Lipid Adjuvant-Stable Emulsion), 2 ml vials
Product after reconstitution	150 µg MSP-1 dissolved in 0.9% NaCl and emulsified with 250 µl of adjuvant GLA-SE

6.1.2 Experimental intervention CHMI part

Proprietary name	PfSPZ Challenge (NF54)
INN	Not applicable
ATC code, if officially registered	Not applicable
Manufacturer PfSPZ Challenge	Sanaria Inc., Rockville, USA
Mode of administration	Direct venous inoculation (DVI)

Storage instructions	Store in liquid nitrogen vapor phase (-150°C to -196°C).<br>Ready to use solution at room temperature for up to 4 h (chemically stable)
PfSPZ Challenge vial	20 µl in 0.7 ml plastic vials with screw caps or with caps with temper evident seals
Product after reconstitution	Diluted suspension of aseptic, purified, metabolically active, infectious, cryopreserved *P. falciparum* sporozoites in PfSPZ diluent

6.2 Comparator

The comparator Verorab® is an inactivated rabies virus vaccine.

It is supplied in a single-dose glass vial containing lyophilised powder (≥ 2.5 IU) with 0.5 ml of solution (sodium chloride 0.4%) in glass syringe.

The diluent is a solution of 0.4% sodium chloride and each glass vial contains maltose and human albumin as excipients.

The marketing authorisation holder is Sanofi Pasteur Ltd, Bangkok, Thailand.

The storage condition for the vaccine is 2-8°C and the reconstituted vaccine is a homogeneous, limpid solution without any particles in suspension.

For adults, the route of administration is i.m. injection in the deltoid region.

6.3 Packaging, labelling and supply

MSP-1 drug product will be labelled, packed, and released by Marken, Kelsterbach under the existing manufacturing license.

The adjuvant is imported, labelled, packaged and released by AAHI under the existing manufacturing and import authorization.

The sporozoites for CHMI challenge will be procured from Sanaria Inc. Packaging, labelling and release will be managed by Sanaria Inc.

The trial medication and control will be labelled according to Tanzanian requirements of Tanzania Medicines and Medical Devices Authority (TMDA).

6.4 Storage conditions

The vaccine must be transported and stored at 2-8°C while the sporozoites for the CHMI must be kept at all times between -150°C to -196°C. The IMP and the sporozoites should not be used beyond the expiry date indicated on the label. The IMP, control vaccine (Verorab®) and the sporozoites must be kept in a secure and limited access storage area (only accessible to authorized persons).

The storage conditions, including the temperature, at the study site must be monitored by the study personnel and appropriate records must be available. Any temperature excursion should be recorded and reported to the Sponsor immediately.

6.5 Administration of experimental and comparator interventions

6.5.1 Experimental intervention - vaccination part

Once randomised to either the SumayaVac-1 (SUM-101) or the rabies control vaccine, the participant will always receive the same dose of the same compound (150 µg MSP-1 protein dissolved in 0.9% NaCl with 250 µl of adjuvant GLA-SE or Verorab®) and there will be no dose adjustments foreseen. Both the SumayaVac-1 (SUM-101) and the rabies control vaccines will be applied as an i.m. injection in the deltoid muscle of the non-dominant arm. The three vaccine doses will be applied in 4 week intervals as described in the schedule of assessments (see section 4.3.1).

6.5.2 Experimental intervention - CHMI part

The participants selected for the CHMI will receive once a DVI of 3.2 x 10^3 purified, infectious sporozoites (PfSPZ Challenge (NF54 strain)) and no dose adjustments are foreseen. As described in the protocol, the CHMI will be done 4 weeks post 3rd vaccination.

6.6 Dose modifications

The doses are defined and described in section 6.1 for the vaccination part and the CHMI part. No dose modifications are foreseen.

6.7 Compliance with study interventions

The pharmacist will prepare the vaccine according to randomisation or CHMI for each participant and label it with the participant ID. Additional details regarding the labelling and dispensing will be described in the Pharmacy Manual.

The vaccinations and the CHMI will be administered at the study site under supervision of site personnel so full compliance is expected. Any non-compliance (such as participant refusal) will be documented accordingly.

6.8 Concomitant interventions (treatments)

Medications taken before the first vaccination will be documented as a prior medication. Medications taken after the first vaccination (e.g. to treat any AEs) will be documented as concomitant medications.

For any disease present at the screening visit, the investigator needs to assess:

- if part of the exclusion criteria.
- if either the disease or its treatment are expected to have any effect on the outcome measures used in this study trial.
- if either the disease or its treatment are expected to interfere with the study IMP.

If none of the above criteria are applicable, the concomitant treatment might be acceptable.

The following drug groups are **not permitted** as concomitant medication:

- In general, any regular systemic medication except contraceptives
- In particular, regular intake of the following drugs:
  - Immunosuppressants (e.g. systemically available glucocorticoids, calcineurin inhibitors)
  - Immunomodulators
  - Cytotoxic or cytostatic drugs

Prophylactic use, intake of more than 5 days or at a dose higher than recommended (refer to medication leaflet) of:

  - Anti-histamines
  - Analgesics

The following drug groups are permitted after individual counselling as concomitant medication:

- Topical medication (except if applied at the site of injection)
- Occasional, sporadic use of drugs not interfering with the immune system
- Hormonal contraceptives
- Food supplements (e.g. Vitamins)

Use of any prior and concomitant medication will be recorded in the source data and eCRF with the following information:

- Reason for treatment
- Name of the drug, type of formulation, route and unit strength
- Dose administered

- Time and duration of treatment

6.9 Experimental Intervention accountability

The MSP-1 drug product, GLA-SE and the sporozoites for CHMI will be stored at the site in accordance with Good Clinical Practice (GCP), GMP requirements and the instructions given by the Sponsor and will be inaccessible to unauthorized personnel. Special storage conditions and a complete record of batch numbers and expiry dates will be kept in the Sponsor's Trial Master File (TMF) and the site-specific elements of this information will be available in the Investigator Site File (ISF). On the day of receipt, the responsible study personnel will confirm receipt of MSP-1 drug product, GLA-SE and the sporozoites for CHMI as per the instructions supplied. The personnel will use MSP-1 drug product, GLA-SE and the sporozoites for CHMI only within the framework of this clinical study and in accordance with this protocol once the Sponsor has approved the use.

The pharmacist must maintain appropriate documentation on accountability of SumayaVac-1 (SUM-101), GLA-SE and the sporozoites for CHMI, including the following information:

- Delivered to the site
- Inventory at the site
- Use by each participant
- Returned or destroyed

6.10 Return or destruction of experimental Intervention

According to Sumaya Biotech's instructions, the unused MSP-1 drug product, GLA-SE and the sporozoites for CHMI are either destroyed in accordance with the site's SOP and national law or returned based on the sponsor's instructions.

7 STUDY ASSESSMENTS

7.1 Procedures per visit

7.1.1 General order of assessments

For the study periods described below, when multiple procedures are scheduled at the same time-point(s) relative to dosing, the following chronology of events should be adhered to, where possible:

- Vital signs (axillary temperature, blood pressure (BP), heart rate (HR) and respiratory rate (RR)). BP and HR after 10 minutes rest in the supine position
- Physical examinations
- AE monitoring (spontaneous and solicited AE monitoring will include specific questioning for tolerability and safety)
- Laboratory safety blood samples (biochemistry, haematology)
- 12-lead ECG after 10 minutes rest in the supine position
- Urine and stool samples will be collected when possible

Every effort should be made to ensure that protocol-required tests and procedures are completed as described. However, it is anticipated that from time to time there may be circumstances outside of the control of the Investigator that may make it unfeasible to perform a certain test or assessment. In those cases, the Investigator must take all steps necessary to ensure the safety and well-being of the participant. When a protocol required test or assessment cannot be performed, the Investigator will document the reason for it and any corrective and preventive actions which he/ she has taken to ensure that required processes are adhered to as soon as possible. The Sponsor study team must be informed of these incidents in a timely manner.

7.1.2 Screening visit 1 (D-35 to D-1)

Written informed consent will be obtained prior to any study procedures.

All healthy participants will be screened within 35 days before randomisation to confirm that they meet the participant selection criteria for the study. Screening can take place over several days to give the healthy participants sufficient time for reflection. Malaria testing should not be done more

than seven days prior to randomisation. Healthy participants who fail screening because they do not meet an inclusion criteria or meet an exclusion criteria temporarily can be re-screened at the Investigator’s discretion. In this case, a new screening procedure will be completed (new screening number, new informed consent, etc.). The previous screening number must not be re-used.

The following assessments will be done to collect the baseline safety data, and to assess the eligibility for the study:

- Demographics and information for identification card (ID).
- Vital signs (axillary temperature, blood pressure (BP), heart rate (HR) and respiratory rate (RR)). BP and HR after 10 minutes rest in a supine position.
- Anthropometrics (body weight and height).
- Complete medical history including prior and concomitant medication (i.e., prescription or non-prescription drugs and dietary supplements taken).
- Review of contraceptive use.
- Full physical examination.
- Check preliminary eligibility.
- Pregnancy test: all women of childbearing potential will undergo a serum pregnancy test.
- Urinalysis: Urine dipstick for protein, glucose and blood.
- Blood sampling for clinical safety laboratory evaluations:
  - Haematology: haemoglobin, WBC including differentiation of eosinophils and neutrophils, platelets.
  - Biochemistry: Alanine aminotransferase (ALT), Aspartate aminotransferase (AST), total bilirubin, creatinine and glucose (random). Troponin sample (at baseline) will be collected and stored to be run retrospectively if needed in case of a cardiac event. The sample collected at the time of the cardiac event will be compared with the sample collected at baseline.
- Blood sampling for serology (HIV, hepatitis B and hepatitis C).
- Blood sampling for exclusion of active clinical malaria by qPCR and TBS.
- 12-lead ECG. Healthy participants should rest in the supine position for 10 minutes before ECG measurements.
- Review of inclusion and exclusion criteria.

7.1.3 Screening visit 2 (D-35 to D-1)

Participants that passed screening visit 1, come for screening visit 2 to discuss the results from screening visit 1 and following assessments will be done:

- Discussion of findings from screening visit 1.
- Check of additional relevant medical history including prior and concomitant medication.
- Review of contraceptive use.
- Information on the use and supply of insecticide-treated bed nets.
- Review of inclusion and exclusion criteria and determine eligibility to participate in the study.

7.1.4 Pre 1st Vaccination (D-1 to D0)

The following assessments will be done **before randomisation:**

- Confirmation of willingness to participate.
- Vital signs (axillary temperature, BP, HR and RR (BP and HR after 10 min rest in a supine position)).
- Body weight measurement.
- Check of additional relevant medical history including prior and concomitant medication.
- Reminder of contraceptive use.
- Focused physical examination which involves an examination of the body system(s) related to participant’s abnormal presentation.
- Reminder of insecticide-treated bed nets use.
- Pregnancy test: all women of childbearing potential will undergo a serum pregnancy test.

- Urinalysis: Urine dipstick for protein, glucose and blood (if previous testing has been done >7 days ago).
- Blood sampling for haematology and biochemistry laboratory evaluations (if previous testing has been done >7 days ago).
- Blood sampling for baseline Malaria testing by qPCR and TBS.
- Blood sampling for baseline humoral & cellular response.
- Blood sampling for baseline exploratory samples.
- Urine and stool collection for retrospective exploratory analysis.
- Review eligibility for vaccination.

7.1.5 1st, 2nd and 3rd Vaccination visit (D0, D28 and D56)

After the pre-1st vaccination assessments have been completed, the randomisation is performed (see section 4.2.1). Then the participant can be dosed intramuscularly in the deltoid muscle of the non-dominant arm. After vaccination, the participant is then accompanied to the waiting area where he/she will stay for the next 2 hours. The participants will be closely observed in this time for any direct adverse events.

After 15 minutes, 30 minutes and 1 hour following assessments will be done:

- Vital signs (axillary temperature, BP, HR and RR (BP and HR after 10 min rest in a supine position)).
- Assessment of possible solicited and unsolicited AEs.
- Inspection and palpation of the injection site.

After 2 hours following assessments will be done:

- Vital signs such as axillary temperature, BP, HR and RR (BP and HR after 10 min rest in a supine position).
- Review of any concomitant medication.
- Focused physical examination which involves an examination of the body system(s) related to participant's abnormal presentation.
- Assessment of possible solicited and unsolicited AEs.

If not contraindicated by an AE or any other reason, the participant can be released for home after the 2 hours assessment.

Following the 1st vaccination, the participants will be escorted home, so that the study staff know where the participant resides to help them trace a participant in event of a missed scheduled appointment.

7.1.6 1-6 days post 1st, 2nd and 3rd vaccination follow-up (D1-6, D29-34 and D57-62)

The participant is followed up daily after each vaccination and before the next site visit either by a telephone call and/ or if deemed necessary by the study team, by a home visit.
Following assessments are done each time during the follow-up:

- Assessment of possible solicited and unsolicited AEs.
- Follow-up of unresolved AEs.
- Review of any concomitant medication.

7.1.7 7 day site visit post 1st, 2nd and 3rd vaccination (D7, D35 and D63)

Participants come for an ambulatory visit to assess AEs and give blood samples for immunological analyses. The visit includes the following procedures:

- Vital signs (axillary temperature, BP, HR and RR (BP and HR after 10 min rest in a supine position)).
- Review of concomitant medication.
- Review of contraceptive use.

- Focused physical examination which involves an examination of the body system(s) related to participant's abnormal presentation to assess solicited and unsolicited AEs.
- Reminder of insecticide-treated bed nets use.
- Capturing of solicited and unsolicited AEs.
- Follow-up of unresolved AEs.
- Blood sampling for haematology and biochemistry laboratory evaluations.
- Blood sampling for exploratory samples (only on D63).
- Blood sampling for Malaria testing by qPCR and TBS (only on D63).

7.1.8 14 day site visit post 1st, 2nd and 3rd vaccination (D14, D42 and D70)

Participants come for an ambulatory visit to assess and follow-up on AEs. The visit includes following procedures:

- Vital signs (axillary temperature, BP, HR and RR (BP and HR after 10 min rest in a supine position)).
- Review of concomitant medication.
- Review of contraceptive use.
- Focused Physical Examination which involves an examination of the body system(s) related to participant's abnormal presentation to assess unsolicited AEs.
- Reminder of insecticide-treated bed nets use.
- Follow-up of unresolved AEs.
- Capturing of unsolicited AEs.
- Blood sampling for haematology and biochemistry laboratory evaluations.

7.1.9 28 day site visit post 1st, 2nd and 3rd vaccination (D28, D56 and D84)

The following assessments will be done on the day of the next vaccination (D28 and 56) or CHMI (D84) **before the participant is vaccinated or challenged with CHMI (as applicable).**

- Confirmation of willingness to participate.
- Vital signs (axillary temperature, BP, HR and RR (BP and HR after 10 min rest in a supine position)).
- Body weight measurement.
- Review of concomitant medication.
- Review of contraceptive use.
- Focused physical examination which involves an examination of the body system(s) related to participant's abnormal presentation.
- Reminder of insecticide-treated bed nets use.
- Capturing of unsolicited AEs.
- Follow-up of unresolved AEs.
- Pregnancy test: all women of childbearing potential will undergo a serum pregnancy test.
- Urinalysis: Urine dipstick for protein, glucose and blood.
- Blood sampling for haematology and biochemistry laboratory evaluations.
- Blood sampling for Malaria (qPCR and TBS).
- Blood sampling for humoral & cellular response.
- Blood sampling for exploratory samples.
- Urine and stool collection for retrospective exploratory analysis.
- Review of eligibility for 2nd/3rd vaccination or CHMI.
- Random selection of participants for CHMI (only on D84).

7.1.10 CHMI (D84)

Participants that are eligible for CHMI (as evaluated based on assessments performed on D84, see section 7.1.9) and are randomly selected will be dosed by DVI in a forearm vein. After CHMI, the participant is then accompanied to the waiting area where he/she will stay for the next 2 hours. The participants will be closely observed in this time for any direct adverse events.

After 15 minutes, 30 minutes and 1 hour, following assessments will be done:

- Vital signs (axillary temperature, BP, HR and RR (BP and HR after 10 min rest in a supine position)).
- Assessment of possible solicited and unsolicited AEs.
- Inspection and palpation of the injection site.

After 2 hours following assessments will be done:

- Vital signs such as axillary temperature, BP, HR and RR (BP and HR after 10 min rest in a supine position).
- Review of any concomitant medication.
- Focused Physical Examination which involves an examination of the body system(s) related to participant's abnormal presentation.
- Assessment of possible solicited and unsolicited AEs.

If not contraindicated by an AE or any other reason, the participant can be discharged home after the 2 hours assessment.

7.1.11 1-4 days post CHMI (D85-88)

The participant is followed up after the CHMI and until 4 days post CHMI, before the confinement period starts at the study site. This is done by either a telephone call and/or if deemed necessary by the study team, by a home visit.

Following assessments are done each time during the follow-up:

- Assessment of possible solicited and unsolicited AEs.
- Follow-up of unresolved AEs.
- Review of any concomitant medication.

7.1.12 Site stay post CHMI (D89- maximum D115)

On day 5 post CHMI the participant returns back to the site and is admitted. The participant will remain within the facility until discharged after his malaria treatment (see 7.1.14).

Following procedures will be done **at admission**:

- Vital signs (axillary temperature, BP, HR and RR (BP and HR after 10 min rest in a supine position)).
- Review of concomitant medication.
- Focused physical examination which involves an examination of the body system(s) related to participant's abnormal presentation.
- Assessment of possible solicited and unsolicited AEs.
- Follow-up of unresolved AEs.
- Blood sampling for haematology and biochemistry laboratory evaluations.
- Blood sampling for malaria qPCR and TBS.
- Blood sampling for intentional malaria drug intake by participant.

For the rest of the site stay, the following procedures will be done:

Once a day

- Blood sampling for TBS.

Twice daily until D98 (CHMI + 5 to 14 Days), thereafter once a day until D112

- Blood sampling for malaria (qPCR).
- Vital signs (axillary temperature, BP, HR and RR (BP and HR after 10 min rest in a supine position)).
- Review of concomitant medication.

- Focused Physical Examination which involves an examination of the body system(s) related to participant's abnormal presentation.
- Assessment of possible solicited and unsolicited AEs (solicited AE check will be done till D91 (7 days post CHMI))

After **one week of confinement (D96)** blood sampling for haematology and biochemistry laboratory evaluations is repeated.

7.1.13 Malaria treatment post-CHMI (between D89-112)

Criteria for being considered Malaria positive post-CHMI:

- qPCR sample gives a value of >500 parasites/µl.
- Malaria symptoms plus a positive RDT (see Table I below).

Scenario	qPCR	TBS	Symptoms	Action plan
1.	Negative	Negative	Negative	Keep observing participant
2.	Negative	Negative	Positive	If participant has malaria symptoms, initiate RDT every 6 hours<br>• If RDT is positive, treat with anti-malarials.<br>• If any of 6 hourly RDT is negative, and symptoms subside, continue with normal follow up schedule.<br>• If three consecutive 6 hourly RDTs are negative, and symptoms persist, clinician may decide to treat based on clinical evaluation. Treatment could include anti-malarials.<br>In case of qPCR, TBS and RDT negative results but presence of symptoms, clinician should start evaluation of other aetiologies.
3.	Negative	Positive	Negative	Keep observing participant

Note:

a. A negative qPCR result is assessed as either parasites not detected or detected at ≤500 parasites/µl
b. In case a participant develops malaria symptoms and also has a positive thick blood smear (at a density of >500 parasites/ µl), anti-malarial treatment will be initiated

Table I: Action plan in case of malaria symptoms

If the participant fulfils one of the criteria mentioned above **OR** reaches D112 without being tested positive for malaria, following procedures will be done at that time:

- Vital signs (axillary temperature, BP, HR and RR (BP and HR after 10 min rest in a supine position)).
- Review of concomitant medication.
- Focused physical examination which involves an examination of the body system(s) related to participant's abnormal presentation.
- Assessment of possible solicited and unsolicited AEs (solicited AE check will be done till D91 (7 days post CHMI).
- Follow-up of unresolved AEs.
- Blood sampling for haematology and biochemistry laboratory evaluations.
- Blood sampling for exploratory samples (volume = 10 ml).
- Blood sampling for malaria qPCR, TBS and genotyping (only if malaria positive).
- Initiation of anti-malarial treatment with ALU plus a single dose of primaquine.

The participant then has to remain in the facility until malaria treatment (3 days) is completed. After initiation of treatment, clinical review will be conducted twice daily to monitor the progress of the participants. qPCR will be conducted daily to monitor the progress of malaria parasite clearance. If 2 consecutive post diagnosis qPCR results are negative (e.g., at 24 and 48 hours post diagnosis),

and the patient has completed the treatment dose (ALU + low dose primaquine) and is clinically stable as judged by the study clinicians, then the participant will be discharged from the Clinical Trial Facility. Participants who remain negative for malaria, do not reach >500 parasites/µl threshold and who do not develop symptoms until CHMI +28 days (D112), will be treated presumptively with ALU + low dose primaquine under direct observation and will be discharged upon completion of treatment and on discretion of the study clinician on D115 (see below). If participants progress to severe malaria, they will be treated according to standard treatment guidelines of Tanzania.
The Pf NF54 strain that will be used for CHMI has been tested and confirmed to be sensitive to ALU, the drug that we will use for treatment. However, in case parasite resistance to ALU is observed, the participant will be managed and treated as per ministry of health guidelines of Tanzania.

7.1.14 Site discharge post CHMI confinement (between D89-115)

Once the malaria treatment is completed, the participant undergoes following procedures before being discharged:

- Vital signs (axillary temperature, BP, HR and RR (BP and HR after 10 min rest in a supine position)).
- Body weight measurement.
- Review of concomitant medication.
- Reminder of contraceptive use.
- Focused physical examination which involves an examination of the body system(s) related to participant's abnormal presentation.
- Reminder of insecticide-treated bed nets use.
- Assessment of possible unsolicited AEs.
- Follow-up of unresolved AEs.
- Urinalysis: Urine dipstick for protein, glucose and blood.
- Blood sampling for haematology and biochemistry laboratory evaluations.
- Blood sampling Malaria (qPCR and TBS).

Once confirmed that no parasites can be detected in 2 consecutive qPCRs and the participant is of general good health she/he can be discharged home.

However, if the discharge is done after D105, the discharge and the D112 visit can be combined. In order to do that, the following procedures will need to be done in addition to the procedures described above (for the discharge visit):

- Blood sampling for humoral response.
- Blood sampling for cellular immune response.
- Blood sampling for exploratory sample (volume = 25 ml).

7.1.15 Vaccination site follow-up (D112, D140) and CHMI long-term site follow-up visits (D168, D252)

Following procedures are to be done on each follow-up visit:

- Vital signs (axillary temperature, BP, HR and RR (BP and HR after 10 min rest in a supine position)).
- Body weight measurement.
- Review of concomitant medication.
- Review of contraceptive use (on D112 (W16), D140 (W20) and D168 (W24)).
- Focused physical examination which involves an examination of the body system(s) related to participant's abnormal presentation.
- Reminder of insecticide-treated bed nets use.
- Assessment of unsolicited AEs.
- Follow-up of unresolved AEs.
- Pregnancy test: all women of childbearing potential will undergo a serum pregnancy test.

- Blood sampling for biochemistry laboratory evaluations.
- Blood sampling for humoral response.
- Blood sampling for cellular immune response (on D112 and D140).
- Blood sampling for exploratory samples (only D112).
- Blood sampling Malaria (qPCR and TBS)

If the participant is discharged after D105, the visit can be combined with the discharge visit as described in section 7.1.14.

7.1.16 Unscheduled visit

Unscheduled visits may occur at any time during the study. The procedures below are to be performed at any unscheduled visit.

- Vital signs (axillary temperature, BP, HR and RR (BP and HR in a supine position)), if clinically indicated.
- Body weight measurement.
- Review of concomitant medication.
- Focused physical examination which involves an examination of the body system(s) related to participant's abnormal presentation.
- Assessment of AEs.
- Follow-up of unresolved AEs.
- Clinical safety laboratory evaluations (e.g., haematology, biochemistry, urinalysis), if clinically indicated or required based on the Investigator's judgement.

Unscheduled visit forms will also be used to document data collected due to prolongation of the confinement period in case the participant still presents symptoms of malaria or based on the Investigator's judgement.

7.2 Total blood volume

The blood volumes planned to be collected from each participant during the course of this study are detailed in section 4.3.1. Additional samples may be required in the event of AEs/SAEs, additional laboratory safety tests or in case a laboratory test needs to be repeated as judged by the Investigator.

8 ASSESSMENT OF SAFETY

8.1 Safety assessments

General questioning about solicited and unsolicited AEs will be done at every visit after administration of the vaccination or CHMI.

During confinement after the CHMI, the general questioning about solicited and unsolicited AEs will be done twice a day between D89-D98 (see details in section 7.1.12).

8.1.1 Physical Examination

A complete physical examination will be conducted at screening and will assess general appearance including height (at screening 1 only) and weight and the following areas/ symptoms: skin, lymph nodes, head and neck, eyes, ears, nose, throat, respiratory, cardiovascular, abdomen, extremities, musculoskeletal neurological and urogenital.

A targeted symptom-directed physical examination will be assessed at designated study visits based on interim medical history and the participant's complaints/observations. At any time, a symptom-directed physical examination can be performed as deemed necessary by the Investigator.

Abnormalities assessed as clinically significant will be reported as an AE.

8.1.2 Vital Signs

Vital signs (BP, HR, axillary temperature and RR) will be collected in the source documents at each study visit and twice daily during the confinement after CHMI for medical purposes.

HR and BP will be taken after 10 minutes rest in a supine position.

During the trial, vital signs will be repeated if they fall outside the normal ranges [49].

If the result of the repeat measurement is still out of range, the Investigator will make an assessment of clinical significance. If the abnormality is assessed during the screening visit, it is captured as medical history. After the participant has received his/her first vaccination, clinically significant abnormalities will be reported as an AE. The investigator will decide on an appropriate course of action.

8.1.3 12-lead ECG safety recording

One ECG is performed at screening visit 1. It can also be performed if there are signs and symptoms suggesting cardiac problems.

Instructions for recording and handling of the ECG will be included in a separate Manual. Repeats will be done in case of an erroneous reading.

The following complaints are considered potentially related to cardiac complications in the adult age group and may lead to cardiac evaluation, including the consultation of a cardiologist (independent physician) if deemed necessary by the investigator:

- Chest pain (other than chest wall pain or respiration-related pain)
- Palpitations
- Shortness of breath

These symptoms can occur in individuals with malaria, especially after initiation of anti-malarial treatment, but a cardiac aetiology is also to be considered. In addition, investigators may also seek cardiac consultation based on other symptoms judged to be cardiac related.

8.1.4 Laboratory assessments

The following laboratory assessments will be conducted in accordance with the local laboratory’s standard operating procedures.

One or more of the laboratory parameters may be repeated at any time during the study as determined by the PI or if indicated by an SAE/AE.

Laboratory abnormalities after the first vaccination assessed as "clinically significant" have to be reported as an AE (section 8.2) only if they meet at least one of the following conditions:

- The abnormality suggests a disease and/or organ toxicity AND this abnormality was not present at the screening visit, or is assessed as having evolved since the screening visit.
- The abnormality results in discontinuation of the study IMP.
- The abnormality requires medical intervention or concomitant therapy.

When reporting an abnormal laboratory result, a clinical diagnosis should be recorded rather than the abnormal value itself, if available (for example, "anaemia" rather than "decreased red blood cell count").

Haematology

Haemoglobin, WBC (differentiation of eosinophils and neutrophils), platelets and haematocrit.

Biochemistry

Biochemistry parameters at screening will include: ALT, AST, total bilirubin, creatinine and glucose (random). Troponin sample (baseline) will be collected and stored to be run

retrospectively if needed in case of a cardiac event. The sample collected at the time of the cardiac event will be compared with the sample collected at baseline.

During subsequent scheduled visits ALT, total bilirubin and creatinine evaluations will be performed.

Urinalysis performed by dipstick

Proteinuria, glucose and blood. (**Note:** Urinalysis should be deferred if a participant is menstruating, but should be performed as soon as possible).

8.2 Collection and reporting of adverse events

8.2.1 Definition of adverse events (AEs)

An adverse event (AE) is defined as any untoward medical occurrence (including a clinically significant abnormal laboratory finding, for example) in a clinical research participant administered with a pharmaceutical product and which does not necessarily have to have a causal relationship with this treatment. Definition of an AE includes worsening (in severity or frequency) of a pre-existing condition ("medical history") before first IMP administration and abnormalities of procedures (i.e. ECG, physical, or neurological examination, etc.) or laboratory results which are assessed as "clinically significant".

8.2.2 Types and recording of AEs

The term "AE" could include any of the following events which develop or increase in severity during the course of the study:

a. Any signs or symptoms whether thought to be related or unrelated to the vaccination/ CHMI under study
b. Any clinically significant laboratory abnormality
c. Any abnormality detected during a physical examination

These data will be recorded in the CRFs, regardless of whether they are thought to be associated with the study or the drug under investigation.

8.2.3 Grading of adverse events severity

For each AE, the Investigator is required to assess the maximum severity. The distinction between severity and seriousness of AEs is to be noted. A severe AE is not necessarily an SAE.

All AEs/SAEs (except laboratory abnormalities and vital signs) will be graded by the Investigator using the terminology: mild, moderate, severe, life-threatening or death according to the following definitions:

- Mild (Grade 1): These events do not interfere with the participant's daily activities.
- Moderate (Grade 2): These events cause some interference with the participant's daily activities and require limited or no medical intervention.
- Severe (Grade 3): These events prevent the participant's daily activities and require intensive therapeutic intervention.
- Life-Threatening (Grade 4): The subject is at significant risk of life; it does not refer to an event which hypothetically might have caused death if it were more severe (life-threatening consequences, urgent intervention required).
- Death (Grade 5) Death related to an event.

Laboratory abnormalities and vital signs listed on the site's grading scheme adapted from FDA Guidance for Industry Toxicity Grading Scale for Healthy Adult and Adolescent Volunteers Enrolled in Preventive Vaccine Clinical Trials document [50] will be graded according to this document. The Investigator's medical and scientific judgement will decide whether an abnormal laboratory finding or other abnormal assessment is clinically significant or not.

8.2.4 AE causality assessment

For all AEs, the Investigator is required to assess if there is a causal relationship between the AE and the IMP, i.e. to determine whether there exists a reasonable possibility that the IMP caused or contributed to the AE(s).

The following categories for relationship to treatment will be used during AE reporting:

- Definitely related: The AE and administration of IMP are related in time, and a direct association can be demonstrated
- Probably related: The AE and administration of IMP are reasonably related in time, and the AE is more likely explained by the IMP than other causes
- Possibly related: The AE and administration of IMP are reasonably related in time, and the AE can be explained equally well by causes other than the IMP
- Unlikely related: A potential relationship between IMP and the AE could exist (i.e. the possibility cannot be excluded), but the AE is most likely explained by causes other than the IMP
- Not related: The AE is clearly explained by another cause not related to the IMP

Note: for regulatory reporting purposes, when compared to binary classification, “not related” corresponds to “not related, unlikely related” and “related” corresponds to “possible, probable and definitely related”.

In addition, for SAEs, the Sponsor will also perform a causality assessment.

8.2.5 Serious adverse events (SAEs)

A “serious” adverse event (SAE) is defined as any event that suggests a significant hazard, contraindication, side effect or precaution. A serious adverse event includes any event that:

1. is **fatal**.
2. is **life threatening**, meaning, the participant was, in the view of the Investigator, at immediate risk of death from the reaction as it occurred, i.e., it does not include a reaction that, had it occurred in a more serious form, might have caused death.
3. is a **persistent or significant disability or incapacity**, i.e., the event causes a substantial disruption of a person’s ability to conduct normal life functions.
4. requires, or prolongs **in-patient hospitalization**; i.e. the AE requires at least an overnight admission or prolongs a hospitalization beyond the expected length of stay according to the protocol. Hospital admissions for surgery planned before study entry, for social reasons, for any elective surgery (i.e. plastic surgery) are NOT to be considered as SAE according to this criterion.
5. is a **congenital anomaly or birth defect**.
6. is an **important medical event**, based upon appropriate medical judgement, that may jeopardize the participant or may require medical or surgical intervention to prevent one of the other outcomes defined as serious.

8.2.6 Expectedness of AEs

The following are local solicited AEs occurring within 7 days after vaccination:

- Warmth
- Erythema
- Itching
- Edema
- Pain
- Infection
- Lipodystrophy

- Ulceration
- Induration
- Necrosis
- Other tissue damage

Note: If needed, photograph(s) of injection site will be taken for clinical evaluation

The following are general solicited AEs occurring within 7 days after vaccination:

- Fatigue
- Chills
- Sweating
- Myalgia
- Arthralgia
- Gastrointestinal symptoms (e.g. nausea, vomiting)
- Headache
- Fever >38°C
- Rash

The following are malaria-related symptoms and signs (solicited AEs) occurring after the CHMI until the participant is considered malaria positive (see section 7.1.12):

- Fever >38°C
- Fatigue
- Headache
- Sweating
- Myalgia
- Arthralgia
- Gastrointestinal symptoms (e.g. nausea, vomiting)
- Malaise
- Diarrhea
- Abdominal pain
- Chest pain
- Shortness of breath
- Palpitations
- Dizziness
- Rigors
- Cough

The Sponsor is responsible for further determining the expectedness of the event, using the Investigator Brochure of the IMP and rabies control vaccine.

8.2.7 Reporting Obligations

The AE reporting period begins with the administration of the 1st vaccination and ends with the last study visit.

Each AE is to be classified by the Investigator as serious or non-serious. This classification will determine the reporting procedure for the event.

All SAEs are to be reported **immediately** (within 24 hours of awareness of the SAE by the Investigator) **to the Sponsor in writing**. All written reports should be transmitted to the Sponsor by e-mail using the SAE report form. This includes a description of the event, onset date and type, duration, severity, relationship to the IMP, outcome, measures taken and all other relevant clinical and laboratory data. In exceptional circumstances, an SAE (or follow-up information) may be

reported by telephone, followed by a completed SAE report form. The initial report is to be followed by submission of additional information (follow-up SAE form) as it becomes available.

SAEs should also be reported on the AE page of the eCRF. It should be noted that the form for reporting of SAE (SAE form) is not the same as the AE section of the eCRF. Where the same data are collected, the two forms must be completed in a consistent manner, and the same medical terminology should be used.

Expedited reporting to Regulatory Authorities (TMDA), Independent Ethics Comittee(s) - National Health Research Ethics Review Committee (NatHREC) and Institutional Review Board(s) (IHI-IRB) will be done by the Investigator on behalf of the Sponsor in following their reporting requirements. Expedited reporting will be done in a blinded manner unless an SAE is thought to be related to the IMP or unblinding is necessary to inform the management of the participant condition.

A suspected unexpected serious adverse reaction (SUSAR) is a suspected AE related to an IMP that is both unexpected and serious.

8.3 Follow-up of adverse events

All AEs should be followed until:

- they are resolved; or
- the Investigator assesses them as “chronic” or “stable”; or
- the participant’s participation in the trial ends (i.e., until their follow-up visit is complete, or otherwise until the last contact with the subject).

In addition, all SAEs (related or not) and those non-serious events assessed by the Investigator as having a reasonable possibility of relationship to the IMP must continue to be followed even after the subject participation in the trial is over. Such events should be followed until they resolve or until the Investigator assesses them as “chronic” or “stable.” The outcome of these events is to be documented in the eCRF (up to the end of study participation) and SAE form (up to and after the end of study participation).

All SAEs will be summarized and reported to IHI-IRB and NatHREC in the required timeframes. The PI, on behalf of the Sponsor, will also report all SAEs to the TMDA within the specified timeframe as required by the regulations.

8.4 Data collection and follow-up of prematurely terminated participants

See section 5.5.1.

9 STATISTICS

9.1 Hypothesis

No formal statistical hypothesis testing is planned for this study due to its descriptive and exploratory nature.

9.2 Determination of sample size

No formal sample size calculation has been performed. The sample size of 40 participants (20 per arm) is within the range of those typically used in early phase trials. It is expected to allow assessment of the safety, reactogenicity and immunogenicity of SumayaVac-1 (SUM-101) in malaria exposed adults of African origin in this setting. The sample size of 25 participants for the CHMI is chosen based on operational considerations.

9.3 Description of statistical methods

Analysis and reporting will follow ICH E9 [51, 52]. All analyses will be performed by the trial statistician, or a suitably qualified delegate with oversight by the trial statistician.

Analyses will be performed for reporting to the SMC, according to the schedules outlined in section 4.5. These analyses will include safety data and will be performed in a blinded manner, with both arms (namely, SumayaVac-1 (SUM-101) or Verorab® control) combined.

The final analysis for the study is planned after all participants have completed follow-up. For the final analysis, the trial statistician will be unblinded, and results will be presented separately by arm.

The numbers of individuals screened and reasons for screening failure will be summarized. The numbers of participants enrolled along with their demographic and other baseline characteristics will be summarized. The completion of follow-up will be summarized, along with details of discontinuations or withdrawals.

Continuous variables will be inspected using histograms: 1) to assess for outliers which may be queried for accuracy, and 2) to assess whether appropriate transformations are required for analyses. In general, continuous variables will be summarized using descriptive statistics such as number, mean, standard deviation, median, maximum and minimum, after transformation if necessary. Categorical variables will be reported as frequencies and percentages. Completeness of data will be summarized. Analyses will be descriptive with no formal comparisons between arms. Measures of precision (such as 95% confidence intervals) for endpoints may be provided where appropriate in order to inform subsequent clinical evaluation of the IMP. Graphical depictions will be used where appropriate to illustrate the data.

9.3.1 Datasets to be analysed, analysis populations

Participant inclusion into each analysis population will be determined prior to the final analysis. The analysis populations will be as follows:

- Intent-to-Treat (ITT) population: all randomised participants.
- Per Protocol (PP) population: subset of the ITT population, who completed the study without any major protocol violation.
- Safety population: all participants having received at least one vaccination.
- CHMI population: all participants having received CHMI.

Participant disposition and demographic data analyses will be based on the ITT population, according to the randomisation arm. All other analyses will be based on the vaccinations received, regardless of the randomisation (any such deviations will be reported).

Participants who drop out of the study before receiving any vaccination will be replaced. In this case, the replacement participant would receive the same allocation as planned for the participant who dropped out, in order to maintain the correct overall numbers of participants allocated to each arm. This is justified by the replaced participant not having received any vaccination, and concealment of allocation being maintained by the blinded nature of the study. Participants who drop out of the study after receiving at least one vaccination will not be replaced.

9.3.1.1 Primary Analysis

In general, AEs and SAEs will be captured using the Medical Dictionary for Regulatory Activities (MedDRA) preferred terms grouped by primary system organ class. Results will include the total occurrences of each type of endpoint, and the number of participants experiencing at least one of each type of endpoint.

For the primary endpoints of (i) local and systemic solicited AEs at least possibly related to IMP recorded from after each vaccination (done on D0, D28 and D56) up to 7 days later and (ii) local and systemic unsolicited reactogenicity recorded from after each vaccination (done on D0, D28 and D56) up to 28 days later, results will be reported overall, and separately by vaccination (first, second, third).

Individual listings of AEs and SAEs (including relevant participant information such as age and gender; and AE details such as onset date, duration, severity, seriousness, expectedness, relation to study drug; see section 8.2) will be provided.

Laboratory safety parameters will be summarized as absolute values. Changes in laboratory safety parameters will be assessed between before each vaccination (done on D0, D28 and D56) and at 28 days later, separately for each vaccination (first, second, third). Changes will also be assessed at 28 days after each vaccination, compared to baseline values (before 1st vaccination).

To evaluate the immunogenicity primary endpoints (see section 4.1.1), antibody responses will be summarized over time, and fold changes of antibody responses will be summarized over time relative to baseline.

9.3.1.2 Secondary Analyses

The secondary endpoints (see section 4.1.2) will be summarized over time, (a) among all participants and (b) among participants undergoing CHMI to allow descriptive assessment in trends over time following CHMI. Continuous and categorical endpoints will be analysed as described in section 9.3.

The following analyses will also be performed:

- Clinical and laboratory safety data (such as haematology, biochemistry, urinalysis, vital signs, physical examination) will be described over time. Changes versus baseline will also be presented, along with AE gradings as appropriate.
- Concomitant therapy will be summarized.

9.3.1.3 Interim analyses

No interim analyses are planned, other than those performed for the SMC. The SMC will perform reviews according to the schedules outlined in section 4.5. Stopping rules and possible outcomes of the SMC meetings are detailed in section 4.5. As hypothesis testing will not be performed, no adjustments to the statistical analysis for multiple testing are required.

9.3.1.4 Safety analysis

Safety parameters are primary endpoints of the trial; see analyses described in section 9.3.1.1.

9.3.1.5 Deviation(s) from the original statistical plan

A full statistical analysis plan will be finalised before any substantial analysis of the data. Deviations from the original statistical analysis plan will be avoided if possible, but if necessary, would be reported in the final report along with justifications for those deviations.

9.4 Handling of data

Data will be summarized and inspected for potential errors. Missing data will be summarized, and percentages will be reported of those with non-missing data. Drop outs (and reasons, if captured) will be reported. There are no imputations planned for missing data or replacement of participants who drop out, except as detailed in section 4.2.1.

10 ETHICAL CONSIDERATIONS

The study will be carried out in accordance to the protocol and with principles enunciated in the current version of the Declaration of Helsinki, the guidelines of GCP issued by ICH, the local law and RA's requirements. In agreement with local requirements, the IEC and RA will receive regular safety reports and will be informed about study stop/end.

10.1 Independent Ethics Committee (IEC)

Prior to enrolment of any participant into this trial, the study protocol and the informed consent form (ICF) will be reviewed and approved by the IHI-IRB and NatHREC in Tanzania.

In parallel, the trial will be submitted to the IEC in Switzerland (Ethikkommission Nordwest- und Zentralschweiz (EKNZ)) to get an ethical statement of the trial.

Premature study end or interruption of the study will be reported within 15 days by the Sponsor or by the PI to the IRB (IHI-IRB) and IECs (NatHREC and EKNZ). The regular end of the study and

the final clinical study report will be submitted in accordance with the applicable timelines after the end of the study.

10.2 Regulatory Authority (RA)

Prior to the start of the clinical trial, approval from the Tanzanian Regulatory Authority- TMDA, will be obtained.

Premature study end or interruption of the study will be reported within 15 days to the competent authority. The regular end of the study and the final clinical study report will be submitted in accordance with the applicable timelines after the end of the study by the PI.

10.3 Evaluation of the risk-benefit ratio

Potential risks with the IMP and the CHMI are detailed in section 2.7.

The participants are unlikely to benefit from the study IMP, but they may profit from a comprehensive health check and protection from rabies upon receiving Verorab rabies vaccine. If a chronic or underlying disease is identified, participants will be referred accordingly within the national health system. As for any Phase I clinical trial, it is not possible to claim any protection from the disease the vaccine is targeting.

In addition, there is the potential that the gained knowledge could help in the development of new vaccines against malaria. This could lead to societal benefits if a new vaccine is identified. The general risks to participants in this study are associated with phlebotomy and with vaccination. The blood volume drawn over the course of the study should not compromise the otherwise healthy subjects.

10.4 Participant information and consent

Written informed consent of a participant, using the IEC approved consent form, must be obtained before any study procedure is performed. Upon showing interest, IEC-approved language and an appropriate and study-specific Participant Information Sheet(s) (PIS) will be made available to the potential participant and the opportunity to discuss the study will be given.

The participants will be fully informed orally by the Investigator or designee about all aspects of the trial, the potential risks and their obligations. The following general principles will be emphasized:
The participation in the study is entirely voluntary.

- The refusal to participate involves no penalty or loss of medical benefits.
- The participant may withdraw from the study at any time.
- The participant is free to ask questions at any time to allow him/her to understand the purpose of the study and the procedures involved.
- The study involves research of an investigational vaccine.
- There is no direct benefit from participating.
- The participant’s general practitioner may be contacted to corroborate their medical history or seek additional information.
- The participant’s blood samples taken as part of the study will be stored at the site and samples will be sent outside Tanzania to collaborating laboratories (see section 15.1). These samples will be identified only by code numbers.
- The aims of the study and tests to be carried out will be explained.
- Reimbursement will be provided for participation as described in section 10.9.
- All relevant clinical and laboratory results (serology, lab tests and interpretations of physical exams) will be shared directly with the concerned participants.
- Relevant aspects regarding data protection and confidentiality.

The participant will be given enough time to make an informed decision about his/her participation in the study. The participant will be asked to read, and consider the statement before signing and dating the ICF. The consent form must be signed and dated by the Investigator (or their designee)

at the same time as the participant. A copy of the fully signed form and PIS will be given to the participant and the original will be retained as part of the study records.

10.5 Registration of clinical trial

The study will be registered with a recognized clinical study registry, e.g., www.clinicaltrials.gov, before commencing any study procedures.

10.6 Participant confidentiality

The Investigator must assure that participants' confidentiality will be maintained and that their identities are protected from unauthorized parties.

Participants' identities will not be disclosed to the Sponsor. The Investigator will maintain a Participant Identification List so that the participants can be identified. The list will stay under secure control of the site with strict access control processes in place.

Individual participant medical information obtained as a result of this study is considered confidential and non-anonymized disclosure to third parties is prohibited. Participant confidentiality will be ensured by utilizing participant identification codes. No patient identifiable data will be collected in the electronic data capture (EDC) tool except for date of birth, as this information is critical for the inclusion of participants and for the statistical analysis of the data. However, data minimization is applied and only month and year of birth are collected for participants.

As part of the informed consent process, the participants will be informed in writing that representatives of the Sponsor (like monitors, auditors, etc.), a RA or Ethics Committee may require direct access to parts of the medical records relevant to the study, including participant's medical history, and that all personal data will be handled strictly confidentially and in accordance with local data protection laws.

Blood samples will be collected at BRTC, Tanzania and samples used for testing of some secondary and exploratory endpoints will be shipped to Germany, Switzerland, Italy and USA.

Study results will be published without providing individual information on the participants that can identify them.

10.7 Participants requiring particular protection

Only healthy and literate adults aged 18-45 years will be asked to participate in this Phase Ib clinical trial.

10.8 Insurance

A clinical trial insurance will be provided by the Sponsor. A copy of the certificate will be filed in the Investigator Site File and the Trial Master File.

The Sponsor is insured to indemnify the Investigator against any claim for damages brought by a participant who suffers from a research- related injury during the performance of the trial according to the protocol, except for claims that arise from malpractice and/or negligence. This is covered by the clinical trial's host institute (Ifakara Health Institute).

In accordance with local regulations, the Sponsor will contract insurance for all study participants.

The Bagamoyo Clinical Trial Facility (BCTF) will be primarily responsible for providing participant's medical care whether it is related or not related to the study for the duration of the trial. If specialist, emergency care or hospitalization is needed, this will be provided at no cost to the participant at the Bagamoyo District Hospital or referral centers in Dar-es-Salaam, Tanzania.

For all unrelated conditions to the study procedures, the participant will be responsible for seeking and funding this care through the normal channels provided by the Ministry of Health and Social Welfare of Tanzania. The obligation to provide medical care for conditions arising during the trial that are not related to study product or study procedure will end with the last study visit of the participant.

10.9 Participant reimbursement

Participants will not be paid for their participation, but all other expenses related to trial participation will be covered by the study. This will include meals, beverages during their in-patient stay and reimbursement for the transport fare (incurred when coming to BCTF for scheduled or unscheduled visits). As compensation for their time, the participants will be given 15,000 TSH (ca. 6.40 USD) at each visit at the site and 20,000 TSH (ca. 8.58 USD) per day in confinement after the CHMI. An additional 5,000 TSH (ca. 2.13 USD) will be given for transport when participants leave the site. Hence, compensation inclusive of meals, beverages, transport costs and time sum up to about 30,000 TSH (ca 12.86 USD) per site visit and 35,000 TSH (ca 15 USD) per day in confinement at the site ward after the CHMI.

10.10 Protocol amendments

A protocol amendment can be initiated by either the Sponsor or the Investigator. The Investigator will provide the reasons for the proposed amendment in writing and will discuss with the Sponsor. Any protocol amendment must be approved and signed by the Sponsor and the PI and must be submitted to the appropriate IEC for information and approval, in accordance with local requirements, and to RA if required. Approval by IEC (and RA, if applicable) must be received before any changes can be implemented, except for changes necessary to eliminate an immediate hazard to study participants, or when the change involves only logistical or administrative aspects of the study, e.g., change of telephone number(s).

11 QUALITY CONTROL AND QUALITY ASSURANCE: DESCRIPTION OF MEASURES

The PI will ensure that all study personnel are appropriately qualified and trained on all important study related aspects, including a protocol-specific training, GCP training, training on data entry and handling, study interventions and assessments. All applicable documentation is maintained on site.

11.1 Data handling and record keeping / archiving

11.1.1 Case Report Forms

Data recorded during the trial will be captured online in electronic Case Report Forms (eCRF). Access to the eCRF is personalized and secured by user specific passwords. For each participant a record is created that will contain all data as required by the protocol. On the eCRF and other trial specific documents, participants will be identified via a unique participant identifier. The participant identification list will stay under secure control of the site with strict access control processes in place.

Data minimization is applied and only month and year of birth will be collected for participants in the eCRF as this information is critical for the inclusion of participants and for the statistical analysis of the data.

To minimize the data in the eCRF after the baseline assessment (screening visit), only clinically significant out of range observations for vital signs and physical examinations, will be captured as AEs in the eCRF. Other values remain as references in the source document only.

The Investigator is responsible for ensuring that data entries are complete, accurate, legible, and timely and confirms this by physically or electronically signing off the eCRF.

11.1.2 Specification of source documents

Source documents may include participant hospital/ clinic records, physician’s and nurse’s notes, appointment book, original laboratory reports, ECGs, pathology and special assessment reports, signed ICFs, consultant letters, AE logs, and logs of participants screened/randomised. Source data can be electronic or paper-based.

Source data should be attributable, legible, contemporaneous, original, accurate, and complete.

For quality assurance, direct access to the source data and related trial documentation needs to be granted for monitoring, audit and inspection purposes. All involved parties will keep the participant data strictly confidential.

11.1.3 Record keeping/ archiving

The PI must maintain adequate and accurate records to enable the conduct of the study to be fully documented and the study data to be subsequently verified. These records include the Investigator Site File, participant clinical source documents and the eCRF. The Investigator Site File will contain the logs of participants screened and randomised, protocol/protocol amendments, SAE Reporting Forms and query forms, approval from Ethics Committee(s) and RAs with correspondence, sample of the approved study information documents and ICF, completed ICFs, drug accountability records, training and authorization forms, hard copies of eCRFs and queries (if applicable), records of maintenance and/or calibration of equipment, and other appropriate documents/correspondence etc. The PI is responsible for storing the Investigator's Site File and other study documentation in a secure location and to archive them at the end of the clinical study.

The Trial Master File will be kept and maintained by the Sponsor.

The electronic data will be kept for 25 years at Swiss TPH after study completion or premature termination of the clinical trial. At the end of the study, a copy of the data from the eCRF will be provided to the Investigator in an appropriate format, e.g., DVD or flash drive, for long-term archiving. The code list and medical data will be kept in a safe place at Ifakara Health Institute for a period of 20 years after the end of the study.

11.2 Data management

The Sponsor is responsible for data management and will have procedures in place for all relevant data management processes, including but not limited to eCRF development, eCRF validation, data cleaning, user access management and archiving.

11.2.1 Data management system

All relevant participants' data collected during the study period will be recorded in electronic Case Report forms (eCRFs) by a validated EDC tool. The EDC tool maintains a time stamped audit trail that records all user-specific data processing operations such as initial data entry, modification and reasons for change.

11.2.2 Data security, access and back-up

The data in the EDC tool will be stored on a secure server in Switzerland, with a defined policy in place for server set-up, maintenance and security. A backup of the data will be performed automatically on a regular basis. All data captured in the EDC tool, will be encrypted and password secured. Only study team members (as listed in the delegation log/study team log), as well as Sponsor study team, monitors, auditors, ethics committee representatives or RAs will be given access to the study data. Participant data will be handled with utmost discretion and will only be accessible to trained and authorized personnel who require access to the data to fulfil their duties within the scope of the study.

11.2.3 Analysis and archiving

After the last visit of the last participant, final data queries will be resolved, the trial database is locked and the data are extracted from the system for analysis. The data are stored electronically in access restricted folders implemented by the Sponsor.

See section 11.1.3 for more details about data storage and archiving.

11.2.4 Electronic and central data validation

The data will be centrally monitored at Swiss TPH by Data Managers and Monitors to ensure completeness and quality of the data and consistency with the study protocol. Off-site and on-site

monitoring activities will be detailed in the data cleaning and reporting plan and monitoring plan, respectively.

11.3 Risk management

11.3.1 Risk identification, assessment and mitigation

A risk management plan will be set up by the Sponsor. Potential risks to the study, their assessment and management will be documented by the Sponsor in a Risk Management Log. The log will be updated continuously throughout the study duration.

Special attention will be paid to risks associated with participant safety, critical study procedures and critical data.

11.3.2 Study-specific preventive measures

Sentinel dosing

To minimize the risk to healthy participants, the administration of the vaccines will be done sequentially with three participants in the sentinel group as outlined in section 4.

Follow-up post vaccination and CHMI

The participant is discharged 2 hours post vaccination or CHMI, which should allow enough time to assess and take care of any discomfort for the participant appearing directly after vaccination or CHMI. Then the participant is discharged and followed-up daily by phone or home visit (if deemed necessary by the study team) until the next visit at site. With this schedule the participant's time at site can be minimised while at the same time discomfort for the participant can be detected and managed early.

Confinement after CHMI and treatment on Day 28 post CHMI

As outlined in section 7.1.12, at 5 days post CHMI, the participant is requested to be admitted to the study site until considered malaria positive and treated or followed up until day 28 post CHMI (D112). This means the participant is under direct supervision of the study staff when the asexual stage malaria potentially develops. Adequate and prompt malaria treatment will be initiated for participant safety.

By treating all participants latest at 28 days post CHMI (D112) the risk of not treating an undetected malaria infection is minimized.

Birth control and pregnancy testing

Women of child-bearing potential (WOCBP):

WOCBP will only be enrolled after a negative highly sensitive serum pregnancy test. Serum pregnancy tests will be conducted at screening visit 1, during the study before 1st (D0), 2nd (D28) and 3rd (D56) vaccination and before CHMI (D84) and at follow up visits after vaccination (D112 and D140) and CHMI (D168 and D252).

WOCBP must agree to actively use a highly effective form of contraception from 4 weeks before 1st vaccination up to 12 weeks after 3rd vaccination or CHMI. One of the following methods of contraception for WOCBP would be acceptable:

- Injectable progesterone containing hormonal contraception
- Implantable progesterone containing contraception
- Intrauterine device (IUD)
- Intrauterine hormonal releasing system (IUS)
- Bilateral tubal occlusion

Male participants must be willing to ensure the use of condoms from before 1st vaccination up to 12 weeks after 3rd vaccination or CHMI. An adequate number of condoms will be distributed to participants free of charge.

Risks associated with epidemics

The presence of an epidemic/pandemic (including for example COVID-19) can have an impact on the trial, trial participants and staff. Healthy participants may be exposed to infection during screening activities in the communities, or whilst at the investigational site, and there is the risk of trial staff transmitting infection to communities from which participants will be recruited. Special attention is paid to the risk for the participants to be exposed to pathogens while staying at the site. Current Standard Precautions measures to prevent infection will be put in place at the site level.

Mitigation steps to be taken include:

- Prior to initiation of recruitment, and during the trial, an assessment will be made by the Investigator and the Sponsor of the current potential risk associated with each aspect of the trial, based on the incidence of infections in the area of recruitment, the site and national directives. The start of recruitment can be delayed (or paused, if already started) if indicated, or mitigation measures taken to protect staff and participants.
- National and international specific guidelines will be followed during the trial to prevent spread of infection, and protective equipment will be provided. Training will be provided to site staff on standard precautions/infection based precautions.
- Specific risk communication and education activities on national preventive directives on COVID-19 will be implemented for participants as well as site staff.
- The investigational site follows its institutional IHI COVID-19 response plan that provides instructions including guidelines on clinical trials, operations at clinics and hospitals, community and field activities, facemasks and operations inside labs.
- In case travel to and from the site is blocked, the site staff will ensure basic follow-up of participants including supporting access to local medical assistance if required while keeping remote contact with the investigational sites.

Only healthy participants will be included in the trial. After confinement following CHMI, it will be ensured that participants will not be discharged back to their communities unless fit after the confinement. These measures will minimize safety risks to participants during follow-up, if visits were interrupted due to travel restrictions.

11.4 Safety monitoring committee (SMC)

An SMC will be established to ensure the safety of study participants is protected, whilst the scientific goals of the study are being met, according to the protocol and in accordance with international and local standards.

The SMC is an additional measure to complement the Sponsor’s and Investigator’s usual procedures for safety monitoring during clinical trials, aiming an in-depth evaluation on the safety criteria, in accordance with the protocol and with international and local standards.

The SMC will meet for evaluation of the Go/ No-Go criteria as described in section 4.5 and if deemed necessary. The decision to proceed to the next step will be made based on blinded safety and tolerability data. As defined in protocol section 4.5, the study will be halted, and the risk to other participants evaluated if any of the stopping criterion is met.

The composition, roles and responsibilities of the SMC will be described in detail in the SMC charter. The SMC will be comprised of a minimum of three persons, not related directly to the current clinical trial and also include one Tanzanian member.

11.5 Translations - Reference language

The reference language for study documents is English.

Documents used in advertisement and information about the study like the information sheet, ICF etc. used for persons outside the study team will be translated to Swahili.

For translated documents there will be a translation form completed by the person having done the translation and a second person verifying the translation.

For the information sheet, ICF(s) and additional documents deemed necessary by the Sponsor or PI, the reference master document will be in English. In those cases, in addition a third person will be performing and documenting a focused review where the layout and structure of the document will be verified.

11.6 Storage of biological material and related health data

Safety blood samples will be destroyed as soon as the analysis of the study data has been completed.

A subset of samples taken for measuring humoral and cellular responses will be analysed in Basel, Switzerland and Heidelberg, Germany and then stored there. Samples taken for transcriptomic analyses of parasites and the human host during the CHMI will be analysed in Basel, Switzerland and the University of Maryland, United States of America. Samples collected for in depth analysis of B-cell responses after vaccination will be analysed in Milan, Italy.

Exploratory samples and source data will be stored and archived at the BRTC. The sampling that can be used for further malaria research or health related research as well as the samples sent to Switzerland, USA, Italy and Germany will be mentioned in the information sheet or ICF(s) and explained to the participants.

All samples stored will be labelled with the participants ID and identifiers that do not allow to identify the study participant directly.

The data obtained in this study may provide the basis for further studies using samples stored for exploratory purposes. For those future evaluations the necessary approvals are required before sharing of the samples is possible.

12 FUNDING

This project is funded by Sumaya Biotech GmBH & Co. KG, Heidelberg, through a Venture Loan Agreement with EMF EEU Malaria Fund Berlin GmbH & Co. KG.

The Sponsor, the PI and supporting organizations/institutions declare to have no conflict of interest.

The Investigators, including the PI and/or any Sub-Investigators, directly involved in the treatment or evaluation of participants may be requested to provide a financial disclosure. All relevant documentation will be filed in the Trial Master File.

13 DISSEMINATION OF RESULTS AND PUBLICATION POLICY

All data and results generated in the study will be owned by the industrial Sponsor, Sumaya Biotech GmBH & Co. KG, who may utilize them in various ways, such as for submission to the RAs.

Swiss TPH as sponsor will make the data derived from the study freely available publicly through appropriate open access databases, repositories and similar tools.

The Investigators will be involved in writing and/or reviewing drafts of the manuscripts, abstracts, press releases and any other publications arising from the study. Apart from obvious flaws to the conduct of the study, which may preclude data publication, safety and efficacy data will be published under the supervision and authorization of the Sponsor in appropriate peer-reviewed journal(s).

Authorship for publication in scientific journals will be offered to each party that has substantially contributed to the work reported in the publication. It is expected that authorship of such publications will be led by the Sponsor in close collaboration with Sumaya Biotech. The study team must seek permission from NatHREC before publishing any manuscript as per local regulations.

The interim and final clinical study report will be the responsibility of the Sponsor and will be submitted to the RAs and ethics committee(s), if required by these bodies (see sections 10.1 and 10.2) when available.

Data from the study may also be used as part of a thesis for a PhD, MD or Masters. Publications arising from this study will be made open access.

The Sponsor encourages the communication and/ or publication of the results, in accordance with the terms of the Master Service Agreement with the Industrial Sponsor Clinical Trial Agreement.

14 REFERENCES

1. Ashley, E.A., A. Pyae Phyo, and C.J. Woodrow, *Malaria.* Lancet, 2018. **391**(10130): p. 1608-1621.

2. *World malaria report 2021.* Geneva: World Health Organization; 2021., 2021. **Licence: CC BY-NC-SA 3.0 IGO**.

3. Phillips, M.A., et al., *Malaria.* Nat Rev Dis Primers, 2017. **3**: p. 17050.

4. Cowman, A.F., et al., *The Molecular Basis of Erythrocyte Invasion by Malaria Parasites.* Cell Host Microbe, 2017. **22**(2): p. 232-245.

5. Raj, D.K., et al., *Antibodies to PfSEA-1 block parasite egress from RBCs and protect against malaria infection.* Science, 2014. **344**(6186): p. 871-7.

6. Singh, S. and C.E. Chitnis, *Molecular Signaling Involved in Entry and Exit of Malaria Parasites from Host Erythrocytes.* Cold Spring Harb Perspect Med, 2017. **7**(10).

7. Gilson, P.R., et al., *Identification and stoichiometry of glycosylphosphatidylinositol-anchored membrane proteins of the human malaria parasite Plasmodium falciparum.* Mol Cell Proteomics, 2006. **5**(7): p. 1286-99.

8. Dijkman, P.M., et al., *Structure of the merozoite surface protein 1 from Plasmodium falciparum.* Sci Adv, 2021. **7**(23).

9. Holder, A.A., et al., *A malaria merozoite surface protein (MSP1)-structure, processing and function.* Mem Inst Oswaldo Cruz, 1992. **87 Suppl 3**: p. 37-42.

10. Lin, C.S., et al., *The merozoite surface protein 1 complex is a platform for binding to human erythrocytes by Plasmodium falciparum.* J Biol Chem, 2014. **289**(37): p. 25655-69.

11. O'Donnell, R.A., et al., *Functional conservation of the malaria vaccine antigen MSP-119across distantly related Plasmodium species.* Nat Med, 2000. **6**(1): p. 91-5.

12. Herrera, S., et al., *A conserved region of the MSP-1 surface protein of Plasmodium falciparum contains a recognition sequence for erythrocyte spectrin.* EMBO J, 1993. **12**(4): p. 1607-14.

13. Baldwin, M.R., et al., *Merozoite surface protein 1 recognition of host glycophorin A mediates malaria parasite invasion of red blood cells.* Blood, 2015. **125**(17): p. 2704-11.

14. Das, S., et al., *Processing of Plasmodium falciparum Merozoite Surface Protein MSP1 Activates a Spectrin-Binding Function Enabling Parasite Egress from RBCs.* Cell Host Microbe, 2015. **18**(4): p. 433-44.

15. Wilson, K.L., et al., *Malaria vaccines in the eradication era: current status and future perspectives.* Expert Rev Vaccines, 2019. **18**(2): p. 133-151.

16. Zavala, F., *RTS,S: the first malaria vaccine.* J Clin Invest, 2022. **132**(1).

17. Witte, D., et al., *Safety and Immunogenicity of Seven Dosing Regimens of the Candidate RTS,S/AS01E Malaria Vaccine Integrated Within an Expanded Program on Immunization Regimen: A Phase II, Single-Center, Open, Controlled Trial in Infants in Malawi.* Pediatr Infect Dis J, 2018. **37**(5): p. 483-491.

18. Rampling, T., et al., *Safety and efficacy of novel malaria vaccine regimens of RTS,S/AS01B alone, or with concomitant ChAd63-MVA-vectored vaccines expressing ME-TRAP.* NPJ Vaccines, 2018. **3**: p. 49.

19. *WHO recommends groundbreaking malaria vaccine for children at risk.* WHO news release.

20. Rts, S.C.T.P., *Efficacy and safety of RTS,S/AS01 malaria vaccine with or without a booster dose in infants and children in Africa: final results of a phase 3, individually randomised, controlled trial.* Lancet, 2015. **386**(9988): p. 31-45.

21. *World Health Organization. Malaria vaccine: WHO position paper.* Weekly Epidemiol Rec, 2016. **91**: p. 33–52

22. Marsh, K. and S. Kinyanjui, *Immune effector mechanisms in malaria.* Parasite Immunol, 2006. **28**(1-2): p. 51-60.

23. Miura, K., *Progress and prospects for blood-stage malaria vaccines.* Expert Rev Vaccines, 2016. **15**(6): p. 765-81.

24. Boyle, M.J., et al., *Human antibodies fix complement to inhibit Plasmodium falciparum invasion of erythrocytes and are associated with protection against malaria.* Immunity, 2015. **42**(3): p. 580-90.

25. Burns, A.L., et al., *Targeting malaria parasite invasion of red blood cells as an antimalarial strategy.* FEMS Microbiol Rev, 2019. **43**(3): p. 223-238.

26. Singh, S., et al., *Immunity to recombinant plasmodium falciparum merozoite surface protein 1 (MSP1): protection in Aotus nancymai monkeys strongly correlates with anti-MSP1 antibody titer and in vitro parasite-inhibitory activity.* Infect Immun, 2006. **74**(8): p. 4573-80.

27. Malkin, E., et al., *Phase 1 study of two merozoite surface protein 1 (MSP1(42)) vaccines for Plasmodium falciparum malaria.* PLoS Clin Trials, 2007. **2**(4): p. e12.

28. Thera, M.A., et al., *Safety and allele-specific immunogenicity of a malaria vaccine in Malian adults: results of a phase I randomized trial.* PLoS Clin Trials, 2006. **1**(7): p. e34.

29. Ogutu, B.R., et al., *Blood stage malaria vaccine eliciting high antigen-specific antibody concentrations confers no protection to young children in Western Kenya.* PLoS One, 2009. **4**(3): p. e4708.

30. Woehlbier, U., et al., *Antibodies against multiple merozoite surface antigens of the human malaria parasite Plasmodium falciparum inhibit parasite maturation and red blood cell invasion.* Malar J, 2010. **9**: p. 77.

31. Blank, A., et al., *Immunization with full-length Plasmodium falciparum merozoite surface protein 1 is safe and elicits functional cytophilic antibodies in a randomized first-in-human trial.* NPJ Vaccines, 2020. **5**(1): p. 10.

32. Kauth, C.W., et al., *The merozoite surface protein 1 complex of human malaria parasite Plasmodium falciparum: interactions and arrangements of subunits.* J Biol Chem, 2003. **278**(25): p. 22257-64.

33. *GUIDELINE ON CLINICAL EVALUATION OF NEW VACCINES.* EMEA/CHMP/VWP/164653/2005, 2005.

34. Cockburn, I.A. and R.A. Seder, *Malaria prevention: from immunological concepts to effective vaccines and protective antibodies.* Nat Immunol, 2018. **19**(11): p. 1199-1211.

35. Toxicology, L.o.P.a., *Report: Examination of an eluate of MSP-1 on pyrogenic properties in rabbits- according to EP 2.6.8.* 2016.

36. Cook, I.F., *Best vaccination practice and medically attended injection site events following deltoid intramuscular injection.* Hum Vaccin Immunother, 2015. **11**(5): p. 1184-91.

37. Shekalaghe, S., et al., *Controlled human malaria infection of Tanzanians by intradermal injection of aseptic, purified, cryopreserved Plasmodium falciparum sporozoites.* Am J Trop Med Hyg, 2014. **91**(3): p. 471-480.

38. Church, L.W., et al., *Clinical manifestations of Plasmodium falciparum malaria experimentally induced by mosquito challenge.* J Infect Dis, 1997. **175**(4): p. 915-20.

39. Roestenberg, M., et al., *Comparison of clinical and parasitological data from controlled human malaria infection trials.* PLoS One, 2012. **7**(6): p. e38434.

40. Laurens, M.B., et al., *A consultation on the optimization of controlled human malaria infection by mosquito bite for evaluation of candidate malaria vaccines.* Vaccine, 2012. **30**(36): p. 5302-4.

41. Chulay, J.D., et al., *Malaria transmitted to humans by mosquitoes infected from cultured Plasmodium falciparum.* Am J Trop Med Hyg, 1986. **35**(1): p. 66-8.

42. Epstein, J.E., et al., *Safety and clinical outcome of experimental challenge of human volunteers with Plasmodium falciparum-infected mosquitoes: an update.* J Infect Dis, 2007. **196**(1): p. 145-54.

43. Costy-Berger, F., *[Preventive rabies vaccination using vaccine prepared from human diploid cells].* Dev Biol Stand, 1978. **40**: p. 101-4.

44. Ajjan, N., et al., *[Results of preventive rabies vaccination with a concentrated vaccine of the PM/WI38-1503-3M rabies strain cultured on human diploid cells. Preparation of mixed antirabies-antitetanus hyperimmune immunoglobulin by plasmapheresis of blood taken from vaccinated veterinary students].* Dev Biol Stand, 1978. **40**: p. 89-100.

45. Nkurunungi, G., et al., *Population differences in vaccine responses (POPVAC): scientific rationale and cross-cutting analyses for three linked, randomised controlled trials assessing the role, reversibility and mediators of immunomodulation by chronic infections in the tropics.* BMJ Open, 2021. **11**(2): p. e040425.

46. Stelekati, E. and E.J. Wherry, *Chronic bystander infections and immunity to unrelated antigens.* Cell Host Microbe, 2012. **12**(4): p. 458-69.

47. Nouatin, O., et al., *Exploratory analysis of the effect of helminth infection on the immunogenicity and efficacy of the asexual blood-stage malaria vaccine candidate GMZ2.* PLoS Negl Trop Dis, 2021. **15**(6): p. e0009361.

48. Kabagenyi, J., et al., *Urban-rural differences in immune responses to mycobacterial and tetanus vaccine antigens in a tropical setting: A role for helminths?* Parasitol Int, 2020. **78**: p. 102132.

49. Jongo, S.K., K. Abdul, U, *BRTC_RITox_001_V3.1. Manual of Reference Intervals and Grading of Selected Abnormal Values.* 2022.

50. *Toxicity Grading Scale for Healthy Adult and Adolescent Volunteers Enrolled in Preventive Vaccine Clinical Trials.* U.S. Department of Health and Human Services, Food and Drug Administration, Center for Biologics Evaluation and Research 2007.

51. *ICH E9 statistical principles for clinical trials. .* European Medicines Agency., 1998.

52. Schulz, K.F., et al., *CONSORT 2010 statement: updated guidelines for reporting parallel group randomised trials.* BMJ, 2010. **340**: p. c332.

15 APPENDICES

15.1 Laboratories analysing biological materials

**Institution**	Swiss Tropical and Public Health Institute
**Laboratory**	Department of Medical Parasitology and Infection Biology<br>Clinical Immunology Unit
**Address**	Kreuzstrasse 2<br>4123 Allschwil, Switzerland
**Contact person**	Prof Claudia Daubenberger<br>claudia.daubenberger@swisstph.ch

**Institution**	Sumaya Biotech GmbH & Co. KG
**Laboratory**	Sumaya Biotech GmbH & Co. KG
**Address**	Vangerowstrasse 20<br>69115 Heidelberg, Germany
**Contact person**	Dr. Andrea Aschenbrenner<br>aschenbrenner@sumaya-biotech.com

**Institution**	University of Heidelberg
**Laboratory**	Centre for Infectious Diseases, Parasitology
**Address**	Im Neuenheimer Feld 324<br>69120 Heidelberg, Germany
**Contact person**	Prof. Michael Lanzer<br>Michael.Lanzer@med.uni-heidelberg.de

**Institution**	Ifakara Health Institute
**Laboratory**	Bagamoyo Research and Training Center
**Address**	P.O. Box 74,<br>Bagamoyo, Tanzania
**Contact person**	Dr. Ally Olotu<br>aolotu@ihi.or.tz

**Institution**	Fondazione Istituto Nazionale Genetica Molecolare – INGM
**Laboratory**	Padiglione “Romeo ed Enrica Invernizzi”
**Address**	Fondazione IRCCS Cà Granda Ospedale Maggiore Policlinico di Milano<br>Via Francesco Sforza 35, 20122 Milan, Italy
**Contact person**	Prof. Antonio Lanzavecchia<br>lanzavecchia@ingm.org

**Institution**	University of Maryland School of Medicine
**Laboratory**	Department of Microbiology and Immunology<br>Health Sciences Facility III
**Address**	670 West Baltimore St<br>21201 Baltimore, Maryland, USA
**Contact person**	Prof. Joana da Silva<br>jcsilva@som.umaryland.edu

**Institution**	German Cancer Research Center (DKFZ)
**Laboratory**	16 Division of B cell Immunology
**Address**	Im Neuenheimer Feld 280<br>69120 Heidelberg, Germany
**Contact person**	Prof. Dr. Hedda Wadermann<br>h.wardemann@dkfz.de

**Institution**	Universidad Complutense de Madrid
**Laboratory**	Department of Biochemistry and Molecular Biology, Veterinary Faculty
**Address**	Avda. Puerta del Hierro s/n<br>Ciudad Universitaria. 28040, Madrid, Spain
**Contact person**	Prof. Jose Manuel Bautista Santa Cruz<br>jmbau@ucm.es

**Institution**	Max Delbrück Center for Molecular Medicine
**Laboratory**	Dr. Misha Kudryashev
**Address**	Robert-Rössle-Strasse 10<br>Building 31.2, room 0207<br>13092 Berlin, Germany
**Contact person**	Dr. Misha Kudryashev<br>mikhail.kudryashev@mdc-berlin.de

**Institution**	Pepperprint GmbH
**Laboratory**	Pepperprint GmbH
**Address**	Rischerstrasse 12<br>69123 Heidelberg, Germany
**Contact person**	Dr. Volker Stadler<br>volker.stadler@pepperprint.com

**Institution**	Glyxera GmbH/Max Planck Institute for Dynamics of Complex Technical Systems
**Laboratory**	Dr. Erdmann Rapp
**Address**	Brenneckestrasse 20<br>ZENIT building<br>39120 Magdeburg, Germany
**Contact person**	Dr. Erdmann Rapp<br>e.rapp@glyxera.com

DocuSign

Certificate Of Completion

Envelope Id: 95E51E3C369D413DA65D92A195943639 | Status: Completed

Subject: MSP-1 3 corrected / Please DocuSign: Clinical Trial Protocol_MSP1_v1.1_12.09.2022

Source Envelope:

Document Pages: 70	Signatures: 5	Envelope Originator:
Certificate Pages: 6	Initials: 0	Mischa Joubert
AutoNav: Enabled		Kreuzstrasse 2,
EnvelopeId Stamping: Disabled		Allschwil, . 4123
Time Zone: (UTC+01:00) Brussels, Copenhagen, Madrid, Paris		mischa.joubert@swisstph.ch
		IP Address: 131.152.225.40

Record Tracking

Status: Original<br>16-Sep-2022 | 07:38	Holder: Mischa Joubert<br>mischa.joubert@swisstph.ch	Location: DocuSign

Signer Events

Signer Events	Signature	Timestamp
Claudia Daubenberger<br>claudia.daubenberger@swisstph.ch<br>Security Level: Email, Account Authentication (Required)	Claudia Daubenberger<br><br>Signature Adoption: Pre-selected Style<br>Signature ID:<br>570FB241-D0EF-4659-B902-859A8C59854D<br>Using IP Address: 46.253.189.66<br><br>With Signing Authentication via DocuSign password<br>With Signing Reasons (on each tab):<br>I approve this document	Sent: 16-Sep-2022 | 07:47<br>Viewed: 16-Sep-2022 | 09:18<br>Signed: 16-Sep-2022 | 09:18

Electronic Record and Signature Disclosure:
Accepted: 19-Aug-2021 | 16:14
ID: f7be9a29-9ecd-4191-8afc-7e294adc1fb1

Signer Events	Signature	Timestamp
Daniel Paris<br>daniel.paris@swisstph.ch<br>Medical Director and Head, Dept. of Medicine<br>Swiss TPH<br>Security Level: Email, Account Authentication (Required)	Daniel Paris<br><br>Signature Adoption: Pre-selected Style<br>Signature ID:<br>608447B2-DF75-4BD0-B115-E05CD1DA9875<br>Using IP Address: 131.152.225.40<br><br>With Signing Authentication via DocuSign password<br>With Signing Reasons (on each tab):<br>I approve this document	Sent: 16-Sep-2022 | 07:47<br>Viewed: 16-Sep-2022 | 08:50<br>Signed: 16-Sep-2022 | 09:04

Electronic Record and Signature Disclosure:
Accepted: 09-Nov-2017 | 08:45
ID: 211fcdff-1764-4c34-bfce-0c930be0616b

Signer Events	Signature	Timestamp
Eric Huber<br>eric.huber@swisstph.ch<br>Project Leader<br>Swiss TPH<br>Security Level: Email, Account Authentication (Required)	Signature Adoption: Uploaded Signature Image<br>Signature ID:<br>8E49A93E-E109-4663-965A-1E67D747FA3F<br>Using IP Address: 131.152.225.40<br><br>With Signing Authentication via DocuSign password<br>With Signing Reasons (on each tab):<br>I approve this document	Sent: 16-Sep-2022 | 07:47<br>Viewed: 16-Sep-2022 | 08:07<br>Signed: 16-Sep-2022 | 08:07
**Electronic Record and Signature Disclosure:**<br>Accepted: 10-Jul-2018 | 14:17<br>ID: 6a234b9d-aa93-4ff2-af51-a0b11fd200dc		
Ernst Böhnlein<br>boehnlein@sumaya-biotech.com<br>Security Level: Email, Account Authentication (Required)	Signature Adoption: Pre-selected Style<br>Signature ID:<br>4829AB79-E23C-41EE-9467-89C0866152F0<br>Using IP Address: 81.89.201.157<br><br>With Signing Authentication via DocuSign password<br>With Signing Reasons (on each tab):<br>Ich genehmige dieses Dokument	Sent: 16-Sep-2022 | 07:47<br>Viewed: 16-Sep-2022 | 10:31<br>Signed: 16-Sep-2022 | 10:32
**Electronic Record and Signature Disclosure:**<br>Accepted: 15-Feb-2022 | 14:55<br>ID: d6109176-6664-4fa2-a686-be32e82b94db		
Fiona Vanobberghen<br>fiona.vanobberghen@swisstph.ch<br>Security Level: Email, Account Authentication (Required)	Signature Adoption: Pre-selected Style<br>Signature ID:<br>99EE8064-61C7-40FC-BC3B-84712F5365C8<br>Using IP Address: 131.152.225.37<br><br>With Signing Authentication via DocuSign password<br>With Signing Reasons (on each tab):<br>I have reviewed this document	Sent: 16-Sep-2022 | 07:47<br>Viewed: 19-Sep-2022 | 09:09<br>Signed: 19-Sep-2022 | 09:10
**Electronic Record and Signature Disclosure:**<br>Accepted: 03-Apr-2020 | 10:50<br>ID: f82a2752-b8bd-4e07-854e-f680e88a1249		

In Person Signer Events	Signature	Timestamp

Editor Delivery Events	Status	Timestamp

Agent Delivery Events	Status	Timestamp

Intermediary Delivery Events	Status	Timestamp

Certified Delivery Events	Status	Timestamp

Carbon Copy Events	Status	Timestamp

Carbon Copy Events	Status	Timestamp
Meera Saxena<br>meera.saxena@swisstph.ch<br>Clinical Research Associate<br>Swiss TPH<br>Security Level: Email, Account Authentication (Required)<br>**Electronic Record and Signature Disclosure:**<br>Accepted: 19-Aug-2022 | 10:38<br>ID: b120d76e-8134-45cd-b2ce-1af4b5ddcbc6	COPIED	Sent: 16-Sep-2022 | 07:47<br>Viewed: 19-Sep-2022 | 09:20

Witness Events	Signature	Timestamp

Notary Events	Signature	Timestamp

Envelope Summary Events	Status	Timestamps
Envelope Sent	Hashed/Encrypted	16-Sep-2022 | 07:47
Certified Delivered	Security Checked	19-Sep-2022 | 09:09
Signing Complete	Security Checked	19-Sep-2022 | 09:10
Completed	Security Checked	19-Sep-2022 | 09:10

Payment Events	Status	Timestamps

Electronic Record and Signature Disclosure

CONSUMER DISCLOSURE

From time to time, Schweizerisches Tropen- und Public Health-Institut (we, us or Company) may be required by law to provide to you certain written notices or disclosures. Described below are the terms and conditions for providing to you such notices and disclosures electronically through the DocuSign, Inc. (DocuSign) electronic signing system. Please read the information below carefully and thoroughly, and if you can access this information electronically to your satisfaction and agree to these terms and conditions, please confirm your agreement by clicking the â€˜I agreeâ€™ button at the bottom of this document.

Getting paper copies

At any time, you may request from us a paper copy of any record provided or made available electronically to you by us. You will have the ability to download and print documents we send to you through the DocuSign system during and immediately after signing session and, if you elect to create a DocuSign signer account, you may access them for a limited period of time (usually 30 days) after such documents are first sent to you. After such time, if you wish for us to send you paper copies of any such documents from our office to you, you will be charged a $0.00 per-page fee. You may request delivery of such paper copies from us by following the procedure described below.

Withdrawing your consent

If you decide to receive notices and disclosures from us electronically, you may at any time change your mind and tell us that thereafter you want to receive required notices and disclosures only in paper format. How you must inform us of your decision to receive future notices and disclosure in paper format and withdraw your consent to receive notices and disclosures electronically is described below.

Consequences of changing your mind

If you elect to receive required notices and disclosures only in paper format, it will slow the speed at which we can complete certain steps in transactions with you and delivering services to you because we will need first to send the required notices or disclosures to you in paper format, and then wait until we receive back from you your acknowledgment of your receipt of such paper notices or disclosures. To indicate to us that you are changing your mind, you must withdraw your consent using the DocuSign â€˜Withdraw Consentâ€™ form on the signing page of a DocuSign envelope instead of signing it. This will indicate to us that you have withdrawn your consent to receive required notices and disclosures electronically from us and you will no longer be able to use the DocuSign system to receive required notices and consents electronically from us or to sign electronically documents from us.

All notices and disclosures will be sent to you electronically

Unless you tell us otherwise in accordance with the procedures described herein, we will provide electronically to you through the DocuSign system all required notices, disclosures, authorizations, acknowledgements, and other documents that are required to be provided or made available to you during the course of our relationship with you. To reduce the chance of you inadvertently not receiving any notice or disclosure, we prefer to provide all of the required notices and disclosures to you by the same method and to the same address that you have given us. Thus, you can receive all the disclosures and notices electronically or in paper format through the paper mail delivery system. If you do not agree with this process, please let us know as described below. Please also see the paragraph immediately above that describes the consequences of your electing not to receive delivery of the notices and disclosures

electronically from us.

How to contact Schweizerisches Tropen- und Public Health-Institut:

You may contact us to let us know of your changes as to how we may contact you electronically, to request paper copies of certain information from us, and to withdraw your prior consent to receive notices and disclosures electronically as follows:

To contact us by email send messages to: andre.lederer@unibas.ch

To advise Schweizerisches Tropen- und Public Health-Institut of your new e-mail address

To let us know of a change in your e-mail address where we should send notices and disclosures electronically to you, you must send an email message to us at andre.lederer@unibas.ch and in the body of such request you must state: your previous e-mail address, your new e-mail address. We do not require any other information from you to change your email address..

In addition, you must notify DocuSign, Inc. to arrange for your new email address to be reflected in your DocuSign account by following the process for changing e-mail in the DocuSign system.

To request paper copies from Schweizerisches Tropen- und Public Health-Institut

To request delivery from us of paper copies of the notices and disclosures previously provided by us to you electronically, you must send us an e-mail to andre.lederer@unibas.ch and in the body of such request you must state your e-mail address, full name, US Postal address, and telephone number. We will bill you for any fees at that time, if any.

To withdraw your consent with Schweizerisches Tropen- und Public Health-Institut

To inform us that you no longer want to receive future notices and disclosures in electronic format you may:

i. decline to sign a document from within your DocuSign session, and on the subsequent page, select the check-box indicating you wish to withdraw your consent, or you may;

ii. send us an e-mail to andre.lederer@unibas.ch and in the body of such request you must state your e-mail, full name, US Postal Address, and telephone number. We do not need any other information from you to withdraw consent.. The consequences of your withdrawing consent for online documents will be that transactions may take a longer time to process..

Required hardware and software

Operating Systems:	WindowsÂ® 2000, WindowsÂ® XP, Windows VistaÂ®; Mac OSÂ® X
Browsers:	Final release versions of Internet ExplorerÂ® 6.0 or above (Windows only); Mozilla Firefox 2.0 or above (Windows and Mac); Safariâ„¢ 3.0 or above (Mac only)
PDF Reader:	AcrobatÂ® or similar software may be required to view and print PDF files
Screen Resolution:	800 x 600 minimum
Enabled Security Settings:	Allow per session cookies

** These minimum requirements are subject to change. If these requirements change, you will be asked to re-accept the disclosure. Pre-release (e.g. beta) versions of operating systems and browsers are not supported.

Acknowledging your access and consent to receive materials electronically

To confirm to us that you can access this information electronically, which will be similar to other electronic notices and disclosures that we will provide to you, please verify that you were able to read this electronic disclosure and that you also were able to print on paper or electronically save this page for your future reference and access or that you were able to e-mail this disclosure and consent to an address where you will be able to print on paper or save it for your future reference and access. Further, if you consent to receiving notices and disclosures exclusively in electronic format on the terms and conditions described above, please let us know by clicking the â€˜I agreeâ€™ button below.
By checking the â€˜I agreeâ€™ box, I confirm that:

- I can access and read this Electronic CONSENT TO ELECTRONIC RECEIPT OF ELECTRONIC CONSUMER DISCLOSURES document; and

- I can print on paper the disclosure or save or send the disclosure to a place where I can print it, for future reference and access; and

- Until or unless I notify Schweizerisches Tropen- und Public Health-Institut as described above, I consent to receive from exclusively through electronic means all notices, disclosures, authorizations, acknowledgements, and other documents that are required to be provided or made available to me by Schweizerisches Tropen- und Public Health-Institut during the course of my relationship with you.

DocuSign

Certificate Of Completion

Envelope Id: 4204C08E0FBF4FD480EB3DAA2A6820E3		Status: Completed
Subject: MSP 1 03 / Please DocuSign: Clinical Trial Protocol_MSP1_v1.1_12.09.2022, part signed.pdf		
Source Envelope:		
Document Pages: 76	Signatures: 1	Envelope Originator:
Certificate Pages: 5	Initials: 0	Mischa Joubert
AutoNav: Enabled		Kreuzstrasse 2,
EnvelopeId Stamping: Disabled		Allschwil, . 4123
Time Zone: (UTC+01:00) Brussels, Copenhagen, Madrid, Paris		mischa.joubert@swisstph.ch
		IP Address: 131.152.225.40

Record Tracking

Status: Original<br>21-Sep-2022 | 08:09	Holder: Mischa Joubert<br>mischa.joubert@swisstph.ch	Location: DocuSign

Signer Events	Signature	Timestamp
Ally Olotu<br>aolotu@ihi.or.tz<br>Principal Investigator<br>Ifakara Health Institute<br>Security Level: Email, Account Authentication (Required)	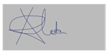<br>Signature Adoption: Uploaded Signature Image<br>Signature ID:<br>5BE062C2-235F-4CA5-803E-77C4D831A97E<br>Using IP Address: 197.250.15.81<br><br>With Signing Authentication via DocuSign password<br>With Signing Reasons (on each tab):<br>I approve this document	Sent: 21-Sep-2022 | 08:14<br>Viewed: 21-Sep-2022 | 09:43<br>Signed: 21-Sep-2022 | 09:44

Electronic Record and Signature Disclosure:
Accepted: 13-Feb-2022 | 18:57
ID: 8c92bce7-3c46-4c02-9ded-47b6703943cd

In Person Signer Events	Signature	Timestamp

Editor Delivery Events	Status	Timestamp

Agent Delivery Events	Status	Timestamp

Intermediary Delivery Events	Status	Timestamp

Certified Delivery Events	Status	Timestamp

Carbon Copy Events	Status	Timestamp
Meera Saxena<br>meera.saxena@swisstph.ch<br>Clinical Research Associate<br>Swiss TPH<br>Security Level: Email, Account Authentication (Required)	COPIED	Sent: 21-Sep-2022 | 08:14

Electronic Record and Signature Disclosure:
Accepted: 19-Aug-2022 | 10:38
ID: b120d76e-8134-45cd-b2ce-1af4b5ddcbc6

Witness Events	Signature	Timestamp

Notary Events	Signature	Timestamp

Envelope Summary Events	Status	Timestamps
Envelope Sent	Hashed/Encrypted	21-Sep-2022 | 08:14
Certified Delivered	Security Checked	21-Sep-2022 | 09:43
Signing Complete	Security Checked	21-Sep-2022 | 09:44
Completed	Security Checked	21-Sep-2022 | 09:44

Payment Events	Status	Timestamps

Electronic Record and Signature Disclosure

CONSUMER DISCLOSURE

From time to time, Schweizerisches Tropen- und Public Health-Institut (we, us or Company) may be required by law to provide to you certain written notices or disclosures. Described below are the terms and conditions for providing to you such notices and disclosures electronically through the DocuSign, Inc. (DocuSign) electronic signing system. Please read the information below carefully and thoroughly, and if you can access this information electronically to your satisfaction and agree to these terms and conditions, please confirm your agreement by clicking the â€˜I agreeâ€™ button at the bottom of this document.

Getting paper copies

At any time, you may request from us a paper copy of any record provided or made available electronically to you by us. You will have the ability to download and print documents we send to you through the DocuSign system during and immediately after signing session and, if you elect to create a DocuSign signer account, you may access them for a limited period of time (usually 30 days) after such documents are first sent to you. After such time, if you wish for us to send you paper copies of any such documents from our office to you, you will be charged a $0.00 per-page fee. You may request delivery of such paper copies from us by following the procedure described below.

Withdrawing your consent

If you decide to receive notices and disclosures from us electronically, you may at any time change your mind and tell us that thereafter you want to receive required notices and disclosures only in paper format. How you must inform us of your decision to receive future notices and disclosure in paper format and withdraw your consent to receive notices and disclosures electronically is described below.

Consequences of changing your mind

If you elect to receive required notices and disclosures only in paper format, it will slow the speed at which we can complete certain steps in transactions with you and delivering services to you because we will need first to send the required notices or disclosures to you in paper format, and then wait until we receive back from you your acknowledgment of your receipt of such paper notices or disclosures. To indicate to us that you are changing your mind, you must withdraw your consent using the DocuSign â€˜Withdraw Consentâ€™ form on the signing page of a DocuSign envelope instead of signing it. This will indicate to us that you have withdrawn your consent to receive required notices and disclosures electronically from us and you will no longer be able to use the DocuSign system to receive required notices and consents electronically from us or to sign electronically documents from us.

All notices and disclosures will be sent to you electronically

Unless you tell us otherwise in accordance with the procedures described herein, we will provide electronically to you through the DocuSign system all required notices, disclosures, authorizations, acknowledgements, and other documents that are required to be provided or made available to you during the course of our relationship with you. To reduce the chance of you inadvertently not receiving any notice or disclosure, we prefer to provide all of the required notices and disclosures to you by the same method and to the same address that you have given us. Thus, you can receive all the disclosures and notices electronically or in paper format through the paper mail delivery system. If you do not agree with this process, please let us know as described below. Please also see the paragraph immediately above that describes the consequences of your electing not to receive delivery of the notices and disclosures

electronically from us.
How to contact Schweizerisches Tropen- und Public Health-Institut:
You may contact us to let us know of your changes as to how we may contact you electronically, to request paper copies of certain information from us, and to withdraw your prior consent to receive notices and disclosures electronically as follows:
To contact us by email send messages to: andre.lederer@unibas.ch

To advise Schweizerisches Tropen- und Public Health-Institut of your new e-mail address
To let us know of a change in your e-mail address where we should send notices and disclosures electronically to you, you must send an email message to us at andre.lederer@unibas.ch and in the body of such request you must state: your previous e-mail address, your new e-mail address. We do not require any other information from you to change your email address..
In addition, you must notify DocuSign, Inc. to arrange for your new email address to be reflected in your DocuSign account by following the process for changing e-mail in the DocuSign system.
To request paper copies from Schweizerisches Tropen- und Public Health-Institut
To request delivery from us of paper copies of the notices and disclosures previously provided by us to you electronically, you must send us an e-mail to andre.lederer@unibas.ch and in the body of such request you must state your e-mail address, full name, US Postal address, and telephone number. We will bill you for any fees at that time, if any.
To withdraw your consent with Schweizerisches Tropen- und Public Health-Institut
To inform us that you no longer want to receive future notices and disclosures in electronic format you may:

i. decline to sign a document from within your DocuSign session, and on the subsequent page, select the check-box indicating you wish to withdraw your consent, or you may;
ii. send us an e-mail to andre.lederer@unibas.ch and in the body of such request you must state your e-mail, full name, US Postal Address, and telephone number. We do not need any other information from you to withdraw consent.. The consequences of your withdrawing consent for online documents will be that transactions may take a longer time to process..

Required hardware and software

Operating Systems:	WindowsÂ® 2000, WindowsÂ® XP, Windows VistaÂ®; Mac OSÂ® X
Browsers:	Final release versions of Internet ExplorerÂ® 6.0 or above (Windows only); Mozilla Firefox 2.0 or above (Windows and Mac); Safariâ„¢ 3.0 or above (Mac only)
PDF Reader:	AcrobatÂ® or similar software may be required to view and print PDF files
Screen Resolution:	800 x 600 minimum
Enabled Security Settings:	Allow per session cookies

** These minimum requirements are subject to change. If these requirements change, you will be asked to re-accept the disclosure. Pre-release (e.g. beta) versions of operating systems and browsers are not supported.
Acknowledging your access and consent to receive materials electronically

To confirm to us that you can access this information electronically, which will be similar to other electronic notices and disclosures that we will provide to you, please verify that you were able to read this electronic disclosure and that you also were able to print on paper or electronically save this page for your future reference and access or that you were able to e-mail this disclosure and consent to an address where you will be able to print on paper or save it for your future reference and access. Further, if you consent to receiving notices and disclosures exclusively in electronic format on the terms and conditions described above, please let us know by clicking the â€˜I agreeâ€™ button below.
By checking the â€˜I agreeâ€™ box, I confirm that:

- I can access and read this Electronic CONSENT TO ELECTRONIC RECEIPT OF ELECTRONIC CONSUMER DISCLOSURES document; and

- I can print on paper the disclosure or save or send the disclosure to a place where I can print it, for future reference and access; and

- Until or unless I notify Schweizerisches Tropen- und Public Health-Institut as described above, I consent to receive from exclusively through electronic means all notices, disclosures, authorizations, acknowledgements, and other documents that are required to be provided or made available to me by Schweizerisches Tropen- und Public Health-Institut during the course of my relationship with you.

Clinical Study Protocol

“A randomised, controlled, double-blind, parallel group, single center Phase Ib trial to assess safety, reactogenicity and immunogenicity of a candidate dual-stage malaria vaccine, SumayaVac-1 (MSP-1 with GLA-SE as adjuvant) in healthy malaria exposed adults of African origin aged 18-45 years”

**Protocol Number**	**P2267-22**		
**Version Number**	2.0	**Document Date**	30.11.2023
**Study Registration**	Clinicaltrials.gov (NCT05644067)<br>TMDA registry (TRC-WEB0022/CTR-REG/0008)		
**Sponsor Contact**	Swiss Tropical and Public Health Institute<br>Kreuzstrasse 2, 4123 Allschwil, Switzerland<br>Tel: +41 61 284 81 11		
**Principal Investigator**	Dr. Ally Olotu<br>Ifakara Health Institute (IHI)<br>P.O. Box 74, Bagamoyo, Tanzania<br>Email: aolotu@ihi.or.tz<br>Tel: +255 718 927 104		
**Funding Agency**	Sumaya Biotech GmbH & Co. KG		
**Investigational medicinal product**	SumayaVac-1 (SUM-101)*		
**Short Study Title**	Assessment of the malaria vaccine candidate SumayaVac-1 in healthy adults aged 18-45 years living in a malaria endemic country		

* The study is colloquially referred to as the ‘MSP1 study’.

The information contained in this document is confidential. It is intended solely for the Investigators, potential Investigators, consultants, or applicable Independent Ethics Committees and Regulatory Authorities. It is understood that this information will not be disclosed to others without prior written authorisation from the Sponsor, except where required by applicable local laws.

Based on Protocol Template, Swissethics-WHO for Clin Trials_v3.1_06.07.2020

1 General Information

I. List of Investigators and other persons involved

Names and titles of key persons in study

Names	Institution	Position	Function in study
Dr. Ally Olotu	Ifakara Health Institute	Senior Research Scientist, Program Leader	Principal Investigator
Dr. Aina-Ekisha Kahatano	Ifakara Health Institute	Research Officer	Clinician
Dr. Maxmillian Mpina	Ifakara Health Institute	Senior Research Scientist	Project Leader
Dr. Omary Hassan	Ifakara Health Institute	Research officer	Clinician
Sarah Mswata	Ifakara Health Institute	Research Scientist	Lab Manager

II. Signatures

Sponsor Representative

Signature	DocuSigned by:<br>Signer Name: Claudia Daubenberger<br>Signing Reason: I approve this document<br>Signing Time: 05-Dec-2023 | 16:08:06 CET<br>570FB241D0EF4659B902859A8C59854D	Date of Signature<br>05-Dec-2023 | 16:08:10 CET
Name	Prof. Dr. Claudia Daubenberger	
Title	Scientific Lead	
Institution	Swiss Tropical and Public Health Institute	
Address	Kreuzstrasse 2<br>4123 Allschwil<br>Switzerland	
Phone	+41 61 284 82 17	

Medical Responsible

Signature	DocuSigned by:<br>Signer Name: Daniel Paris<br>Signing Reason: I approve this document<br>Signing Time: 05-Dez-2023 | 16:10:42 CET<br>608447B2DF754BD0B115E05CD1DA9875	Date of Signature<br>05-Dec-2023 | 16:10:53 CET
Name	Prof. Dr. Daniel Paris	
Title	Medical Director	
Institution	Swiss Tropical and Public Health Institute	
Address	Kreuzstrasse 2<br>4123 Allschwil<br>Switzerland	
Phone	+41 61 284 81 23	

Project Responsible

Signature	DocuSigned by:<br>Signer Name: Suzanne Gajewski<br>Signing Reason: I have reviewed this document<br>Signing Time: 05-Dec-2023 | 15:22:37 CET<br>C8AE53226B55485F88AF2971BBD20CB5	Date of Signature<br>05-Dec-2023 | 15:22:41 CET
Name	Suzanne Gajewski	
Title	Clinical Research Manager	
Institution	Swiss Tropical and Public Health Institute	
Address	Kreuzstrasse 2<br>4123 Allschwil<br>Switzerland	
Phone	+41 61 284 89 62	

Statistician

Signature	DocuSigned by:<br>Signer Name: Fiona Vanobberghen<br>Signing Reason: I have reviewed this document<br>Signing Time: 06-Dec-2023 | 11:53:21 CET<br>99EE806461C740FCBC3B84712F5365C8	Date of Signature<br>06-Dec-2023 | 11:53:24 CET
Name	Fiona Vanobberghen	
Title	Statistician	
Institution	Swiss Tropical and Public Health Institute	
Address	Kreuzstrasse 2<br>4123 Allschwil<br>Switzerland	
Phone	+41 61 284 87 16	

Industrial Sponsor Representative

Signature	DocuSigned by:<br>Name des Unterzeichners: Ernst Böhnlein<br>Signiergrund: Ich genehmige dieses Dokument<br>Signierzeit: 05-Dez-2023 | 16:04:59 MEZ<br>4829AB79E23C41EE946789C0866152F0	Date of Signature<br>05-Dez-2023 | 16:05:06 MEZ
Name	Dr. Ernst Böhnlein	
Title	Managing Director	
Institution	Sumaya Biotech GmbH & Co. KG	
Address	Vangerowstrasse 20<br>69115 Heidelberg<br>Germany	
Phone	+49 6221 5 88 04 20	

Principal Investigator(s)

- I have read this protocol and agree that it contains all necessary details for carrying out this study. I will conduct the study as outlined herein and will complete the study within the time designated.
- I will ensure that all individuals and parties contributing to this study are qualified and I will implement procedures to ensure integrity of study tasks and data.
- I will provide copies of the protocol and all pertinent information to all individuals responsible to me who assist in the conduct of this study. I will discuss this material with them to ensure they are fully informed regarding the drug and the conduct of the study.
- I will use only the informed consent forms approved by the Sponsor or its representative and will fulfil all responsibilities for submitting pertinent information to the Independent Ethics Committees responsible for this study.
- I agree that the Sponsor or its representatives shall have access to any source documents from which Case Report Form information may have been generated.
- I agree to conduct the study in compliance with the current version of the Declaration of Helsinki, ICH-GCP E6(R2) as well as all the national legal and regulatory requirements.

Principal Investigator

Signature	DocuSigned by:<br>Signer Name: Ally Olotu<br>Signing Reason: I approve this document<br>Signing Time: 07-Dec-2023 | 15:34:19 CET<br>5BE062C2235F4CA5803E77C4D831A97E	Date of Signature<br>07-Dec-2023 | 15:34:25 CET
Name	Dr. Ally Olotu	
Title	Principal Investigator	
Institution	Ifakara Health Institute (IHI)	
Address	P.O. Box 74, Bagamoyo, Tanzania	
Phone	+255 718 927 104	

III. Protocol History

Version Number	Release Date	Comments
1.0	12 SEP 2022	Initial version
2.0	30 NOV 2023	- Removal of the controlled human malaria infection (CHMI) part of the study. Justification: The PfSPZ challenge component was put on hold by the FDA due to particulate matter identified in one vial of the PfSPZ inoculum produced by Sanaria for the SumayaVac-1 (SUM101) study. A new lot of the inoculum product could not be prepared and its purity verified in time for use during this study.<br>- Correction to the wording for the inclusion criteria on contraception requirements prior to vaccination.<br>- Administrative changes throughout the document.

IV. Summary of Changes From Previous Version

Section Number	Section Name	Summary of Changes
Not applicable (NA)	Title	Removed reference to CHMI
NA	Title page	Added registration numbers from clinicaltrials.gov and TMDA registry
NA	Title page	Added note to study being colloquially referred to as MSP1
1	General Information> I. List of Investigators and other persons involved	Updated names and positions of study team members as per active site delegation log
1	General Information> II. Signatures	Updated title of Medical Responsible
1	General Information> II. Signatures	Updated name, title and phone number of Project Responsible
1	General Information> III. Protocol History	Section added to capture the history of changes
1	General Information> IV. Summary of Changes From Previous Version	Section added to capture detailed changes from the previous version
1	General Information> V. Table of Contents	Updated numbering and sections based on changes in the entire document
1	General Information> VI. Abbreviations	- Updated table of abbreviations based on changes made in protocol<br>- Updated the name and abbreviation of the Bagamoyo clinical trial facility (BCTF) from Bagamoyo Research Trial Center (BRTC) due to the name change throughout the document
1	General Information> VII. Synopsis	Updated:<br>- Project lead name<br>- Study title<br>- Protocol version and date

Section Number	Section Name	Summary of Changes
		- Trial registration reference numbers<br>- Secondary and exploratory objectives to remove CHMI-related aspects and isotype distribution from secondary objective because it is part of the exploratory objective 1<br>- The second, third, fourth, sixth, eight, ninth, tenth and eleventh exploratory objectives to correct wording<br>- Added missing exploratory objectives (number twelfth and thirteenth) for the endpoints investigating the presence of intestinal helminth infections<br>- Primary, secondary and exploratory endpoints to remove all CHMI-related assessments<br>- Moved the secondary endpoint on assessing cellular immune responses in all participants by ELISpot using PBMCs, flow cytometry based immunophenotyping and function gene expression analysis to exploratory endpoints<br>- Study design<br>- Inclusion and exclusion criteria to remove CHMI-related wording and to update wording of inclusion criteria 3 to clarify contraception needs by male and female participants<br>- Measurements and procedures<br>- Number of participants for second part of study removed<br>- Study product/ intervention<br>- Study duration<br>- Study centre name<br>- Statistical analysis
2.2	Rationale for MSP-1 vaccine	Added updated information about RTS,S and R21 malaria vaccines
2.3	General treatment / Administration	- Added reference to MSP1 antigen of SUM-101 vaccine as the ‘drug product’<br>- Added name of adjuvant, ‘GLA-SE’<br>- Removed text regarding Sporozoites for CHMI
2.6	Immunogenicity studies> ELISpot Investigations	Updated T-cell response testing
3.1.1	Study rationale	Removed text regarding CHMI
3.1.3	Secondary objective	Updated objective and removed reference to CHMI and isotype distribution which is part of the exploratory objective 1
3.1.4	Exploratory objectives	- Updated objectives to remove CHMI-related aspects<br>- Added objectives for investigation of intestinal helminth infections to match the endpoints in protocol version 1.1

Section Number	Section Name	Summary of Changes
		- Removed CHMI-related objectives
3.2.1	Scientific justification - study population	Replaced 'gender' by 'sexes'
Previously 3.2.3	Scientific justification- CHMI	- Section removed completely<br>- Removal of section led to re-numbering of sections
3.2.5	Scientific justification- retrospective helminth infection analysis	Removed reference to CHMI on D84
4	Study Design	- Updated the study details and removed all references to CHMI (Part 2) of the study<br>- Updated Figure 1- Study design and removed CHMI part of the study
4.1.1	Primary endpoints	- Added measurement of endpoint to assess humoral immunogenicity 'in sera' to harmonize with synopsis<br>- Updated the description for endpoint to evaluate humoral immunogenicity and removed reference to analysis on D168 and D252 CHMI long term follow up visits<br>- Updated the method of assessing fold change of antibody responses to align with synopsis
4.1.2	Secondary endpoints	- Updated endpoints to remove CHMI-related aspects and visits<br>- Removed CHMI-related endpoints (development of parasitemia by qPCR and TBS)<br>- Updated endpoint for cellular immune responses to be done on total PBMCs
4.1.3	Exploratory endpoints	Harmonized to synopsis
4.2.1	Randomization	-Removed section about random selection of 25 participants for CHMI<br>-Added clarification that prepared dose(s) are labelled with participant ID and/or randomization number
4.2.2	Double blinding of vaccination and unblinding	Added, offering of Verorab to participants from Verorab group who withdrew consent during the study or were early discontinued on study unblinding
4.2.3	Masking of syringe	Updated instruction for pharmacist to handover re-constituted vaccines
4.3	Study duration and duration of participant's participation	Update study duration with deletion of CHMI part of the study
4.3.1	Schedule of events	- Removed CHMI part of the study, CHMI-related long term visits, and updated total blood volume withdrawn<br>- Harmonized instruction (b) with the details of measurements under the vital signs investigations in the text body<br>- Added dot for ECG as needed under Unscheduled Visit
4.4	Early termination of study	- Removed instance of CHMI

Section Number	Section Name	Summary of Changes
		- Harmonized throughout text for MSP1 antigen to be referred to as IMP
4.5.1	Study pause at any time point	- Remove instance of CHMI
4.5.2	Study pause at defined time-points	- Changed reference from three No-Go criteria to two No-Go criteria<br>- Removed 'No-Go criteria after all vaccinations and CHMI of group 1 and before CHMI of group 2'
Previously 4.5.3	Study pause for individual participants before CHMI	Removed whole section
4.6	Temporary contraindications for continuing vaccination	- Removed reference to CHMI<br>- Included guidance on temporary pause to be assessed on a case by case basis in consultation with Sponsor
5.2	Recruitment	Removed references to CHMI
5.3	Inclusion criteria	- Removed reference to CHMI from criteria 3<br>- Updated wording of criteria 3 to clarify contraception needs by male and female participants
5.4	Exclusion criteria	- Removed reference to CHMI from criteria 9
5.5.1	Withdrawal and discontinuation of individual participants	Updated wording on scheduling follow up visits for discontinued participants
6.1	Identity of investigational products	Removed CHMI part
Previously 6.1.2	Experimental intervention CHMI part	Removed section
6.3	Packaging, labelling and supply	- Updated name of company labeling, packaging and releasing MSP1 drug product<br>- Removed reference to CHMI
6.4	Storage conditions	- Removed CHMI-related storage conditions<br>- Added 'control vaccine' not to be used beyond expiry date
6.5.1	Experimental intervention - SumayaVac-1 (SUM-101) vaccine	Updated title to include vaccine name
Previously 6.5.2	Experimental intervention- CHMI part	Removed section
6.6	Dose modifications	Removed reference to CHMI
6.7	Compliance with study interventions	Removed reference to CHMI
6.8	Concomitant interventions (treatments)	- Re-worded details of capturing route of administration of prior and concomitant medication in source and eCRF<br>- Removed start time of concomitant medication as it was intended to pertain primarily to start of ALU after CHMI
6.9	Experimental Intervention accountability	- Removed reference to CHMI<br>- Added the word adjuvant after GLA-SE for clarity
6.10	Return or destruction of experimental Intervention	- Removed reference to Sporozoites<br>- Updated decision to destroy unused IMP

Section Number	Section Name	Summary of Changes
7.1.2	Screening visit 1 (D-35 to D-1)	- Replaced term 'screening number' with 'participant ID'<br>- Added haematocrit to parameters measured in haematology
7.1.3	Screening visit 2 (D-35 to D-1)	Updated wording for distribution of insecticide treated bed nets after meeting eligibility criteria
7.1.5	1st, 2nd and 3rd Vaccination visit (D0, D28 and D56)	Under the section 'After 2 hours', removed the words 'such as', since the vital signs parameters are clearly defined
7.1.7	7 day site visit post 1st, 2nd and 3rd vaccination (D7, D35 and D63)	- Added '(only on D63)' after 'immunological analyses' for clarity<br>- Added ALT, total bilirubin and creatinine for clarity for biochemistry analysis on 7 day follow up site visits<br>- Removed blood sampling for Malaria testing by qPCR and TBS (only on D63), as this was to be done for the purpose of confirming malaria negativity before CHMI
7.1.8	14 day site visit post 1st, 2nd and 3rd vaccination (D14, D42 and D70)	Added ALT, total bilirubin and creatinine for clarity for biochemistry analysis on 14 day follow up site visits
7.1.9	28 day site visit post 1st, 2nd and 3rd vaccination (D28, D56 and D84)	- Reworded the time-points of assessments for clarity<br>- Added ALT, total bilirubin and creatinine for clarity for biochemistry analysis on 28 day follow up site visits<br>- Removed reference to CHMI and CHMI-related assessments
Previously 7.1.10	CHMI (D84)	Removed section
Previously 7.1.11	1-4 days post CHMI (D85-88)	Removed section
Previously 7.1.12	Site stay post CHMI (D89-maximun D115)	Removed section
Previously 7.1.13	Malaria treatment post-CHMI (between D89-112)	Removed section
Previously 7.1.14	Site discharge post CHMI confinement (between D89-115)	Removed section
7.1.10	Vaccination site follow-up (D112, D140) visits	- Updated section to remove reference to CHMI and CHMI-related visits<br>- Removed D168 and D252 follow up visits<br>- Added ALT, total bilirubin and creatinine for clarity for biochemistry analysis on D112 and D140 follow up site visits<br>- Removed sentence regarding combining visits if participant 'discharge' (from clinic stay) is after D105, since this is no longer relevant
7.1.11	Unscheduled visit	- Added use of any other procedure deemed necessary by clinician<br>- Re-worded documentation of unscheduled visits

Section Number	Section Name	Summary of Changes
8.1	Safety assessments	Removed reference to CHMI and CHMI-related assessments
8.1.2	Vital signs	Removed reference to CHMI and CHMI-related assessments
8.1.3	12-lead ECG safety recording	Added use of ECG 'at any time' in case of cardiac events for clarity
8.1.4	Laboratory assessments	- Re-worded clinically significant laboratory abnormalities for clarity<br>- Biochemistry- added references to visits to clarify which assessments are done at which visit
8.2.2	Types and recording of AEs	Removed reference to CHMI
8.2.3	Grading of adverse events severity	- Added reference to CTCAE for grading AEs<br>- Added reference to site's manual of reference intervals for grading laboratory abnormalities and vital signs
8.2.6	Expectedness of AEs	Removed malaria related symptoms in reference to CHMI
9.2	Determination of sample size	Removed CHMI-related sample size
9.3	Description of statistical methods	Updated wording on trial statistician performing analyses that will be reported in SMC meetings and clinical study report
9.3.1	Datasets to be analysed, analysis populations	- Removed reference to CHMI<br>- Updated population on which immunogenicity assessments will be performed
9.3.1.1	Primary analysis	Replaced gender with sex
9.3.1.2	Secondary analyses	Removed reference to CHMI
9.3.1.3	Interim analyses	Added information on what analyses will be done and by whom
10.3	Evaluation of the risk-benefit ratio	Removed reference to CHMI
10.5	Registration of clinical trial	Updated the trial 'was' registered
10.9	Participant reimbursement	- Updated compensation details<br>- Removed reference to CHMI
11.1.1	Case Report Forms	- Removed vital signs as all vital signs captured in eCRF not only out of range values<br>- Re-worded the meaning of baseline assessment to include D0 pre-vac visit<br>- Changed terms 'out of range' to 'abnormal' in reference to physical examination and 'values' to 'documentation'
11.1.3	Record keeping/ archiving	Updated number of years for archiving the code list and medical data to 25 years
11.3.2	Study-specific preventive measures	- Removed reference to CHMI<br>- Removed section in reference to preventive measures during confinement after CHMI<br>- Under the section birth control and pregnancy testing- removed reference to CHMI and CHMI-related follow-up visits

Section Number	Section Name	Summary of Changes
		- Under the section for risks associated with epidemics- removed section in reference to confinement post CHMI
11.4	Safety monitoring committee (SMC)	- Added requirement of 'at least' 1 Tanzanian member
11.6	Storage of biological material and related health data	- Removed transcriptomic analyses of parasites
12	Funding	- Administrative corrections to funder name
13	Dissemination of results and publication policy	For clarity added the clinical trial agreement is with the site
14	References	Updated references

V. Table of contents

1 General Information ... 2
I. List of Investigators and other persons involved ... 2
II. Signatures ... 3
III. Protocol History ... 6
IV. Summary of Changes From Previous Version ... 6
V. Table of contents ... 13
VI. Abbreviations ... 16
VII. Synopsis ... 19
2 Background information ... 26
2.1 Malaria disease ... 26
2.2 Rationale for MSP-1 vaccine ... 26
2.3 General treatment/ Administration ... 27
2.4 Efficacy ... 27
2.5 Scientific justification - Pharmacodynamics & Pharmacokinetics ... 27
2.6 Immunogenicity studies ... 28
2.7 Safety and tolerability ... 28
2.8 Toxicity ... 29
2.9 Justification for the dosage and dosage plan, and the length of treatment ... 29
3 Objectives and purpose ... 29
3.1 Study rationale and objectives ... 29
3.1.1 Study rationale ... 29
3.1.2 Primary objectives ... 29
3.1.3 Secondary objective ... 29
3.1.4 Exploratory objectives ... 30
3.2 Scientific justification and rationale ... 30
3.2.1 Scientific justification - study population ... 30
3.2.2 Scientific justification - vaccinations ... 30
3.2.3 Scientific justification - Dose ... 31
3.2.4 Scientific justification for deviation from Verorab® dosing schema ... 31
3.2.5 Scientific justification- retrospective helminth infection analysis ... 31
4 Study design ... 31
4.1 Primary and secondary endpoints ... 32
4.1.1 Primary endpoints ... 32
4.1.2 Secondary endpoints ... 32
4.1.3 Exploratory endpoints ... 33
4.2 Measures to minimize bias ... 34
4.2.1 Randomization ... 34
4.2.2 Double blinding of vaccination and unblinding ... 34
4.2.3 Masking of syringe ... 35
4.3 Study duration and duration of participant's participation ... 35
4.3.1 Schedule of events ... 36
4.4 Early termination of the study ... 37
4.5 Stopping rules ... 37
4.5.1 Study pause at any time point ... 37
4.5.2 Study pause at defined time-points ... 38
4.6 Temporary contraindications for continuing vaccination ... 38
5 Selection of the study participants ... 38
5.1 Study setting ... 38
5.2 Recruitment ... 39
5.3 Inclusion criteria ... 40
5.4 Exclusion criteria ... 40
5.5 Criteria for discontinuation of study ... 41
5.5.1 Withdrawal and discontinuation of individual participants ... 41
5.5.2 Discontinuation because stopping rule is met ... 41
6 Treatment of participants ... 42
6.1 Identity of investigational products ... 42
6.1.1 Experimental intervention vaccination ... 42
6.2 Comparator ... 42
6.3 Packaging, labelling and supply ... 42
6.4 Storage conditions ... 43
6.5 Administration of experimental and comparator interventions ... 43
6.5.1 Experimental intervention - SumayaVac-1 (SUM-101) vaccine ... 43
6.6 Dose modifications ... 43
6.7 Compliance with study interventions ... 43

6.8 Concomitant interventions (treatments) ... 43
6.9 Experimental Intervention accountability ... 44
6.10 Return or destruction of experimental Intervention ... 44
7 Study assessments ... 44
7.1 Procedures per visit ... 44
7.1.1 General order of assessments ... 44
7.1.2 Screening visit 1 (D-35 to D-1) ... 45
7.1.3 Screening visit 2 (D-35 to D-1) ... 45
7.1.4 Pre 1st Vaccination (D-1 to D0) ... 46
7.1.5 1st, 2nd and 3rd Vaccination visit (D0, D28 and D56) ... 46
7.1.6 1-6 days post 1st, 2nd and 3rd vaccination follow-up (D1-6, D29-34 and D57-62) ... 47
7.1.7 7 day site visit post 1st, 2nd and 3rd vaccination (D7, D35 and D63) ... 47
7.1.8 14 day site visit post 1st, 2nd and 3rd vaccination (D14, D42 and D70) ... 47
7.1.9 28 day site visit post 1st, 2nd and 3rd vaccination (D28, D56 and D84) ... 47
7.1.10 Vaccination site follow-up (D112, D140) visits ... 48
7.1.11 Unscheduled visit ... 48
7.2 Total blood volume ... 49
8 Assessment of safety ... 49
8.1 Safety assessments ... 49
8.1.1 Physical Examination ... 49
8.1.2 Vital Signs ... 49
8.1.3 12-lead ECG safety recording ... 49
8.1.4 Laboratory assessments ... 50
8.2 Collection and reporting of adverse events ... 50
8.2.1 Definition of adverse events (AEs) ... 50
8.2.2 Types and recording of AEs ... 50
8.2.3 Grading of adverse events severity ... 51
8.2.4 AE causality assessment ... 51
8.2.5 Serious adverse events (SAEs) ... 51
8.2.6 Expectedness of AEs ... 52
8.2.7 Reporting Obligations ... 52
8.3 Follow-up of adverse events ... 53
8.4 Data collection and follow-up of prematurely terminated participants ... 53
9 Statistics ... 53
9.1 Hypothesis ... 53
9.2 Determination of sample size ... 53
9.3 Description of statistical methods ... 54
9.3.1 Datasets to be analysed, analysis populations ... 54
9.4 Handling of data ... 55
10 Ethical considerations ... 55
10.1 Independent Ethics Committee (IEC) ... 56
10.2 Regulatory Authority (RA) ... 56
10.3 Evaluation of the risk-benefit ratio ... 56
10.4 Participant information and consent ... 56
10.5 Registration of clinical trial ... 57
10.6 Participant confidentiality ... 57
10.7 Participants requiring particular protection ... 57
10.8 Insurance ... 57
10.9 Participant reimbursement ... 58
10.10 Protocol amendments ... 58
11 Quality control and quality assurance: description of measures ... 58
11.1 Data handling and record keeping / archiving ... 58
11.1.1 Case Report Forms ... 58
11.1.2 Specification of source documents ... 59
11.1.3 Record keeping/ archiving ... 59
11.2 Data management ... 59
11.2.1 Data management system ... 59
11.2.2 Data security, access and back-up ... 59
11.2.3 Analysis and archiving ... 60
11.2.4 Electronic and central data validation ... 60
11.3 Risk management ... 60
11.3.1 Risk identification, assessment and mitigation ... 60
11.3.2 Study-specific preventive measures ... 60
11.4 Safety monitoring committee (SMC) ... 61
11.5 Translations - Reference language ... 61
11.6 Storage of biological material and related health data ... 62
12 Funding ... 62

13 Dissemination of results and publication policy........62
14 References........63
15 Appendices........65
15.1 Laboratories analysing biological materials........65

VI. Abbreviations

AAHI	Access to Advanced Health Institute
ADCC-NK cells	Antibody-Dependent Cell-Mediated Cytotoxicity Natural Killer Cells
ADRB	Antibody-Dependent Respiratory Burst
AE	Adverse Event
ALT	Alanine Aminotransferase
ALU	Artemether/ Lumefantrine
AST	Aspartate Aminotransferase
A-V heart block	Atrioventricular heart block
BCTF	Bagamoyo Clinical Trial Facility
BMI	Body Mass Index
BP	Blood Pressure
CD4	Cluster of Differentiation 4
CD8	Cluster of Differentiation 8
CRF	Case Report Form
CRP	C-Reactive Protein
CTCAE	Common Terminology Criteria for Adverse Events
E. coli	Escherichia coli
ECG	Electrocardiogram
eCRF	Electronic Case Report Form
EDC	Electronic Data Capture
EKNZ	Ethikkommission Nordwest- und Zentralschweiz
ELISA	Enzyme-Linked Immunosorbent Assay
ELISpot	Enzyme-Linked Immune Absorbent Spot
EMF EU	End Malaria Fund European Union
FDA	United States Food and Drug Administration
GCP	Good Clinical Practice
GIA	Growth Inhibition Assay
GLA-SE	Glucopyranosyl Lipid Adjuvant-Stable Emulsion
GMP	Good Manufacturing Practice
GPA	Glycophorin A
GPI	Glycosylphosphatidylinositol
ɣδ T-cell	Gamma Delta T-cells
HBV	Hepatitis B Virus
HCV	Hepatitis C Virus
HIV	Human Immunodeficiency Virus

HR	Heart Rate
i.m.	Intramuscular
ICF	Informed Consent Form
ICH	International Council on Harmonisation of Technical Requirements for Registration of Pharmaceuticals for Human Use
ICS	Intracellular Cytokine Staining
ID	Identification
IEC	Independent Ethics Committee
IFN-γ	Interferon Gamma
IgG	Immunoglobulin G
IgM	Immunoglobulin M
IHI	Ifakara Health Institute
IL-10	Interleukin-10
IMP	Investigational Medicinal Product
INN	International Nonproprietary Name
IRB	Institutional Review Board
ISF	Investigator Site File
ITT	Intent to Treat
IU	International Unit
IUD	Intrauterine Device
IUS	Intrauterine Hormonal Releasing System
Kg	Kilogram
m^2	meter square
MAC	Membrane Attack Complex
MD	Doctor of Medicine
MHC	Major Histocompatibility Complex
ml	Millilitre
MSP-1	Merozoite Surface Protein-1
MSPDBL	MSP Duffy Binding-Like
NaCl	Sodium Chloride
NatHREC	National Health Research Ethics Review Committee
NOAEL	No Observed Adverse Effect Level
PBMC	Peripheral Blood Mononuclear Cells
PfSPZ	Plasmodium falciparum Sporozoites
PhD	Doctor of Philosophy
PI	Principal Investigator
PV	Parasitophorous Vacuoles

qPCR	Quantitative Polymerase Chain Reaction
RA	Regulatory Authority
RBC	Reb Blood Cell
RDT	Rapid Diagnostic Test
ROS	Reactive Oxygen Species
RR	Respiratory Rate
SAE	Serious Adverse Event
SMC	Safety Monitoring Committee
SOP	Standard Operating Procedure
SUSAR	Suspected Unexpected Serious Adverse Reaction
Swiss TPH	Swiss Tropical and Public Health Institute
TBS	Thick Blood Smear
TH1	Type 1 T Helper
TMDA	Tanzania Medicines and Medical Devices Authority
TMF	Trial Master File
TSH	Tanzanian Shilling
USD	United States Dollar
WBC	White Blood Cell
WHO	World Health Organization
WOCBP	Women of Child Bearing Potential
µg	Microgram
µl	Microlitre

VII. Synopsis

**Sponsor**	Swiss Tropical and Public Health Institute
**Project Lead**	Claudia Daubenberger (Scientific) & Suzanne Gajewski (Operational)
**Study Title**	A randomised, controlled, double-blind, parallel group, single center Phase Ib trial to assess safety, reactogenicity and immunogenicity of a candidate dual-stage malaria vaccine, SumayaVac-1 (MSP-1 with GLA-SE as adjuvant) in healthy, malaria exposed adults of African origin aged 18-45 years
**Short Title**	Assessment of the malaria vaccine candidate SumayaVac-1 in healthy adults aged 18-45 years living in a malaria endemic country
**Protocol Version and Date**	V2.0, 30 November 2023
**Trial registration**	Clinicaltrials.gov (NCT05644067)<br>TMDA registry (TRC-WEB0022/CTR-REG/0008)
**Study Category with Rationale**	Risk category according to HRA (Clin O) - category C<br>Investigational Medicinal Product is not authorized for use
**Clinical Phase**	Phase Ib
**Background and Rationale**	Malaria remains a major infectious disease causing a heavy burden of mortality and morbidity in populations living in tropical and subtropical regions. However, despite important insights gained in different vaccine development approaches, there is currently no vaccine with sufficient and long-lasting protection against malaria. The Phase Ia results of the proposed vaccine candidate showed promising results in Caucasian participants, however, results in the population living in the malaria affected regions are needed.

**Objective(s)**	**To evaluate in healthy adults of African origin previously exposed to the malaria parasite receiving SumayaVac-1 (SUM-101) versus rabies control (Verorab®) vaccine:**<br>**Primary Objectives**<br>• Safety and reactogenicity of SumayaVac-1 (SUM-101).<br>• Immunogenicity of SumayaVac-1 (SUM-101).<br>**Secondary Objectives**<br>• SumayaVac-1 (SUM-101) vaccine-induced antibody levels, *in vitro* effector functions.<br>**Exploratory Objectives**<br>• Comparison of SumayaVac-1 (SUM-101) induced immunoglobulin isotype distribution and duration between malaria pre-exposed and malaria naïve participants from the previous Phase Ia study in Heidelberg.<br>• Fine scale epitope mapping of SumayaVac-1 (SUM-101) specific antibodies using peptide arrays, to investigate humoral immune response at baseline with vaccine induced responses.<br>• Comparison of SumayaVac-1 (SUM-101) induced cellular immunity between malaria pre-exposed and malaria naïve participants from the previous Phase Ia study in Heidelberg including fine scale epitope mapping of MSP-1 specific CD4+ and CD8+ T cells.<br>• Investigation of the B- and T-Cell repertoire before and after SumayaVac-1 (SUM-101) vaccination.<br>• Integrated transcriptome and immunoglobulin gene repertoire analyses of MSP-1 specific B-cells using single-cell technologies.<br>• Glycosylation patterns of MSP-1 specific functional antibodies.<br>• Investigate off-target IgG and IgM repertoire after SumayaVac-1 (SUM-101) vaccination using immunoproteomics.<br>• Investigate the structure of MSP-1 protein bound to functional antibodies by cryo-electron tomography to map conformational epitopes.<br>• To evaluate mechanisms of malaria specific antibody diversity generation, post-translational modification of antibodies, and B- and T-cell memory generation and maintenance.<br>• *Ex vivo* assessment of changes in human peripheral blood transcriptome in participants having received SumayaVac-1 (SUM-101) versus Verorab® rabies vaccine.<br>• Description of ɣδ T-cell receptor repertoire, transcriptome, functional activity and phenotypes in participants having received SumayaVac-1 (SUM-101) versus Verorab® rabies vaccine.<br>• Investigate impact of presence of intestinal helminth infections on SumayaVac-1(SUM-101) induced humoral immune response.<br>• Investigate impact of presence of intestinal helminth infections on SumayaVac-1(SUM-101) induced cellular immune response.
**Primary, Secondary and Exploratory Endpoints**	**Primary endpoints:**<br>The following endpoints will be measured in all participants to evaluate the safety and reactogenicity of SumayaVac-1 (SUM-101):<br>• Local and systemic solicited adverse events (AEs) at least possibly related to the investigational medicinal product (IMP) recorded after each vaccination (done on D0, D28 and D56) up to 7 days later.

	• Local and systemic unsolicited reactogenicity recorded after each vaccination (done on D0, D28 and D56) up to 28 days later.<br>• Any serious adverse events (SAE) occurring after the 1st vaccination until the participant's last visit.<br>• Changes in laboratory safety parameters between baseline (D0 before 1st vaccination) to 28 days after each of the vaccinations.<br>• Changes in laboratory safety parameters between values just prior to each vaccination (on D0, D28 and D56) and values 28 days after that vaccination.<br>The following endpoints will be measured in all participants in sera collected at D0 pre-vaccination, D28 (W4), D56 (W8), D84 (W12), D112 (W16) and D140 (W20), to evaluate the humoral immunogenicity:<br>• Longevity of antibody responses to SumayaVac-1 (SUM-101) by ELISA.<br>• Fold change of antibody responses to SumayaVac-1 (SUM-101) in comparison to baseline (D0 pre-vaccination) by ELISA.<br>**Secondary endpoints:**<br>The following endpoints will be measured in the sera or blood of all participants, at D0 pre-vaccination, D28 (W4), D56 (W8), D84 (W12), D112 (W16) and D140 (W20):<br>• Evaluation of the opsonic phagocytosis activity.<br>• Evaluation of complement fixation, activation and/or membrane attack complex (MAC) formation.<br>• Evaluation of antibody-dependent respiratory burst (ADRB) activity.<br>• Evaluation of antibody-dependent cellular cytotoxicity (ADCC-NK cells) activity.<br>• Evaluation of immune-mediated growth inhibition activity on a panel of *Plasmodium falciparum (P.falciparum)* lines.<br>The following endpoint will be assessed among SumayaVac-1 (SUM-101) participants only:<br>• Comparison of MSP-1 IgG antibody concentrations by ELISA between malaria pre-exposed participants in the current study and malaria naïve participants from the previous Phase Ia study in Heidelberg.<br>**Exploratory endpoints:**<br>• Comparison of SumayaVac-1 (SUM-101) induced immunoglobulin isotype distribution and duration between malaria pre-exposed and malaria naïve participants from the previous Phase Ia study in Heidelberg.<br>• Fine scale epitope mapping of SumayaVac-1 (SUM-101) specific antibodies, using peptide arrays, to investigate baseline humoral responses and vaccine induced humoral responses<br>• Comparison of SumayaVac-1 (SUM-101) induced cellular immunity between malaria pre-exposed and malaria naïve participants from the previous Phase Ia study in Heidelberg including fine scale epitope mapping of MSP-1 specific CD4+ and CD8+ T cells.<br>• Investigation of the B- and T-Cell receptor repertoire before and after SumayaVac-1 (SUM-101) vaccination.<br>• Cellular immune responses to SumayaVac-1 (SUM-101) in all participants by (i) ELISpot assays using total PBMCs, (ii) SumayaVac-1 (SUM-101) specific cells characterised by flow cytometry-based immunophenotyping using intracellular cytokine

	staining (ICS), (iii) functional gene expression analysis, and/or other assays to be defined; at D0 pre-vaccination and D56 (W8), D84 (W12), D112 (W16) and D140 (W20).<br>• Integrated transcriptome and immunoglobulin gene repertoire analyses of MSP-1 specific B-cells using single-cell technologies.<br>• Glycosylation patterns of MSP-1 specific functional antibodies.<br>• Investigate off-target IgG and IgM repertoire after SumayaVac-1 (SUM-101) vaccination using immunoproteomics.<br>• Investigate the structure of MSP-1 protein bound to functional antibodies by cryo-electron tomography to map conformational epitopes.<br>• *Ex vivo* assessment of changes in human peripheral blood transcriptome in participants having received SumayaVac-1 (SUM-101) versus Verorab® rabies vaccine.<br>• Description of ɣδ T-cell receptor repertoire, transcriptome, functional activity and phenotypes in participants having received SumayaVac-1 (SUM-101) versus Verorab® rabies vaccine.<br>• Investigate the impact of the presence of intestinal helminth infections on vaccine-induced humoral immune response by comparing the quality and quantity of SumayaVac-1 (SUM-101) specific antibody isotypes between helminth infected and non-infected participants at baseline.<br>• Investigate the impact of the presence of intestinal helminth infections on SumayaVac-1 (SUM-101) vaccine-induced cellular immune responses by comparing the quality and quantity of SumayaVac-1 (SUM-101) specific cytokine production and ICS results between helminth infected and non-infected participants at baseline.
**Study Design**	This is a randomised, controlled, double-blind, parallel group, single center Phase Ib trial to assess the safety, reactogenicity, and immunogenicity of SumayaVac-1 (SUM-101) in healthy, malaria-exposed adults of African origin aged 18-45 years.<br>In total, 40 participants will be enrolled (male and female). 20 participants will be randomised to receive three monthly inoculations (on D0, D28 and D56) with the investigational product, SumayaVac-1 (SUM-101), and 20 participants will be randomised to receive the registered rabies vaccine, Verorab®, as controls.<br>For operational reasons the participants will be split in two groups of 20 participants.<br>• **Group 1** will have a sentinel subgroup (2 SumayaVac-1 (SUM-101) & 1 Verorab® rabies vaccine) with a 48 hours safety surveillance period before the remaining 17 participants (8 SumayaVac-1 (SUM-101) & 9 Verorab® rabies vaccine) of the group 1 receive their 1st vaccination.<br>• **Group 2** will be composed of 20 participants (10 SumayaVac-1 (SUM-101) & 10 Verorab® rabies vaccine). All visits in group 2 will be shifted by at least 3 weeks compared to group 1 to create minimal overlap of study-related activities.<br>After each vaccination (done on D0, D28 and D56), the participants will remain at the facility for 2 hours prior to their discharge for home. The participant will be called daily by phone (or home visits if required) until 6 days post vaccination for follow-up.<br>For all participants, vaccination follow-up visits at the site will occur at 7, 14 and 28 days after each vaccination.

**Inclusion/Exclusion Criteria**	**Inclusion criteria**<br>1. Written informed consent obtained before any study procedure.<br>2. Literate participants aged 18-45 years of African origin.<br>3. Female participants practicing contraception from 4 weeks before 1st immunization and both female and male participants willing to practice contraception up to 12 weeks after the last immunization.<br>4. Available to participate in follow-up for the duration of the study.<br>5. Contactable by phone during the whole study period.<br>6. At least two years residence in the Bagamoyo district or nearby districts in Coastal and Dar-es-Salaam regions and planning to reside there for at least 9 more months.<br>7. Agreement to provide personal contact information and contact information of another household member or close friend.<br>8. Female participants must be willing to avoid pregnancy if selected for participation in the trial and to undergo multiple serum pregnancy testing.<br>9. Confirmation of understanding of design, procedures, risk and benefits of the study in a test with maximum of two attempts.<br>10. General good health based on assessment of medical history and clinical examination.<br>**Exclusion Criteria**<br>1. Previous participation in any malaria vaccine trial in the last 3 years.<br>2. Participation in any other clinical trial involving investigational medicinal products within 30 days prior to the screening assessment.<br>3. Previous history of drug or alcohol abuse interfering with normal social function within one year prior to enrolment.<br>4. Previous vaccination with a rabies vaccine.<br>5. Intake of chronic medication, especially immunosuppressive agents (steroids, immunomodulating drugs) during the 13 weeks preceding the screening visit or during the study period.<br>6. Known hypersensitivity to any of the vaccine components (adjuvant or protein) or anti-malarial treatments.<br>7. Body mass index (BMI) of <18 or >30 Kg/m^2.<br>8. Participants unable to be closely followed for social, geographic or psychological reasons.<br>9. Any vaccination from 4 weeks prior to the 1st vaccination and (none planned) up to 6 weeks after the 3rd vaccination.<br>10. Symptoms, physical signs or laboratory values suggestive of systemic disorders, including renal, hepatic, cardiovascular, pulmonary, skin, immunodeficiency, psychiatric and other conditions, which could interfere with the interpretation of the trial results or compromise the health of the participants.<br>11. Abnormal electrocardiogram on screening: pathologic Q wave and significant ST-T wave changes, left ventricular hypertrophy, clinically significant arrhythmias, left bundle branch block, secondary or tertiary A-V (atrio-ventricular) heart block.<br>12. Any clinically significant laboratory values at screening outside of normal ranges for study participants.<br>13. Malaria positivity at screening (microscopy or qPCR positive).<br>14. Positive HIV, HBV or HCV tests.<br>15. For females: Positive pregnancy test or actively breast feeding.

**Measurements and Procedures**	Written informed consent will be taken prior to any study procedure. Healthy participants will be screened and randomised before administration of the study IMP.<br>On the day of the 1st vaccination, the health status of the participant is re-checked and eligibility confirmed. Samples for safety, humoral, cellular and exploratory measurements are taken before 1st vaccination (baseline). Vaccination is administered and the participant remains at site for 2 hours before being discharged. Telephone follow-ups (or home visits if required) will be performed daily until 6 days after each vaccination. On 7, 14 and 28 days after each vaccination (done on D0, D28 and D56), the participant will be invited to come back to the facility and health status is checked.<br>Further participant follow-up visits will take place at D112 (W16) and D140 (W20) post 1st vaccination. Sampling for humoral and cellular responses will be performed at D112 (W16) and D140 (W20). Additionally, sampling for exploratory measurements will be performed at D112 (W16) (Vac1 + 4 month follow-up site visit).
**Number of Participants with Rationale (if no Power Analysis conducted)**	**<u>Vaccination: 40 adult participants</u>**<br>Overall: 20 SumayaVac-1 (SUM-101) and 20 Verorab® control<br>**Group 1**<br>Sentinel sub-group<br>- 2 SumayaVac-1 (SUM-101)<br>- 1 Verorab® control<br>Follower Group<br>- 8 SumayaVac-1 (SUM-101)<br>- 9 Verorab® control<br>**Group 2**<br>- 10 SumayaVac-1 (SUM-101)<br>- 10 Verorab® control<br>As this is a Phase I trial, no formal sample size calculation has been done. The sample size is considered sufficient to examine the safety and reactogenicity of SumayaVac-1 (SUM-101), and humoral and cellular immunogenicity.
**Study Product / Intervention**	**<u>Vaccination:</u>**<br>Intra-muscular injection of SumayaVac-1 (SUM-101) composed of 150 µg MSP-1 drug product + 5 µg GLA-SE adjuvant
**Control Intervention (if applicable)**	**<u>Vaccination control:</u>**<br>Intra-muscular injection of rabies vaccine (Verorab®)
**Study Duration**	The total study duration including the screening period (~4 weeks) combining Group 1 and 2 will be approximately 35 weeks.<br>The study duration for each participant will be ~24 weeks (4 weeks of screening period plus 20 weeks of vaccinations and long-term follow-up visits).
**Study Schedule**	First participant first visit: 30 Aug 2023<br>Last participant last visit: Estimated April, 2024
**Investigator(s)**	Ally Olotu
**Study Centre**	The Ifakara Health Institute Bagamoyo Clinical Trial Facility

**Statistical Analysis incl. Power Analysis**	See above for sample size justification.<br>To evaluate the safety and reactogenicity primary endpoints, AEs and SAEs will be presented according to the endpoint definitions above. AE reporting will include verbatim term, preferred term (PT), system organ class (SOC), treatment, severity, relationship to the interventional products, and seriousness, reporting numbers of participants experiencing each event and total numbers of each event. Laboratory safety parameters will be summarized as absolute values and changes at 28 days after each vaccination compared to baseline (before 1st vaccination) and compared to values just prior to each vaccination.<br>To evaluate the immunogenicity primary endpoints as defined above, SumayaVac-1 (SUM-101) vaccine induced humoral immunogenicity will be summarized as antibody responses to SumayaVac-1 (SUM-101) by ELISA over time, and fold changes of antibody responses relative to baseline.<br>For all analyses, data will be listed for each participant. Descriptive analyses will be performed (number of observations, arithmetic or geometric mean, standard deviation, minimum, maximum, median, interquartile range, as appropriate, for continuous data, and counts and percentages for categorical data). Results will be presented by vaccination received.<br>Further details will be elaborated in a Statistical Analysis Plan (SAP), including for the secondary and exploratory objectives.
**Ethical consideration**	This study will be conducted in compliance with the protocol, the current version of the Declaration of Helsinki, the ICH-GCP as well as all national legal and regulatory requirements. This protocol will be reviewed by the Ethikkommission Nordwest- und Zentralschweiz (EKNZ, Ethics Committee of Northern and Central Switzerland) and also be reviewed and approved by the Ifakara Health Institute Review Board (IHI-IRB) and the National Health Research Ethics Sub-Committee (NatHREC) in Tanzania before starting the study.

2 BACKGROUND INFORMATION

2.1 Malaria disease

Malaria is a life threatening disease and considered to be one of the biggest global health burdens caused by an infectious disease, particularly in Sub-Saharan Africa [1]. The 2021 World Health Organization (WHO) malaria report estimated that there were 241 million malaria cases in 85 malaria endemic countries in 2020, an additional 14 million malaria cases compared to 2019. In total, an estimated 627,000 malaria-related deaths were reported in 2020, with the WHO African Region home to 95% of all reported malaria cases and deaths [2]. The malaria disease is caused by parasites belonging to *Plasmodium* species with *P. falciparum* accounting for 99.7% of estimated malaria cases in the WHO African Region in 2020 [2]. Malaria infections mainly affect vulnerable populations, namely children aged under 5 years, accounting for 77% of all malaria deaths, and primigravid pregnant women [2].

The parasite life cycle of malaria involves two hosts, the human host, where asexual reproduction takes place, and the mosquito vector, where sexual reproduction occurs [3]. Malaria transmission to humans is initiated through the bites of infected female Anopheles mosquitoes. During a blood meal, sporozoites of malaria parasites are inoculated into the human skin and some of these sporozoites are transported through the bloodstream to the liver [3]. In the liver, sporozoites infect hepatocytes and develop into liver stage merozoites 6-7 days after sporozoite inoculation [3]. Thousands of merozoites can then be released by each infected hepatocyte into the blood stream, where they initiate asexual reproduction by invading the red blood cells (RBCs) [3]. The invasion of RBCs by merozoites follows a sequence of complex receptor-ligand interactions and the formation of parasitophorous vacuoles (PV) after invagination of the RBC membrane [4]. This allows the parasites to enter a nutrient rich environment, highly protected by the immune system since RBCs lack the expression of Major Histocompatibility Complex (MHC) class I and MHC class II molecules. After erythrocyte invasion, merozoites develop into ring, trophozoite and schizont stages within the PV in about 48 hours in the case of *P. falciparum*, until newly developed merozoites egress by sequential rupture of the parasitophorous vacuole membrane and host cell membrane [5, 6]. Free merozoites invade new RBCs within minutes and during this short time window, the merozoite surface is exposed to factors of the immune system [4].

A number of membrane integral and peripheral proteins form a thick fibrillar coat on the merozoite surface. The Merozoite Surface Protein (MSP)-1 is one of the most abundant proteins on the merozoite surface which has been studied in detail with respect to its biology and structure [7, 8]. MSP-1 consists of four subunits, namely p83, p30, p38 and p42, which are held together non-covalently. A glycosylphosphatidylinositol (GPI) anchor links the complex to the merozoite membrane via the p42 subunit [9]. The MSP-1 complex is known to interact with a range of other MSPs, including MSP-3, MSP-6, MSP-7 and MSP-9, MSP Duffy binding-like (MSPDBL)-1 and MSPDBL-2, forming various different complexes on the parasite surface [10]. MSP-1 is essential for *P. falciparum* development given that MSP-1 knockdown mutants could not be generated *in vitro* [11]. MSP-1 might be involved in early RBC attachment and invasion of merozoites through binding to the RBC surface proteins glycophorin A (GPA) and band 3 [12, 13]. MSP-1 also interacts with the erythrocyte cytoskeleton protein spectrin and plays a role in egression from RBCs [14].

2.2 Rationale for MSP-1 vaccine

Large, international research efforts have been invested into the development of anti-malaria vaccination strategies. Currently, the World Health Organization (WHO) has approved RTS,S, and R21 malaria pre-erythrocytic vaccines for deployment to infants 5-17 months and <3 years of age respectively in areas of moderate to high transmission [15]. However, while 30% reduction of severe malaria is achieved, the protective efficacy of RTS,S is still moderate and short-lived. A 39% reduction in overall malaria incidence in a Phase III clinical study conducted in children who received four injections was achieved over a follow-up period of 48 months [16, 17]. On the other hand, the R21 vaccine was shown to reduce symptomatic cases of malaria by 75% during the 12 months following a 3-dose series. This high efficacy is similar to the efficacy demonstrated when RTS,S is given seasonally (66%) [15]. Therefore, a next generation malaria vaccine development approaches

that complement RTS,S and R21 would be essential to further strengthen malaria control measures in the future.

The clinical presentation of malaria is caused by asexual blood stage parasites and immuno-epidemiological studies have shown that people living in malaria endemic areas can acquire immunity to clinical disease with time and age after repeated episodes of infection [18]. This protection is very likely based on the development of asexual blood stage parasite binding antibodies as has been demonstrated in the proof of concept study of passive antibody transfer in which transfusion of purified Immunoglobulin G (IgG) from malaria-immune adults to juvenile clinical malaria patients drastically reduced blood stage parasitemia [19]. A number of follow-up immuno-epidemiological studies have strongly suggested that antibodies targeting the proteins expressed on the merozoite surface confer protective immunity against asexual blood stage parasites [20]. Therefore, the idea of targeting the step of malaria parasite invasion of RBCs as an anti-malarial strategy has gained strong scientific support over many years [21]. Based on a number of biological, functional, epidemiological and immunological studies investigating MSP-1, this protein is currently considered a prime candidate antigen for vaccine development against malaria [21].

So far, vaccine development efforts have focused on the C-terminal p42 or p19 fragments of MSP-1. Preclinical studies in the non-human primate Aotus monkey model demonstrated that immunization with p42 formulated with Freund's adjuvant, Montanide, ISA720 or AS02A, can elicit partial protection against *P. falciparum* blood stage challenge with the homologous parasite strain [22]. Level of protection depended on the adjuvant used and correlated with the Enzyme-Linked Immunosorbent Assay (ELISA) titres and invasion inhibitory activity of antibodies elicited by the vaccine formulation [22].

Phase 1 and 2 trials in humans indicated that recombinant p42 formulated with AS02 adjuvant was immunogenic and elicited antibodies that recognized native MSP-1 [23, 24]. However, immunization of individuals residing in Africa with p42 formulated with AS02 did not protect against natural challenge in the field [25].

The lack of protection observed in these studies was most likely due to the inability of this vaccine formulation to elicit high-titre and functional, growth inhibitory antibodies against blood stage parasites [24]. Importantly, additive effects of inhibitory antibodies targeting all MSP-1 subunits, as well as some associated MSPs, have been shown to inhibit parasite growth in RBC cultures [26].

A recently completed Phase Ia clinical trial in Heidelberg, Germany, reported promising immunogenicity of a full-length recombinant MSP-1 "heterodimer" composed of two peptide chains corresponding to the N- and C-terminal fragments of MSP-1, namely p83/30 and p38/42, respectively [27]. Importantly, this complex seems to assemble similar to the native MSP-1 complex, raising the possibility of inducing broadly reactive antibodies by vaccination that will cross-react with the native, merozoite expressed MSP-1 resulting in enhanced anti-parasite immunity [28].

2.3 General treatment/ Administration

For this Phase Ib study, the same formulation tested in the previous Phase Ia study in Heidelberg, Germany will be used [27]. Sumaya Biotech GmbH & Co.KG will provide the MSP-1 malaria antigen (drug product) and corresponding adjuvant (Glucopyranosyl Lipid Adjuvant-Stable Emulsion (GLA-SE) shipped from Access to Advanced Health Institute (AAHI), Seattle), while the control for the vaccination, a rabies vaccine (Verorab®), will be purchased locally in Tanzania. The vaccinations will be administered through intramuscular injection (i.m.) in the deltoid muscle.

2.4 Efficacy

As the previous Phase Ia trial was a first-in human study, no efficacy endpoints were defined, and therefore there are currently no data on the efficacy of the vaccine.

2.5 Scientific justification - Pharmacodynamics & Pharmacokinetics

In accordance with the *Guideline on clinical evaluation of vaccines* (EMEA/CHMP/VWP/164653/2005) [29], pharmacodynamics and pharmacokinetic studies are applicable if the vaccine contains novel adjuvants or excipients. This is not the case for this study.

However, immunogenicity studies characterising the immune response to the vaccine (as described below) essentially comprise pharmacodynamic studies for a vaccine.

2.6 Immunogenicity studies

Seroconversion and anti-MSP-1 IgG and IgM antibody titres

In the previous Phase Ia study, all 24 participants who received the vaccine seroconverted compared to the placebo (n=4) and the Glucopyranosyl Lipid Adjuvant-Stable Emulsion (GLA-SE) adjuvant-only groups (n=4), where an increase in MSP-1 specific antibody (IgG and IgM) titres was not observed.

The MSP-1 specific IgG and IgM antibody titres peaked around four weeks after the third immunization, exceeding the titres found in semi-immune individuals from malaria endemic areas. The fourth immunization administered 4 months after the 3rd vaccination boosted the declining MSP-1 antibody titres. Following the 4th immunization, specific IgG antibodies persisted for at least 6 months at levels seen in malaria semi-immune individuals. Long-lasting MSP-1 specific antibody titres were also observed for IgM.

There were no significant differences between the three dose levels (25 µg MSP-1, 50 µg MSP-1 or 150 µg MSP-1), regarding the IgG or IgM titres or their time courses. However, participants who received only three immunizations instead of four, generally had lower MSP-1 specific IgM and IgG levels 12 months after the first immunization irrespective of the dose.

Antibody-dependent respiratory burst (ADRB)

In the evaluation of the Phase Ia study, the ADRB assay was only assessed at baseline and four weeks post 3rd and 4th vaccination. The *in vitro* assay showed that the IMP could induce antibodies indicating its ability to stimulate immune effector cells and neutrophil granulocytes upon opsonization of merozoites. The arithmetic mean values of the amount of reactive oxygen species (ROS) for all three groups of vaccinated participants increased from baseline to four weeks post vaccination.

Growth inhibition assay (GIA)

In the previous Phase Ia study, no growth inhibitory activity could be shown in the GIA by analysing the purified IgG collected four weeks post 3rd vaccination from 14 SumayaVac-1 (SUM-101) immunised participants.

ELISpot investigations

In the completed Phase Ia study, enzyme-linked immunosorbent spot-forming cell assay (ELISpot) assays were performed at baseline and 3 and 6 months post 1st vaccination and prior to 4th vaccination.

To investigate MSP-1 specific T-cell responses, peripheral blood mononuclear cells (PBMCs) were exposed to full-length MSP-1 (as a recall antigen) in cultured interferon gamma (IFN-γ) ELISpot assay. Results from the selected participants showed significant increase in cultured ELISpot responses at 4 weeks post 3rd vaccination and remained stable in 5 out of 6 participants for more than 4 months. One participant had a decrease in PBMCs in recall stimulation at 6 months post 1st vaccination.

Those results suggest that as a next step, CHMI studies could help evaluate the efficacy of the vaccine candidate induced immunity against a homologous *P. falciparum* strain.

2.7 Safety and tolerability

In the previous Phase Ia clinical trial, the vaccination was generally well tolerated. There were no serious adverse events (SAEs), no dose-limiting toxicities, and no events resulting in permanent disability or premature withdrawal from the study. There was further no pattern of adverse events (AEs), suggestive of off-target effects. A total of 562 AEs occurred in the 32 participants with local injection site reactions exhibiting the highest frequency.

Laboratory values did not show any clinically relevant pattern of change except for an increase in C-reactive protein (CRP, a marker for systemic inflammation) in the first days after each vaccination. In all vaccination observation cycles, 93% of AEs were of mild nature and transient. Only two severe events occurred, which were assessed as being not related to the vaccination. One event was a hypertension grade III (according to the Common Terminology Criteria for Adverse Events 4.0 (CTCAE)) prior to vaccination, which resolved without treatment and was attributed to the stress of the anticipated vaccination. The other was a tendon rupture occurring 50 days after the last vaccination. Overall, tolerability was comparable in all dose groups (25 µg MSP-1, 50 µg MSP-1 or 150 µg MSP-1) and the GLA-SE group, and the vaccine did not show any dose-dependent adverse effects. In the post-trial follow-up, one participant showed a moderate event of a transient inflammatory disease, and one severe adverse event occurred, which was an unrelated accidental injury from sports. All other post trial contacts did not reveal any clinically relevant adverse events.

2.8 Toxicity

The MSP-1 protein is produced in *Escherichia coli (E.coli)* bacteria and supplied with >99% purity under good manufacturing practice (GMP) conditions and as stable substance. Examination in Sprague-Dawley rats at a dose >200 fold (based on body weight) of the intended highest human dose and >15 fold for *Aotus griseimembra* monkeys did not reveal any compound-related findings [26].

2.9 Justification for the dosage and dosage plan, and the length of treatment

In the Phase Ia clinical trial, antibody titres were independent of the immunization dose (25 µg, 50 µg and 150 µg). It is unclear whether the lower doses would be suitable in a clinical setting and in the proposed Phase Ib trial. Individuals living in malaria endemic areas (adults and infants) might require a higher dose given their lower responsiveness to vaccination compared with healthy malaria naïve adults [30]. Thus, three monthly inoculations (to be done on D0, D28 and D56) with the 150 µg dose are planned.

3 Objectives and purpose

3.1 Study rationale and objectives

3.1.1 Study rationale

The proposed clinical trial is the second administration of this antigen and adjuvant combination to humans [27]. The trial will enrol healthy adults pre-exposed to the malaria parasite and will be the first administration of the IMP to healthy participants with some pre-existing immunity. The goal of the study is to test the vaccine candidate early in the development path in the population living in malaria endemic areas. This study will be performed in a randomised, double-blinded, controlled design to evaluate the safety, reactogenicity and immunogenicity of the candidate malaria vaccine, SumayaVac-1 (SUM-101) (MSP-1 with GLA-SE as adjuvant).

3.1.2 Primary objectives

- To evaluate the safety and reactogenicity of SumayaVac-1 (SUM-101) in healthy adults of African origin, previously exposed to the parasite in comparison to the Verorab® control vaccine.
- To evaluate the immunogenicity of SumayaVac-1 (SUM-101) in healthy adults of African origin previously exposed to the parasite in comparison to the Verorab® control vaccine.

3.1.3 Secondary objective

- To evaluate the SumayaVac-1 (SUM-101) vaccine-induced antibody levels, *in vitro* effector functions in participants having received SumayaVac-1 (SUM-101) versus rabies control (Verorab®) vaccine.

3.1.4 Exploratory objectives

- Comparison of SumayaVac-1 (SUM-101) induced immunoglobulin isotype distribution and duration between malaria pre-exposed and malaria naïve participants from the previous Phase Ia study in Heidelberg.
- Fine scale epitope mapping of SumayaVac-1 (SUM-101) specific antibodies using peptide arrays, to investigate humoral immune response at baseline with vaccine induced responses.
- Comparison of SumayaVac-1 (SUM-101) induced cellular immunity between malaria pre-exposed and malaria naïve participants from the previous Phase Ia study in Heidelberg including fine scale epitope mapping of MSP-1 specific CD4+ and CD8+ T cells.
- Investigation of the B- and T-Cell receptor repertoire before and after SumayaVac-1 (SUM-101) vaccination.
- Integrated transcriptome and immunoglobulin gene repertoire analyses of MSP-1 specific B-cells using single-cell technologies.
- Glycosylation patterns of MSP-1 specific functional antibodies.
- Investigate off-target IgG and IgM repertoire after SumayaVac-1 (SUM-101) vaccination using immunoproteomics.
- Investigate the structure of MSP-1 protein bound to functional antibodies by cryo-electron tomography to map conformational epitopes.
- To evaluate mechanisms of malaria specific antibody diversity generation, post-translational modification of antibodies and B- and T-cell memory generation and maintenance.
- *Ex vivo* assessment of changes in human peripheral blood transcriptome in participants having received SumayaVac-1 (SUM-101) versus Verorab® rabies vaccine.
- Description of γδ T-cell receptor repertoire, transcriptome, functional activity and phenotypes in participants having received SumayaVac-1 (SUM-101) versus Verorab® rabies vaccine.
- Investigate impact of presence of intestinal helminth infections on SumayaVac-1 (SUM-101) induced humoral immune response
- Investigate impact of presence of intestinal helminth infections on SumayaVac-1 (SUM-101) induced cellular immune response

3.2 Scientific justification and rationale

3.2.1 Scientific justification - study population

This study includes healthy men and healthy non-pregnant and non-breastfeeding women between 18 and 45 years old residing in Tanzania and of African origin.

As this is a Phase Ib clinical trial, healthy participants residing in a malaria endemic country are essential to evaluate the safety and reactogenicity in the target population.

Healthy participants of both sexes will be enrolled to generate data on both sexes. Women of childbearing potential (WOCBP) and men will be required to use a highly effective form of contraception (see section 11.3.2).

3.2.2 Scientific justification - vaccinations

The rationale for 3 immunisations results from preclinical studies in mice and rabbits indicating that 3 immunisations led to higher titres than 2 immunisations. Further, pyrogenicity studies conducted in rabbits with a dose equivalent to four times the human dose regimen did not highlight any safety issue [31].

The 0, 4 and 8 weeks immunisation schedule for the three vaccinations in the vaccination part of the study can later be easily integrated into the immunisation programme for childhood vaccines as recommended by the WHO.

Preferred site for routine vaccinations is by i.m. administration. Further, injection of small volumes like 0.5 ml into the deltoid muscle is a very frequently practiced vaccination procedure. After technically correct injection, the local AEs at injection site are generally mild and transient [32].

3.2.3 Scientific justification - Dose

150 µg MSP-1 protein was the highest dose used in the preceding Phase Ia trial with the same IMP in healthy, malaria-naïve participants and was shown to have good safety and tolerability [27]. This dose was also considered as the no-observed-adverse-effect-level (NOAEL) in toxicology studies in rats, which gives a safety factor of >200 considering the body weight of the study population. The dose of 150 µg MSP-1 protein is used in the current proposed study where the participants are pre-exposed to malaria and helminth infections resulting in a likely reduced immune responsiveness.

3.2.4 Scientific justification for deviation from Verorab® dosing schema

The dosing scheme proposed for Verorab® is generally the following: Three doses of 0.5 ml are administered at D0, D7 and D28 for vaccination. In order to maintain double-blinding, the dosing scheme for Verorab® will be the same as for the IMP (D0, D28 and D56).

The rabies vaccine, Verorab®, has been chosen as the comparator because participants will benefit from receiving the rabies vaccine as rabid animals reside in the study area and three doses of the rabies vaccine induces protective antibody levels. When the rabies vaccine is administered according to the recommended vaccination schedule (D0, D7, D21), nearly 100% of subjects attain a protective titre. High antibody titres have also been demonstrated with off-label immunization with the rabies vaccines. Among participants in England, Germany, France and Belgium who received two vaccinations one month apart, nearly 100% of the participants developed specific antibodies and the geometric mean titre for the group was 10 IU/ml [33, 34] well above the protective antibody titres >0.5 IU/ml. Our proposed vaccination schedule of D0, D28 and D56 is therefore expected to be successful in conferring protective immunity against rabies among the control participants. However, all participants will be advised to seek medical attention immediately if bitten or scratched by a potentially rabid animal.

3.2.5 Scientific justification- retrospective helminth infection analysis

The potential impact of prevalent worm infections on vaccine immunogenicity and efficacy has gained increasing attention. Evidence has been generated over the past years that active helminth infections are associated with lower responses to a wide range of vaccination approaches including tetanus toxoid (subunit vaccine), Bacille Calmette-Guerin (live attenuated vaccine) and cholera (live attenuated vaccine) [35]. Vaccination of helminth-infested participants resulted in lower vaccine-induced T-cell proliferation and Type 1 T helper (TH1) cytokine production but increased immune-regulatory cytokine production like interleukin 10 (IL-10) [36-38]. Hence, a retrospective analysis on stool and urine samples collected at baseline (D0 before 1st vaccination), at D28 (before 2nd vaccination), D56 (before 3rd vaccination) and D84 will be performed to correlate antibody responses to any potential active helminth infection at that time.

4 STUDY DESIGN

This is a randomised, controlled, double-blind, parallel group, single center Phase Ib trial to assess safety, reactogenicity and immunogenicity of SumayaVac-1 (SUM-101) in healthy, malaria pre-exposed participants of African origin aged 18-45 years.

The vaccination schedule for the study is designed as follows (**Figure 1**):

In total, 40 participants will be enrolled (male and female). 20 participants will be randomised to receive three monthly inoculations (on D0, D28 and D56) with the IMP, SumayaVac-1 (SUM-101), and 20 participants will be randomised to receive the registered rabies vaccine (Verorab®) as controls. For operational reasons the participants in the vaccinations part will be divided in two groups of 20 participants:

- **Group 1** will have a sentinel subgroup (2 SumayaVac-1 (SUM-101) & 1 Verorab® rabies vaccine) where the 48 hours post vaccination safety data will be analysed (see chapter

4.5.2) before the remaining 17 participants (8 SumayaVac-1 (SUM-101) & 9 rabies vaccine) of group 1 receive their first vaccination.
- **Group 2** will be composed of 20 participants (10 SumayaVac-1 (SUM-101) & 10 Verorab® rabies vaccine). All visits in group 2 will be shifted by at least 3 weeks compared to group 1 to create minimal overlap of study related activities.

After each vaccination (done on D0, D28 and D56), the participant will stay for at least 2 hours at the facility before being discharged. The participant will be called by phone or visited at home (if needed) daily until 6 days post vaccination for follow-up.

For all the participants, on-site vaccination follow-up visits will occur at 7, 14 and 28 days after each vaccination (done on D0, D28 and D56).

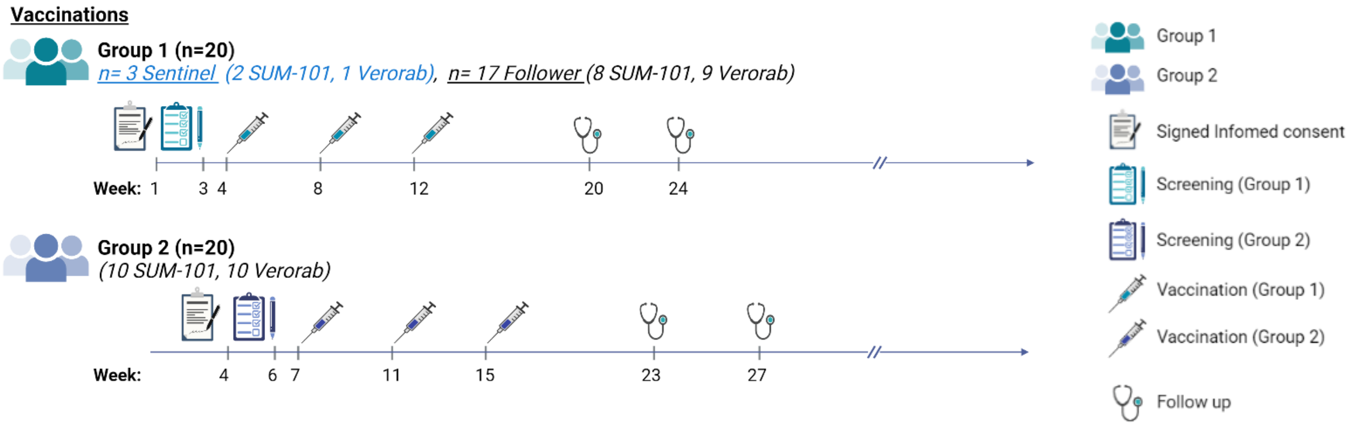

FIGURE 1 – STUDY DESIGN.
Time is indicated in calendar weeks from start of the study (not from randomisation for individual participants).

4.1 Primary and secondary endpoints

4.1.1 Primary endpoints

The following endpoints will be assessed in all participants to evaluate the safety and reactogenicity of SumayaVac-1 (SUM-101):

- Local and systemic solicited adverse events (AEs) at least possibly related to IMP recorded after each vaccination (done on D0, D28 and D56) up to 7 days later.
- Local and systemic unsolicited reactogenicity recorded after each vaccination (done on D0, D28 and D56) up to 28 days later.
- Any SAE occurring after the first vaccination until the participant's last visit.
- Changes in laboratory safety parameters between baseline (D0 before 1st vaccination) to 28 days after each of the vaccinations.
- Changes in laboratory safety parameters between values just prior to each vaccination (on D0, D28 and D56) and values 28 days after that vaccination.

The following endpoints will be measured in all participants in sera collected at D0 pre-vaccination, D28 (W4), D56 (W8), D84 (W12), D112 (W16) and D140 (W20), to evaluate the humoral immunogenicity:

- Longevity of antibody responses to SumayaVac-1 (SUM-101) by ELISA.
- Fold change of antibody responses to SumayaVac-1 (SUM-101) in comparison to baseline (D0 pre-vaccination) by ELISA.

4.1.2 Secondary endpoints

The following endpoints will be measured in the sera or blood of all participants, at D0 pre-vaccination, D28 (W4), D56 (W8), D84 (W12), D112 (W16) and D140 (W20):

- Evaluation of the opsonic phagocytosis activity.
- Evaluation of complement fixation, activation and/or membrane attack complex (MAC) formation.
- Evaluation of antibody-dependent respiratory burst (ADRB) activity.
- Evaluation of antibody-dependent cellular cytotoxicity (ADCC-NK cells) activity.
- Evaluation of immune-mediated growth inhibition activity on a panel of *P. falciparum* lines.

The following endpoint will be assessed among SumayaVac-1 (SUM-101) participants only:

- Comparison of MSP-1 IgG antibody concentrations by ELISA between malaria pre-exposed participants in the current study and malaria naïve participants from the previous Phase Ia study in Heidelberg.

4.1.3 Exploratory endpoints

- Comparison of SumayaVac-1 (SUM-101) induced immunoglobulin isotype distribution and duration between malaria pre-exposed and malaria naïve participants from the previous Phase Ia study in Heidelberg.
- Fine scale epitope mapping of SumayaVac-1 (SUM-101) specific antibodies, using peptide arrays, to investigate baseline humoral responses and vaccine induced humoral responses.
- Comparison of SumayaVac-1 (SUM-101) induced cellular immunity between malaria pre-exposed and malaria naïve participants from the previous Phase Ia study in Heidelberg, including fine scale epitope mapping of MSP-1 specific CD4+ and CD8+ T cells.
- Investigation of the B- and T-Cell receptor repertoire before and after SumayaVac-1 (SUM-101) vaccination.
- Cellular immune responses to SumayaVac-1 (SUM-101) in all participants by (i) ELISpot assays using total PBMCs, (ii) SumayaVac-1 (SUM-101) specific cells characterised by flow cytometry-based immunophenotyping using intracellular cytokine staining (ICS), (iii) functional gene expression analysis, and/or other assays to be defined; at D0 pre-vaccination and D56 (W8), D84 (W12), D112 (W16) and D140 (W20).
- Integrated transcriptome and immunoglobulin gene repertoire analyses of MSP-1 specific B-cells using single-cell technologies.
- Glycosylation patterns of MSP-1 specific functional antibodies.
- Investigate off-target IgG and IgM repertoire after SumayaVac-1 (SUM-101) vaccination using immunoproteomics.
- Investigate the structure of MSP-1 protein bound to functional antibodies by cryo-electron tomography to map conformational epitopes.
- *Ex vivo* assessment of changes in human peripheral blood transcriptome in participants having received SumayaVac-1 (SUM-101) versus Verorab® rabies vaccine.
- Description of γδ T-cell receptor repertoire, transcriptome, functional activity and phenotypes in participants having received SumayaVac-1 (SUM-101) versus Verorab® rabies vaccine.
- Investigate the impact of the presence of intestinal helminth infections on vaccine-induced humoral immune response by comparing the quality and quantity of SumayaVac-1 (SUM-101) specific antibody isotypes between helminth infected and non-infected participants at baseline.
- Investigate the impact of the presence of intestinal helminth infections on SumayaVac-1 (SUM-101) vaccine-induced cellular immune responses by comparing the quality and quantity of SumayaVac-1 (SUM-101) specific cytokine production and ICS results between helminth infected and non-infected participants at baseline.

4.2 Measures to minimize bias

4.2.1 Randomization

Participants will be randomised either to the SumayaVac-1 (SUM-101) vaccine or the Verorab® rabies vaccine which serves as a comparator.

For both groups the overall ratio will be 1:1. Three sentinel participants will be vaccinated first in Group 1.

The randomisation scheme is as follows:

- Group 1 (n=20): 3 sentinels (2 SumayaVac-1 (SUM-101), 1 Verorab®), followed by 17 participants (8 SumayaVac-1 (SUM-101), 9 Verorab®)
- Group 2 (n=20): 20 participants (10 SumayaVac-1 (SUM-101), 10 Verorab®)

An independent statistician (not part of the analysis team) at Swiss TPH will generate the allocation sequence using appropriate software. The independent statistician will provide the randomisation list in a secure manner to the site's pharmacist and will also maintain the randomisation list on a secure server with restricted access.

The pharmacist will allocate and prepare the vaccine for each participant according to the randomisation list (allocation of treatment to the next participant ID according to the sequential list) and label the prepared dose(s) with the participant ID and/or randomisation number. The randomisation list will be kept locked in the pharmacy and remain inaccessible to any other site staff. At the 2nd and 3rd vaccination, the pharmacist will refer to the randomisation list to confirm the vaccine allocated (SumayaVac-1 (SUM-101) or Verorab®) to each participant.

4.2.2 Double blinding of vaccination and unblinding

This trial will be conducted in a double-blinded manner. Namely, the participants, site staff, sponsor staff, the study monitor(s) and the trial statistician will be blinded to the treatment allocation. The independent statistician and the pharmacist are not blind throughout the study.

The pharmacist will be supplied with opaque, sealed envelopes (prepared by an independent person), each labelled with the corresponding randomisation number on the outside and containing inside the allocated treatment. These envelopes will be kept in the pharmacy with restricted access and handed out to clinical staff in case of emergency.

If necessary, a separate unblinded monitor may be used to perform drug monitoring and to check accountability during the conduct of the study. Procedures should be followed to avoid unblinding of other study personnel.

If required and indicated, advisor(s) independent from the study team may be appointed to review unblinded data to assist decision-making during Safety Monitoring Meetings.

If unblinding is required earlier in the interest of a participant's safety, the Investigator will discuss the matter with the Sponsor before opening the individual code-break envelope for that participant. In a medical emergency, the Principal Investigator (PI) or delegate may open the individual code-break envelope for that participant without prior consultation with the Sponsor. In that event, the PI or delegate will notify the Sponsor as soon as possible that the randomisation code has been broken for the respective participant.

If the code for a participant is broken, the reason for doing so will have to be fully documented and entered in source documents and the electronic Case Report Form (eCRF).

At the end of the study upon unblinding, the participants will be informed about the vaccination they received during the study. The participants who received SumayaVac-1 (SUM-101), will be offered a rabies vaccination. Further, upon unblinding, participants who were originally enrolled in the Verorab group and withdrew consent during the study or were early discontinued, will be offered to complete remaining series of rabies vaccine.

4.2.3 Masking of syringe

As the SumayaVac-1 (SUM-101) vaccine and the rabies control vaccine may not have the same appearance, in order to conduct the trial in double-blind manner as described in 4.2.2, the syringe (in both cases 0.5 ml) will be prepared and masked by the unblinded pharmacist before handing over to the nurse for administration. Further, since the preparation times for Sumayavac-1 (SUM-101) and Verorab are different, the pharmacists will ensure that they wait the same amount of time for each vaccine before handing it over to the nurses for administration. Details are described in the corresponding working instruction/standard operating procedure (SOP)/ manual.

4.3 Study duration and duration of participant's participation

The total study duration including the screening period (~4 weeks) combining Group 1 and 2 will be approximately 35 weeks.

The study duration for each participant will be ~24 weeks (4 weeks of screening period plus 20 weeks of 3 vaccinations administered monthly and two long-term follow-up visits).

4.3.1 Schedule of events

Study Visit (V)	V1	V2	V3		V4-V9	V10	V11	V12		V13-V18	V19	V20	V21		V22-V27	V28	V29	V30	V31	V32	UV
Day (D)/ Week (W)	-D35 to -D1		D0		D1-6	D7	D14	D28 (W4)		D29-34	D35	D42	D56 (W8)		D57-62	D63	D70	D84 (W12)	D112 (W16)	D140 (W20)	Anytime
	Screening		1st Vaccination					2nd Vaccination					3rd Vaccination								
	-35 to -1d		Pre-Vac -1d to 0d		1-6d [a]	7d	14d	28d	0d	1-6d [a]	7d	14d	28d	0d	1-6d [a]	7d	14d	28d	Vac1 + 4m	Vac1 + 5m	
Window	-35 to -1d		-1d	0d			± 2d	± 3d	± 3d			± 2d	± 3d	± 3d			± 2d	± 3d	± 7d	± 7d	
**Clinical Procedures**																					
Clinic Visit	●	●	●	●		●	●	●	●		●	●	●	●		●	●	●	●	●	●
Phone call/Home Visit [a]					●					●					●						
Informed consent	●																				
Vital Signs [b]	●		●	●●●●		●	●	●	●●●●		●	●	●	●●●●		●	●	●	●	●	●
Medical history	●	●	●																		
Prior/Concomitant medication	●	●	●	●	●	●	●	●	●	●	●	●	●	●	●	●	●	●	●	●	●
Contraception use	●	●	●			●	●	●			●	●	●			●	●	●	●	●	
Physical exam [c]	●		●	●		●	●	●	●		●	●	●	●		●	●	●	●	●	●
Bed nets use [d]		●	●			●	●	●			●	●	●			●	●	●	●	●	
ECG	●																				●
Eligibility [e]	●	●	●					●					●					●			
Randomisation				●														●			
Vaccination				●					●					●							
AE *Solicited*				●●●●	●	●			●●●●	●	●			●●●●	●	●					
AE *Unsolicited*				●●●●	●	●	●	●	●●●●	●	●	●	●	●●●●	●	●	●	●	●	●	●
**Lab Evaluations**																					
Pregnancy test (1 ml)	●		●					●					●					●	●	●	Based on need
Urinalysis	●		(●)f					●					●					●			
Haematology (1 ml)	●		(●)f			●	●	●			●	●	●			●	●	●			
Biochemistry (2 ml)	●		(●)f			●	●	●			●	●	●			●	●	●	●	●	
Serology (1 ml)	●																				
Urine and Stool			●					●					●					●			
Parasitology (2 ml) *qPCR*	●		●					●					●					●	●	●	
Parasitology (2 ml) *TBS*	●		●					●					●					●	●	●	
Immunology *Humoral (ml)*			27					27					27					27	27	27	
Immunology *Cellular (ml)*			25					25					25					25	25	25	
Exploratory (ml)			25					25					25			25		25	25		
Blood (ml)/ per visit	7		83			3	3	83			3	3	83			28	3	83	82	57	
Total blood (ml) in study	~ 521 ml																				

a. Daily phone calls or home visit if necessary

b. At screening 1 visit include anthropometrics (body weight and height). At D0 , D28, D56 Pre-Vac visits and D84 follow-up visit include body weight.

c. Complete at Screening 1 and focused at all other visits

d. Supplied at Screening visit 2

e. Eligibility at screening refers to review of inclusion/exclusion criteria to be enrolled for the study and eligibility at pre-vaccination visits is to review safety data to assess if participant can receive next vaccination

f. Repeated only if screening was done more than 7 days before

Legend: Informed consent; Screening; Vaccination; Phone follow up; Site follow up

4.4 Early termination of the study

Premature termination of this study may occur due to a Regulatory Authority (RA) decision, change in opinion of the Independent Ethics Committee (IEC) or Institutional Review Board (IRB), or drug safety problems.

Both the Sponsor and the Investigator reserve the right to terminate the study at any time before enrolment of the intended number of participants, but intend to exercise this right only for valid scientific or administrative reasons. Should early termination be necessary, both parties will arrange the procedures on an individual study basis after review and consultation. In terminating the study, the Sponsor and the Investigator will assure that adequate consideration is given to the protection of the participants' interests.

Reasons for early termination by the Sponsor(s) may include but are not be limited to:

- Safety issues with the IMP (see stopping rules in section 4.5).
- Low enrolment rate.
- Protocol violations.
- Inaccurate or incomplete data.
- Unsafe or unethical practices.
- Following the recommendation of the Safety Monitoring Committee (SMC), RA or the IEC.
- Unforeseen events such as an endemic/ pandemic.

Reasons for early termination by the Investigator may include but are not be limited to:

- Ethical concerns.
- Lack of eligible healthy participants.
- When the safety of the participants is doubtful or at risk.
- Alterations in accepted clinical practice that make the continuation of a clinical trial unwise.
- Early evidence of harm from the experimental intervention.

In the event that a study is terminated early, either by the Sponsor or by the Investigator, the Investigator must:

- Complete all documentation and eCRFs to the fullest extent possible.
- Destroy or return all test articles (IMP including adjuvant) according to the Sponsor's instructions and local regulations.
- Return all related study materials to the Sponsor.
- Answer all questions from the Sponsors or their representatives related to participant data collected before the termination of the study.
- Ensure that participants enrolled in the study who had not yet reached 28 days post last vaccination follow-up visit are followed up with the necessary medical care.
- Provide in writing the reasons for the decision to RA, IEC, IRB and the Sponsor if needed.

4.5 Stopping rules

4.5.1 Study pause at any time point

In case one of the following stopping rule is met at any time point in the study, the study will be temporarily interrupted (see section 5.5.2), and the SMC will do an ad-hoc assessment and conduct a thorough review before a decision is taken to continue or terminate the study.

- Any SAE possibly, probably or definitely related to the SumayaVac-1 (SUM-101) vaccination.
- Any cardiac event that does not meet the criteria for an SAE.
- On the advice of local safety monitor, investigator, sponsor, SMC, Regulatory Authority and Ethics Committee.

4.5.2 Study pause at defined time-points

There are stopping rules (**No-Go criteria**) defined at two time-points (see below) in the course of this study. If one stopping rule (No-Go criterion) is met, the PI will be asked to break the blind (see section 4.2.2) for the concerned participant(s). The No-Go criteria are only applicable if the concerned participant(s) received SumayaVac-1 (SUM-101).

The SMC will check at defined time-points (see below) that none of the No-Go criteria are met and document the recommendation as described in the SMC charter.

No-Go criteria before vaccination of followers in group 1

If one of the following criteria is applicable to the safety data of 48 hours after 1st vaccination of the sentinel sub-group (n=3), the study will be interrupted and further investigations are needed before any further vaccination is administered:

- Any SAE possibly, probably or definitely related to the SumayaVac-1 (SUM-101) vaccination.
- Any Grade 3 or 4 AE possibly, probably or definitely related to the vaccination within 48 hours after the 1st vaccination persisting at Grade 3 (or higher) for >24 hours.

No-Go criteria before 1st vaccination of group 2 or 2nd vaccination of group 1

If one of the following criteria is applicable to the group 1 safety data at day 14, the study will be interrupted after the 1st vaccination of group 1 (n=20) and further investigations will be needed before group 2 can receive the 1st vaccination or group 1 can further proceed to the 2nd and 3rd vaccinations.

- Any SAE possibly, probably or definitely related to the SumayaVac-1 (SUM-101) vaccination.
- >4 participants (>20% of group 1) experience the same grade 3 or 4 AE possibly, probably or definitely related to the vaccination beginning within 48 hours after the 1st vaccination and persisting at grade 3 (or higher) for >48 hours.

4.6 Temporary contraindications for continuing vaccination

Contraindications for vaccination leading to a temporary pause in the vaccination schedule include:

- The participant is ill on the day of vaccination.
- The participant has abnormal laboratory parameters that have been determined to be clinically significant.
- The participant has malaria or has taken or is taking medication for malaria or for another acute illness that may affect the vaccine.
- On request of the participant.
- Other reasons based on the investigator’s clinical judgement.

If a temporary pause in the vaccination schedule for a particular participant occurs, the investigator should notify the sponsor immediately, and the decision to continue the vaccination will be assessed on a case by case basis.

Continuing vaccination after a temporary pause may require repeating some or all of the pre-vaccination investigations if the pause is longer than the allowed window. The extent of the repeated investigations will be agreed in advance with the sponsor.

5 Selection of the study participants

5.1 Study setting

The study will be conducted at the Bagamoyo Clinical Trial Facility (BCTF) in Tanzania where screening, enrolment and follow-up of the healthy participants will be done. BCTF is part of the IHI

Bagamoyo branch and therefore has access to the institutional research clinical laboratory with adequate facilities for the conduct of safety and several other assessments.

5.2 Recruitment

After approval has been received by relevant Ethics Committees to work in the community, local authorities will be informed about the study in order to receive permission to approach the community. Adult residents of Bagamoyo town and surrounding areas of Bagamoyo district will be recruited using a rolling recruitment, screening and enrolment process. In addition, healthy participants from the site's participant database may be approached and invited if they meet the study profile and are interested. This process will continue until all study participants have been enrolled. The recruitment strategies are based on the experience from previous trials conducted by the clinical team in BCTF aiming to achieve adequate participant enrolment to reach target sample size. The estimated recruitment period is 3 months. Study advertisement (approved by IEC) will be used to invite targeted and potential community members to attend sensitization meetings which will be conducted at designated community areas. Locally appropriate methods of advertisement will be used, such as flyers, announcements via speakers or local radio stations. In addition to local community members, these sensitization meetings may also involve ward leaders, community health workers and key opinion leaders in the community. To ensure adequate delivery of the information, meetings will primarily be conducted in Swahili.

At the community-based sensitization meetings, Investigators will explain the current burden of malaria and the need for an adequate malaria vaccination will be discussed in a culturally appropriate manner. Investigators will also explain the concept of the clinical trial and the community members will be made aware of the known and potential risks and benefits of the IMP to be used in this trial.

The outline of the proposed trial including the rationale, aims and study procedures such as consenting, screening process and randomisation will be shared. It will also be made clear that the study will require screening for malaria and other chronic conditions such as Human Immunodeficiency Virus (HIV) and hepatitis. Further, it will be made clear that the screening processes will include a range of criteria to be checked covering clinical status as well as the ability to comply with the protocol and ethical requirements. Moreover, it will be explained that exclusion from participating in the trial does not mean the potential participant is unhealthy, nor will their provision for clinical care, if relevant, be affected in any way. It will be made clear that the confidentiality of all this information will be protected.

Opportunity will be provided for the community members to ask questions and receive responses from the Investigators. Potentially interested participants will be asked to register their names at the end of the meetings with the study staff who will complete preliminary registration forms for all those interested in a possible participation. These registries will be used by the study team to complete individual trial participation checklists and generate list of potential participants who will be invited to the clinic based sensitization meeting at BCTF. All data will be kept confidential at all times, by the Investigator and relevant clinicians.

During the clinic-based sensitization meetings, Investigators will provide further details via the participant information sheet on the mechanism of the IMP, risks, benefits and procedures such as blood sampling, in-patient stay, follow-up visits and the need for efficient contraception. Study team members will discuss the existing understanding of the participants with regards to the study and will share more detailed information and clarify any questions in Swahili.

At the same occasion they will confirm that the participants are able to read and write (to ensure adequate informed consent for participants). Those interested and considered as potential participants for screening will be provided with the participant information sheet to read at home and for opportunity to discuss with their family members. Potential candidates will be contacted and invited to a screening visit at the BCTF where they will undergo the individual informed consent process prior to any study screening procedures.

To ensure that enrolment goals are met, this recruitment plan allows for longitudinal screening of the participants. In addition, longitudinal compliance with study visits and procedures will be encouraged through the provision of a study calendar to participants, using multiple contact methods (phone calls, home visits by community mobilizers, having names and phone numbers of close contacts), and free transportation to the BCTF if needed.

5.3 Inclusion criteria

1. Written informed consent obtained before any study procedure.
2. Literate participants aged 18-45 years of African origin.
3. Female participants practicing contraception from 4 weeks before 1st immunization and both female and male participants willing to practice contraception up to 12 weeks after the last immunization.
4. Available to participate in follow-up for the duration of study.
5. Contactable by phone during the whole study period.
6. At least two years residence in the Bagamoyo district or nearby districts in Coastal and Dar-es-Salaam regions and planning to reside there for at least 9 more months.
7. Agreement to provide personal contact information and contact information of another household member or close friend.
8. Female participants must be willing to avoid pregnancy if selected for participation in the trial and to undergo multiple serum pregnancy testing.
9. Confirmation of understanding of design, procedures, risk and benefits of the study in a test with maximum of two attempts.
10. General good health based on assessment of medical history and clinical examination.

5.4 Exclusion criteria

1. Previous participation in any malaria vaccine trial in the last 3 years.
2. Participation in any other clinical trial involving investigational medicinal products within 30 days prior to the screening assessment.
3. Previous history of drug or alcohol abuse interfering with normal social function within one year prior to enrolment.
4. Previous vaccination with a rabies vaccine.
5. Intake of chronic medication, especially immunosuppressive agents (steroids, immunomodulating drugs) during the 13 weeks preceding the screening visit or during the study period.
6. Known hypersensitivity to any of the vaccine components (adjuvant or protein) or anti-malarial treatments.
7. Body mass index (BMI) of <18 or >30 Kg/m^2.
8. Participants unable to be closely followed for social, geographic or psychological reasons.
9. Any vaccination from 4 weeks prior to the 1st vaccination and (none planned) up to 6 weeks after the 3rd vaccination.
10. Symptoms, physical signs or laboratory values suggestive of systemic disorders, including renal, hepatic, cardiovascular, pulmonary, skin, immunodeficiency, psychiatric and other conditions, which could interfere with the interpretation of the trial results or compromise the health of the participants.
11. Abnormal electrocardiogram (ECG) on screening: pathologic Q wave and significant ST-T wave changes, left ventricular hypertrophy, clinically significant arrhythmias, left bundle branch block, secondary or tertiary A-V heart block.
12. Any clinically significant laboratory values at screening outside of normal ranges for study participants.
13. Malaria positivity at screening (Microscopy or qPCR positive).

14. Positive HIV, Hepatitis B (HBV) or Hepatitis C (HCV) tests.
15. For females: Positive pregnancy test or actively breast feeding.

5.5 Criteria for discontinuation of study

5.5.1 Withdrawal and discontinuation of individual participants

In accordance with the principles of the current version of the Declaration of Helsinki and any other applicable regulations, a participant has the right to withdraw from the study at any time and for any reason and is not obliged to give his or her reasons for doing so.

If a participant withdraws consent, no further evaluations should be performed and no attempts should be made to collect additional data, with the exception of safety data, which should be collected if possible and in accordance with the participant's agreement. The Sponsor may retain and continue to use any data collected before any withdrawal of consent. However, if the participant explicitly asks the Investigator to destroy all identifiable samples taken from the participant and does not allow to analyse his/her data, the Investigator will accept and will ensure to initiate all necessary steps to do so. Participant withdrawal and any agreements made with the participant will be documented in the participant's file.

The Investigator may discontinue a participant from the study at any time in the interest of the participant's health and well-being. In addition, the participant may be discontinued for any of the following reasons:

- Administrative decision by the Investigator
- Participant non-compliance with study requirements
- Any event which requires discontinuation of the study involvement or results in inability to continue to comply with study procedures
- Recommendation by the SMC (see section 11.4)

Prior to discontinuing a participant from study treatment, it is recommended that the Investigator contacts the Sponsor's Medical Monitor and forwards appropriate supporting documents for review and discussion. The decision to discontinue a participant remains the responsibility of the treating physician and will not be delayed or refused by the Sponsor. If a female participant is discontinued due to pregnancy, she will be referred to routine pre- or ante-natal care. Further, since there are unknown risks for the participant and the unborn child, they will be followed up for safety purposes by reviewing their ante-natal assessment reports until delivery or after the end of pregnancy.

The reason(s) for discontinuation will be recorded in the eCRF and source documents.

Participants who discontinue the study before receiving any study IMP (or control, according to randomisation) will be replaced. Participants who discontinue the study after having received at least one dose of study IMP (or control, according to randomisation) will not be replaced.

If, for any reason, a participant is discontinued from the study before his/her last visit, the Investigator is required, as feasible, to perform the safety procedures planned for the end of treatment. The discontinued participant will be asked if they agree to come for follow-up visits for safety evaluations (clinical and laboratory) as determined reasonable by the Investigator (e.g. weekly or monthly depending on the condition), and as a minimum for follow-up visits on D112 and D140.

5.5.2 Discontinuation because stopping rule is met

If one of the stopping rules is met (see section 4.5), the study will be temporarily interrupted and the Sponsor will request an ad hoc meeting of the SMC to review the event(s) and associated safety data.

After the SMC review and communication of recommendation, Swiss TPH, Sumaya Biotech and the PI will decide if a longer term safety hold is needed for further investigation or the study can be continued. The Ethics Committees and RAs need to be informed accordingly.

The temporary interruption of the study means that no further vaccinations can be performed. Remaining study procedures related to safety should be completed. Remaining study procedures related to research assays, including collection of samples, may be continued if the participant agrees and Swiss TPH, Sumaya Biotech and the PI confirm that it should be done.

6 TREATMENT OF PARTICIPANTS

6.1 Identity of investigational products

All 40 participants will be randomised (1:1) to either 150 µg MSP-1 drug product + 5 µg GLA-SE adjuvant (SumayaVac-1 (SUM-101) vaccine) or the comparator rabies vaccine (Verorab®)

6.1.1 Experimental intervention vaccination

Proprietary name	SumayaVac-1 (SUM-101)
International Nonproprietary Name (INN)	Not applicable
ATC code, if officially registered	Not applicable
Manufacturer MSP-1 malaria antigen	Praxis Pharmaceuticals, Vitoria, Spain
Manufacturer GLA-SE	Access to Advanced Health Institute (AAHI), Seattle, USA
Manufacturer 0.9% NaCl	B. Braun Melsungen AG
Mode of administration	Intramuscular injection (i.m.) in the deltoid muscle
Storage instructions	Lyophilized protein 2 – 8°C<br>Ready to use solution in 0.9% NaCl and adjuvant at room temperature for up to 48 h (chemically stable)
MSP-1	150 µg lyophilized MSP-1 protein in 2 ml stoppered vials
Adjuvant	GLA-SE (Glucopyranosyl Lipid Adjuvant-Stable Emulsion), 2 ml vials
Product after reconstitution	150 µg MSP-1 dissolved in 0.9% NaCl and emulsified with 250 µl of adjuvant GLA-SE

6.2 Comparator

The comparator Verorab® is an inactivated rabies virus vaccine.

It is supplied in a single-dose glass vial containing lyophilised powder (≥ 2.5 IU) with 0.5 ml of solution (sodium chloride 0.4%) in glass syringe.

The diluent is a solution of 0.4% sodium chloride and each glass vial contains maltose and human albumin as excipients.

The marketing authorisation holder is Sanofi Pasteur Ltd, Bangkok, Thailand.

The storage condition for the vaccine is 2-8°C and the reconstituted vaccine is a homogeneous, limpid solution without any particles in suspension.

For adults, the route of administration is i.m. injection in the deltoid region.

6.3 Packaging, labelling and supply

MSP-1 drug product will be labelled, packed, and released by NextPharma GmbH, Goettingen, Germany under the existing manufacturing license.

The adjuvant is imported, labelled, packaged and released by AAHI under the existing manufacturing and import authorization.

The trial medication and control will be labelled according to Tanzanian requirements of Tanzania Medicines and Medical Devices Authority (TMDA).

6.4 Storage conditions

The vaccine must be transported and stored at 2-8°C. The IMP and control vaccine (Verorab®) should not be used beyond the expiry date indicated on the label. The IMP and control vaccine (Verorab®) must be kept in a secure and limited access storage area (only accessible to authorized persons).

The storage conditions, including the temperature, at the study site must be monitored by the study personnel and appropriate records must be available. Any temperature excursion should be recorded and reported to the Sponsor immediately.

6.5 Administration of experimental and comparator interventions

6.5.1 Experimental intervention - SumayaVac-1 (SUM-101) vaccine

Once randomised to either the SumayaVac-1 (SUM-101) or the rabies control vaccine, the participant will always receive the same dose of the same compound (150 µg MSP-1 protein dissolved in 0.9% NaCl with 250 µl of adjuvant GLA-SE or Verorab®) and there will be no dose adjustments foreseen. Both the SumayaVac-1 (SUM-101) and the rabies control vaccines will be applied as an i.m. injection in the deltoid muscle of the non-dominant arm. The three vaccine doses will be applied in 4 week intervals as described in the schedule of assessments (see section 4.3.1).

6.6 Dose modifications

The doses are defined and described in section 6.1 for the vaccinations. No dose modifications are foreseen.

6.7 Compliance with study interventions

The pharmacist will prepare the vaccine according to randomisation for each participant and label it with the participant ID. Additional details regarding the labelling and dispensing will be described in the Pharmacy Manual.

The vaccinations will be administered at the study site under supervision of site personnel so full compliance is expected. Any non-compliance (such as participant refusal) will be documented accordingly.

6.8 Concomitant interventions (treatments)

Medications taken before the first vaccination will be documented as a prior medication. Medications taken after the first vaccination (e.g. to treat any AEs) will be documented as concomitant medications.

For any disease present at the screening visit, the investigator needs to assess:

- if part of the exclusion criteria.
- if either the disease or its treatment are expected to have any effect on the outcome measures used in this study trial.
- if either the disease or its treatment are expected to interfere with the study IMP.

If none of the above criteria are applicable, the concomitant treatment might be acceptable.

The following drug groups are **not permitted** as concomitant medication:

- In general, any regular systemic medication except contraceptives
- In particular, regular intake of the following drugs:
    - Immunosuppressants (e.g. systemically available glucocorticoids, calcineurin inhibitors)

  - Immunomodulators
  - Cytotoxic or cytostatic drugs

Prophylactic use, intake of more than 5 days or at a dose higher than recommended (refer to medication leaflet) of:

  - Anti-histamines
  - Analgesics

The following drug groups are permitted after individual counselling as concomitant medication:

- Topical medication (except if applied at the site of injection)
- Occasional, sporadic use of drugs not interfering with the immune system
- Hormonal contraceptives
- Food supplements (e.g. Vitamins)

Use of any prior and concomitant medication will be recorded in the source data and eCRF with the following information:

- Reason for treatment
- Name of the drug
- Route of administration
- Dose administered
- Duration of treatment

6.9 Experimental Intervention accountability

The MSP-1 drug product and GLA-SE adjuvant will be stored at the site in accordance with Good Clinical Practice (GCP), GMP requirements and the instructions given by the Sponsor and will be inaccessible to unauthorized personnel. Special storage conditions and a complete record of batch numbers and expiry dates will be kept in the Sponsor's Trial Master File (TMF) and the site-specific elements of this information will be available in the Investigator Site File (ISF). On the day of receipt, the responsible study personnel will confirm receipt of MSP-1 drug product and GLA-SE adjuvant as per the instructions supplied. The personnel will use MSP-1 drug product and GLA-SE adjuvant only within the framework of this clinical study and in accordance with this protocol once the Sponsor has approved the use.

The pharmacist must maintain appropriate documentation on accountability of SumayaVac-1 (SUM-101) and GLA-SE adjuvant including the following information:

- Delivered to the site
- Inventory at the site
- Use by each participant
- Returned or destroyed

6.10 Return or destruction of experimental Intervention

The unused MSP-1 drug product and GLA-SE adjuvant will be destroyed in accordance with the site's SOP and national law.

7 Study assessments

7.1 Procedures per visit

7.1.1 General order of assessments

For the study periods described below, when multiple procedures are scheduled at the same time-point(s) relative to dosing, the following chronology of events should be adhered to, where possible:

- Vital signs (axillary temperature, blood pressure (BP), heart rate (HR) and respiratory rate (RR)). BP and HR after 10 minutes rest in the supine position
- Physical examinations

- AE monitoring (spontaneous and solicited AE monitoring will include specific questioning for tolerability and safety)
- Laboratory safety blood samples (biochemistry, haematology)
- 12-lead ECG after 10 minutes rest in the supine position
- Urine and stool samples will be collected when possible

Every effort should be made to ensure that protocol-required tests and procedures are completed as described. However, it is anticipated that from time to time there may be circumstances outside of the control of the Investigator that may make it unfeasible to perform a certain test or assessment. In those cases, the Investigator must take all steps necessary to ensure the safety and well-being of the participant. When a protocol required test or assessment cannot be performed, the Investigator will document the reason for it and any corrective and preventive actions which he/ she has taken to ensure that required processes are adhered to as soon as possible. The Sponsor study team must be informed of these incidents in a timely manner.

7.1.2 Screening visit 1 (D-35 to D-1)

Written informed consent will be obtained prior to any study procedures.

All healthy participants will be screened within 35 days before randomisation to confirm that they meet the participant selection criteria for the study. Screening can take place over several days to give the healthy participants sufficient time for reflection. Malaria testing should not be done more than seven days prior to randomisation. Healthy participants who fail screening because they do not meet an inclusion criteria or meet an exclusion criteria temporarily can be re-screened at the Investigator's discretion. In this case, a new screening procedure will be completed (new participant ID, new informed consent, etc.). The previous participant ID must not be re-used.

The following assessments will be done to collect the baseline safety data, and to assess the eligibility for the study:

- Demographics and information for identification card (ID).
- Vital signs (axillary temperature, blood pressure (BP), heart rate (HR) and respiratory rate (RR)). BP and HR after 10 minutes rest in a supine position.
- Anthropometrics (body weight and height).
- Complete medical history including prior and concomitant medication (i.e., prescription or non-prescription drugs and dietary supplements taken).
- Review of contraceptive use.
- Full physical examination.
- Check preliminary eligibility.
- Pregnancy test: all women of childbearing potential will undergo a serum pregnancy test.
- Urinalysis: Urine dipstick for protein, glucose and blood.
- Blood sampling for clinical safety laboratory evaluations:
    - Haematology: haemoglobin, WBC including differentiation of eosinophils and neutrophils, platelets and haematocrit.
- Biochemistry: Alanine aminotransferase (ALT), Aspartate aminotransferase (AST), total bilirubin, creatinine and glucose (random). Troponin sample (at baseline) will be collected and stored to be run retrospectively if needed in case of a cardiac event. The sample collected at the time of the cardiac event will be compared with the sample collected at baseline.
- Blood sampling for serology (HIV, hepatitis B and hepatitis C).
- Blood sampling for exclusion of active clinical malaria by qPCR and TBS.
- 12-lead ECG. Healthy participants should rest in the supine position for 10 minutes before ECG measurements.
- Review of inclusion and exclusion criteria.

7.1.3 Screening visit 2 (D-35 to D-1)

Participants that passed screening visit 1, come for screening visit 2 to discuss the results from screening visit 1 and following assessments will be done:

- Discussion of findings from screening visit 1.
- Check of additional relevant medical history including prior and concomitant medication.
- Review of contraceptive use.
- Review of inclusion and exclusion criteria and determine eligibility to participate in the study.
- If eligibility criteria is met, distribute an insecticide-treated bed net and provide information on its use

7.1.4 Pre 1st Vaccination (D-1 to D0)

The following assessments will be done **before randomisation:**

- Confirmation of willingness to participate.
- Vital signs (axillary temperature, BP, HR and RR (BP and HR after 10 min rest in a supine position)).
- Body weight measurement.
- Check of additional relevant medical history including prior and concomitant medication.
- Reminder of contraceptive use.
- Focused physical examination which involves an examination of the body system(s) related to participant’s abnormal presentation.
- Reminder of insecticide-treated bed nets use.
- Pregnancy test: all women of childbearing potential will undergo a serum pregnancy test.
- Urinalysis: Urine dipstick for protein, glucose and blood (if previous testing has been done >7 days ago).
- Blood sampling for haematology and biochemistry laboratory evaluations (if previous testing has been done >7 days ago).
- Blood sampling for baseline Malaria testing by qPCR and TBS.
- Blood sampling for baseline humoral & cellular response.
- Blood sampling for baseline exploratory samples.
- Urine and stool collection for retrospective exploratory analysis.
- Review eligibility for vaccination.

7.1.5 1st, 2nd and 3rd Vaccination visit (D0, D28 and D56)

After the pre-1st vaccination assessments have been completed, the randomisation is performed (see section 4.2.1). Then the participant can be dosed intramuscularly in the deltoid muscle of the non-dominant arm. After vaccination, the participant is then accompanied to the waiting area where he/she will stay for the next 2 hours. The participants will be closely observed in this time for any direct adverse events.

After 15 minutes, 30 minutes and 1 hour following assessments will be done:

- Vital signs (axillary temperature, BP, HR and RR (BP and HR after 10 min rest in a supine position)).
- Assessment of possible solicited and unsolicited AEs.
- Inspection and palpation of the injection site.

After 2 hours following assessments will be done:

- Vital signs (axillary temperature, BP, HR and RR (BP and HR after 10 min rest in a supine position).
- Review of any concomitant medication.
- Focused physical examination which involves an examination of the body system(s) related to participant’s abnormal presentation.
- Assessment of possible solicited and unsolicited AEs.

If not contraindicated by an AE or any other reason, the participant can be released for home after the 2 hours assessment.

Following the 1st vaccination, the participants will be escorted home, so that the study staff know where the participant resides to help them trace a participant in event of a missed scheduled appointment.

7.1.6 1-6 days post 1st, 2nd and 3rd vaccination follow-up (D1-6, D29-34 and D57-62)

The participant is followed up daily after each vaccination and before the next site visit either by a telephone call and/ or if deemed necessary by the study team, by a home visit.

Following assessments are done each time during the follow-up:

- Assessment of possible solicited and unsolicited AEs.
- Follow-up of unresolved AEs.
- Review of any concomitant medication.

7.1.7 7 day site visit post 1st, 2nd and 3rd vaccination (D7, D35 and D63)

Participants come for an ambulatory visit to assess AEs and give blood samples for immunological analyses (only on D63). The visit includes the following procedures:

- Vital signs (axillary temperature, BP, HR and RR (BP and HR after 10 min rest in a supine position)).
- Review of concomitant medication.
- Review of contraceptive use.
- Focused physical examination which involves an examination of the body system(s) related to participant's abnormal presentation to assess solicited and unsolicited AEs.
- Reminder of insecticide-treated bed nets use.
- Capturing of solicited and unsolicited AEs.
- Follow-up of unresolved AEs.
- Blood sampling for haematology and biochemistry (ALT, total bilirubin and creatinine) laboratory evaluations.
- Blood sampling for exploratory samples (only on D63).

7.1.8 14 day site visit post 1st, 2nd and 3rd vaccination (D14, D42 and D70)

Participants come for an ambulatory visit to assess and follow-up on AEs. The visit includes following procedures:

- Vital signs (axillary temperature, BP, HR and RR (BP and HR after 10 min rest in a supine position)).
- Review of concomitant medication.
- Review of contraceptive use.
- Focused Physical Examination which involves an examination of the body system(s) related to participant's abnormal presentation to assess unsolicited AEs.
- Reminder of insecticide-treated bed nets use.
- Follow-up of unresolved AEs.
- Capturing of unsolicited AEs.
- Blood sampling for haematology and biochemistry (ALT, total bilirubin and creatinine) laboratory evaluations.

7.1.9 28 day site visit post 1st, 2nd and 3rd vaccination (D28, D56 and D84)

The following assessments will be done **before the participant is vaccinated** on D28 and D56, as well as on D84 at 28 day site visit follow-up after the 3rd vaccination:

- Confirmation of willingness to participate.
- Vital signs (axillary temperature, BP, HR and RR (BP and HR after 10 min rest in a supine position)).
- Body weight measurement.
- Review of concomitant medication.
- Review of contraceptive use.

- Focused physical examination which involves an examination of the body system(s) related to participant's abnormal presentation.
- Reminder of insecticide-treated bed nets use.
- Capturing of unsolicited AEs.
- Follow-up of unresolved AEs.
- Pregnancy test: all women of childbearing potential will undergo a serum pregnancy test.
- Urinalysis: Urine dipstick for protein, glucose and blood.
- Blood sampling for haematology and biochemistry (ALT, total bilirubin and creatinine) laboratory evaluations.
- Blood sampling for Malaria (qPCR and TBS).
- Blood sampling for humoral & cellular response.
- Blood sampling for exploratory samples.
- Urine and stool collection for retrospective exploratory analysis.
- Review of eligibility for 2nd/3rd vaccination (on D28 or D56, respectively).

7.1.10 Vaccination site follow-up (D112, D140) visits

Following procedures are to be done on each follow-up visit:

- Vital signs (axillary temperature, BP, HR and RR (BP and HR after 10 min rest in a supine position)).
- Body weight measurement.
- Review of concomitant medication.
- Review of contraceptive use
- Focused physical examination which involves an examination of the body system(s) related to participant's abnormal presentation.
- Reminder of insecticide-treated bed nets use.
- Assessment of unsolicited AEs.
- Follow-up of unresolved AEs.
- Pregnancy test: all women of childbearing potential will undergo a serum pregnancy test.
- Blood sampling for biochemistry (ALT, total bilirubin and creatinine) laboratory evaluations.
- Blood sampling for humoral response.
- Blood sampling for cellular immune response.
- Blood sampling for exploratory samples (only D112).
- Blood sampling Malaria (qPCR and TBS)

7.1.11 Unscheduled visit

Unscheduled visits may occur at any time during the study. The procedures below are to be performed at any unscheduled visit.

- Vital signs (axillary temperature, BP, HR and RR (BP and HR in a supine position)), if clinically indicated.
- Body weight measurement.
- Review of concomitant medication.
- Focused physical examination which involves an examination of the body system(s) related to participant's abnormal presentation.
- Assessment of AEs.
- Follow-up of unresolved AEs.
- Clinical safety laboratory evaluations (e.g., haematology, biochemistry, urinalysis), if clinically indicated or required based on the Investigator's judgement.
- Any other procedure that may be relevant based on the participant's clinical presentation (e.g. ECG)

Any unscheduled follow-up will be documented on the unscheduled visit forms.

7.2 Total blood volume

The blood volumes planned to be collected from each participant during the course of this study are detailed in section 4.3.1. Additional samples may be required in the event of AEs/SAEs, additional laboratory safety tests or in case a laboratory test needs to be repeated as judged by the Investigator.

8 Assessment of safety

8.1 Safety assessments

General questioning about solicited and unsolicited AEs will be done at every visit after administration of the vaccination.

8.1.1 Physical Examination

A complete physical examination will be conducted at screening and will assess general appearance including height (at screening 1 only) and weight and the following areas/ symptoms: skin, lymph nodes, head and neck, eyes, ears, nose, throat, respiratory, cardiovascular, abdomen, extremities, musculoskeletal neurological and urogenital.

A targeted symptom-directed physical examination will be assessed at designated study visits based on interim medical history and the participant’s complaints/observations. At any time, a symptom-directed physical examination can be performed as deemed necessary by the Investigator.

Abnormalities assessed as clinically significant will be reported as an AE.

8.1.2 Vital Signs

Vital signs (BP, HR, axillary temperature and RR) will be collected in the source documents at each study visit for medical purposes.

HR and BP will be taken after 10 minutes rest in a supine position.

During the trial, vital signs will be repeated if they fall outside the normal ranges [39] [40].

If the result of the repeat measurement is still out of range, the Investigator will make an assessment of clinical significance. If the abnormality is assessed during the screening visit, it is captured as medical history. After the participant has received his/her first vaccination, clinically significant abnormalities will be reported as an AE. The investigator will decide on an appropriate course of action.

8.1.3 12-lead ECG safety recording

One ECG is performed at screening visit 1. It can also be performed at any time if there are signs and symptoms suggesting cardiac problems.

Instructions for recording and handling of the ECG will be included in a separate Manual. Repeats will be done in case of an erroneous reading.

The following complaints are considered potentially related to cardiac complications in the adult age group and may lead to cardiac evaluation, including the consultation of a cardiologist (independent physician) if deemed necessary by the investigator:

- Chest pain (other than chest wall pain or respiration-related pain)
- Palpitations
- Shortness of breath

These symptoms can occur in individuals with malaria, especially after initiation of anti-malarial treatment, but a cardiac aetiology is also to be considered. In addition, investigators may also seek cardiac consultation based on other symptoms judged to be cardiac related.

8.1.4 Laboratory assessments

The following laboratory assessments will be conducted in accordance with the local laboratory's standard operating procedures.

One or more of the laboratory parameters may be repeated at any time during the study as determined by the PI or if indicated by an SAE/AE.

Laboratory abnormalities after the first vaccination assessed as "clinically significant" have to be reported as an AE (section 8.2) only if they meet at least one of the following conditions:

- The abnormality suggests a disease and/or organ toxicity AND this abnormality was not present at the screening visit, or is assessed as having evolved since the screening visit.
- The abnormality results in discontinuation of the study IMP.
- The abnormality requires medical intervention or concomitant therapy.

When reporting an abnormal laboratory result, a clinical diagnosis should be recorded rather than the abnormal value itself, if available (for example, "anaemia" rather than "decreased red blood cell count").

Haematology

Haemoglobin, WBC (differentiation of eosinophils and neutrophils), platelets and haematocrit.

Biochemistry

At screening: Biochemistry parameters at screening will include: ALT, AST, total bilirubin, creatinine and glucose (random). Troponin sample (baseline) will be collected and stored to be run retrospectively if needed in case of a cardiac event. The sample collected at the time of the cardiac event will be compared with the sample collected at baseline.

Subsequent visits: During subsequent scheduled visits ALT, total bilirubin and creatinine evaluations will be performed.

Urinalysis performed by dipstick

Proteinuria, glucose and blood. (**Note:** Urinalysis should be deferred if a participant is menstruating, but should be performed as soon as possible).

8.2 Collection and reporting of adverse events

8.2.1 Definition of adverse events (AEs)

An adverse event (AE) is defined as any untoward medical occurrence (including a clinically significant abnormal laboratory finding, for example) in a clinical research participant administered with a pharmaceutical product and which does not necessarily have to have a causal relationship with this treatment. Definition of an AE includes worsening (in severity or frequency) of a pre-existing condition ("medical history") before first IMP administration and abnormalities of procedures (i.e. ECG, physical, or neurological examination, etc.) or laboratory results which are assessed as "clinically significant".

8.2.2 Types and recording of AEs

The term "AE" could include any of the following events which develop or increase in severity during the course of the study:

a. Any signs or symptoms whether thought to be related or unrelated to the vaccination under study
b. Any clinically significant laboratory abnormality
c. Any abnormality detected during a physical examination

These data will be recorded in the CRFs, regardless of whether they are thought to be associated with the study or the drug under investigation.

8.2.3 Grading of adverse events severity

For each AE, the Investigator is required to assess the maximum severity. The distinction between severity and seriousness of AEs is to be noted. A severe AE is not necessarily an SAE.

All AEs/SAEs (except laboratory abnormalities and vital signs) will be graded by the Investigator using the Common Terminology Criteria for Adverse Events (CTCAE) terminology: mild, moderate, severe, life-threatening or death according to the following definitions:

- Mild (Grade 1): These events do not interfere with the participant's daily activities.
- Moderate (Grade 2): These events cause some interference with the participant's daily activities and require limited or no medical intervention.
- Severe (Grade 3): These events prevent the participant's daily activities and require intensive therapeutic intervention.
- Life-Threatening (Grade 4): The subject is at significant risk of life; it does not refer to an event which hypothetically might have caused death if it were more severe (life-threatening consequences, urgent intervention required).
- Death (Grade 5) Death related to an event.

Laboratory abnormalities and vital signs listed in the current version of the site's grading scheme (Manual of Reference Intervals and Grading of Selected Abnormal Values) adapted from FDA Guidance for Industry Toxicity Grading Scale for Healthy Adult and Adolescent Volunteers Enrolled in Preventive Vaccine Clinical Trials document [41], will be graded according to this document. The Investigator's medical and scientific judgement will decide whether an abnormal laboratory finding or other abnormal assessment is clinically significant or not.

8.2.4 AE causality assessment

For all AEs, the Investigator is required to assess if there is a causal relationship between the AE and the IMP, i.e. to determine whether there exists a reasonable possibility that the IMP caused or contributed to the AE(s).

The following categories for relationship to treatment will be used during AE reporting:

- Definitely related: The AE and administration of IMP are related in time, and a direct association can be demonstrated
- Probably related: The AE and administration of IMP are reasonably related in time, and the AE is more likely explained by the IMP than other causes
- Possibly related: The AE and administration of IMP are reasonably related in time, and the AE can be explained equally well by causes other than the IMP
- Unlikely related: A potential relationship between IMP and the AE could exist (i.e. the possibility cannot be excluded), but the AE is most likely explained by causes other than the IMP
- Not related: The AE is clearly explained by another cause not related to the IMP

Note: for regulatory reporting purposes, when compared to binary classification, "not related" corresponds to "not related, unlikely related" and "related" corresponds to "possible, probable and definitely related".

In addition, for SAEs, the Sponsor will also perform a causality assessment.

8.2.5 Serious adverse events (SAEs)

A "serious" adverse event (SAE) is defined as any event that suggests a significant hazard, contraindication, side effect or precaution. A serious adverse event includes any event that:

1. is **fatal**.

2. is **life threatening**, meaning, the participant was, in the view of the Investigator, at immediate risk of death from the reaction as it occurred, i.e., it does not include a reaction that, had it occurred in a more serious form, might have caused death.
3. is a **persistent or significant disability or incapacity**, i.e., the event causes a substantial disruption of a person's ability to conduct normal life functions.
4. requires, or prolongs **in-patient hospitalization**; i.e. the AE requires at least an overnight admission or prolongs a hospitalization beyond the expected length of stay according to the protocol. Hospital admissions for surgery planned before study entry, for social reasons, for any elective surgery (i.e. plastic surgery) are NOT to be considered as SAE according to this criterion.
5. is a **congenital anomaly or birth defect**.
6. is an **important medical event**, based upon appropriate medical judgement, that may jeopardize the participant or may require medical or surgical intervention to prevent one of the other outcomes defined as serious.

8.2.6 Expectedness of AEs

The following are local solicited AEs occurring within 7 days after vaccination:

- Warmth
- Erythema
- Itching
- Edema
- Pain
- Infection
- Lipodystrophy
- Ulceration
- Induration
- Necrosis
- Other tissue damage

Note: If needed, photograph(s) of injection site will be taken for clinical evaluation

The following are general solicited AEs occurring within 7 days after vaccination:

- Fatigue
- Chills
- Sweating
- Myalgia
- Arthralgia
- Gastrointestinal symptoms (e.g. nausea, vomiting)
- Headache
- Fever >38°C
- Rash

The Sponsor is responsible for further determining the expectedness of the event, using the Investigator Brochure of the IMP and rabies control vaccine.

8.2.7 Reporting Obligations

The AE reporting period begins with the administration of the 1st vaccination and ends with the last study visit.

Each AE is to be classified by the Investigator as serious or non-serious. This classification will determine the reporting procedure for the event.

All SAEs are to be reported **immediately** (within 24 hours of awareness of the SAE by the Investigator) **to the Sponsor in writing**. All written reports should be transmitted to the Sponsor by e-mail using the SAE report form. This includes a description of the event, onset date and type, duration, severity, relationship to the IMP, outcome, measures taken and all other relevant clinical and laboratory data. In exceptional circumstances, an SAE (or follow-up information) may be reported by telephone, followed by a completed SAE report form. The initial report is to be followed by submission of additional information (follow-up SAE form) as it becomes available.

SAEs should also be reported on the AE page of the eCRF. It should be noted that the form for reporting of SAE (SAE form) is not the same as the AE section of the eCRF. Where the same data are collected, the two forms must be completed in a consistent manner, and the same medical terminology should be used.

Expedited reporting to Regulatory Authorities (TMDA), Independent Ethics Comittee(s) - National Health Research Ethics Review Committee (NatHREC) and Institutional Review Board(s) (IHI-IRB) will be done by the Investigator on behalf of the Sponsor in following their reporting requirements. Expedited reporting will be done in a blinded manner unless an SAE is thought to be related to the IMP or unblinding is necessary to inform the management of the participant condition.

A suspected unexpected serious adverse reaction (SUSAR) is a suspected AE related to an IMP that is both unexpected and serious.

8.3 Follow-up of adverse events

All AEs should be followed until:

- they are resolved; or
- the Investigator assesses them as “chronic” or “stable”; or
- the participant’s participation in the trial ends (i.e., until their follow-up visit is complete, or otherwise until the last contact with the subject).

In addition, all SAEs (related or not) and those non-serious events assessed by the Investigator as having a reasonable possibility of relationship to the IMP must continue to be followed even after the subject participation in the trial is over. Such events should be followed until they resolve or until the Investigator assesses them as “chronic” or “stable.” The outcome of these events is to be documented in the eCRF (up to the end of study participation) and SAE form (up to and after the end of study participation).

All SAEs will be summarized and reported to IHI-IRB and NatHREC in the required timeframes. The PI, on behalf of the Sponsor, will also report all SAEs to the TMDA within the specified timeframe as required by the regulations.

8.4 Data collection and follow-up of prematurely terminated participants

See section 5.5.1.

9 Statistics

9.1 Hypothesis

No formal statistical hypothesis testing is planned for this study due to its descriptive and exploratory nature.

9.2 Determination of sample size

No formal sample size calculation has been performed. The sample size of 40 participants (20 per arm) is within the range of those typically used in early phase trials. It is expected to allow assessment of the safety, reactogenicity and immunogenicity of SumayaVac-1 (SUM-101) in malaria exposed adults of African origin in this setting.

9.3 Description of statistical methods

Analysis and reporting will follow ICH E9 [42, 43]. All analyses that will be reported in SMC meetings and in clinical study report will be performed by the trial statistician, or a suitably qualified delegate with oversight by the trial statistician.

Analyses will be performed for reporting to the SMC, according to the schedules outlined in section 4.5. These analyses will include safety data and will be performed in a blinded manner, with both arms (namely, SumayaVac-1 (SUM-101) or Verorab® control) combined.

The final analysis for the study is planned after all participants have completed follow-up. For the final analysis, the trial statistician will be unblinded, and results will be presented separately by arm.

The numbers of individuals screened and reasons for screening failure will be summarized. The numbers of participants enrolled along with their demographic and other baseline characteristics will be summarized. The completion of follow-up will be summarized, along with details of discontinuations or withdrawals.

Continuous variables will be inspected using histograms: 1) to assess for outliers which may be queried for accuracy, and 2) to assess whether appropriate transformations are required for analyses. In general, continuous variables will be summarized using descriptive statistics such as number, mean, standard deviation, median, maximum and minimum, after transformation if necessary. Categorical variables will be reported as frequencies and percentages. Completeness of data will be summarized. Analyses will be descriptive with no formal comparisons between arms. Measures of precision (such as 95% confidence intervals) for endpoints may be provided where appropriate in order to inform subsequent clinical evaluation of the IMP. Graphical depictions will be used where appropriate to illustrate the data.

9.3.1 Datasets to be analysed, analysis populations

Participant inclusion into each analysis population will be determined prior to the final analysis. The analysis populations will be as follows:

- Intent-to-Treat (ITT) population: all randomised participants.
- Per Protocol (PP) population: subset of the ITT population, who completed the study without any major protocol violation.
- Safety population: all participants having received at least one vaccination.

Participant disposition and demographic data analyses will be based on the ITT population, according to the randomisation arm. All other analyses will be based on the safety population by the vaccinations received, regardless of the randomisation (any such deviations will be reported). For the immunogenicity endpoints, analyses will also be performed using the PP population.

Participants who drop out of the study before receiving any vaccination will be replaced. In this case, the replacement participant would receive the same allocation as planned for the participant who dropped out, in order to maintain the correct overall numbers of participants allocated to each arm. This is justified by the replaced participant not having received any vaccination, and concealment of allocation being maintained by the blinded nature of the study. Participants who drop out of the study after receiving at least one vaccination will not be replaced.

9.3.1.1 Primary Analysis

In general, AEs and SAEs will be captured using the Medical Dictionary for Regulatory Activities (MedDRA) preferred terms grouped by primary system organ class. Results will include the total occurrences of each type of endpoint, and the number of participants experiencing at least one of each type of endpoint.

For the primary endpoints of (i) local and systemic solicited AEs at least possibly related to IMP recorded from after each vaccination (done on D0, D28 and D56) up to 7 days later and (ii) local and systemic unsolicited reactogenicity recorded from after each vaccination (done on D0, D28 and

D56) up to 28 days later, results will be reported overall, and separately by vaccination (first, second, third).

Individual listings of AEs and SAEs (including relevant participant information such as age and sex; and AE details such as onset date, duration, severity, seriousness, expectedness, relation to study drug; see section 8.2) will be provided.

Laboratory safety parameters will be summarized as absolute values. Changes in laboratory safety parameters will be assessed between before each vaccination (done on D0, D28 and D56) and at 28 days later, separately for each vaccination (first, second, third). Changes will also be assessed at 28 days after each vaccination, compared to baseline values (before 1st vaccination).

To evaluate the immunogenicity primary endpoints (see section 4.1.1), antibody responses will be summarized over time, and fold changes of antibody responses will be summarized over time relative to baseline.

9.3.1.2 Secondary Analyses

The secondary endpoints (see section 4.1.2) will be summarized over time to allow descriptive assessment in trends over time. Continuous and categorical endpoints will be analysed as described in section 9.3.

The following analyses will also be performed:

- Clinical and laboratory safety data (such as haematology, biochemistry, urinalysis, vital signs, physical examination) will be described over time. Changes versus baseline will also be presented, along with AE gradings as appropriate.
- Concomitant therapy will be summarized.

9.3.1.3 Interim analyses

Interim analyses are planned for the SMC. The SMC will perform reviews according to the schedules outlined in section 4.5. Stopping rules and possible outcomes of the SMC meetings are detailed in section 4.5. An interim analysis of only the primary outcomes, fold change of antibody responses and longevity of antibody responses of group 1 samples only will be performed in a blinded manner by the laboratory at the University of Heidelberg. As hypothesis testing will not be performed, no adjustments to the statistical analysis for multiple testing are required.

9.3.1.4 Safety analysis

Safety parameters are primary endpoints of the trial; see analyses described in section 9.3.1.1.

9.3.1.5 Deviation(s) from the original statistical plan

A full statistical analysis plan will be finalised before any substantial analysis of the data. Deviations from the original statistical analysis plan will be avoided if possible, but if necessary, would be reported in the final report along with justifications for those deviations.

9.4 Handling of data

Data will be summarized and inspected for potential errors. Missing data will be summarized, and percentages will be reported of those with non-missing data. Drop outs (and reasons, if captured) will be reported. There are no imputations planned for missing data or replacement of participants who drop out, except as detailed in section 4.2.1.

10 Ethical considerations

The study will be carried out in accordance to the protocol and with principles enunciated in the current version of the Declaration of Helsinki, the guidelines of GCP issued by ICH, the local law and RA's requirements. In agreement with local requirements, the IEC and RA will receive regular safety reports and will be informed about study stop/end.

10.1 Independent Ethics Committee (IEC)

Prior to enrolment of any participant into this trial, the study protocol and the informed consent form (ICF) will be reviewed and approved by the IHI-IRB and NatHREC in Tanzania.

In parallel, the trial will be submitted to the IEC in Switzerland (Ethikkommission Nordwest- und Zentralschweiz (EKNZ)) to get an ethical statement of the trial.

Premature study end or interruption of the study will be reported within 15 days by the Sponsor or by the PI to the IRB (IHI-IRB) and IECs (NatHREC and EKNZ). The regular end of the study and the final clinical study report will be submitted in accordance with the applicable timelines after the end of the study.

10.2 Regulatory Authority (RA)

Prior to the start of the clinical trial, approval from the Tanzanian Regulatory Authority- TMDA, will be obtained.

Premature study end or interruption of the study will be reported within 15 days to the competent authority. The regular end of the study and the final clinical study report will be submitted in accordance with the applicable timelines after the end of the study by the PI.

10.3 Evaluation of the risk-benefit ratio

Potential risks with the IMP are detailed in section 2.7.

The participants are unlikely to benefit from the study IMP, but they may profit from a comprehensive health check and protection from rabies upon receiving Verorab rabies vaccine. If a chronic or underlying disease is identified, participants will be referred accordingly within the national health system. As for any Phase I clinical trial, it is not possible to claim any protection from the disease the vaccine is targeting.

In addition, there is the potential that the gained knowledge could help in the development of new vaccines against malaria. This could lead to societal benefits if a new vaccine is identified. The general risks to participants in this study are associated with phlebotomy and with vaccination. The blood volume drawn over the course of the study should not compromise the otherwise healthy subjects.

10.4 Participant information and consent

Written informed consent of a participant, using the IEC approved consent form, must be obtained before any study procedure is performed. Upon showing interest, IEC-approved language and an appropriate and study-specific Participant Information Sheet(s) (PIS) will be made available to the potential participant and the opportunity to discuss the study will be given.

The participants will be fully informed orally by the Investigator or designee about all aspects of the trial, the potential risks and their obligations. The following general principles will be emphasized:
The participation in the study is entirely voluntary.

- The refusal to participate involves no penalty or loss of medical benefits.
- The participant may withdraw from the study at any time.
- The participant is free to ask questions at any time to allow him/her to understand the purpose of the study and the procedures involved.
- The study involves research of an investigational vaccine.
- There is no direct benefit from participating.
- The participant's general practitioner may be contacted to corroborate their medical history or seek additional information.
- The participant's blood samples taken as part of the study will be stored at the site and samples will be sent outside Tanzania to collaborating laboratories (see section 15.1). These samples will be identified only by code numbers.

- The aims of the study and tests to be carried out will be explained.
- Reimbursement will be provided for participation as described in section 10.9.
- All relevant clinical and laboratory results (serology, lab tests and interpretations of physical exams) will be shared directly with the concerned participants.
- Relevant aspects regarding data protection and confidentiality.

The participant will be given enough time to make an informed decision about his/her participation in the study. The participant will be asked to read, and consider the statement before signing and dating the ICF. The consent form must be signed and dated by the Investigator (or their designee) at the same time as the participant. A copy of the fully signed form and PIS will be given to the participant and the original will be retained as part of the study records.

10.5 Registration of clinical trial

The study was registered with a recognized clinical study registry, e.g., www.clinicaltrials.gov, before commencing any study procedures.

10.6 Participant confidentiality

The Investigator must assure that participants' confidentiality will be maintained and that their identities are protected from unauthorized parties.

Participants' identities will not be disclosed to the Sponsor. The Investigator will maintain a Participant Identification List so that the participants can be identified. The list will stay under secure control of the site with strict access control processes in place.

Individual participant medical information obtained as a result of this study is considered confidential and non-anonymized disclosure to third parties is prohibited. Participant confidentiality will be ensured by utilizing participant identification codes. No patient identifiable data will be collected in the electronic data capture (EDC) tool except for date of birth, as this information is critical for the inclusion of participants and for the statistical analysis of the data. However, data minimization is applied and only month and year of birth are collected for participants.

As part of the informed consent process, the participants will be informed in writing that representatives of the Sponsor (like monitors, auditors, etc.), a RA or Ethics Committee may require direct access to parts of the medical records relevant to the study, including participant's medical history, and that all personal data will be handled strictly confidentially and in accordance with local data protection laws.

Blood samples will be collected at BCTF, Tanzania and samples used for testing of some secondary and exploratory endpoints will be shipped to Germany, Switzerland, Italy and USA.

Study results will be published without providing individual information on the participants that can identify them.

10.7 Participants requiring particular protection

Only healthy and literate adults aged 18-45 years will be asked to participate in this Phase Ib clinical trial.

10.8 Insurance

A clinical trial insurance will be provided by the Sponsor. A copy of the certificate will be filed in the Investigator Site File and the Trial Master File.

The Sponsor is insured to indemnify the Investigator against any claim for damages brought by a participant who suffers from a research- related injury during the performance of the trial according to the protocol, except for claims that arise from malpractice and/or negligence. This is covered by the clinical trial's host institute (Ifakara Health Institute).

In accordance with local regulations, the Sponsor will contract insurance for all study participants.

The Bagamoyo Clinical Trial Facility (BCTF) will be primarily responsible for providing participant's medical care whether it is related or not related to the study for the duration of the trial. If specialist, emergency care or hospitalization is needed, this will be provided at no cost to the participant at the Bagamoyo District Hospital or referral centers in Dar-es-Salaam, Tanzania.

For all unrelated conditions to the study procedures, the participant will be responsible for seeking and funding this care through the normal channels provided by the Ministry of Health and Social Welfare of Tanzania. The obligation to provide medical care for conditions arising during the trial that are not related to the IMP or study procedure will end with the last study visit of the participant.

10.9 Participant reimbursement

Participants will not be paid for their participation, but all other expenses related to trial participation will be covered by the study. This will include meals, beverages and reimbursement for the transport fare whenever they attend to BCTF for scheduled or unscheduled visits. As compensation for their time, the participants will be given 15,000 TSH (ca. 6.40 USD) at each visit at the site. An additional 5,000 TSH (ca. 2.13 USD) will be given for transport when participants leave the site. Hence, compensation inclusive of meals, beverages, transport costs and time sum up to about 30,000 TSH (ca 12.86 USD) per site visit.

10.10 Protocol amendments

A protocol amendment can be initiated by either the Sponsor or the Investigator. The Investigator will provide the reasons for the proposed amendment in writing and will discuss with the Sponsor. Any protocol amendment must be approved and signed by the Sponsor and the PI and must be submitted to the appropriate IEC for information and approval, in accordance with local requirements, and to RA if required. Approval by IEC (and RA, if applicable) must be received before any changes can be implemented, except for changes necessary to eliminate an immediate hazard to study participants, or when the change involves only logistical or administrative aspects of the study, e.g., change of telephone number(s).

11 QUALITY CONTROL AND QUALITY ASSURANCE: DESCRIPTION OF MEASURES

The PI will ensure that all study personnel are appropriately qualified and trained on all important study related aspects, including a protocol-specific training, GCP training, training on data entry and handling, study interventions and assessments. All applicable documentation is maintained on site.

11.1 Data handling and record keeping / archiving

11.1.1 Case Report Forms

Data recorded during the trial will be captured online in electronic Case Report Forms (eCRF). Access to the eCRF is personalized and secured by user specific passwords. For each participant a record is created that will contain all data as required by the protocol. On the eCRF and other trial specific documents, participants will be identified via a unique participant identifier. The participant identification list will stay under secure control of the site with strict access control processes in place.

Data minimization is applied and only month and year of birth will be collected for participants in the eCRF as this information is critical for the inclusion of participants and for the statistical analysis of the data.

To minimize the data in the eCRF after the baseline assessment (at screening visit or D0 pre-vac visit), only clinically significant abnormal observations for physical examinations will be captured as AEs in the eCRF. Other documentation remain as references in the source document only.

The Investigator is responsible for ensuring that data entries are complete, accurate, legible, and timely and confirms this by physically or electronically signing off the eCRF.

11.1.2 Specification of source documents

Source documents may include participant hospital/ clinic records, physician's and nurse's notes, appointment book, original laboratory reports, ECGs, pathology and special assessment reports, signed ICFs, consultant letters, AE logs, and logs of participants screened/randomised. Source data can be electronic or paper-based.

Source data should be attributable, legible, contemporaneous, original, accurate, and complete.

For quality assurance, direct access to the source data and related trial documentation needs to be granted for monitoring, audit and inspection purposes. All involved parties will keep the participant data strictly confidential.

11.1.3 Record keeping/ archiving

The PI must maintain adequate and accurate records to enable the conduct of the study to be fully documented and the study data to be subsequently verified. These records include the Investigator Site File, participant clinical source documents and the eCRF. The Investigator Site File will contain the logs of participants screened and randomised, protocol/protocol amendments, SAE Reporting Forms and query forms, approval from Ethics Committee(s) and RAs with correspondence, sample of the approved study information documents and ICF, completed ICFs, drug accountability records, training and authorization forms, hard copies of eCRFs and queries (if applicable), records of maintenance and/or calibration of equipment, and other appropriate documents/correspondence etc. The PI is responsible for storing the Investigator's Site File and other study documentation in a secure location and to archive them at the end of the clinical study.

The Trial Master File will be kept and maintained by the Sponsor.

The electronic data will be kept for 25 years at Swiss TPH after study completion or premature termination of the clinical trial. At the end of the study, a copy of the data from the eCRF will be provided to the Investigator in an appropriate format, e.g., DVD or flash drive, for long-term archiving. The code list and medical data will be kept in a safe place at Ifakara Health Institute for a period of 25 years after the end of the study.

11.2 Data management

The Sponsor is responsible for data management and will have procedures in place for all relevant data management processes, including but not limited to eCRF development, eCRF validation, data cleaning, user access management and archiving.

11.2.1 Data management system

All relevant participants' data collected during the study period will be recorded in electronic Case Report forms (eCRFs) by a validated EDC tool. The EDC tool maintains a time stamped audit trail that records all user-specific data processing operations such as initial data entry, modification and reasons for change.

11.2.2 Data security, access and back-up

The data in the EDC tool will be stored on a secure server in Switzerland, with a defined policy in place for server set-up, maintenance and security. A backup of the data will be performed automatically on a regular basis. All data captured in the EDC tool, will be encrypted and password secured. Only study team members (as listed in the delegation log/study team log), as well as Sponsor study team, monitors, auditors, ethics committee representatives or RAs will be given access to the study data. Participant data will be handled with utmost discretion and will only be accessible to trained and authorized personnel who require access to the data to fulfil their duties within the scope of the study.

11.2.3 Analysis and archiving

After the last visit of the last participant, final data queries will be resolved, the trial database is locked and the data are extracted from the system for analysis. The data are stored electronically in access restricted folders implemented by the Sponsor.

See section 11.1.3 for more details about data storage and archiving.

11.2.4 Electronic and central data validation

The data will be centrally monitored at Swiss TPH by Data Managers and Monitors to ensure completeness and quality of the data and consistency with the study protocol. Off-site and on-site monitoring activities will be detailed in the data cleaning and reporting plan and monitoring plan, respectively.

11.3 Risk management

11.3.1 Risk identification, assessment and mitigation

A risk management plan will be set up by the Sponsor. Potential risks to the study, their assessment and management will be documented by the Sponsor in a Risk Management Log. The log will be updated continuously throughout the study duration.

Special attention will be paid to risks associated with participant safety, critical study procedures and critical data.

11.3.2 Study-specific preventive measures

Sentinel dosing

To minimize the risk to healthy participants, the administration of the vaccines will be done sequentially with three participants in the sentinel group as outlined in section 4.

Follow-up post vaccination

The participant is discharged 2 hours post vaccination, which should allow enough time to assess and take care of any discomfort for the participant appearing directly after vaccination. Then the participant is discharged and followed-up daily by phone or home visit (if deemed necessary by the study team) until the next visit at site. With this schedule the participant's time at site can be minimised while at the same time discomfort for the participant can be detected and managed early.

Birth control and pregnancy testing

Women of child-bearing potential (WOCBP):

WOCBP will only be enrolled after a negative highly sensitive serum pregnancy test. Serum pregnancy tests will be conducted at screening visit 1, during the study before 1st (D0), 2nd (D28) and 3rd (D56) vaccination and at follow up visits after vaccination (D84, D112 and D140).

WOCBP must agree to actively use a highly effective form of contraception from 4 weeks before 1st vaccination up to 12 weeks after 3rd vaccination. One of the following methods of contraception for WOCBP would be acceptable:

- Injectable progesterone containing hormonal contraception
- Implantable progesterone containing contraception
- Intrauterine device (IUD)
- Intrauterine hormonal releasing system (IUS)
- Bilateral tubal occlusion

Male participants must be willing to ensure the use of condoms from before 1st vaccination up to 12 weeks after 3rd vaccination. An adequate number of condoms will be distributed to participants free of charge.

Risks associated with epidemics

The presence of an epidemic/pandemic (including for example COVID-19) can have an impact on the trial, trial participants and staff. Healthy participants may be exposed to infection during screening activities in the communities, or whilst at the investigational site, and there is the risk of trial staff transmitting infection to communities from which participants will be recruited. Special attention is paid to the risk for the participants to be exposed to pathogens while staying at the site. Current Standard Precautions measures to prevent infection will be put in place at the site level.

Mitigation steps to be taken include:

- Prior to initiation of recruitment, and during the trial, an assessment will be made by the Investigator and the Sponsor of the current potential risk associated with each aspect of the trial, based on the incidence of infections in the area of recruitment, the site and national directives. The start of recruitment can be delayed (or paused, if already started) if indicated, or mitigation measures taken to protect staff and participants.
- National and international specific guidelines will be followed during the trial to prevent spread of infection, and protective equipment will be provided. Training will be provided to site staff on standard precautions/infection based precautions.
- Specific risk communication and education activities on national preventive directives on COVID-19 will be implemented for participants as well as site staff.
- The investigational site follows its institutional IHI COVID-19 response plan that provides instructions including guidelines on clinical trials, operations at clinics and hospitals, community and field activities, facemasks and operations inside labs.
- In case travel to and from the site is blocked, the site staff will ensure basic follow-up of participants including supporting access to local medical assistance if required while keeping remote contact with the investigational sites.

Only healthy participants will be included in the trial.

11.4 Safety monitoring committee (SMC)

An SMC will be established to ensure the safety of study participants is protected, whilst the scientific goals of the study are being met, according to the protocol and in accordance with international and local standards.

The SMC is an additional measure to complement the Sponsor's and Investigator's usual procedures for safety monitoring during clinical trials, aiming an in-depth evaluation on the safety criteria, in accordance with the protocol and with international and local standards.

The SMC will meet for evaluation of the Go/ No-Go criteria as described in section 4.5 and if deemed necessary. The decision to proceed to the next step will be made based on blinded safety and tolerability data. As defined in protocol section 4.5, the study will be halted, and the risk to other participants evaluated if any of the stopping criterion is met.

The composition, roles and responsibilities of the SMC will be described in detail in the SMC charter. The SMC will be comprised of a minimum of three persons, not related directly to the current clinical trial and also include at least one Tanzanian member.

11.5 Translations - Reference language

The reference language for study documents is English.

Documents used in advertisement and information about the study like the information sheet, ICF etc. used for persons outside the study team will be translated to Swahili.

For translated documents there will be a translation form completed by the person having done the translation and a second person verifying the translation.

For the information sheet, ICF(s) and additional documents deemed necessary by the Sponsor or PI, the reference master document will be in English. In those cases, in addition a third person will

be performing and documenting a focused review where the layout and structure of the document will be verified.

11.6 Storage of biological material and related health data

Safety blood samples will be destroyed as soon as the analysis of the study data has been completed.

A subset of samples taken for measuring humoral and cellular responses will be analysed in Basel, Switzerland and Heidelberg, Germany and then stored there. In addition, some samples might be shipped and analysed at the University of Maryland, United States of America. Samples collected for in depth analysis of B-cell responses after vaccination will be analysed in Milan, Italy.

Exploratory samples and source data will be stored and archived at the BCTF. The sampling that can be used for further malaria research or health related research as well as the samples sent to Switzerland, USA, Italy and Germany will be mentioned in the information sheet or ICF(s) and explained to the participants.

All samples stored will be labelled with the participants ID and identifiers that do not allow to identify the study participant directly.

The data obtained in this study may provide the basis for further studies using samples stored for exploratory purposes. For those future evaluations the necessary approvals are required before sharing of the samples is possible.

12 FUNDING

This project is funded by Sumaya Biotech GmbH & Co. KG, Heidelberg, through a Venture Loan Agreement with EMF EU Malaria Fund Berlin GmbH & Co. KG.

The Sponsor, the PI and supporting organizations/institutions declare to have no conflict of interest.

The Investigators, including the PI and/or any Sub-Investigators, directly involved in the treatment or evaluation of participants may be requested to provide a financial disclosure. All relevant documentation will be filed in the Trial Master File.

13 DISSEMINATION OF RESULTS AND PUBLICATION POLICY

All data and results generated in the study will be owned by the industrial Sponsor, Sumaya Biotech GmbH & Co. KG, who may utilize them in various ways, such as for submission to the RAs.

Swiss TPH as sponsor will make the data derived from the study freely available publicly through appropriate open access databases, repositories and similar tools.

The Investigators will be involved in writing and/or reviewing drafts of the manuscripts, abstracts, press releases and any other publications arising from the study. Apart from obvious flaws to the conduct of the study, which may preclude data publication, safety and efficacy data will be published under the supervision and authorization of the Sponsor in appropriate peer-reviewed journal(s).

Authorship for publication in scientific journals will be offered to each party that has substantially contributed to the work reported in the publication. It is expected that authorship of such publications will be led by the Sponsor in close collaboration with Sumaya Biotech. The study team must seek permission from NatHREC before publishing any manuscript as per local regulations.

The interim and final clinical study report will be the responsibility of the Sponsor and will be submitted to the RAs and ethics committee(s), if required by these bodies (see sections 10.1 and 10.2) when available.

Data from the study may also be used as part of a thesis for a PhD, MD or Masters. Publications arising from this study will be made open access.

The Sponsor encourages the communication and/ or publication of the results, in accordance with the terms of the Master Service Agreement with the Industrial Sponsor and the site Clinical Trial Agreement.

14 References

1. Ashley, E.A., A. Pyae Phyo, and C.J. Woodrow, *Malaria.* Lancet, 2018. **391**(10130): p. 1608-1621.
2. *World malaria report 2021.* Geneva: World Health Organization; 2021., 2021. **Licence: CC BY-NC-SA 3.0 IGO**.
3. Phillips, M.A., et al., *Malaria.* Nat Rev Dis Primers, 2017. **3**: p. 17050.
4. Cowman, A.F., et al., *The Molecular Basis of Erythrocyte Invasion by Malaria Parasites.* Cell Host Microbe, 2017. **22**(2): p. 232-245.
5. Raj, D.K., et al., *Antibodies to PfSEA-1 block parasite egress from RBCs and protect against malaria infection.* Science, 2014. **344**(6186): p. 871-7.
6. Singh, S. and C.E. Chitnis, *Molecular Signaling Involved in Entry and Exit of Malaria Parasites from Host Erythrocytes.* Cold Spring Harb Perspect Med, 2017. **7**(10).
7. Gilson, P.R., et al., *Identification and stoichiometry of glycosylphosphatidylinositol-anchored membrane proteins of the human malaria parasite Plasmodium falciparum.* Mol Cell Proteomics, 2006. **5**(7): p. 1286-99.
8. Dijkman, P.M., et al., *Structure of the merozoite surface protein 1 from Plasmodium falciparum.* Sci Adv, 2021. **7**(23).
9. Holder, A.A., et al., *A malaria merozoite surface protein (MSP1)-structure, processing and function.* Mem Inst Oswaldo Cruz, 1992. **87 Suppl 3**: p. 37-42.
10. Lin, C.S., et al., *The merozoite surface protein 1 complex is a platform for binding to human erythrocytes by Plasmodium falciparum.* J Biol Chem, 2014. **289**(37): p. 25655-69.
11. O'Donnell, R.A., et al., *Functional conservation of the malaria vaccine antigen MSP-119across distantly related Plasmodium species.* Nat Med, 2000. **6**(1): p. 91-5.
12. Herrera, S., et al., *A conserved region of the MSP-1 surface protein of Plasmodium falciparum contains a recognition sequence for erythrocyte spectrin.* EMBO J, 1993. **12**(4): p. 1607-14.
13. Baldwin, M.R., et al., *Merozoite surface protein 1 recognition of host glycophorin A mediates malaria parasite invasion of red blood cells.* Blood, 2015. **125**(17): p. 2704-11.
14. Das, S., et al., *Processing of Plasmodium falciparum Merozoite Surface Protein MSP1 Activates a Spectrin-Binding Function Enabling Parasite Egress from RBCs.* Cell Host Microbe, 2015. **18**(4): p. 433-44.
15. *WHO recommends R21/Matrix-M vaccine for malaria prevention in updated advice on immunization.* 2023.
16. Rts, S.C.T.P., *Efficacy and safety of RTS,S/AS01 malaria vaccine with or without a booster dose in infants and children in Africa: final results of a phase 3, individually randomised, controlled trial.* Lancet, 2015. **386**(9988): p. 31-45.
17. *World Health Organization. Malaria vaccine: WHO position paper.* Weekly Epidemiol Rec, 2016. **91**: p. 33–52
18. Marsh, K. and S. Kinyanjui, *Immune effector mechanisms in malaria.* Parasite Immunol, 2006. **28**(1-2): p. 51-60.
19. Miura, K., *Progress and prospects for blood-stage malaria vaccines.* Expert Rev Vaccines, 2016. **15**(6): p. 765-81.
20. Boyle, M.J., et al., *Human antibodies fix complement to inhibit Plasmodium falciparum invasion of erythrocytes and are associated with protection against malaria.* Immunity, 2015. **42**(3): p. 580-90.
21. Burns, A.L., et al., *Targeting malaria parasite invasion of red blood cells as an antimalarial strategy.* FEMS Microbiol Rev, 2019. **43**(3): p. 223-238.
22. Singh, S., et al., *Immunity to recombinant plasmodium falciparum merozoite surface protein 1 (MSP1): protection in Aotus nancymai monkeys strongly correlates with anti-MSP1 antibody titer and in vitro parasite-inhibitory activity.* Infect Immun, 2006. **74**(8): p. 4573-80.
23. Malkin, E., et al., *Phase 1 study of two merozoite surface protein 1 (MSP1(42)) vaccines for Plasmodium falciparum malaria.* PLoS Clin Trials, 2007. **2**(4): p. e12.
24. Thera, M.A., et al., *Safety and allele-specific immunogenicity of a malaria vaccine in Malian adults: results of a phase I randomized trial.* PLoS Clin Trials, 2006. **1**(7): p. e34.

25. Ogutu, B.R., et al., *Blood stage malaria vaccine eliciting high antigen-specific antibody concentrations confers no protection to young children in Western Kenya.* PLoS One, 2009. **4**(3): p. e4708.
26. Woehlbier, U., et al., *Antibodies against multiple merozoite surface antigens of the human malaria parasite Plasmodium falciparum inhibit parasite maturation and red blood cell invasion.* Malar J, 2010. **9**: p. 77.
27. Blank, A., et al., *Immunization with full-length Plasmodium falciparum merozoite surface protein 1 is safe and elicits functional cytophilic antibodies in a randomized first-in-human trial.* NPJ Vaccines, 2020. **5**(1): p. 10.
28. Kauth, C.W., et al., *The merozoite surface protein 1 complex of human malaria parasite Plasmodium falciparum: interactions and arrangements of subunits.* J Biol Chem, 2003. **278**(25): p. 22257-64.
29. *GUIDELINE ON CLINICAL EVALUATION OF NEW VACCINES.* EMEA/CHMP/VWP/164653/2005, 2005.
30. Cockburn, I.A. and R.A. Seder, *Malaria prevention: from immunological concepts to effective vaccines and protective antibodies.* Nat Immunol, 2018. **19**(11): p. 1199-1211.
31. Toxicology, L.o.P.a., *Report: Examination of an eluate of MSP-1 on pyrogenic properties in rabbits- according to EP 2.6.8.* 2016.
32. Cook, I.F., *Best vaccination practice and medically attended injection site events following deltoid intramuscular injection.* Hum Vaccin Immunother, 2015. **11**(5): p. 1184-91.
33. Costy-Berger, F., *[Preventive rabies vaccination using vaccine prepared from human diploid cells].* Dev Biol Stand, 1978. **40**: p. 101-4.
34. Ajjan, N., et al., *[Results of preventive rabies vaccination with a concentrated vaccine of the PM/WI38-1503-3M rabies strain cultured on human diploid cells. Preparation of mixed antirabies-antitetanus hyperimmune immunoglobulin by plasmapheresis of blood taken from vaccinated veterinary students].* Dev Biol Stand, 1978. **40**: p. 89-100.
35. Nkurunungi, G., et al., *Population differences in vaccine responses (POPVAC): scientific rationale and cross-cutting analyses for three linked, randomised controlled trials assessing the role, reversibility and mediators of immunomodulation by chronic infections in the tropics.* BMJ Open, 2021. **11**(2): p. e040425.
36. Stelekati, E. and E.J. Wherry, *Chronic bystander infections and immunity to unrelated antigens.* Cell Host Microbe, 2012. **12**(4): p. 458-69.
37. Nouatin, O., et al., *Exploratory analysis of the effect of helminth infection on the immunogenicity and efficacy of the asexual blood-stage malaria vaccine candidate GMZ2.* PLoS Negl Trop Dis, 2021. **15**(6): p. e0009361.
38. Kabagenyi, J., et al., *Urban-rural differences in immune responses to mycobacterial and tetanus vaccine antigens in a tropical setting: A role for helminths?* Parasitol Int, 2020. **78**: p. 102132.
39. Mbarak H, R.M., Ali A, *Manual of Reference Intervals and Grading of Selected Abnormal Values, BRTC_RITox_001_V3.2.* 2023.
40. Jongo, S.K., K. Abdul, U, *BRTC_RITox_001_V3.1. Manual of Reference Intervals and Grading of Selected Abnormal Values.* 2022.
41. *Toxicity Grading Scale for Healthy Adult and Adolescent Volunteers Enrolled in Preventive Vaccine Clinical Trials.* U.S. Department of Health and Human Services, Food and Drug Administration, Center for Biologics Evaluation and Research 2007.
42. *ICH E9 statistical principles for clinical trials. .* European Medicines Agency., 1998.
43. Schulz, K.F., et al., *CONSORT 2010 statement: updated guidelines for reporting parallel group randomised trials.* BMJ, 2010. **340**: p. c332.

15 APPENDICES

15.1 Laboratories analysing biological materials

**Institution**	Swiss Tropical and Public Health Institute
**Laboratory**	Department of Medical Parasitology and Infection Biology<br>Clinical Immunology Unit
**Address**	Kreuzstrasse 2<br>4123 Allschwil, Switzerland
**Contact person**	Prof Claudia Daubenberger<br>claudia.daubenberger@swisstph.ch

**Institution**	Sumaya Biotech GmbH & Co. KG
**Laboratory**	Sumaya Biotech GmbH & Co. KG
**Address**	Vangerowstrasse 20<br>69115 Heidelberg, Germany
**Contact person**	Dr. Andrea Aschenbrenner<br>aschenbrenner@sumaya-biotech.com

**Institution**	University of Heidelberg
**Laboratory**	Centre for Infectious Diseases, Parasitology
**Address**	Im Neuenheimer Feld 324<br>69120 Heidelberg, Germany
**Contact person**	Prof. Michael Lanzer<br>Michael.Lanzer@med.uni-heidelberg.de

**Institution**	Ifakara Health Institute
**Laboratory**	Bagamoyo Clinical Trial Facility
**Address**	P.O. Box 74,<br>Bagamoyo, Tanzania
**Contact person**	Dr. Ally Olotu<br>aolotu@ihi.or.tz

**Institution**	Fondazione Istituto Nazionale Genetica Molecolare – INGM
**Laboratory**	Padiglione “Romeo ed Enrica Invernizzi”
**Address**	Fondazione IRCCS Cà Granda Ospedale Maggiore Policlinico di Milano<br>Via Francesco Sforza 35, 20122 Milan, Italy
**Contact person**	Prof. Antonio Lanzavecchia<br>lanzavecchia@ingm.org

**Institution**	University of Maryland School of Medicine
**Laboratory**	Department of Microbiology and Immunology<br>Health Sciences Facility III
**Address**	670 West Baltimore St<br>21201 Baltimore, Maryland, USA
**Contact person**	Prof. Joana da Silva<br>jcsilva@som.umaryland.edu

**Institution**	German Cancer Research Center (DKFZ)
**Laboratory**	16 Division of B cell Immunology
**Address**	Im Neuenheimer Feld 280<br>69120 Heidelberg, Germany
**Contact person**	Prof. Dr. Hedda Wadermann<br>h.wardemann@dkfz.de

**Institution**	Universidad Complutense de Madrid
**Laboratory**	Department of Biochemistry and Molecular Biology, Veterinary Faculty
**Address**	Avda. Puerta del Hierro s/n<br>Ciudad Universitaria. 28040, Madrid, Spain
**Contact person**	Prof. Jose Manuel Bautista Santa Cruz<br>jmbau@ucm.es

**Institution**	Max Delbrück Center for Molecular Medicine
**Laboratory**	Dr. Misha Kudryashev
**Address**	Robert-Rössle-Strasse 10<br>Building 31.2, room 0207<br>13092 Berlin, Germany
**Contact person**	Dr. Misha Kudryashev<br>mikhail.kudryashev@mdc-berlin.de

**Institution**	Pepperprint GmbH
**Laboratory**	Pepperprint GmbH
**Address**	Rischerstrasse 12<br>69123 Heidelberg, Germany
**Contact person**	Dr. Volker Stadler<br>volker.stadler@pepperprint.com

**Institution**	Glyxera GmbH/Max Planck Institute for Dynamics of Complex Technical Systems
**Laboratory**	Dr. Erdmann Rapp
**Address**	Brenneckestrasse 20<br>ZENIT building<br>39120 Magdeburg, Germany
**Contact person**	Dr. Erdmann Rapp<br>e.rapp@glyxera.com

DocuSign

Certificate Of Completion

Envelope Id: D0AAF71567CB49E08FF1FA3F49D4E575

Status: Completed

Subject: MSP1 3 ctp / Corrected Version / DocuSign: CTP_MSP1_v2.0_30.11.2023.pdf

Source Envelope:

Document Pages: 67	Signatures: 6	Envelope Originator:
Certificate Pages: 6	Initials: 0	Mischa Joubert
AutoNav: Enabled		Kreuzstrasse 2,
EnvelopeId Stamping: Disabled		Allschwil, . 4123
Time Zone: (UTC+01:00) Brussels, Copenhagen, Madrid, Paris		mischa.joubert@swisstph.ch
		IP Address: 131.152.225.37

Record Tracking

Status: Original<br>05-Dec-2023 | 15:15	Holder: Mischa Joubert<br>mischa.joubert@swisstph.ch	Location: DocuSign

Signer Events | Signature | Timestamp

Ally Olotu
aolotu@ihi.or.tz
Principal Investigator
Ifakara Health Institute
Security Level: Email, Account Authentication (Required)

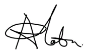

Signature Adoption: Uploaded Signature Image
Signature ID:
5BE062C2-235F-4CA5-803E-77C4D831A97E
Using IP Address: 197.250.15.81

With Signing Authentication via DocuSign password
With Signing Reasons (on each tab):
I approve this document

Sent: 05-Dec-2023 | 15:20
Resent: 07-Dec-2023 | 15:27
Viewed: 07-Dec-2023 | 15:34
Signed: 07-Dec-2023 | 15:34

Electronic Record and Signature Disclosure:
Accepted: 13-Feb-2022 | 18:57
ID: 8c92bce7-3c46-4c02-9ded-47b6703943cd

Claudia Daubenberger
claudia.daubenberger@swisstph.ch
Security Level: Email, Account Authentication (Required)

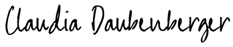

Signature Adoption: Pre-selected Style
Signature ID:
570FB241-D0EF-4659-B902-859A8C59854D
Using IP Address: 131.152.225.35

With Signing Authentication via DocuSign password
With Signing Reasons (on each tab):
I approve this document

Sent: 05-Dec-2023 | 15:20
Viewed: 05-Dec-2023 | 16:07
Signed: 05-Dec-2023 | 16:08

Electronic Record and Signature Disclosure:
Accepted: 19-Aug-2021 | 16:14
ID: f7be9a29-9ecd-4191-8afc-7e294adc1fb1

Signer Events	Signature	Timestamp
Daniel Paris<br>daniel.paris@swisstph.ch<br>Medical Director and Head, Dept. of Medicine<br>Swiss TPH<br>Security Level: Email, Account Authentication (Required)	Signature Adoption: Pre-selected Style<br>Signature ID:<br>608447B2-DF75-4BD0-B115-E05CD1DA9875<br>Using IP Address: 131.152.225.37<br><br>With Signing Authentication via DocuSign password<br>With Signing Reasons (on each tab):<br>I approve this document	Sent: 05-Dec-2023 | 15:20<br>Viewed: 05-Dec-2023 | 16:10<br>Signed: 05-Dec-2023 | 16:10
**Electronic Record and Signature Disclosure:**<br>Accepted: 09-Nov-2017 | 08:45<br>ID: 211fcdff-1764-4c34-bfce-0c930be0616b		
Ernst Böhnlein<br>boehnlein@sumaya-biotech.com<br>Security Level: Email, Account Authentication (Required)	Signature Adoption: Pre-selected Style<br>Signature ID:<br>4829AB79-E23C-41EE-9467-89C0866152F0<br>Using IP Address: 81.89.201.173<br><br>With Signing Authentication via DocuSign password<br>With Signing Reasons (on each tab):<br>Ich genehmige dieses Dokument	Sent: 05-Dec-2023 | 15:20<br>Viewed: 05-Dec-2023 | 16:03<br>Signed: 05-Dec-2023 | 16:05
**Electronic Record and Signature Disclosure:**<br>Accepted: 15-Feb-2022 | 14:55<br>ID: d6109176-6664-4fa2-a686-be32e82b94db		
Fiona Vanobberghen<br>fiona.vanobberghen@swisstph.ch<br>Security Level: Email, Account Authentication (Required)	Signature Adoption: Pre-selected Style<br>Signature ID:<br>99EE8064-61C7-40FC-BC3B-84712F5365C8<br>Using IP Address: 89.226.218.71<br>Signed using mobile<br>With Signing Authentication via DocuSign password<br>With Signing Reasons (on each tab):<br>I have reviewed this document	Sent: 05-Dec-2023 | 15:20<br>Viewed: 06-Dec-2023 | 11:52<br>Signed: 06-Dec-2023 | 11:53
**Electronic Record and Signature Disclosure:**<br>Accepted: 03-Apr-2020 | 10:50<br>ID: f82a2752-b8bd-4e07-854e-f680e88a1249		
Suzanne Gajewski<br>suzanne.gajewski@swisstph.ch<br>Clinical Research Manager<br>Swiss TPH<br>Security Level: Email, Account Authentication (Required)	Signature Adoption: Drawn on Device<br>Signature ID:<br>C8AE5322-6B55-485F-88AF-2971BBD20CB5<br>Using IP Address: 131.152.225.37<br><br>With Signing Authentication via DocuSign password<br>With Signing Reasons (on each tab):<br>I have reviewed this document	Sent: 05-Dec-2023 | 15:20<br>Viewed: 05-Dec-2023 | 15:22<br>Signed: 05-Dec-2023 | 15:22

Signer Events	Signature	Timestamp
**Electronic Record and Signature Disclosure:**<br>Accepted: 28-Nov-2017 | 16:58<br>ID: 3b72df2a-7b7c-4b8a-88f2-ff0d04e62748		

In Person Signer Events	Signature	Timestamp

Editor Delivery Events	Status	Timestamp

Agent Delivery Events	Status	Timestamp

Intermediary Delivery Events	Status	Timestamp

Certified Delivery Events	Status	Timestamp

Carbon Copy Events	Status	Timestamp
Meera Saxena<br>meera.saxena@swisstph.ch<br>Clinical Research Associate<br>Swiss TPH<br>Security Level: Email, Account Authentication (Required)<br>**Electronic Record and Signature Disclosure:**<br>Accepted: 19-Aug-2022 | 10:38<br>ID: b120d76e-8134-45cd-b2ce-1af4b5ddcbc6	COPIED	Sent: 05-Dec-2023 | 15:20

Witness Events	Signature	Timestamp

Notary Events	Signature	Timestamp

Envelope Summary Events	Status	Timestamps
Envelope Sent	Hashed/Encrypted	05-Dec-2023 | 15:20
Certified Delivered	Security Checked	05-Dec-2023 | 15:22
Signing Complete	Security Checked	05-Dec-2023 | 15:22
Completed	Security Checked	07-Dec-2023 | 15:34

Payment Events	Status	Timestamps

Electronic Record and Signature Disclosure

CONSUMER DISCLOSURE

From time to time, Schweizerisches Tropen- und Public Health-Institut (we, us or Company) may be required by law to provide to you certain written notices or disclosures. Described below are the terms and conditions for providing to you such notices and disclosures electronically through the DocuSign, Inc. (DocuSign) electronic signing system. Please read the information below carefully and thoroughly, and if you can access this information electronically to your satisfaction and agree to these terms and conditions, please confirm your agreement by clicking the â€˜I agreeâ€™ button at the bottom of this document.

Getting paper copies

At any time, you may request from us a paper copy of any record provided or made available electronically to you by us. You will have the ability to download and print documents we send to you through the DocuSign system during and immediately after signing session and, if you elect to create a DocuSign signer account, you may access them for a limited period of time (usually 30 days) after such documents are first sent to you. After such time, if you wish for us to send you paper copies of any such documents from our office to you, you will be charged a $0.00 per-page fee. You may request delivery of such paper copies from us by following the procedure described below.

Withdrawing your consent

If you decide to receive notices and disclosures from us electronically, you may at any time change your mind and tell us that thereafter you want to receive required notices and disclosures only in paper format. How you must inform us of your decision to receive future notices and disclosure in paper format and withdraw your consent to receive notices and disclosures electronically is described below.

Consequences of changing your mind

If you elect to receive required notices and disclosures only in paper format, it will slow the speed at which we can complete certain steps in transactions with you and delivering services to you because we will need first to send the required notices or disclosures to you in paper format, and then wait until we receive back from you your acknowledgment of your receipt of such paper notices or disclosures. To indicate to us that you are changing your mind, you must withdraw your consent using the DocuSign â€˜Withdraw Consentâ€™ form on the signing page of a DocuSign envelope instead of signing it. This will indicate to us that you have withdrawn your consent to receive required notices and disclosures electronically from us and you will no longer be able to use the DocuSign system to receive required notices and consents electronically from us or to sign electronically documents from us.

All notices and disclosures will be sent to you electronically

Unless you tell us otherwise in accordance with the procedures described herein, we will provide electronically to you through the DocuSign system all required notices, disclosures, authorizations, acknowledgements, and other documents that are required to be provided or made available to you during the course of our relationship with you. To reduce the chance of you inadvertently not receiving any notice or disclosure, we prefer to provide all of the required notices and disclosures to you by the same method and to the same address that you have given us. Thus, you can receive all the disclosures and notices electronically or in paper format through the paper mail delivery system. If you do not agree with this process, please let us know as described below. Please also see the paragraph immediately above that describes the consequences of your electing not to receive delivery of the notices and disclosures

electronically from us.

How to contact Schweizerisches Tropen- und Public Health-Institut:

You may contact us to let us know of your changes as to how we may contact you electronically, to request paper copies of certain information from us, and to withdraw your prior consent to receive notices and disclosures electronically as follows:

To contact us by email send messages to: andre.lederer@unibas.ch

To advise Schweizerisches Tropen- und Public Health-Institut of your new e-mail address

To let us know of a change in your e-mail address where we should send notices and disclosures electronically to you, you must send an email message to us at andre.lederer@unibas.ch and in the body of such request you must state: your previous e-mail address, your new e-mail address. We do not require any other information from you to change your email address..

In addition, you must notify DocuSign, Inc. to arrange for your new email address to be reflected in your DocuSign account by following the process for changing e-mail in the DocuSign system.

To request paper copies from Schweizerisches Tropen- und Public Health-Institut

To request delivery from us of paper copies of the notices and disclosures previously provided by us to you electronically, you must send us an e-mail to andre.lederer@unibas.ch and in the body of such request you must state your e-mail address, full name, US Postal address, and telephone number. We will bill you for any fees at that time, if any.

To withdraw your consent with Schweizerisches Tropen- und Public Health-Institut

To inform us that you no longer want to receive future notices and disclosures in electronic format you may:

i. decline to sign a document from within your DocuSign session, and on the subsequent page, select the check-box indicating you wish to withdraw your consent, or you may;

ii. send us an e-mail to andre.lederer@unibas.ch and in the body of such request you must state your e-mail, full name, US Postal Address, and telephone number. We do not need any other information from you to withdraw consent.. The consequences of your withdrawing consent for online documents will be that transactions may take a longer time to process..

Required hardware and software

Operating Systems:	WindowsÂ® 2000, WindowsÂ® XP, Windows VistaÂ®; Mac OSÂ® X
Browsers:	Final release versions of Internet ExplorerÂ® 6.0 or above (Windows only); Mozilla Firefox 2.0 or above (Windows and Mac); Safariâ„¢ 3.0 or above (Mac only)
PDF Reader:	AcrobatÂ® or similar software may be required to view and print PDF files
Screen Resolution:	800 x 600 minimum
Enabled Security Settings:	Allow per session cookies

** These minimum requirements are subject to change. If these requirements change, you will be asked to re-accept the disclosure. Pre-release (e.g. beta) versions of operating systems and browsers are not supported.

Acknowledging your access and consent to receive materials electronically

To confirm to us that you can access this information electronically, which will be similar to other electronic notices and disclosures that we will provide to you, please verify that you were able to read this electronic disclosure and that you also were able to print on paper or electronically save this page for your future reference and access or that you were able to e-mail this disclosure and consent to an address where you will be able to print on paper or save it for your future reference and access. Further, if you consent to receiving notices and disclosures exclusively in electronic format on the terms and conditions described above, please let us know by clicking the â€˜I agreeâ€™ button below.
By checking the â€˜I agreeâ€™ box, I confirm that:

- I can access and read this Electronic CONSENT TO ELECTRONIC RECEIPT OF ELECTRONIC CONSUMER DISCLOSURES document; and

- I can print on paper the disclosure or save or send the disclosure to a place where I can print it, for future reference and access; and

- Until or unless I notify Schweizerisches Tropen- und Public Health-Institut as described above, I consent to receive from exclusively through electronic means all notices, disclosures, authorizations, acknowledgements, and other documents that are required to be provided or made available to me by Schweizerisches Tropen- und Public Health-Institut during the course of my relationship with you.
